# *Aedes aegypti* HPX8C modulates immune responses against viral infection

**DOI:** 10.1371/journal.pntd.0007287

**Published:** 2019-04-15

**Authors:** Ju-Mei Wang, Yang Cheng, Zuo-Kun Shi, Xiao-Feng Li, Long-Sheng Xing, Hong Jiang, Dan Wen, Yong-Qiang Deng, Ai-Hua Zheng, Cheng-Feng Qin, Zhen Zou

**Affiliations:** 1 State Key Laboratory of Integrated Management of Pest Insects and Rodents, Institute of Zoology, Chinese Academy of Sciences, Beijing, China; 2 University of Chinese Academy of Sciences, Beijing, China; 3 Department of Virology, State Key Laboratory of Pathogen and Biosecurity, Beijing Institute of Microbiology and Epidemiology, Beijing, China; 4 Guangzhou Eighth People’s Hospital, Guangzhou Medical University, Guangzhou, China; Tulane University School of Public Health and Tropical Medicine, UNITED STATES

## Abstract

Mosquitoes act as vectors of numerous pathogens that cause human diseases. Dengue virus (DENV) transmitted by mosquito, *Aedes aegypti*, is responsible for dengue fever epidemics worldwide with a serious impact on human health. Currently, disease control mainly relies on vector targeted intervention strategies. Therefore, it is imperative to understand the molecular mechanisms underlying the innate immune response of mosquitoes against pathogens. In the present study, the expression profiles of immunity-related genes in the midgut responding to DENV infection by feeding were analyzed by transcriptome and quantitative real-time PCR. The level of Antimicrobial peptides (AMPs) increased seven days post-infection (d.p.i.), which could be induced by the Toll immune pathway. The expression of reactive oxygen species (ROS) genes, including antioxidant genes, such as HPX7, HPX8A, HPX8B, HPX8C were induced at one d.p.i. and peaked again at ten d.p.i. in the midgut. Interestingly, down-regulation of the antioxidant gene HPX8C by RNA interference led to reduction in the virus titer in the mosquito, probably due to the elevated levels of ROS. Application of a ROS inhibitor and scavenger molecules further established the role of oxygen free radicals in the modulation of the immune response to DENV infection. Overall, our comparative transcriptome analyses provide valuable information about the regulation of immunity related genes in the transmission vector in response to DENV infection. It further allows us to identify novel molecular mechanisms underlying the host-virus interaction, which might aid in the development of novel strategies to control mosquito-borne diseases.

## Author summary

HPX8C is a heme-containing peroxidase, which can move reactive oxygen species (ROS) damage to the organism by reducing H_2_O_2_ to H_2_O. Previously, the peroxidase gene has been shown to modulate midgut immunity and regulate anti-malarial response in mosquitoes. In this study, the classical immune signaling pathways, Toll and IMD genes might be late responses against the viruses. HPX8C was demonstrated here to play a role in antiviral immunity against DENV infection in *Ae*. *Aegypti* mosquitoes. HPX8C expression was induced by DENV infection and continued to increase with an elevated virus titer. In HPX8C-depleted mosquitoes, the ROS level was found to be increased with a corresponding decrease in the DENV and ZIKV virus titer. Therefore, it was speculated that HPX8C mediated immune responses against the DENV in the mosquito in the late stage of viral infection, which could be controlled by Toll pathway.

## Introduction

Hematophagous vectors such as mosquitoes transmit a variety of harmful infections that cause devastating diseases, such as malaria, dengue fever, and Zika syndrome [1]. Once infected, a mosquito can transmit pathogens to healthy people for the rest of its life [2]. Mosquitoes, like other insects, do not possess adaptive immunity like that of vertebrates [3]; thus, the innate immune system is essential for controlling parasite and arbovirus infections [4–7]. Although interactions between the pathogens and vectors are complex, an in-depth understanding of this could be helpful in developing pathogen control strategies or new ways to control the vector. Much knowledge has already been acquired from research on anti-*Plasmodium* and anti-bacterial defenses of mosquitoes. In the mosquito fat body, Toll and IMD are two major immune signaling pathways. Activation of the Toll and IMD pathways allows NF-κB factors to enter the nucleus and transcriptionally activate the expression of Antimicrobial peptides (AMPs) and other immunity related genes [8]. AMPs have broad spectrum activity against bacteria, fungi and *Plasmodium* parasites [9]. It has been reported that transgenic mosquitoes co-expressing two or more effector molecules, such as Cecropin A or Defensin A, with synergistic effects on parasites exhibit anti-malarial phenotypes [10]. The JAK-STAT pathway has also been shown to be involved in anti-*Plasmodium* defense [11].

Immune signaling pathways are also universal in antiviral immunity. Toll and the JAK-STAT pathways play essential roles in resistance to ZIKV infection [12]. The RNA interference (RNAi) pathway has also been implicated in the vector immune defense against infecting pathogens, such as chikungunya virus (CHIKV) and dengue virus (DENV) [3,13]. Reports demonstrated that *Ae*. *aegypti* activated the RNAi, JAK/STAT and Toll pathways 10 days post viral infection, thereby limiting the viral infection [14]. C-type lectins (CTL) in arthropods interact with viruses and facilitate the infection [15]. *Mos*GCTL-3 (mosquito galactose-specific C-type lectin-1) was induced by DENV, interacting with its E protein [16]. *Mos*PTP-1 can recruit *mos*GCTL-1 to enhance the viral entry [17]. Microbiota in mosquitoes has been shown to influence DENV infection, and viral infection activates anti-bacterial responses [18]. NS1 proteins have been used to enhance flavivirus acquisition by mosquitoes [19], and a lethal infection with this virus was shown to be prevented by a conserved antiviral mechanism, which may facilitate the transmission of flavivirus in nature [20].

Reactive oxygen species (ROS), such as singlet oxygen, hydrogen peroxides (H_2_O_2_), hydroxyl radical and superoxide are important immune factors utilized by host insects to kill microbes [21–23]. The synthesis and degradation of ROS is a multi-step process and involves an array of enzymes, such as superoxide dismutase (SOD), catalase (CAT) and peroxidases. SOD can catalyze the hydrolysis of superoxide into oxygen and H_2_O_2_. CAT hydrolyses hydrogen peroxide into H_2_O and oxygen, peroxidases, which includes heme peroxidase (HPX), glutathione peroxidase (GPX) and thioredoxin peroxidase (TPX), can also reduce H_2_O_2_ to H_2_O. An elevated level of ROS has been shown to protect *Drosophila melanogaster* from virus-induced mortality and is associated with *Wolbachia*-mediated antiviral immunity [24]. The regulation of ROS balance in mosquitoes is complex. ROS can kill parasites in the midgut and in the hemocoele of the mosquito and ROS deficiency reduces the mosquito resistance to pathogen infection, increasing the mosquito intestinal epithelial damage, and even leading to mosquito death [25,26]. In HPX15 depleted *Anopheles stephensi* mosquitoes, the midgut microbiota was suppressed [27]. HPX2 and NADPH oxidase 5 (NOX5) mediates midgut epithelial nitration and anti-plasmodial defense in mosquito *Anopheles gambiae* [28]. The previous report demonstrated the up-regulated expression of HPX7 and CuSOD2 after infection with Yellow fever virus, DENV, or West Nile virus [29]. Our previous report also indicated that reduction of the ROS level can rescue the defect in infection of zika virus (ZIKV) that loss of the glycosylation site in the mosquito midgut [30].

As a model virus, DENV is suitable for the mechanistic study of antiviral immunity in mosquitoes. In this work, the midgut transcriptomes of immunity-related genes were obtained from DENV-challenged mosquitoes. ROS-regulated antioxidant genes were identified as candidate genes related to DENV infection and the role of HPX8C, a ROS-related gene, in antiviral immunity was further studied. Depletion of HPX8C gave rise to ROS enhancement and was shown to play an important role in antiviral immunity against DENV. Therefore, our results provide valuable information for understanding the evolution of the innate immune system in host insects during pathogen transmission, which can aid in the development of novel approaches for more effective control of mosquito borne diseases.

## Materials and methods

### Ethics statement

The mouse studies were performed in accordance with the guidelines of the Chinese Regulations of Laboratory Animals (Ministry of Science and Technology of People’s Republic of China) and Laboratory Animal-Requirements of Environment and Housing Facilities (GB 14925–2010, National Laboratory Animal Standardization Technical Committee). Other animal experiments and protocols were approved by the Committee on Ethics of Animal experiments of the Institute of Zoology, Chinese Academy of Science (Permit Number: AEI-903-2013). All animal experiments were performed under sodium pentobarbital anesthesia to minimize animal suffering. DENV experiments were performed under biosafety level 2 (BSL2) and animal BSL3 (A-BSL3) containment.

### Animals and cell line

The *Ae*. *aegypti* Rockefeller/UGAL mosquito strain was maintained in laboratory culture. Adult mosquitoes were reared on 10% sugar solution at 27 °C under a photoperiod of 12 h: 12 h (Light: Dark) and 80% relative humidity [31,32]. Specific-pathogen-free BALB/c mice blood were purchased from Beijing Vital River Laboratory Animal Technology (licensed by Charles River). The *Aedes albopictus* cell line C6/36 cells were maintained in RPMI 1640 medium supplemented with 10–20% FBS at 28 °C and 5% (vol/vol) CO_2_.

### Viral infection in the mosquito

The DENV strain used in this study was DENV type 2 strain 43 (GenBank acession number, AF204178.1), which was prepared from culture supernatants of infected mosquito C6/36 cells or infected suckling mouse (BALB/c strain) brain suspensions according to previous procedures [33]. The ZIKV strain used in this study was MR766 NIID strain (GenBank accession number, HQ234498). The plaque assay was performed according to the previous procedures [30]. For infection experiments, the virus diluted in RPMI 1640 medium was mixed with mouse blood (BALB/c strain) at a 1:1 ratio and kept at 37 °C for 30 min (Unless otherwise specified, the viral titer is 10^6^ plaque-forming unit (Pfu)/ml). Then the mixture was fed to 4-day-old mosquitoes through a membrane. Mosquitoes successfully blood fed were maintained in a separate laboratory culture [31,32].

### Sample preparation and RNA extraction

Mosquitoes were dissected in phosphate buffered saline (PBS) and midguts and carcasses (remainder of the mosquito after midgut was removed) were isolated from 25–30 individual mosquitoes [34]. The samples were collected in 0.7 ml RLT buffer (Qiagen) and stored at -80 °C until extraction, at which time they were homogenized using a motor-driven pellet pestle mixer (Kontes). Total RNA was extracted from mosquitoes according to the manufacturer’s instructions using RNeasy Mini Kit (Qiagen). RNA concentration was determined on Molecular Devices Spectramax i3 (Molecular Devices).

### Quantitative real-time PCR

A 10-ng sample of total RNA was used as the template for quantitative real-time PCR (qPCR), which was performed using a One Step SYBR PrimeScript RT-PCR Kit (Perfect Real Time, Takara) on a PIKOREAL 96 qPCR system (Thermo Fisher Scientific). Thermal cycle parameters were 42 °C for 5 min, 95 °C for 10 s, followed by 40 cycles of 95 °C for 5 s, 60 °C for 30 s. Quantitative experiments were performed in biological triplicate, and 40 ribosomal protein 7 (AAEL009496) was used as internal control. Student’s *t*-tests were used to determine the significance of difference in expression between treated and control groups. One way ANOVA followed by a Tukey’s multiple comparison test were conducted to test for pairwise differences. Primers are listed in S3 Table.

### Library construction and Illumina sequencing

The mRNA was purified from total RNA using the mRNA purification kit (Invitrogen) following manufacturer’s instructions. Paired-end RNA-seq libraries were prepared according to the Illumina’s library construction protocol. The libraries were sequenced on Illumina HiSeq 2000 platform (Illumina) in the Beijing Institutes of Life Science (Chinese Academy of Sciences). FASTQ files of raw-reads were produced and sorted by barcodes.

### Bioinformatics analysis

Raw reads obtained from the sequencing were preprocessed using in-house PERL scripts, including adaptor removal and low quality reads filtering. The genome of *Ae*. *aegypti* (accession ID: AaegL1.4) which was downloaded from VectorBase, was used as the annotation file [32]. The clean reads were mapped to the genome using Tophat2 to estimate the expression level of all the transcripts with the default parameters [35,36]. We used cufflinks to calculate the FPKM (fragments per kilobase of transcript per million) of the transcripts, and DEGseq package in R was employed to determine the differentially expressed genes (DEGs) [37]. To gain insight into the tissue-specific transcriptional changes in response to infection by DENV, we performed pair-wise comparisons between libraries to create a union of the DEGs. The genes with *p* values less than 0.05 were regarded as differentially expressed. The immune genes were then assigned based on immunodb [38]. Hierarchical clustering of gene expression levels was performed using Pearson distance as the distance measure with average linkage method [39]. Transcriptome data were deposited to NCBI SRA (accession No. SRP110563).

### Synthesis and micro-injection of double stranded RNA (dsRNA)

dsRNA-mediated gene silencing was used to investigate the role of *Ae*. *aegypti* HPX8C during DENV infection. The dsRNAs were synthesized using cDNA templates possessing T7 RNA polymerase promoter sequences on both ends (S3 Table), according to the protocol of T7 RiboMAX Express RNAi system kit (Promega). Micro-injection of dsRNA was performed as described previously [40]. Female mosquitoes were cold anaesthetized on the Echotherm chilling/heating dry bath (Torrey scientific). A Nanoliter 2000 injector (World Precision Instrument) was used to introduce 0.67 μg per 200 nl dsRNA into the thorax of female mosquitoes 1 day post eclosion [17]. Experiments were conducted under the same conditions and in triplicate. qPCR was performed to confirm the efficiency of gene silencing.

### Protein expression and preparation of antibody

HPX8C was derived from nucleotide position 49–2370 bp in the ORF. The cDNA was amplified by RT-PCR following the user manual of the PrimeScript RT reagent Kit with gDNA Eraser kit. The HPX8C was then inserted into the prokaryotic expression vector pET-28a (+) using FastCloning method [41]. The recombinant plasmid, confirmed by sequencing, was designated as pET-HPX8C. The rabbit polyclonal antibody against HPX8C was produced at the Beijing Genomics Institute.

### Western blotting analysis

Mosquito carcasses, 30 each, were homogenized by pellet pestle in 100 μl of lysis buffer [42]. Aliquots of mosquito protein samples were resolved using SDS-polyacrylamide gels (Bio-Rad) and transferred to PVDF membranes (Merck Millipore). Then, membranes were blocked with Starting Block T20 (PBS) Blocking Buffer (Thermo Fisher Scientific) and incubated with the primary antibody against HPX8C (1:3000). Following three washes with TBS containing Tween-20, the membranes were incubated with goat anti-rabbit horseradish peroxidase-conjugated antibody (Sigma, 1:10000). After washing four times with TBS containing Tween-20, membranes were incubated with SuperSignal West Pico Chemiluminescent Substrate reagent for 5 min (Thermo Fisher Scientific) before visualization. The antibody against α-tubulin (Cell Signaling Technology) was used as the loading control.

### Immunofluorescence staining and confocal microscopy

Dissected midguts were placed on chamber slides and fixed in 4% paraformaldehyde. Samples were blocked in 1% bovine serum albumin (BSA) at room temperature for 1 h before antibody incubations. 4G2 (a mouse monoclonal antibody against the E protein of DENV) and the antibody against HPX8C (1: 50) were used as primary antibodies [43,44]. Alexa Fluor 488 and Alexa Fluor 594 (Invitrogen) were used as secondary antibodies. The Hoechst 33258 (Invitrogen) was added to the last wash for 10 min to stain the nucleus. Subsequently, the chambers were removed from slides, and labeled samples were mounted in Prolong (Invitrogen) for further observations. Images were acquired using a Zeiss LSM 710 Confocal Microscope.

### ROS measurement

The ROS levels in mosquito tissues were detected using a 10 μM solution of the General Oxidative Stress Indicator, CM-H2DCFDA (5-(and-6)-chloromethyl-2’, 7’-dichlorodihydrofluorescein diacetate, acetyl ester, Molecular probes) according to the manufacturer’s instructions. The carcass tissues were incubated with CM-H2DCFDA and were examined under a Zeiss Axioskop 40 with an Axiocam MRC5 using a Zeiss-710 filter set. Identical exposure parameters were used to compare fluorescence levels in different images, which were acquired using a Zeiss LSM 710 Confocal Microscope.

### Peroxidase activity assay

Peroxidase assays were performed using Amplex Red Hydrogen Peroxide/Peroxidase Assay Kit (Invitrogen, A22188), following the manufacturer’s instructions. Briefly, tissues were dissected in about 200 μl of PBS and were homogenized using a motor-driven pellet pestle mixer (Kontes). The mixture was centrifuged at 13000 *g* for 15 min at 4 °C. The supernatant was mixed with 100 μM Amplex Red reagent containing 2 mM H_2_O_2_, and the mixture was kept in the dark at room temperature for 30 min. Fluorescence was then measured at 560 nm (excitation 562 nm) using a microplate reader Spectramax i3 (Molecular Devices). For each data point, the value obtained was deducted from the no-HRP control. Peroxidase content was calculated from the standard curve generated and expressed as U/g of protein.

### SOD activity assays

Total SOD activity was determined using the Superoxide Dismutase assay kit (Hydroxylamine method, Nanjing Jian Cheng Bio-engineering Institute, A001–1). First, tissues were dissected in about 200 μl of PBS and samples were homogenized using a motor-driven pellet pestle mixer (Kontes). Then, the mixture samples were subjected to 13000 *g* for 15 min at 4 °C, and the supernatant was collected following the manufacturer’s instructions. Each sample was analyzed in triplicate. Protein concentration was determined using the BCA protein assay kit (CWbiotech) with BSA as the standard. SOD activity was expressed as U/mg of protein.

### Determination of H_2_O_2_ content

H_2_O_2_ was determined using the Amplex Red hydrogen peroxide/peroxidase assay kit (Invitrogen, A22188). In detail, dissected tissues were homogenized in 400 μl PBS. Then, samples were centrifuged at 13000 *g* for 15 min at 4 °C, and 50 μl of the supernatant was transferred into each microplate well of 96-well plates. An equal amount of mixture of the working solution of 100 μM Amplex Red reagent and 0.2 U/ml HRP was added to each well and kept for 30 min in the dark. Fluorescence was monitored using microplate reader Spectramax i3 (Molecular Devices). Excitation and emission wavelength were 530 nm and 590 nm. H_2_O_2_ content was calculated from the standard curve and expressed as μM/g protein.

### ROS inhibition experiment

ROS scavenger Apocynin (APO) was purchased from Abcam Company. The APO was diluted with DMSO to a working solution with a final concentration of 100 mM. Then, an appropriate volume of APO working solution was added to the virus suspension and BALB/c mouse blood mixture to a final concentration of 300 μM. The ROS generation inhibitor NAC was purchased from Amresco and was diluted with water to a working solution with a final concentration of 613 mM. Then, an appropriate volume of this working solution was added to the virus suspension and mouse blood mixture to a final concentration of 12 mM. Finally, blood feeding was performed according to the above method.

## Results

### Global transcriptome profiling in DENV infected *Ae*. *aegypti* mosquitoes

In order to identify the genes activated in response to viral infection, we performed global transcriptome profiling in *Ae*. *aegypti* mosquitoes after blood feeding. DENV-infected blood meal was provided 4 days post eclosion with different titers of virus (10^3^, 10^4^, 10^5^, 10^6^ and 10^7^ Pfu/ml). Subsequently, the virus titer was determined in the mosquitoes 10 d.p.i. We observed that the DENV level increased significantly in both the midgut and carcass when fed with a blood meal containing a viral titer of 10^6^ Pfu/ml (S1A and S1B Fig). Interestingly, dynamics of the virus titer were found to be different between the mosquito midgut and carcass during the first week. Then, the virus titer significantly increased at 15 days in the midgut, while it kept increasing from 10 days in the carcass (S1C and S1D Fig).

**Fig 1:**
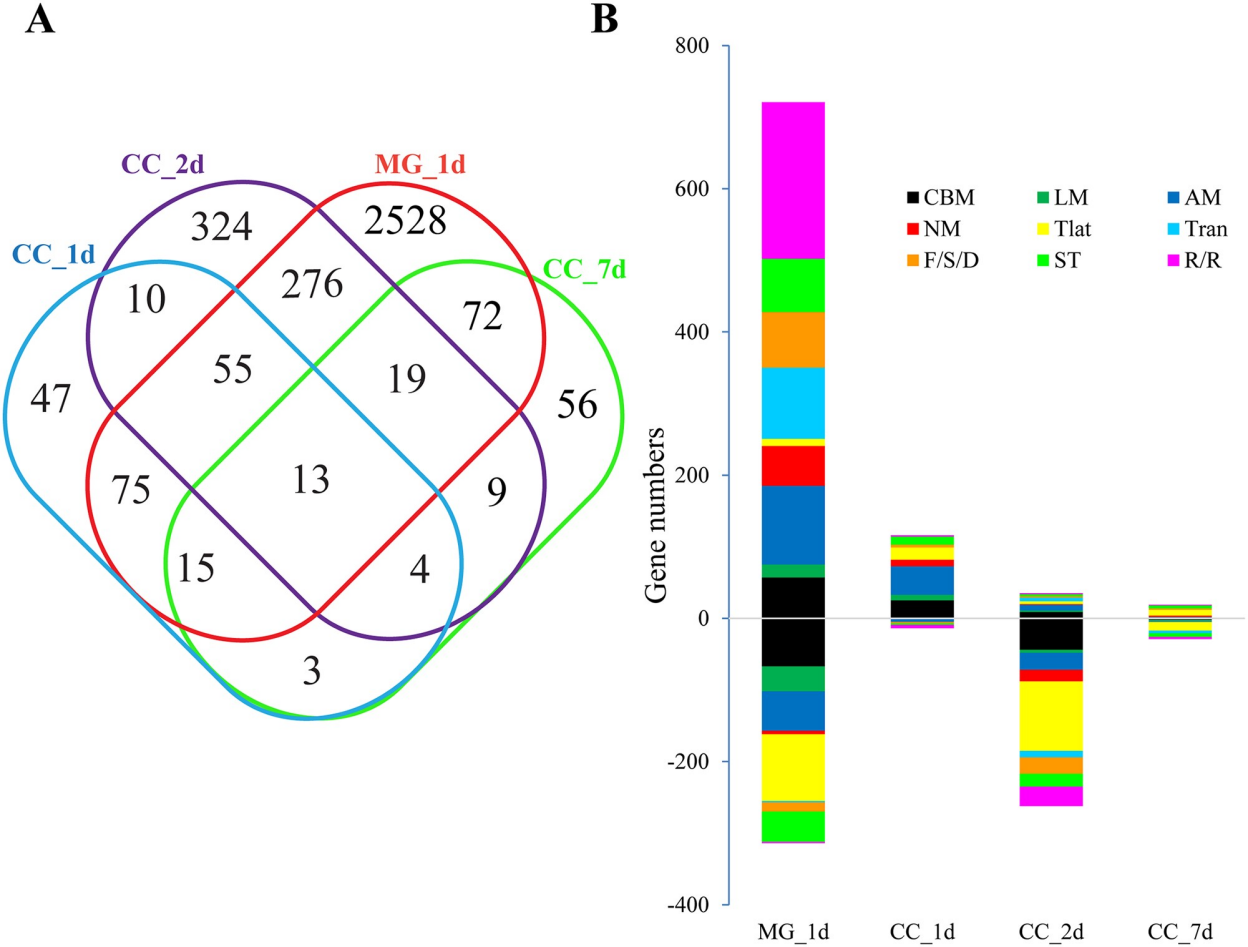
Transcriptional regulation of gene cohorts in response to dengue virus infection. **A**, Venn diagram representing unique and shared transcriptome regulation in DENV-infected mosquito tissues. Hierarchical clustering analysis of DEGs in the midgut and carcass of *Ae*. *aegypti* infected by DENV at 1, 2 and 7 d.p.i., respectively. All genes with *p* values less than 0.05 under at least two experimental conditions were regarded as differentially expressed. The DEGs common to both mosquito tissues were used for Venn diagram. CC_1d, carcass 1 d.p.i.; CC_2d, carcass 2 d.p.i.; CC_7d, carcass 7 d.p.i.; MG_1d, midgut 1 d.p.i. **B**, Functional classification of DEGs in the DENV-infected mosquito midgut and carcass. CBM, carbohydrate metabolism; LM, lipid metabolism; NM, nucleotide metabolism; AM, amino acid metabolism; Tran, transcription; Tlat, translation; F/S/D, folding, sorting and degradation; R/R, replication and repair; ST, signal transduction.

The genome-wide transcription profiling approach was applied to investigate the different immune responses generated in the midgut and carcass of *Ae*. *aegypti* in response to viral infection. The expression profiles of genes in the midgut and carcass were obtained by comparison of the virus challenged transcriptomes at 1, 2 and 7 d.p.i. In total, eight paired-end sequencing libraries were constructed and sequenced. Ultimately, about 92 GB data were obtained, of which each group was represented by at least 20 million reads. All the DEGs were listed in S1 Table. Venn diagram and functional classification analyses were performed to study the commonality and individuality of transcriptome features in the midgut and carcass (S1 Table) (*p*<0.05). More genes displayed change in expression in the midgut 1 d.p.i. (3053 DEGs and 2528 genes were specifically differentially expressed in midgut) than in the carcass (222 DEGs at 1 d.p.i., 710 DEGs at 2 d.p.i., 191 DEGs at 7 d.p.i.). This result suggests that the genes in midgut are more sensitive to viral infection at the early stage, while the mRNA levels of genes in the carcass are less variable against viral infections (S1 Table and Fig 1A).

The genes related to replication and repair, amino acid metabolism and transcription occupy the majority of the up-regulated gene transcripts in the midgut. The other functional pathways affected by viral infection include folding, sorting and degradation, signal transduction, lipid metabolism and nucleotide metabolism. The genes related to amino acid metabolism, carbohydrate metabolism and translation were mostly observed in the carcass 1 d.p.i. However, the gene transcripts that were down-regulated by viral infection 2 d.p.i. in the carcass, were associated with translation, carbohydrate metabolism and replication and repair (Fig 1B).

### Variation in expression of immune response genes after viral infection

To study the host immune response against viral infection, we focused on the expression profiles of immune pathway genes after DENV infection. Transcriptome-based analysis revealed variations in expression of 103 immunity-related gene transcripts in the midgut 1 d.p.i. (*p*<0.05), 56 of which were up-regulated and 47 down-regulated after viral infection. In contrast, in the carcass, only 45 transcripts exhibited change in expression. In particular, only 1 gene transcript was up-regulated and 7 down-regulated at 1 d.p.i.; 7 were up-regulated and 6 down-regulated at 2 d.p.i.; and 14 were up-regulated and 10 down-regulated at 7 d.p.i. Compared with the carcass, a more pronounced change in immunity-related gene transcripts was observed in the midgut (S2 Table).

In the midgut 1 d.p.i., of 73 transcripts encoding clip-domain serine protease (CLIP) genes, 24 were differentially expressed, among which, 22 were up-regulated and only 2 down-regulated by viral infection. In the genes encoding CTL family members, 5 transcripts were up-regulated and the 2 other down-regulated. Eight transcripts encoding Serpin were up-regulated. Almost half of the Galectin (GALE), one-third of the peptidoglycan recognition protein (PGRP) and caspase family gene transcripts were down-regulated by viral infection. Compared with data from 7 days post viral infection in the carcass, the difference in gene expression at 1 day and 2 days was less significant. Only 8 and 13 genes showed differential expression at 1 day and 2 d.p.i., respectively, and 24 genes expressing differentially 7 d.p.i. in the carcass.

**Fig 2:**
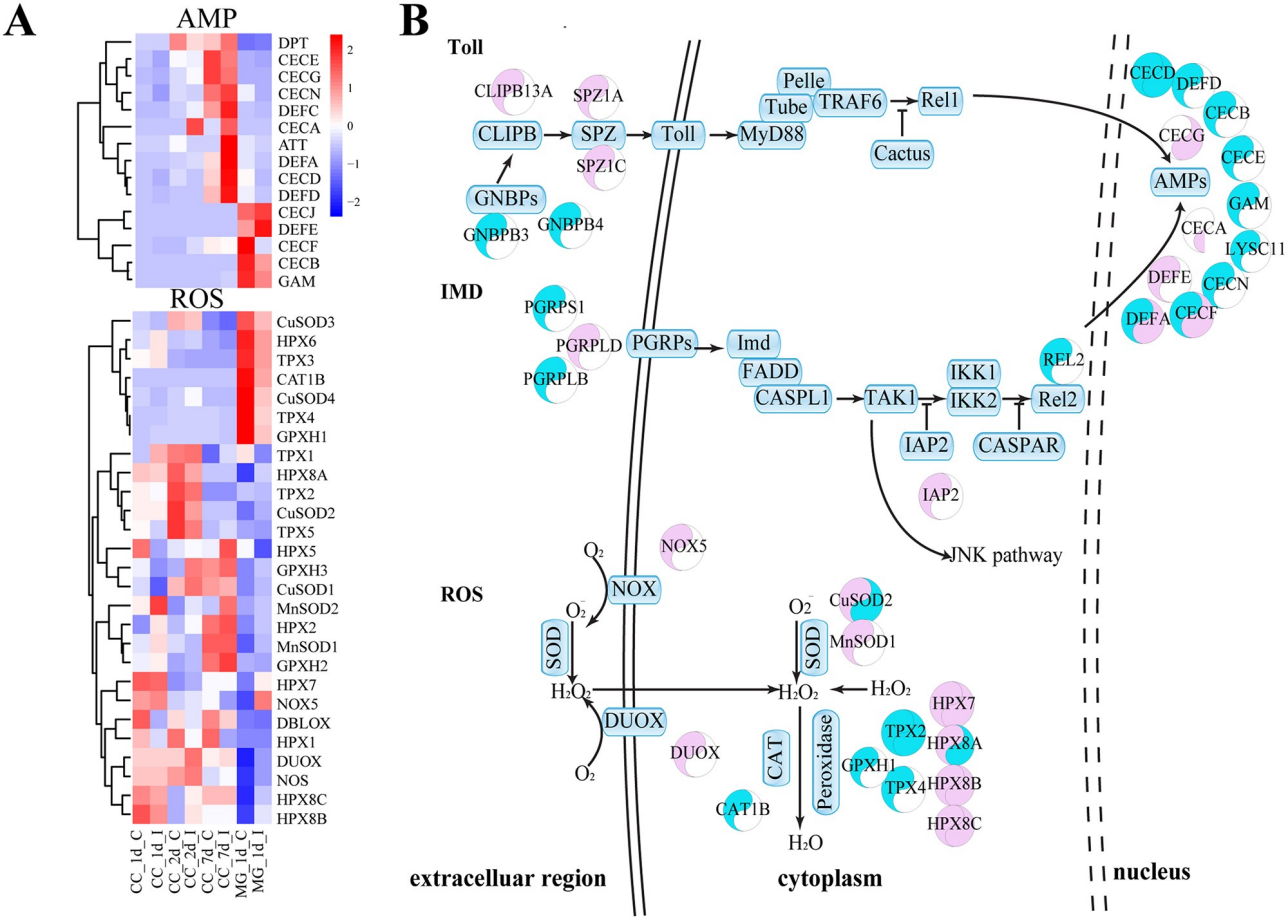
Immune pathways in *Ae*. *aegypti* mosquitoes infected by DENV. **A**, Heatmap of transcriptional variation in expression of immune-related genes (ROS pathways and AMP genes) in DENV-infected mosquitoes. CC_1d_C, carcass 1 day post normal blood meal; CC_1d_I, carcass 1 day post DENV-infected blood meal; CC_2d_C, carcass 2 days post normal blood meal; CC_2d_I, carcass 2 days post DENV-infected blood meal; CC_7d_C, carcass 7 days post normal blood meal; CC_7d_I, carcass 2 days post DENV-infected blood meal; MG_1d_C, midgut 1 day post normal blood meal; MG_1d_I, midgut 1 day post DENV-infected blood meal. **B**, The regulation of immune pathways in *Ae*. *aegypti* in response to viral infection. The left semicircle represents the midguts of mosquitoes infected by DENV 1 d.p.i., the right semicircle represents the carcasses of mosquitoes infected by DENV 2 d.p.i. The color Red represents gene expression increased by DENV infection. The color Blue represents gene expression decreased by DENV infection. The genes with *p* values less than 0.05 were regarded as differentially expressed.

In the midgut, 8 of 15 transcripts encoding AMPs decreased at 1 d.p.i., while only defensin E (DEFE) increased in transcript level. Additionally, CLIPB13A, SPZ1A, SPZ1C, GNBPB3, GNBPB4 and PGRPS1 from Toll and IMD pathway were differentially regulated in the midgut upon viral infection, suggesting the involvement of immune signaling pathway in the antiviral response (Fig 2B). Furthermore, the transcripts of 8 ROS-related genes, such as NOX5, DUOX, CuSOD2, MnSOD1, HPX7, HPX8A, HPX8B and HPX8C, increased in the midgut 1 d.p.i. (Fig 2). Subsequently, the transcriptome data was confirmed by qPCR analysis (S2 Fig).

### ROS-related immune response to DENV infection

Transcriptome-based analysis indicates that expression of ROS-related genes was dramatically up-regulated in DENV-infected midguts. Of 27 transcripts of ROS-related genes, 8 were up-regulated in the midgut 1 day post DENV infection (Fig 2A). Expression levels of four members (HPX7, HPX8A, HPX8B and HPX8C) of the peroxidase family were up-regulated, while two other transcripts encoding TPX2 and TPX4 were down-regulated (Fig 2A). Strikingly, transcription levels of HPX7, HPX8A, HPX8B and HPX8C were up-regulated around 58-, 50-, 45- and 46-fold in the midgut, respectively (S2 Table). Transcription levels of DUOX and CuSOD2 were up-regulated around 32- and 7-fold, respectively (S2 Table). The mRNA levels of TPX4, GPXH1 and CAT1 were down-regulated. In the carcass, the fold changes in transcripts of HPX7, HPX8B and HPX8C were not as significant as in the midgut. The up-regulation of transcripts encoding NOX, SOD, DUOX and peroxidases indicates that the ROS pathway is activated by viral infection (Fig 2A). These indicated that Toll might be involved in the regulation, since previous report revealed that the ROS activation depended on Toll pathway [45].

**Fig 3:**
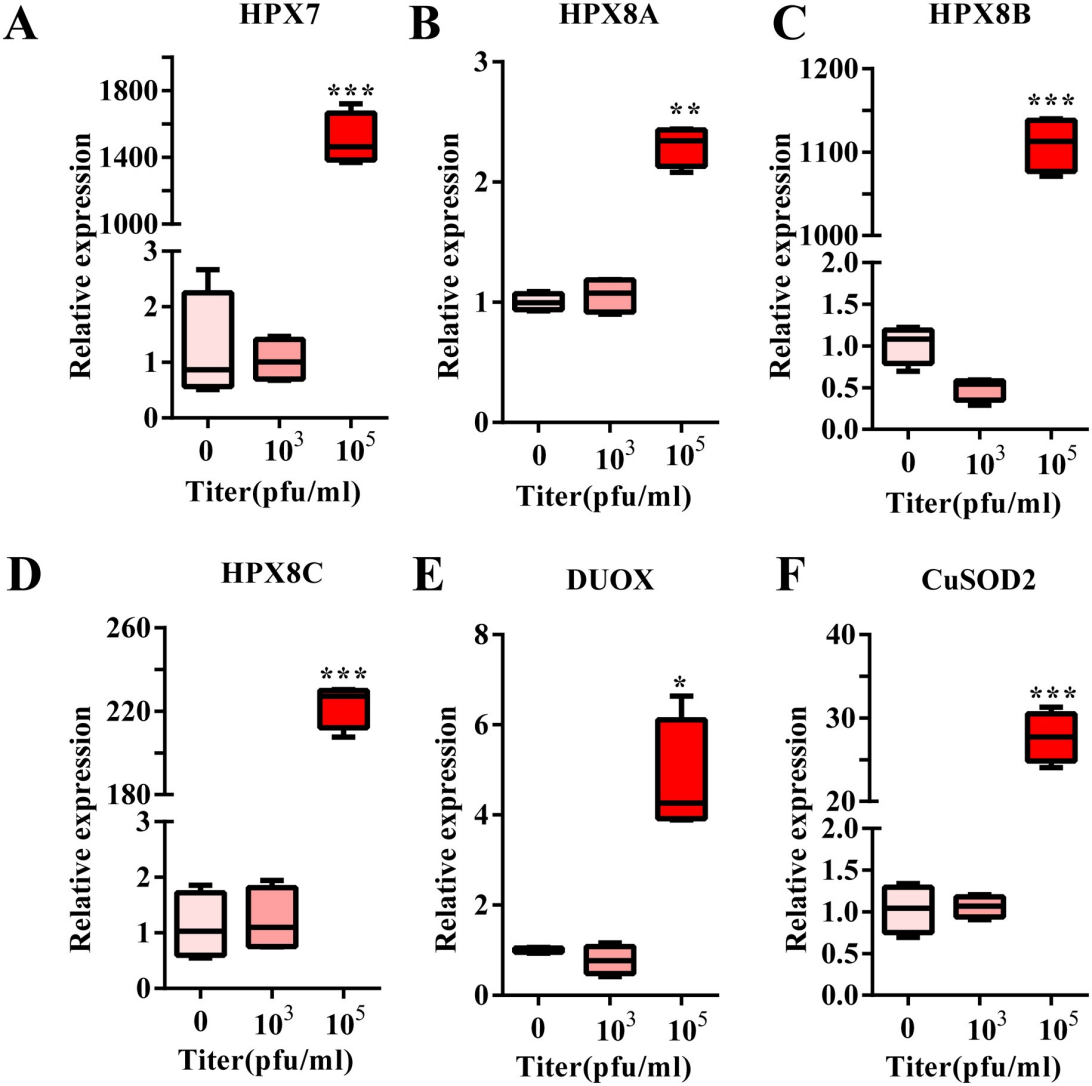
ROS-related gene expression in response to various DENV titers. **A**-**F**, Expression of ROS pathway related genes (**A**, HPX7; **B**, HPX8A; **C**, HPX8B; **D**, HPX8C; **E**, DUOX; **F**, CuSOD2.) at 1 d.p.i. at different titers of DENV in the midgut. The DENV was mixed 1:1 with BALB/c mouse blood (final virus titers: 0, 10^3^, 10^5^ Pfu/ml). All experiments were repeated in triplicate. Student’s t-tests were used to determine the significance of difference in expression between treated and control groups. Data are represented as mean ± SEM; * *p*< 0.05. ** *p*<0.001, *** *p*<0.0001.

**Fig 4:**
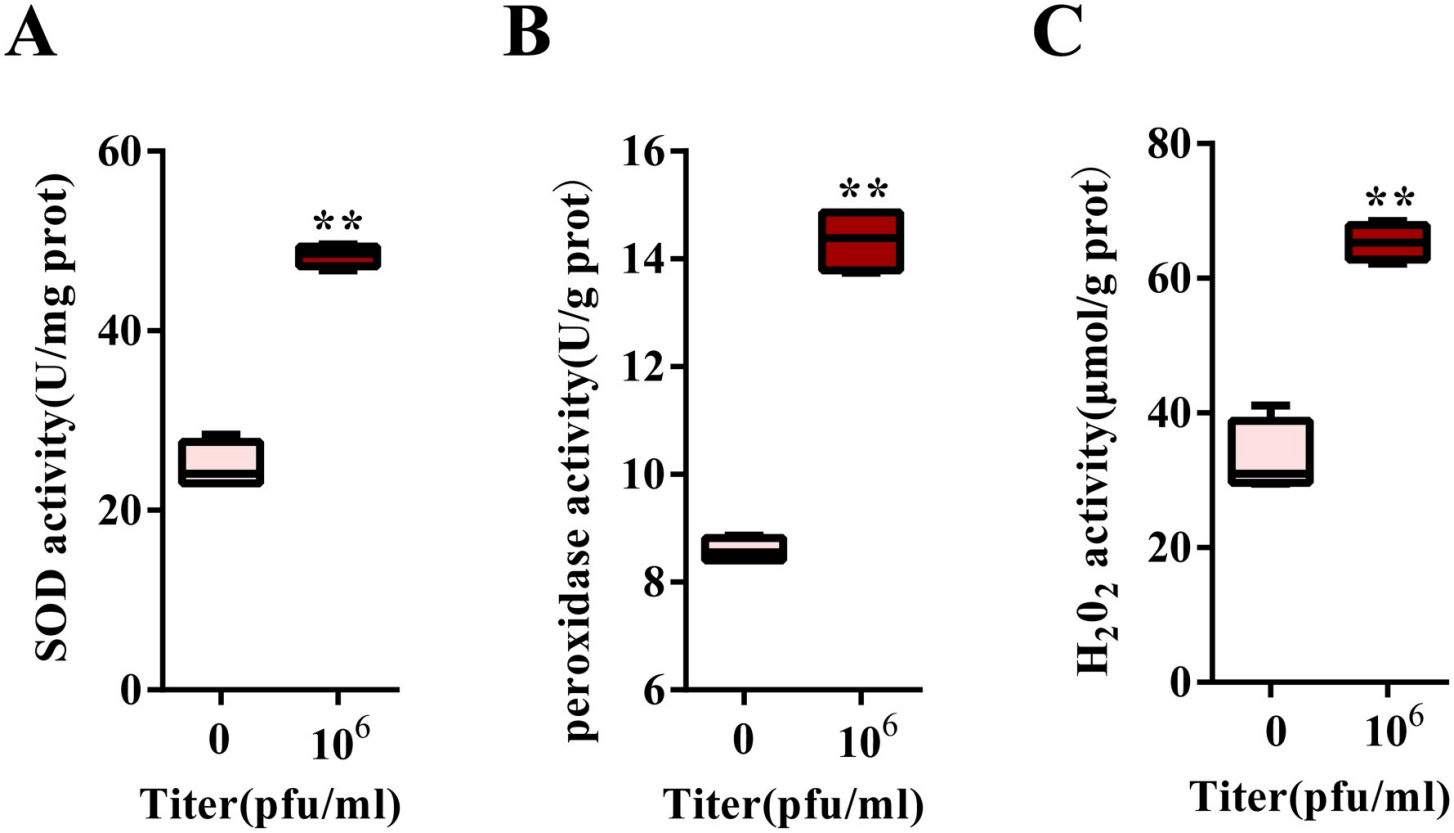
ROS-related activity induced by DENV infection. **A**, SOD activity in the midgut 1 day PBM (normal blood or DENV-infected blood) (mean ± SEM). **B**, Peroxidase activity in the midgut 1 day PBM (normal blood or DENV-infected blood). **C**, Hydrogen peroxide activity in the midgut 1 day PBM (normal blood or DENV-infected blood). All experiments were done in triplicate. Student’s *t*-tests were used to determine the significance of difference in expression between treated and control groups. Data are represented as mean ± SEM; ** *p*<0.01. The DENV virus was mixed 1:1 with commercial BALB/c mouse blood (the final virus titers are 0 and 10^6^ Pfu/ml in RPMI 1640 medium).

To establish the relationship between the mRNA level and DENV titer, we detected the induced level of ROS-related genes under different DENV titer infections. We observed that a DENV titer of 10^3^ Pfu/ml could not induce the up-regulation of any ROS-related genes. However, when the virus titer was increased to 10^5^ Pfu/ml, there was an increase in HPX8A and DUOX2 expression from 2- to 6-fold. Interestingly, DENV infection leads to a 25-fold increase in CuSOD2 expression, while each of HPX7, HPX8B and HPX8C was increased by more than 200-fold (Fig 3). Further, measurement of peroxidase activity at 1 d.p.i., revealed up-regulated activity of SOD, peroxidase and H_2_O_2_ at a titer of 10^6^ Pfu /ml (Fig 4).

**Fig 5:**
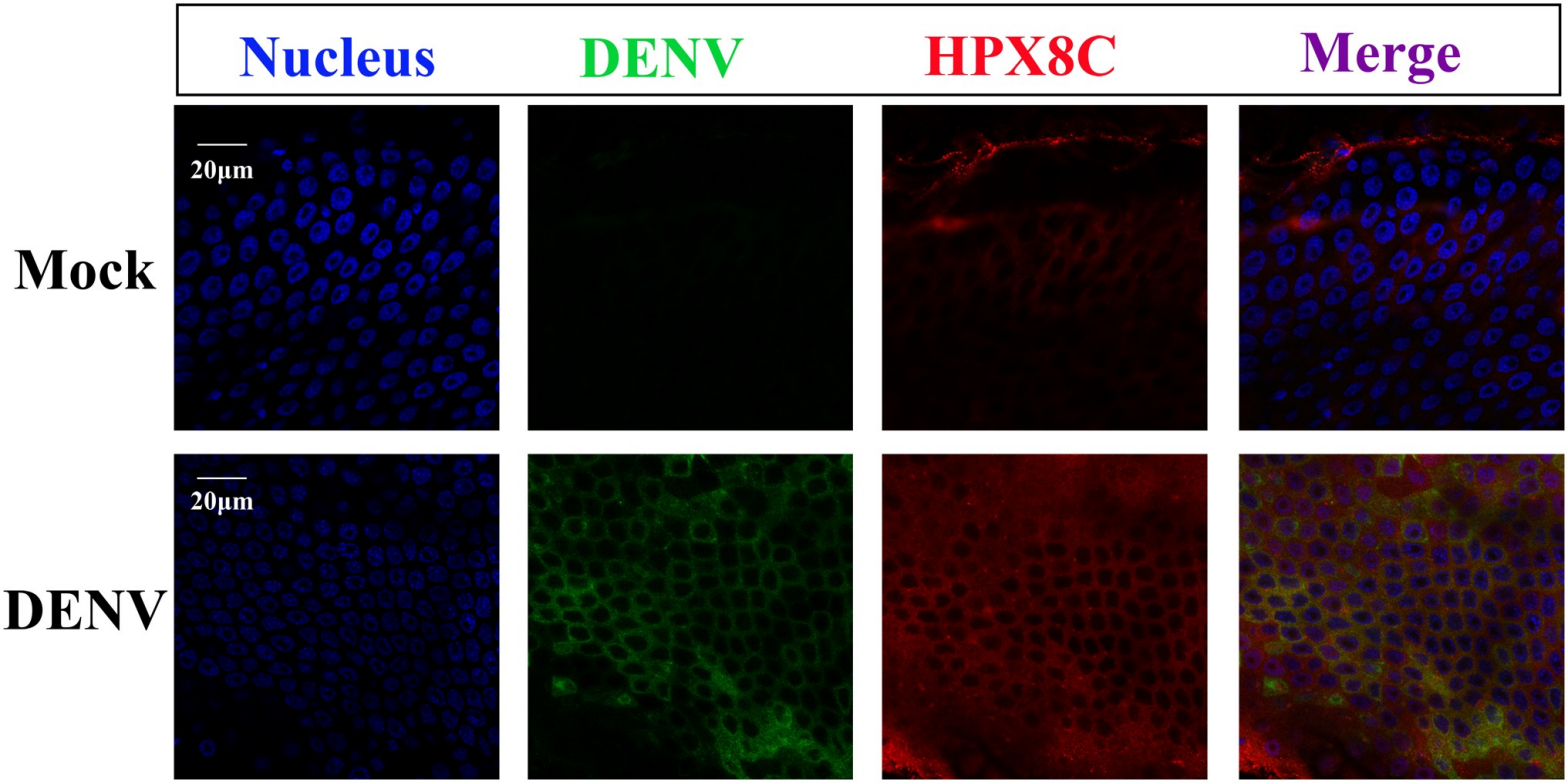
Immunofluorescent staining of DENV-infected midguts 10 days PBM. The midgut cells are stained with antibodies to E protein of DENV (green) or HPX8C (red). The nuclei of the midgut cell is stained with Hoechst (blue).

Next, experiments were designed to assess the temporal relationships between antioxidase immune genes and viral infection in the midgut. The mRNA levels of antioxidant genes were studied 3 hours and at 1, 2, 3, 7, 10 and 15 d.p.i. using a qPCR method. Results showed that ROS-related genes (HPX7, HPX8A, HPX8B, HPX8C, CuSOD2 and DUOX) reached their peak levels in the midgut at 1 and 10 d.p.i. (S3 Fig). The qPCR results of these genes at 1 d.p.i. are consistent with the results of the transcriptome data in the midgut. However, the gene expression was less variable in the carcass during the DENV infection (S3 Fig). The immune-fluorescence method was also used to detect the expression of HPX8C and DENV in mosquitoes post DENV infection (Fig 5), and results demonstrate that the level of the virus increased significantly in the DENV-infected mosquito midgut 10 days post blood meal (PBM). Similarly, the protein expression level of HPX8C in DENV infection group was higher than control in the midgut. This result is consistent with the expression of mRNA level of HPX8C above. Therefore, HPX8C may play an important role in the immune response against DENV infection in the mosquito midgut.

### The role of the antioxidant HPX8C in immunity against viral infection

**Fig 6:**
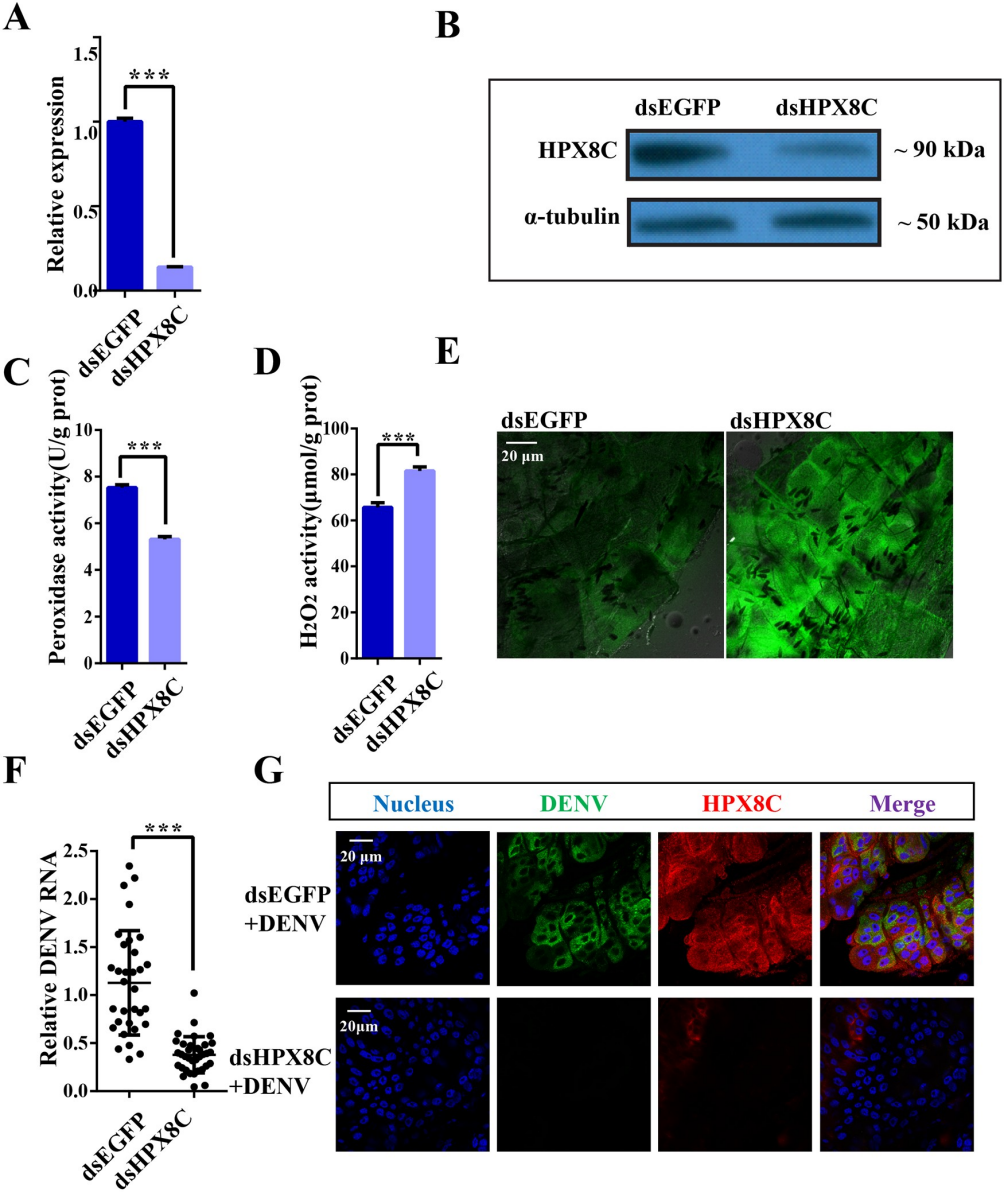
ROS is involved in the anti-dengue virus defense. **A**, HPX8C mRNA expression at 1 day PBM in the midgut of mosquitoes injected with dsEGFP or dsHPX8C. Student’s *t*-tests were used to determine the significance of difference in expression between treated and control groups. Data are represented as mean ± SEM, *** *p*<0.001. Each treatment group includes 30 mosquitoes. The results were accumulated from three independent experiments. **B**, HPX8C protein expression in mosquitoes injected with dsEGFP or dsHPX8C. **C**, Peroxidase activity measured in the mosquito injected with dsEGFP or dsHPX8C. Peroxidase content was expressed as U/g protein. Student’s *t*-tests were used to determine the significance of difference in expression between treated and control groups. Data are represented as mean ± SEM, *** *p*<0.001. **D**, Measurement of H_2_O_2_ level in the mosquito injected with dsEGFP or dsHPX8C. H_2_O_2_ content was expressed as μM/g protein. Student’s *t*-tests were used to determine the significance of difference in expression between treated and control groups. Data are presented as mean ± SEM, *** *p*<0.001. **E**, Measurement of ROS level in the mosquitoes injected with dsEGFP or dsHPX8C. **F**, Effect of RNAi mediated-HPX8C depletion on DENV viral infection in the mosquito 10 days PBM (13 days post RNAi). Student’s *t*-tests were used to determine the significance of difference in expression between treated and control groups. Data were produced using qPCR and presented as mean ± SEM, *** *p*<0.001. Each treatment group comprised 30 mosquitoes. **G**, Immunofluorescent staining of DENV-infected midguts with E protein and HPX8C antibody 10 days PBM. The midgut cell nuclei are stained with Hoechst (blue).

In order to study the role of HPX8C in the immune defense against viral infection, RNA interference (RNAi) method was used to down-regulate HPX8C by injecting dsRNAs into thoraces of female mosquitoes 1 day post eclosion following a micro-injection procedure. Three days later, the mRNA expression level of HPX8C was detected by means of qPCR to be significantly decreased (by 86%), indicating high efficiency of RNAi (Fig 6A, S4 Fig). The mRNA expression level of HPX8C also showed significant reduction in the carcass of HPX8C-depleted mosquitoes. The depletion in HPX8C protein level was further confirmed by western blotting (Fig 6B).

HPX8C-depleted mosquitoes were homogenized for determination of peroxidase and H_2_O_2_ activities. Compared with the control group, peroxidase activity reduced by ~27.28%-30.90% (Fig 6C), while H_2_O_2_ level in HPX8C-depleted mosquitoes increased by ~21.44%-26.60% (Fig 6D). Additionally, the carcass of HPX8C-depleted mosquitoes was dissected for detection of ROS activity under a confocal microscope using a CM-H2DCFDA assay kit. Compared with mosquitoes injected with dsEGFP, HPX8C-depleted mosquitoes showed higher ROS activity (Fig 6E). To study the effect of the decreased ROS level on viral infection, we measured the DENV titer in HPX8C RNAi-treated mosquitoes. In the midgut, the virus titer was found to be 66.42% lower in these HPX8C-depleted mosquitoes than in the control group (Fig 6F). This result was confirmed using immunofluorescence staining and confocal microscopy (Fig 6G).

**Fig 7:**
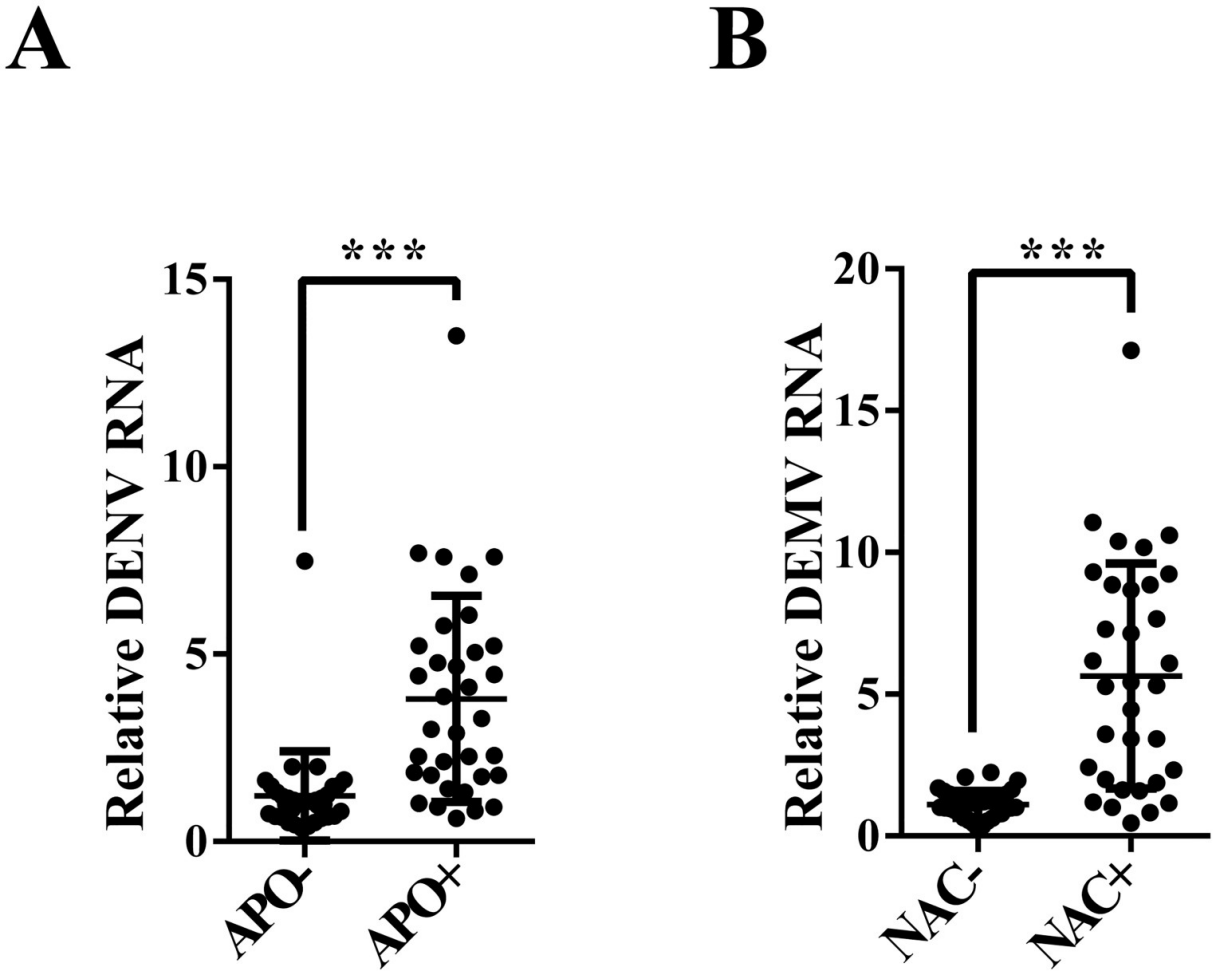
Reduced levels of ROS affect the anti-dengue virus defense. **A**, Effect of treatment with ROS scavenger APO on DENV infection in the mosquito 10 days PBM (13 days post RNAi) (mean ± SEM). **B**, Effect of ROS inhibitor NAC treatment on DENV infection in the mosquito 10 days PBM (13 days post RNAi) (mean ± SEM). Student’s t-tests were used to determine the significance of difference in expression between treated and control groups. *** *p*<0.001. Each treatment group comprised 30 mosquitoes.

**Fig 8:**
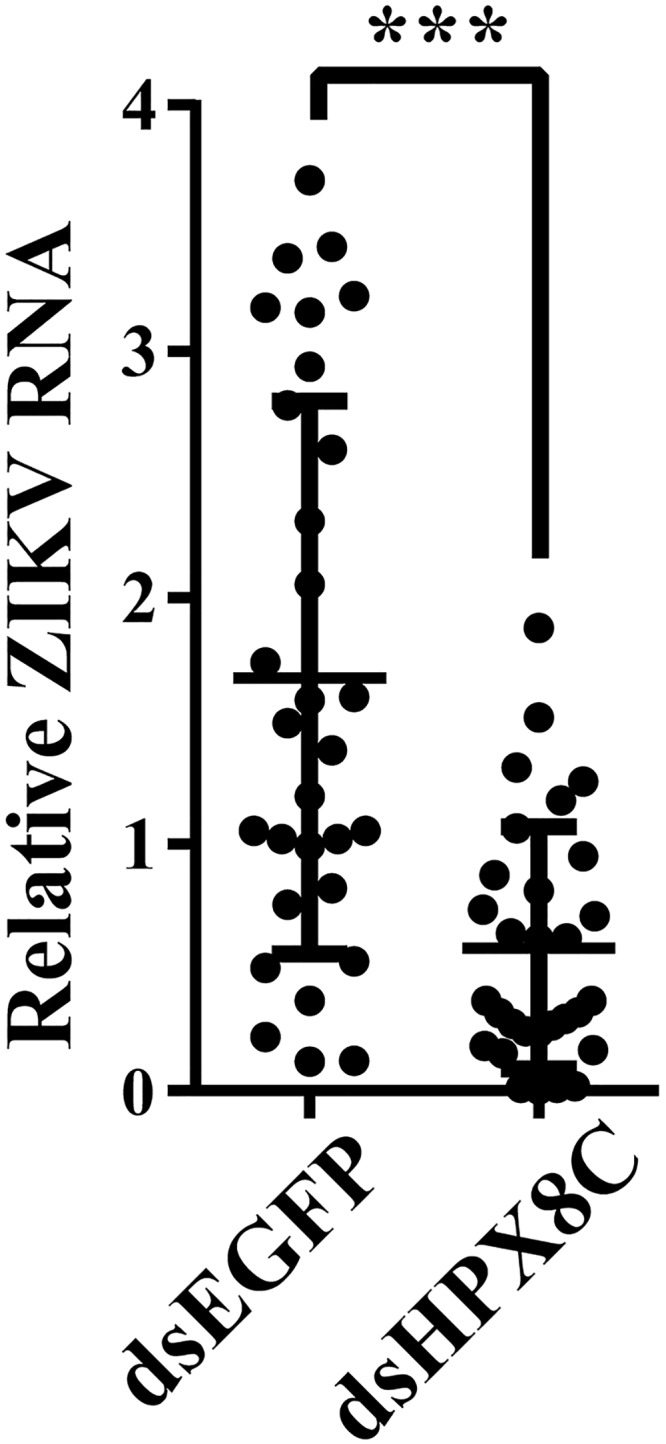
HPX8C is involved in the anti-Zika virus defense. Effect of RNAi mediated-HPX8C depletion on ZIKV infection in the mosquito 7 days PBM (mean ± SEM). Student’s t-tests were used to determine the significance of difference in expression between treated and control groups. *** *p*<0.001. Each treatment group comprised 30 mosquitoes.

The ROS scavenger APO is a selective NADPH oxidase inhibitor that prevents the production of the ROS. Another ROS inhibitor, N-acetyl-L-cysteine (NAC), contains thiol (-SH), which upon oxidation can reduce the disulfide bond in the biological macromolecules and remove ROS at the same time. In our study, the HPX8C-depleted mosquitoes were treated with APO and NAC followed by infection with DENV. In the APO-treated group, the viral titer in the whole body was 2.12 times higher than in the control group (Fig 7A), and was 4.07 times higher in the NAC-treated group (Fig 7B). From the above results, it can be speculated that ROS may play an important role in the DENV infection process. In order to investigate whether the impact of HPX8C applies to other viruses, we measured the ZIKV titer in HPX8C RNAi-treated mosquitoes. Interestingly, ZIKV titer in these HPX8C-depleted mosquitoes decreased by 65.85% than in the EGFP-depleted group (Fig 8).

## Discussion

The expression of genes regulated by viral infection has been shown previously to be tissue-specific [29,46]. The midgut is the first line of mosquito defense from invading pathogens. Our study showed that the virus titer in the midgut decreased significantly at 1 d.p.i. (S1 Fig), indicating that host genes may trigger an anti-dengue immune response to reduce the virus titer. Thus, the time point of 1 d.p.i. was chosen for future experiments. Additionally, immune responses to DENV in the carcass at early (1 day and 2 days) and late stages (7 days) were also studied. We then conducted RNA sequencing to reveal the gene transcripts of *Ae*. *aegypti* affected by DENV infection.

Previous studies have indicated the role of the Toll pathway in regulation of DENV infection in *Ae*. *aegypti* mosquitoes [47]. The JAK-STAT pathway has also been suggested to play a pivotal role in anti-dengue response in mosquitoes, independent of the Toll and RNAi pathways [48]. AMPs form a line of immune defense against pathogen invading in the host [49], and expression levels of AMP genes were found to be decreased in both midgut (8 of 15 AMP genes) and carcass (5 of 15 AMP genes) at 1 d.p.i. (Fig 2A), and then subsequently increased in the carcass (8 of 15 AMP genes) at 7 d.p.i., consistent with previous reports [29]. It appears that Toll pathways play a significant role at the late stage of viral infection. This result is consistent with previous findings [47]. The phenoloxidase (PO) cascade has been shown to play a key role in defense against Semliki Forest virus in mosquitoes [50], although its role in antiviral immunity of mosquitoes is unclear. In our study, prophenoloxidase1 (PPO1), PPO3 and PPO5 were induced by the DENV in the mosquito midgut at 1 d.p.i. (S2 Table). However, understanding the specific mechanism behind PO-mediated antiviral immunity needs further investigation.

In our current study, peroxidases and SODs were found to be induced by viral infection in the early stage, and their expression varied in a dose-dependent manner with increasing DENV titer (Fig 4). These results are similar to previous reports demonstrating up-regulated expression of HPX7, HPX8A, HPX8B, HPX8C and CuSOD2 at 1 day after infection with Yellow fever virus, DENV or West Nile virus [29]. Similarly, we also observed an increase in HPX8A, HPX8B and HPX8C expression after DENV infection in the midgut 1 and 10 d.p.i. (S3 Fig). Previous report indicated that elevated ROS level are controlled by Toll pathway [45]. It was speculated that peroxidases and SODs were involved in responses against the DENV in the mosquito. In mice, another peroxidase, GPX-1, can reduce the pulmonary inflammatory response caused by influenza A virus [51]. In airway epithelial cells, increasing antioxidant can significantly affect respiratory syncytial virus-associated oxidative cellular signaling and cell damage [52]. In contrast, the expression of ROS-related genes (HPX2, HPX7, HPX8A, HPX8B, HPX8C, MnSOD1 and TPX2) was inhibited in the *Ae*. *aegypti* midgut 1 day after infection with *Plasmodium gallinaceum*, whereas DBLOX, DUOX1, TPX3 and TPX2 levels were induced [11]. Therefore, it might be suggested that the function of ROS-related genes was different in anti-malarial response and anti-DENV immunity.

ROS generated in response to viral infection in epithelial cells can lead to lung injury by inducing oxidative stress and inflammation [53]. Mitochondrial ROS can regulate the sensitivity of mosquito intestinal epithelium to *Plasmodium* infection [54]. In our study, the DENV and ZIKV viral titer in the HPX8C-depleted mosquitoes decreased significantly (Figs 6F and 6G and 7C). The level of HPX8c affects both DENV and ZIKV levels, indicating that the role of HPX8C is universal. In the HPX8C-depleted mosquitoes, there was an increase in ROS activity (Fig 6D and 6E).

Alternatively, reduction in the ROS activity by ROS scavenger or ROS inhibitor resulted in a higher DENV titer than the control group (Fig 7A and 7B). These findings imply ROS was related to antivirus immunity. ROS is also a major anti-pathogenic molecule induced by pulmonary influenza viral infections [55]. In this study, the antioxidants and ROS have been found to be associated with the early stage of antiviral immunity. Accumulation of ROS induced by dsHPX8C also affects ZIKV levels in mosquitoes. Our previous report indicated that reduction of the ROS level can rescue the defect in infection of E glycosylation site deficient mutant ZIKV in the mosquito midgut [30]. This result provides new research clues for our research. In the future, we will explore how ROS and HPX8C affect the DENV and the molecular mechanism underlying HPX8C-mediated antiviral responses, thus providing a new theoretical basis for antiviral immunity and virus control.

## Accession numbers

RUNX4: EU604102; *Mos*GCTL-3: AAEL000535; *mos*GCTL-1: AAEL000563; *Mos*PTP-1: AAEL013105; NS1 proteins of DENV1 (GZ/XNC strain): FJ176780; NS1 proteins of DENV3 (ThD3 strain): AY676352; NS1 proteins of DENV type 2 strain 43: AF204178.1; NS1 proteins of ZIKV MR766 NIID strain: HQ234498; 40S ribosomal protein 7: AAEL009496. The other accession numbers are shown in S1 Table.

## Supporting information

S1 Table DEGs in the midgut and carcass post DENV infection. (PDF)

S2 Table Immune DEGs post DENV infection. (PDF)

S3 Table Primers used for qPCR and synthesis of dsRNAs. (PDF)

S1 Fig DENV virus in the midgut and carcass post DENV infection. **A**, The DENV titer dynamics in the midgut 10 d.p.i. (post-infection). **B**, The DENV titer dynamics in the carcass 10 d.p.i. **C**, The DENV titer dynamics in the midgut post infection. **D**, DENV titer dynamics in the carcass post infection. Each treatment group comprised 30 mosquitoes. Identical letters are not significant difference (*p* > 0.05), while different letters indicate significant difference (*p* < 0.05) determined by one way ANOVA followed by a Tukey’s multiple comparison test. All experiments were repeated in triplicate. Data are represented as mean ± SEM.
(TIF)

S2 Fig AMP genes induced by DENV infection. The mRNA levels of *attacin* (ATT) (A), *cecropin* A (CECA) (B), *defencin* C (DEFC) (C) and *gambicin* 1 (D) 1 day post DENV infection in the midgut. The mRNA levels of *attacin* (ATT) (E), *cecropin* A (CECA) (F), *defencin* C (G) and *gambicin* 1 (H) 1 day post DENV infection in the carcass. All experiments were repeated in triplicate. Student’s t-tests were used to determine the significance of difference in expression between treated and control groups. Data are represented as mean ± SEM. * *p*<0.05, ** *p*<0.01, *** *p*<0.001.
(TIF)

S3 Fig Temporal variation in mRNA expression of ROS pathway genes in the DENV-infected mosquitoes. mRNA levels of HPX8C(A), HPX7(B), HPX8A(C), HPX8B(D), CuSOD2(E), DUOX(F) were detected using qPCR post 10^6^ Pfu /ml viral infection in *Ae*. *aegypti* midgut. The mRNA levels of HPX8C(G), HPX7(H), HPX8A(I), HPX8B(J), CuSOD2(K), DUOX(L) were detected using qPCR post 10^6^ Pfu/ml viral infection in *Ae*. *aegypti* carcass. Total RNA was isolated from the midgut or carcass of mosquitoes at seven time points post viral infection. The control is healthy BALB/c mouse blood mixed with RPMI 1640 medium. Identical letters are not significant difference (p > 0.05), while different letters indicate significant difference (*p* < 0.05) determined by one way ANOVA followed by a Tukey’s multiple comparison test. All experiments were repeated in triplicate. Data are represented as mean ± SEM.
(TIF)

S4 Fig The efficiency of HPX8C RNAi. HPX8C mRNA expression at 1 day PBM in mosquitoes carcass injected with dsEGFP or dsHPX8C. All experiments were repeated in triplicate. Student’s t-tests were used to determine the significance of difference in expression between treated and control groups. Data are represented as mean ± SEM. * *p*<0.05.
(TIF)
