# Supplementary material for: Aedes aegypti HPX8C modulates immune responses against viral infection
Source: PLoS Negl Trop Dis. 2019 Apr 15;13(4):e0007287. doi: 10.1371/journal.pntd.0007287 (PMC6464178; doi:10.1371/journal.pntd.0007287)
Supplement: S1 Table — (PDF) [file pntd.0007287.s001.pdf]

S1_Table. DEGs in the midgut and carcass post DENV infection (A union of DEGs in the midgut and carcass post DENV infection (p<0.05)).

gene_id	Name	CC_1d_C	CC_1d_I	CC_2d_C	CC_2d_I	CC_7d_C	CC_7d_I	MG_1d_C	MG_1d_I
AAEL004676	(s)-2-hydroxy-acid oxidase	3.87111	3.93576	8.63243	8.68273	17.6695	12.9273	90.2663	37.9054
AAEL005035	1,4-dihydroxy-2-naphthoate octaprenyltransferase	18.7914	21.1232	15.5013	15.6223	17.2524	18.71	5.41493	12.0859
AAEL006885	14-3-3zeta	278.775	284.117	250.087	228.156	309.989	312.396	789.028	468.35
AAEL010399	1-acylglycerol-3-phosphate acyltransferase	33.9263	56.4738	33.7794	34.0979	53.1303	43.9873	47.7603	46.1609
AAEL006321	1-acylglycerol-3-phosphate acyltransferase	17.2484	15.1407	22.8393	25.9546	27.0626	21.3652	64.1843	25.2902
AAEL011898	1-acyl-sn-glycerol-3-phosphate acyltransferase	43.8957	43.3115	5.43332	9.14915	6.24364	4.89792	360.498	223.445
AAEL003006	2-deoxyglucose-6-phosphate phosphatase	8.83919	9.97297	30.7836	54.7097	16.9668	13.8827	21.6359	10.376
AAEL005400	2-hydroxyacid dehydrogenase	88.8045	89.7429	127.257	105.909	83.2964	83.7314	65.399	67.5078
AAEL010235	30 kDa salivary gland allergen Aed a 3 Precursor	0.53186	0.119732	0.783058	0.391204	0.093936	0.102639	6.92205	0.576563
AAEL008006	3-hydroxyacyl-coa dehyrogenase	25.1243	26.4269	14.1222	44.3966	6.46595	6.9204	464.483	254.639
AAEL006684	3-hydroxyisobutyrate dehydrogenase	54.552	50.6424	48.7919	50.6766	44.788	43.6298	26.5486	37.3272
AAEL008933	3-hydroxyisobutyrate dehydrogenase	4.05392	3.89404	8.3974	1.78523	5.16731	3.89623	0.325692	1.8632
AAEL011249	4-hydroxybenzoate octaprenyltransferase	31.5002	30.1581	36.991	34.5111	22.9437	20.866	14.8699	24.2127
AAEL003746	4-Hydroxybutyrate CoA-transferase, putative	86.9119	91.8326	107.704	112.057	65.0767	60.7179	113.783	109.9
AAEL010442	4-hydroxyphenylpyruvate dioxygenase	5.81904	17.1955	0.316541	0.514758	2.53491	1.88133	0.350959	4.05893
AAEL007090	4-nitrophenylphosphatase	0.42823	0.40282	0.466095	0.358659	0.389822	1.18498	373.481	139.71
AAEL010099	4-nitrophenylphosphatase	0.581809	0.552986	2.32991	3.03128	7.78041	4.36777	267.538	110.162
AAEL007097	4-nitrophenylphosphatase	2696.42	2215.16	6636.55	3622.54	935.35	1017.91	34.7894	1404.31
AAEL007463	5,10-methylenetetrahydrofolate reductase, putative	15.5349	16.6192	7.06242	0	7.76637	7.56651	29.4458	21.665
AAEL017904	5.8S_rRNA	0	0	0	0	0	0	0	216.978
AAEL008925	5-formyltetrahydrofolate cyclo-ligase	5.08628	5.33633	7.06456	4.96075	9.13437	8.90087	0.649227	4.10632
AAEL010694	5-formyltetrahydrofolate cyclo-ligase	17.0652	21.5295	25.0439	23.522	30.8614	32.4563	3.15215	14.8224
AAEL004728	5-methyltetrahydrofolate:homocysteine methyltransferase	136.42	149.922	28.0507	24.5822	5.62257	4.4617	27.9963	98.5917
AAEL014715	67 kDa polymerase-associated factor PAF67, putative	23.5205	35.5419	19.31	15.0805	45.9167	45.4641	24.5248	33.7217

AAEL005446	6-phosphofructo-2-kinase/fructose-2,6-bisphosphatase	34.4717	32.5839	48.8198	59.2906	26.9905	27.0963	10.6291	20.8356
AAEL013132	6-phosphofructo-2-kinase/fructose-2,6-bisphosphatase short form	56.7523	52.6302	62.8146	57.6047	51.5228	53.1555	38.8578	43.7157
AAEL001642	6-pyruvoyltetrahydropterin synthase, putative	57.2732	56.4922	74.9531	68.3828	48.9192	52.4141	27.0768	40.4073
AAEL009871	80 kda MCM3-associated protein	15.1721	14.5105	19.446	15.0679	20.1449	20.6169	3.9558	10.9056
AAEL013179	8-oxoguanine DNA glycosylase	4.18039	5.43502	2.60329	3.44846	3.11328	3.25314	0.350177	4.29039
AAEL008431	a kinase anchor protein	87.5652	87.8968	110.425	116.848	85.1101	92.3673	34.3865	49.3896
AAEL008134	abc transporter	19.6904	21.4492	15.3064	16.8136	34.528	31.7864	310.665	172.023
AAEL008629	abc transporter	13.1108	12.9758	6.69266	4.34709	7.36198	6.50179	548.744	277.461
AAEL008632	abc transporter	6.72101	7.30183	8.21793	8.50546	12.8173	10.1071	163.179	72.428
AAEL005491	abc transporter	1.5971	1.86031	4.9849	22.5308	3.4108	1.97802	2.59782	2.19455
AAEL013372	abc transporter	11.0335	7.8628	17.8116	49.5856	8.90582	7.72676	0.59932	4.62222
AAEL014428	abc transporter	13.1376	10.5539	2.34071	3.72588	3.77969	3.62635	0.845313	5.44866
AAEL006634	acetyl-coa acetyltransferase, mitochondrial (acetoacetyl-coa thiolase)	60.7542	94.6843	52.9049	38.5685	67.8605	57.0149	72.2614	67.1843
AAEL006307	acetyl-coa carboxylase	99.6515	111.723	77.786	83.116	112.218	95.2982	27.6328	53.5035
AAEL003903	acid phosphatase-1	87.3432	83.3273	90.2318	71.181	42.0658	46.8424	165.311	125.319
AAEL010482	acidic membrane protein, putative	38.2878	28.9458	19.9623	28.461	33.4642	36.9995	13.3345	33.771
AAEL008216	aconitase	57.8606	55.9532	15.5171	30.7432	52.5775	50.3872	186.438	116.445
AAEL011197	actin	920.489	863.89	1091.58	1000.51	733.046	748.352	5110.94	2251.43
AAEL011750	actin	26.273	25.3204	55.1915	48.0128	16.6344	15.6401	121.03	66.0227
AAEL012310	actin	36.382	35.3001	34.6801	34.2249	29.2874	25.2288	168.071	77.9085
AAEL004616	actin	77.7993	75.81	76.54	74.3945	27.7873	35.4406	596.694	298.307
AAEL011317	actin	25.2647	26.2789	38.9663	34.0696	34.6179	36.1471	13.8835	20.0393
AAEL004646	actin	0.138715	0	0.131454	0.075892	0.254474	0.370131	151.066	55.4105
AAEL013661	actin binding protein, putative	133.605	125.565	220.096	257.937	32.4218	33.981	585.51	345.807
AAEL005424	actin binding protein, putative	102.546	80.9836	178.078	145.78	81.3068	75.124	29.4386	64.6738
AAEL011972	actin binding protein, putative	40.2678	43.1675	31.0509	25.0363	35.9273	40.4326	187.743	91.1577
AAEL007546	actinlated protein 2/3 complex subunit 1A	43.8203	45.8232	48.1674	46.5838	56.2426	57.2731	374.85	170.904
AAEL001711	activin receptor type I, putative	20.7601	19.802	19.0846	19.4965	19.4019	18.9835	6.26433	13.4038
AAEL007555	acyl-coa dehydrogenase	62.0017	90.2358	16.3648	11.8423	30.9545	34.6188	40.414	62.3521
AAEL004739	acyl-coa dehydrogenase	56.094	60.7301	75.9965	119.53	64.1384	57.8725	73.5126	57.0963
AAEL008574	acyl-CoA oxidase	25.9415	27.1291	18.0168	20.1387	26.8467	21.2334	658.207	326.019

AAEL008841	acyl-CoA oxidase	4.31102	5.07967	3.14108	3.92567	7.46103	6.45365	160.687	71.5247
AAEL003569	acyl-coa thioesterase	10.0048	9.73181	12.8002	15.4925	15.7596	16.4802	60.0692	23.8336
AAEL005992	adam (a disintegrin and metalloprotease	0.391161	0.359409	5.71591	15.2563	3.44887	0.654253	9.34046	3.15946
AAEL005364	adaptin, alpha/gamma/epsilon	1.23398	1.11176	1.37398	1.32475	2.06492	1.74944	75.6704	31.8642
AAEL005662	adenosine diphosphatase	55.1602	58.8617	54.5054	60.1675	53.5391	52.9348	206.481	101.439
AAEL001856	adenosine kinase	29.4101	28.6145	43.1595	37.2102	18.8757	17.9785	8.05156	16.6387
AAEL001102	adenosine kinase	88.1867	93.5266	117.913	113.508	198.602	199.315	57.2054	67.356
AAEL005383	Adenosine monophosphate-protein transferase FICD homolog	47.4617	41.5805	53.6896	72.8428	21.5778	23.9558	7.45375	27.524
AAEL008341	adenosylhomocysteinase	66.3543	74.11	58.7641	51.8471	161.388	164.112	38.6609	53.7422
AAEL005177	adenylate cyclase	26.8325	28.39	33.5096	33.0777	14.0486	15.461	190.711	102.437
AAEL009314	adenylate cyclase	1.35791	0.753159	91.0577	25.3825	2.61328	3.24937	0.679218	0.84256
AAEL001687	adenylate cyclase	0.654551	0.288342	0.84136	1.07863	0.70405	0.602728	107.602	42.0307
AAEL001691	adenylate cyclase	33.837	30.9515	21.1743	30.4605	27.5221	28.447	6.40537	17.062
AAEL012731	adenylate kinase 1, putative	79.0981	83.5514	51.5533	40.3597	99.8673	99.3894	47.8565	72.8252
AAEL005605	adenylsulfate kinase	26.378	27.3627	19.6339	25.7325	13.2055	13.3405	162.824	85.7798
AAEL011821	adenylyl cyclase-associated protein	23.6675	22.9147	30.6362	16.9281	16.6635	17.3854	253.463	114.735
AAEL013074	adenylyl cyclase-associated protein	29.5594	27.7199	39.6721	25.3152	21.5307	18.9935	283.547	105.229
AAEL014717	adiponectin receptor	75.1139	110.648	98.2327	232.019	102.259	131.802	236.389	130.532
AAEL004855	adp,atp carrier protein	938.076	1012.7	939.151	800.491	2219.3	2156.17	1477.98	1153.86
AAEL007209	ADPbosylation factor, putative	31.1411	30.232	63.5988	55.9045	23.9413	21.0789	117.573	55.2566
AAEL014379	adrenodoxin	54.3481	67.1709	28.6785	27.5762	66.1565	60.0478	376.472	177.112
AAEL004393	afadin (af-6 protein)	9.56498	10.6542	11.4634	10.7304	12.1243	12.0641	51.2285	18.0893
AAEL009872	alanine aminotransferase	33.1065	30.8782	62.414	110.052	42.3967	36.9063	232.674	138.465
AAEL009875	alanine aminotransferase	98.8901	117.866	16.3625	8.74336	31.6748	40.5229	2.41271	39.0252
AAEL005821	alanyl aminopeptidase	0.183469	0.084826	0.081499	0.070663	1.18874	2.01975	398.891	194.386
AAEL005808	alanyl aminopeptidase	1.67812	1.73783	0.226992	0.224038	1.34978	0.688957	218.791	97.5525
AAEL011130	alcohol dehydrogenase	25.6957	9.45769	22.45	64.2899	43.2978	34.5682	4.81002	1.5656
AAEL012457	alcohol dehydrogenase	0.522052	0.205146	1.38625	1.40434	1.31787	2.12041	192.89	66.1818
AAEL011126	alcohol dehydrogenase	8.31078	3.94485	14.8713	31.0742	10.6451	8.91807	0.803701	0.598906
AAEL015034	alcohol dehydrogenase	29.3683	27.4435	43.1489	41.975	15.8022	18.8063	7.80694	25.7123
AAEL011756	aldehyde dehydrogenase	63.6694	94.8477	9.57193	3.53899	29.1936	23.1566	928.601	559.168
AAEL009664	aldehyde dehydrogenase	2.16762	2.29373	2.40905	2.73551	1.10051	1.68028	110.929	57.3745
AAEL006163	aldehyde oxidase	0	0	0.054083	3.7441	0	0	0	0.023408
AAEL010384	aldehyde oxidase	0.82775	0.847586	4.23649	3.07126	2.83905	2.518	148.362	60.2795

AAEL006157	aldehyde oxidase	0	0	1.07641	15.3227	0.022071	0	0.075845	0
AAEL010382	aldehyde oxidase	0.671116	0.360225	1.25057	0.927272	5.46883	2.46606	128.207	57.3939
AAEL009948	ALDH9948	30.235	33.4149	64.391	40.8464	24.6685	22.8673	44.5485	25.4909
AAEL008663	aldo-keto reductase	11.4185	14.1742	8.32742	8.50209	4.61821	4.02841	110.572	55.713
AAEL004088	aldo-keto reductase	107.229	117.544	183.268	225.692	134.543	126.062	326.233	195.21
AAEL009134	aldo-keto reductase	0	0	0.109927	0.063961	1.24675	0.075081	273.57	121.12
AAEL004118	aldo-keto reductase	34.776	41.5388	44.0201	31.2111	33.506	32.5818	414.501	226.345
AAEL004086	aldo-keto reductase	14.1211	13.1771	11.9512	7.95055	14.7616	16.1755	98.7268	48.3256
AAEL003154	aldo-keto reductase	26.4711	13.77	2.81546	2.19018	5.71849	5.20498	4.03467	8.34019
AAEL015291	aldose 1-epimerase, putative	27.0771	29.1052	10.058	11.6011	7.35125	6.82837	3.97134	18.134
AAEL010590	aldose-1-epimerase	6.31034	5.54742	4.70041	4.96364	5.86992	5.66563	102.134	38.2106
AAEL015070	alkaline phosphatase	0	0.033318	0	0.072605	0.129914	0	70.8888	24.4928
AAEL000931	alkaline phosphatase	55.449	45.4973	167	106.349	48.8552	48.9904	34.2567	31.1231
AAEL003309	alkaline phosphatase	9.00228	5.73644	7.70927	7.74325	4.50832	6.30004	2113.86	1058.83
AAEL011175	alkaline phosphatase	0	0.053034	0	0	0.254141	0.184946	185.628	78.7772
AAEL003298	alkaline phosphatase	1.17478	1.05515	0.598223	0.346096	3.60759	6.18346	4879.11	1719.54
AAEL009077	alkaline phosphatase	12.9406	12.1315	44.5844	97.4289	7.53479	8.47036	0.462733	7.51877
AAEL003286	alkaline phosphatase	0.818323	0.825199	0.698219	0.269064	2.07186	3.75093	4565.13	1410.26
AAEL010405	alkyldihydroxyacetonephosphate synthase	1.68955	2.27725	0.795403	0.604869	2.04085	2.34797	57.0121	25.6049
AAEL014045	allantoicase	3.15514	2.74008	7.64747	23.1137	7.45455	11.085	10.9022	5.54359
AAEL005997	allergen, putative	381.027	332.276	223.955	308.398	207.806	202.293	25.4449	163.757
AAEL000244	alpha(1,3)fucosyltransferase	0.540906	0.50375	1.49579	2.80508	1.16322	1.10875	46.0818	20.1217
AAEL009658	alpha,alpha-trehalase	55.2222	55.353	87.8751	58.1537	53.7749	48.6174	83.513	59.414
AAEL000678	alpha-amylase	0.083065	0.065453	0.062616	0.023962	0.049985	0.108791	83.5286	38.0956
AAEL000667	alpha-amylase	5.39572	4.5899	9.60406	8.28175	6.95432	8.20766	1364.71	626.21
AAEL003434	alpha-amylase	2.14971	2.00374	2.23671	2.162	3.12387	3.54174	877.308	350.76
AAEL010536	alpha-amylase	8.87846	9.06497	11.8706	11.2573	7.64501	8.60176	0.726158	4.32646
AAEL000642	alpha-amylase	24.2631	17.6479	2.25845	3.29331	8.68171	9.94221	258.926	117.199
AAEL010389	alpha-esterase	3.02676	3.50062	4.02419	4.31305	2.97368	3.45963	169.881	91.4278
AAEL005188	alpha-galactosidase/alpha-n-acetylgalactosaminidase	0.198187	0.60423	0.206802	0.171487	0.143176	0.156032	1015	1087.81
AAEL005460	alpha-galactosidase/alpha-n-acetylgalactosaminidase	11.0341	9.75191	7.64502	37.7461	9.25019	9.47399	215.418	111.057
AAEL005481	alpha-glucosidase	17.9737	16.9658	37.2684	32.5704	21.1145	22.9111	134.533	64.1394
AAEL004369	alpha-glucosidase	1.39372	0.177614	1.75322	0.738442	0.411098	1.3019	1023.58	413.99

AAEL010653	alpha-l-fucosidase	6.5881	7.36896	2.40793	2.77052	9.35087	7.17641	1299.18	707.448
AAEL005477	amidase	49.0712	47.3802	23.6709	32.7191	46.773	49.2194	369.79	173.849
AAEL003581	amidophosphoribosyltransferase	80.0358	120.197	39.8379	28.2779	72.0696	71.503	11.7669	44.82
AAEL008913	amino acid transporter	0.671157	0.629874	0.117691	0.10935	0.631842	1.3088	390.968	184.382
AAEL007458	amino acid transporter	19.8461	14.0004	29.5538	19.9634	8.50511	8.86694	0.248954	8.94571
AAEL007197	amino acid transporter	36.3213	32.9238	13.2527	11.4431	29.1595	25.2422	10.6304	20.3139
AAEL003970	amino acid transporter	38.258	31.0336	5.78701	35.8846	8.9271	12.8002	502.839	219.415
AAEL007191	amino acid transporter	0.305324	0.397181	0.184498	0.133869	0.442893	1.04969	242.506	124.226
AAEL014161	amino acids transporter	59.6094	64.248	27.816	43.0948	41.8365	44.1042	36.4828	43.856
AAEL010276	aminomethyltransferase	38.8538	68.6871	8.93251	3.94989	47.4294	42.9385	0.952755	15.9536
AAEL000101	AMP dependent coa ligase	20.4687	20.6761	3.0186	4.46905	6.07429	4.9873	230.604	105.317
AAEL000415	AMP dependent coa ligase	50.5984	48.2244	125.614	141.322	37.5682	32.4217	15.8845	29.2842
AAEL000572	AMP dependent ligase	61.5089	53.1677	44.622	70.6621	24.3622	24.3735	14.9135	35.7024
AAEL010853	AMP dependent ligase	0.058303	0	0.230465	0.193001	0.053156	3.6421	0	6.61597
AAEL013611	AMP dependent ligase	1.62345	1.31558	1.95121	2.61115	2.27472	2.92023	1217.3	641.497
AAEL002668	AMP dependent ligase	8.82928	8.36301	22.2758	11.9717	14.6244	13.8305	12.1019	9.1285
AAEL012431	AMP dependent ligase	11.4842	12.2309	20.7847	25.1517	7.72426	7.08226	0.399276	6.19396
AAEL006823	AMP dependent ligase	4.86096	5.69478	4.10523	4.13213	4.31904	3.75582	415.267	195.069
AAEL001501	anamorsin, putative	34.2574	34.6986	35.6602	33.0473	33.4853	28.8386	105.766	53.0119
AAEL014212	androgen induced inhibitor of proliferation (as3) / pds5	32.2176	31.4429	38.8245	32.1788	41.6821	41.3541	14.9739	23.2051
AAEL009310	angiotensin-converting enzyme (dipeptidyl carboxypeptidase	1.30876	1.66685	1.45987	1.39453	1.95056	2.47399	1081.72	1072.24
AAEL008389	ankyrin repeatch membrane-spanning protein	59.3381	55.2955	102.101	79.721	49.262	52.8928	111.387	75.1574
AAEL011302	annexin	109.22	106.16	93.8823	89.3038	99.8654	104.445	361.49	133.743
AAEL005407	annexin x	19.979	17.1789	41.1909	22.8116	17.0166	17.708	4.98002	10.3955
AAEL006511	anopheles stephensi ubiquitin	1236.97	1267.28	611.222	544.215	3568.58	3637.2	2252.19	1871.97
AAEL003921	anopheles stephensi ubiquitin, putative	46.7479	39.8965	69.7223	41.3775	54.1171	55.5766	24.8828	37.1217
AAEL002554	anosmin, putative	65.7843	85.8265	24.8763	29.2773	126.782	119.583	1.4594	39.9848
AAEL001022	anterior fat body protein	40.5815	30.9198	106.254	79.4345	23.6364	23.7507	249.548	107.14
AAEL001020	anterior fat body protein	44.6625	46.6068	41.6539	40.4055	76.1876	76.0188	210.94	103.058
AAEL000757	anterior fat body protein	8.5208	12.6446	8.05973	8.40329	9.57329	11.1277	554.307	285.446
AAEL012830	anti-silencing protein	62.1749	56.5636	100.741	78.9855	25.3678	26.2125	18.5566	35.5712
AAEL010781	ap endonuclease	76.6498	73.1435	154.459	110.285	41.2273	42.6965	18.2347	50.3517
AAEL013063	APG18A	46.6237	34.1112	49.3441	70.1452	9.24261	10.0088	180.482	101.579

AAEL010641	APG7A	54.3223	49.5966	63.6473	63.1594	47.8888	47.0705	26.2316	33.9912
AAEL007162	APG8	254.948	217.945	273.053	330.829	112.562	123.415	1236.71	688.387
AAEL008789	apolipophorin-III, putative	830.989	491.772	338.441	308.573	77.8832	63.333	98.633	255.749
AAEL009569	apolipoprotein D, putative	76.8495	80.8082	85.1005	91.8056	68.427	75.7232	5.95149	43.9352
AAEL009560	apolipoprotein D, putative	15.431	21.6747	4.97041	9.97873	15.0221	24.6741	54.7575	68.1204
AAEL013574	apolipoprotein D, putative	100.935	108.615	32.539	26.7968	101.637	103.098	67.4635	82.2376
AAEL009561	apolipoprotein D, putative	1.24257	1.43926	2.12588	1.61441	3.17675	2.96094	137.441	60.0465
AAEL010986	apyrase, putative	7.43917	6.70834	11.2062	14.1922	7.86904	7.32408	306.617	159.449
AAEL005008	aquaporin	75.5221	138.021	27.7295	7.41057	64.0202	64.2813	132.655	147.275
AAEL003550	aquaporin	28.2255	24.3764	69.5038	56.4976	13.344	18.5428	165.68	75.2071
AAEL007065	Arf1	259.825	282.95	298.043	264.959	203.34	203.25	393.922	273.552
AAEL004236	arf6 guanine nucleotide exchange factor	13.1593	13.8891	13.006	15.1943	11.7803	11.9554	74.0645	32.1964
AAEL002675	arginase	8.31086	14.9102	14.3409	2.71116	90.7445	83.5892	0.403565	5.27258
AAEL009185	arginine or creatine kinase	199.472	189.962	162.699	133.987	73.6938	80.9006	1109.65	707.502
AAEL006473	arginine/serinech splicing factor	72.8332	73.5463	87.7361	85.5537	31.9803	35.8047	35.525	45.7607
AAEL007286	argininech protein, putative	97.5997	136.004	70.5598	69.5147	37.8218	44.5787	113.441	95.0667
AAEL003345	argininosuccinate lyase	579.014	488.256	164.756	240.295	334.213	289.871	14.8374	132.65
AAEL004701	argininosuccinate synthase	9.91927	12.1611	16.5058	8.54195	23.7614	22.6776	0.184114	4.56398
AAEL002887	arm	27.1509	26.4345	18.0794	20.3648	22.9525	22.951	145.834	71.2483
AAEL005461	armadillo repeat protein	33.1552	30.4489	36.8746	33.8988	40.6719	40.8294	11.6055	18.6804
AAEL014238	aromatic amino acid decarboxylase	71.6462	70.672	90.0481	102.06	45.9892	46.9836	1.19455	34.5269
AAEL010735	aromatic amino acid decarboxylase	0.883644	0.920854	0.936422	6.47741	0.554148	0.534787	0	1.08417
AAEL010737	aromatic amino acid decarboxylase	107.945	108.313	143.171	152.246	67.4726	72.5928	2.61819	52.1955
AAEL009059	arp2/3 complex 16 kd subunit (P16-arc)	48.2989	51.6363	46.5743	49.6025	50.3063	47.5573	286.033	152.882
AAEL009041	arp2/3 complex 20 kd subunit	31.7936	32.8623	30.762	28.2969	33.4974	31.7528	109.965	52.4694
AAEL013168	arrowhead	21.0612	18.7004	33.5531	28.4719	10.1493	10.795	0.509882	7.13728
AAEL015631	asparagine synthetase	2.0075	1.48171	1.20869	0.816372	6.01763	5.86483	5.97055	0.424205
AAEL012579	aspartate aminotransferase	91.555	97.74	131.259	125.829	82.0482	80.0009	48.6491	72.4781
AAEL011518	aspartyl-tRNA synthetase	19.644	29.4331	14.1015	15.8001	17.2369	15.7803	26.7751	33.1224
AAEL012175	ATP synthase alpha subunit mitochondrial	348.191	376.591	388.549	347.194	636.018	588.694	613.407	505.571
AAEL008787	ATP synthase alpha subunit vacuolar	64.1472	65.5573	70.7955	73.383	45.0865	48.5256	605.012	311.587
AAEL002827	ATP synthase beta subunit	290.032	302.886	352.276	299.912	353.347	337.74	335.058	305.78
AAEL003393	ATP synthase beta subunit	108.527	127.671	133.505	100.505	286.432	264.916	224.423	170.029
AAEL008790	ATP synthase coupling factor B, putative	12.8184	13.6198	15.4877	14.6166	17.1725	18.0681	5.30769	12.903
AAEL010823	ATP synthase delta chain	84.9839	94.4429	90.2768	71.0006	125.959	126.48	216.729	138.852

AAEL002504	ATP synthase delta chain, mitochondrial	204.479	223.641	218.757	184.33	273.816	267.108	335.458	278.785
AAEL005798	ATP synthase subunit beta vacuolar	61.1741	59.8172	63.7403	63.1386	60.7331	62.3144	420.223	233.386
AAEL009808	ATP synthase subunit d	53.0541	55.5775	49.6179	52.8172	42.4422	45.9259	356.898	191.497
AAEL006256	ATPase subunit, putative	206.653	269.673	172.818	125.795	413.289	342.545	465.439	344.627
AAEL012740	ATPase subunit, putative	667.134	723.711	780.977	660.198	826.72	871.892	1088.52	919.238
AAEL012549	atpase, class vi, type 11c	0.171153	0.24977	0.269081	0.243162	2.25356	1.20389	133.887	47.1897
AAEL015146	ATP-binding cassette sub-family A member 3, putative	4.14698	3.99925	4.27832	3.0948	7.34907	6.21811	58.8154	21.5971
AAEL012702	ATP-binding cassette sub-family A member 3, putative	1.02077	0.91828	1.20555	1.1712	12.6554	2.97579	344.54	112.208
AAEL012701	ATP-binding cassette sub-family A member 3, putative	5.98044	5.51348	5.82889	5.81949	10.2966	9.06804	82.0174	30.8528
AAEL005499	ATP-binding cassette transporter	0.465102	0.523325	0.201201	0.314362	1.45096	1.01619	53.8416	24.7823
AAEL011269	ATP-binding cassette transporter	13.6444	15.416	21.5486	44.7312	31.8866	31.787	7.83157	10.7727
AAEL012395	ATP-binding cassette transporter	3.55958	3.58935	2.33683	1.68068	12.5585	3.60136	19.6857	8.22085
AAEL005244	ATP-binding cassette transporter, putative	7.4182	6.80689	10.3662	33.3808	6.27636	4.70946	6.3633	6.69413
AAEL004297	ATP-citrate synthase	167.882	168.667	113.869	96.7367	171	158.43	84.129	116.095
AAEL005043	ATP-dependent bile acid permease	0.192543	0.277194	0.205342	0.342867	1.13901	0.581822	104.944	48.4464
AAEL005045	ATP-dependent bile acid permease	0.184927	0.136435	0.478197	0.260268	2.42021	0.775337	87.7263	37.7201
AAEL007512	ATP-dependent RNA and DNA helicase	17.6846	17.2415	22.0965	16.234	17.1514	16.9126	3.16372	9.70869
AAEL013235	ATP-dependent RNA helicase	41.5365	42.8003	37.8847	40.7757	44.1406	43.91	19.5688	31.1515
AAEL004117	ATP-dependent RNA helicase	32.3495	32.6111	24.9512	32.2472	41.2515	39.5626	14.915	23.8767
AAEL014155	ATP-dependent RNA helicase	68.7765	65.5951	60.5758	66.6787	50.4274	52.3854	119.444	63.171
AAEL006806	atrial natriuretic peptide receptor	1.93112	2.67013	9.66639	2.41544	5.45569	2.72319	53.7921	18.8546
AAEL003389	ATT	0.373466	0.429452	1.39704	0.432053	0.413053	8.32031	1.0427	0.252965
AAEL003388	axis inhibition protein, axin	22.1086	23.0885	34.9815	38.4015	29.3081	31.9331	6.72683	14.3367
AAEL001337	band 4.1-like protein 2, putative	5.04573	5.95172	5.77044	6.79364	7.47755	6.47339	39.9468	15.2381
AAEL008692	barrier-to-autointegration factor, putative	73.4789	80.7436	137.779	107.817	72.826	64.9084	110.712	95.7156
AAEL001779	bax inhibitor	135.973	142.643	126.199	129.597	93.5407	95.5987	467.493	283.303
AAEL006234	beta 1,3-galactosyltransferase	7.44048	6.97999	8.66369	7.5189	7.81076	8.2915	58.759	20.2769
AAEL005845	beta chain spectrin	40.5301	42.3756	49.2404	35.1524	33.3303	34.1389	135.32	75.2412
AAEL006242	beta chain spectrin	15.8735	15.324	17.8905	12.608	11.5551	0	149.897	54.4983
AAEL009700	beta lactamase domain	21.8512	24.0102	21.0204	17.0475	21.3855	27.423	633.006	336.081

AAEL002253	beta-1,3-n-acetylglucosaminyltransferase radical fringe (o-fucosylpeptide 3-beta-n-acetylglucosaminyltransferase)	12.9191	10.6847	9.77727	5.88098	5.94642	4.59494	0.854501	7.25032
AAEL005302	beta-1,4-galactosyltransferase	13.8361	14.7272	19.0375	16.4121	15.8167	14.5194	1.09777	5.615
AAEL000105	beta-alanine synthase, putative	261.973	329.626	97.1219	97.492	31.1538	32.7396	6.01656	138.252
AAEL004580	beta-galactosidase	0.297111	0.257475	0.371333	0.423308	0.864713	0.675261	453.395	194.93
AAEL004661	beta-hexosaminidase	27.7123	25.7177	19.7514	21.2557	18.8016	17.7237	212.072	89.3321
AAEL004931	beta-hexosaminidase b	5.44445	9.15896	6.47136	5.67857	14.4966	17.4075	2292.49	1406.7
AAEL013867	beta-mannosidase	8.9406	7.32431	6.35826	9.60854	5.12974	5.82958	361.532	182.911
AAEL011202	bhlhzip transcription factor bigmax	34.7672	30.5761	148.925	110.799	39.2067	47.3163	61.9355	42.2498
AAEL013305	bifunctional dihydrofolate reductase-thymidylate synthase	79.1006	74.0341	86.8403	97.7417	47.5461	49.3574	6.36713	44.2472
AAEL012825	bifunctional purine biosynthesis protein	75.8837	126.326	40.4321	32.9885	53.1751	50.0963	56.3016	80.408
AAEL013656	bm-40 precursor	67.4505	67.1838	20.678	25.1992	55.8225	60.9902	9.62591	31.6577
AAEL003861	bmp-induced factor	11.2095	10.8393	14.2106	12.1117	9.81487	9.19217	0.424575	5.26102
AAEL013239	bone morphogenetic protein	11.8493	10.9949	4.06823	4.58927	6.10848	5.41605	0.266503	4.5297
AAEL001684	boule protein, putative	69.1341	66.5032	62.605	74.541	48.8021	52.3559	3.65893	32.8324
AAEL003056	brain chitinase and chia	0.623023	1.346	0.872404	0.460008	5.57405	5.10671	525.782	246.731
AAEL002969	brain chitinase and chia	5.52337	3.19701	2.20353	2.24287	28.5871	11.3007	0.222038	0.766767
AAEL002964	brain chitinase and chia	20.4529	18.9933	8.25123	6.23211	32.979	21.347	1.32923	6.32907
AAEL003066	brain chitinase and chia	21.7802	23.6685	22.7367	21.613	25.9861	28.8473	10366.2	4646.26
AAEL006147	brain chitinase and chia	4.0359	4.54022	2.6001	2.95949	4.90397	8.14928	24.4139	6.43944
AAEL002959	brain chitinase and chia	1.51722	1.34526	0.839106	0.679532	24.0121	4.89937	0.084636	0.504275
AAEL004918	brat protein	40.5711	36.9875	0	99.5167	21.63	13.2637	13.6788	24.5372
AAEL014016	breast cancer metastasis-suppressor	30.6289	30.8546	52.1248	44.2828	22.9711	22.4394	10.556	20.6249
AAEL010509	bridging integrator	22.4777	22.7302	27.0128	26.8171	21.9221	21.9881	9.72064	16.4112
AAEL000001	bystin	13.818	16.9015	16.4641	12.4278	23.7329	24.8973	6.27421	12.1972
AAEL010924	ca-activated cl channel protein	7.89873	7.67128	0.140903	0.212879	0.785859	0.744329	0.053431	3.77685
AAEL009616	cadherin	0.42638	0.436127	0.405136	8.54255	0.452925	0.560903	0.219243	0.167739
AAEL001038	cak1	17.6769	17.2929	24.631	22.7032	16.3954	17.9146	4.65584	10.5277
AAEL007494	calcineurin b subunit	25.9088	22.5691	22.2431	26.7629	30.6432	33.4892	248.094	115.26
AAEL015549	calcineurin b subunit	4.97521	4.52587	4.92124	5.13453	8.29872	6.57482	50.2844	22.7875
AAEL002221	calcium and integrin-binding protein 1	34.2781	33.9696	47.4636	54.4654	21.9929	21.263	14.6324	24.0374
AAEL013823	calcium/calmodulin dependent protein kinase ii	15.0849	15.8656	18.8862	19.0899	13.0496	12.6157	1.58514	8.20368

AAEL010804	calcium/calmodulin-dependent protein kinase kinase	27.4137	22.6063	16.8646	11.5977	16.0303	16.2089	0.545626	13.4863
AAEL011441	calcium/calmodulin-dependent protein kinase type 1 (camki)	39.1492	43.067	60.8816	60.049	34.1353	34.9536	11.9968	28.3695
AAEL012513	calcium-binding protein E63-1	0.528735	0.790647	1.3325	0.791465	1.53244	1.36733	85.9349	37.8419
AAEL006582	calcium-transporting atpase sarcoplasmic/endoplasmic reticulum type (calcium pump)	130.78	125.408	85.4167	107.255	89.795	92.1833	77.6463	91.8027
AAEL011979	calmodulin	32.5621	34.4396	7.77059	10.2338	8.74453	11.5074	330.254	156.364
AAEL003952	calpain, putative	19.0481	21.5413	14.6963	13.3821	27.6798	25.6015	6.06196	12.8587
AAEL006922	calponin/transgelin	162.239	143.594	145.168	131.331	96.615	90.425	600.897	294.715
AAEL001005	calreticulin	182.558	255.431	72.5474	85.4835	74.1184	93.8728	304.876	220.829
AAEL011773	calreticulin	144.363	200.797	48.0587	58.7381	74.5716	74.7104	255.01	202.46
AAEL003499	calsyntenin-1 precursor	11.9948	10.0588	3.94725	4.75207	3.43358	3.66646	90.9194	44.2053
AAEL001976	camp-dependent protein kinase catalytic subunit	10.0937	10.9729	24.1555	24.4394	14.2288	15.2112	0.296178	6.63328
AAEL015314	camp-dependent protein kinase type ii regulatory subunit	7.71426	7.36553	11.369	11.9629	9.94807	9.6164	51.4475	23.2868
AAEL009839	cap binding protein	31.6034	31.0193	31.6766	32.4269	22.6821	23.2281	12.5238	21.201
AAEL009475	carbamoyl-phosphate synthase large chain	21.222	20.7499	13.7641	14.4959	40.9638	35.9979	2.39543	11.129
AAEL005337	carbonic anhydrase	13.9396	11.706	234.747	101.746	2.88414	3.36795	71.6894	51.4067
AAEL004930	carbonic anhydrase	40.5069	37.7679	88.39	81.2019	45.588	52.1467	906.664	469.116
AAEL008828	carbonic anhydrase	0.144838	0.369954	0.269969	0.168083	0.397851	0.865941	88.6245	34.4703
AAEL009330	carbonic anhydrase II, putative	0.377035	0.31161	1.83232	1.08493	9.92499	19.84	2447.7	1043.62
AAEL009323	carbonic anhydrase precursor	0.26576	0.056372	123.666	54.6369	7.33135	3.44946	210.014	98.5001
AAEL009691	carboxylase:pyruvate/acetyl-coa/propionyl-coa	108.985	124.537	104.147	129.354	85.3939	75.3328	46.9785	64.7835
AAEL000905	carboxylesterase	3.64729	3.63967	4.48275	5.3877	9.38699	6.00036	221.532	96.2559
AAEL000889	carboxylesterase	11.9265	11.107	37.8947	34.1042	9.3747	12.2672	0.152463	3.67061
AAEL000898	carboxylesterase	34.8565	32.5488	35.0915	34.4386	40.1246	40.0735	202.9	78.5425
AAEL010782	carboxypeptidase	0.762449	1.86186	0.559733	0.862079	2.1732	2.16995	7376.93	3905.02
AAEL010776	carboxypeptidase	0.530469	0.66486	0.27355	0.211491	1.97916	3.82641	5104.05	2567.99
AAEL005458	carnitine o-acyltransferase	33.3072	29.6771	41.8377	72.0162	32.2201	29.5205	75.2773	46.7381
AAEL002323	casein kinase	27.1249	22.8067	24.8619	24.6653	32.53	31.7994	1.64777	11.1235

AAEL012094	casein kinase ii, alpha chain (cmgc group iv)	123.024	130.062	102.196	106.504	138.805	133.315	97.6376	104.632
AAEL005955	CASPS17	1.92547	2.58704	2.08732	2.34496	1.55394	1.07421	50.4388	19.0633
AAEL003439	CASPS18	0	0.075384	0.093315	0	3.44321	0.262745	28.2583	9.5937
AAEL003444	CASPS19	0.334776	0.315756	0.098945	0.056934	2.81238	1.03847	73.7674	27.1631
AAEL014658	CASPS20	0	0	0.0761	0.055922	1.50087	1.53714	292.058	121.807
AAEL014348	CASPS8	6.58448	6.29951	1.78985	1.77348	7.97398	3.84427	119.074	46.9911
AAEL013407	CAT1	89.7224	99.0689	78.9036	80.8678	99.7913	99.4291	1285.66	660.103
AAEL007599	cathepsin b	6299.58	6320.17	4.14691	7.2796	1.97952	2.25606	110.606	3011.8
AAEL009637	cathepsin b	478.266	373.475	156.088	157.542	243.873	234.428	911.669	507.544
AAEL015312	cathepsin b	9965.59	9732.34	25.5514	56.0486	3.52054	3.76924	221.412	3462.41
AAEL009642	cathepsin b	1608.82	1594.45	0.697558	2.56142	4.14303	3.98139	29.3865	740.555
AAEL012216	cathepsin b	1676.05	1580.88	2.22104	3.24984	0.495003	0.624834	35.9527	2126.17
AAEL002833	cathepsin l	346.56	293.635	316.514	397.886	457.879	484.095	811.874	429.338
AAEL006389	cathepsin l	541.409	449.689	972.33	1027.19	150.19	160.752	10.0772	243.811
AAEL014902	cation efflux protein/ zinc transporter	26.33	24.2217	54.661	45.3179	26.2904	21.3733	66.9281	29.3607
AAEL001239	cation efflux protein/ zinc transporter	6.81391	1.11634	1.44553	2.05593	3.06004	1.41318	2.11284	1.65386
AAEL002557	cationic amino acid transporter	58.2252	53.7816	148.49	57.9324	42.5416	44.6453	3.85625	30.9341
AAEL008902	cation-transporting atpase fly	20.7417	21.0568	58.7474	40.3852	30.6708	26.2922	82.4669	38.3442
AAEL002853	ccaat/enhancer binding protein	3.60042	6.10731	0.152776	0.100631	8.91523	0.614003	17.4337	10.7167
AAEL011751	ccar1	31.4079	30.8914	30.3909	26.6676	15.4315	13.5065	13.0704	21.6744
AAEL004341	CCEAE1O	6.62341	5.52478	2.18829	1.65585	9.48009	8.3128	0.249142	3.29542
AAEL015590	CCEJHE1O	0.128975	0.075929	9.97956	45.3024	0.087416	0.031606	0.071774	0.108268
AAEL005198	CCEJHE2F	0.03065	0.036169	7.90972	51.4698	0.027338	0.030139	0	0.051624
AAEL005182	CCEJHE4F	0.130292	0.229677	83.763	142.767	0.17775	0.095869	0.216565	0.165225
AAEL009400	ccr4-associated factor	88.4831	86.6602	128.393	112.484	70.5461	66.2606	17.3348	54.8899
AAEL011500	Cdc42 protein, putative	6.45256	6.41621	13.0789	9.35415	9.71925	7.49357	218.404	105.902
AAEL012339	cdk1	9.15998	8.49367	17.2675	16.7084	1.05276	1.1817	0.479729	4.16771
AAEL008621	cdk1	41.3101	37.8529	71.1927	69.5687	24.7669	23.2465	5.65032	22.7276
AAEL000627	CECA	0	0	0	23.6197	2.95653	22.1999	0	0
AAEL004223	CECB	0	0	14.641	19.7656	75.6512	94.8143	9317.64	5138.19
AAEL000598	CECD	8.79978	0	23.0968	7.33895	66.9018	169.905	41.6176	5.40842
AAEL000611	CECE	57.6952	0	151.912	130.319	518.766	403.119	26.2734	0
AAEL000625	CECF	12.6296	0	2.2785	26.1567	70.5723	60.6711	278.3	28.5072
AAEL015515	CECG	3.13851	0	31.3471	56.638	229.9	177.97	0	0

AAEL017211	CECH	3.11774	0	2.23281	10.3432	8.52579	33.4826	1733.4	844.434
AAEL000621	CECN	69.3293	11.326	99.03	82.8992	348.352	410.676	129.503	32.8904
AAEL012821	ced-6	49.3992	43.2566	38.3075	38.6434	52.699	45.787	9.3718	32.5321
AAEL012967	ced-6	61.8938	64.6456	39.1296	40.4565	65.4358	62.1671	21.2779	33.661
AAEL008827	cell adhesion molecule	11.6495	14.4153	21.2139	19.1936	3.67312	4.13176	3.8088	9.8338
AAEL004477	cell division control protein	46.0497	45.2926	41.7333	40.9298	28.0428	29.0609	28.2548	35.0425
AAEL014025	cell division cycle 20 (cdc20) (fizzy)	50.6718	46.4116	75.6224	62.7838	19.2509	18.5112	1.73635	24.6385
AAEL007783	centromere protein-A, putative	46.2773	46.4026	97.9685	79.6274	33.9855	37.1011	0.882716	25.8658
AAEL003514	centromere/microtubule binding protein cbf5	63.866	71.5335	86.8544	73.869	95.5114	87.3662	19.57	40.3936
AAEL011678	centrosomal protein, putative	7.74432	7.74656	9.14495	8.83449	6.07079	5.9357	3.03167	8.09636
AAEL007030	ceramidase	27.6356	25.4098	28.1324	24.1081	17.2923	18.4383	594.048	284.078
AAEL013214	cgmp-dependent protein kinase	1.58842	1.38426	0.287867	0.168518	1.13814	0.907666	235.079	91.1559
AAEL005165	chaperone protein dnaj	402.398	403.943	586.504	517.486	552.209	535.814	480.3	348.762
AAEL009320	chaperonin	107.721	110.852	111.629	99.4054	170.403	175.747	62.3574	73.7749
AAEL011584	chaperonin-60kD, ch60	65.2325	73.9336	95.559	95.4532	75.9159	72.2643	29.3062	52.3517
AAEL013119	charged multivesicular body protein	14.3178	15.1502	15.8271	14.4111	16.2794	11.3371	66.5062	32.1844
AAEL001336	charged multivesicular body protein 2a	29.5776	28.2624	34.1154	30.9114	32.1012	29.8436	152.004	71.0606
AAEL001698	charged multivesicular body protein 4b	34.7304	32.8505	31.4937	27.4703	41.6206	39.8982	123.024	62.6449
AAEL013066	checkpoint kinase	55.3419	50.7686	74.7599	54.8443	60.9553	57.6607	14.7919	38.5835
AAEL005618	chitin synthase	0.144346	0.213291	0.118832	0.136838	2.42127	1.7215	360.397	136.719
AAEL007547	chloride channel protein	28.44	29.279	33.7051	35.0166	45.9483	47.5875	69.7276	34.3568
AAEL010245	chloride channel, putative	29.0936	30.7481	25.4324	21.7691	24.4426	25.5848	16.622	22.9194
AAEL007761	chloride intracellular channel	41.8189	41.2484	74.3063	77.3642	33.8911	32.427	174.554	97.2127
AAEL005977	chondroitin 4-sulfotransferase	0.222928	0.347876	16.8763	6.58521	0.889916	1.0308	33.6	12.9754
AAEL014365	chromaffin granule amine transporter, putative	95.1492	92.7571	119.567	105.612	83.6668	83.7683	1.5975	45.2453
AAEL013940	chromatin assembly factor i P60 subunit	26.0765	22.574	28.8538	30.6447	16.8244	17.7342	1.69906	14.6182
AAEL000173	chromatin assembly factor-I p150 subunit, putative	39.0642	39.3981	30.798	27.8132	53.6446	49.1008	10.6063	29.3878
AAEL013136	chromodomain helicase DNA binding protein	55.726	52.8047	70.7694	60.715	70.3791	69.0492	37.0803	45.1098
AAEL013135	chromodomain helicase DNA binding protein	40.9382	39.9812	50.356	41.669	48.4896	48.0939	25.5678	33.1041
AAEL009392	chromosome region maintenance protein 5/exportin	38.4036	36.1833	16.6285	24.7675	36.0235	37.0656	15.5617	24.6093

AAEL010568	chromosome-associated kinesin KIF4A (chromokinesin)	11.1011	12.0951	20.6044	18.3081	11.1938	10.464	1.10287	7.46177
AAEL009680	chymotrypsin, putative	0.81602	0.301886	1.38932	0.836902	1.66027	3.04074	1223.28	616.003
AAEL011470	cis,cis-muconate transport protein MucK, putative	124.694	112.938	81.6384	77.7662	51.7035	51.7468	2.04823	63.7946
AAEL010513	class b basic helix-loop-helix protein (bhlhb) (differentially expressed in chondrocytes) (mdec) (sharp)	12.142	12.0226	7.90876	6.99852	20.2918	18.8498	85.5348	41.6019
AAEL002601	CLIPA1	20.8877	20.2882	16.3438	19.9463	66.6592	62.9874	0.598246	4.96649
AAEL002590	CLIPA12	14.2392	14.7767	13.3948	14.893	22.8928	24.5718	1.54576	9.0413
AAEL002595	CLIPA14	109.018	118.573	89.034	76.8584	281.784	244.075	8.85063	38.0838
AAEL002301	CLIPA5	35.307	44.2294	22.9111	21.1211	118.824	103.352	12.6123	20.5642
AAEL002629	CLIPA6	68.8413	62.0262	78.5813	74.2772	97.0535	88.191	3.60168	28.1524
AAEL000074	CLIPB1	40.5224	44.7011	29.3709	29.1442	134.438	130.26	1.87514	20.3812
AAEL003243	CLIPB13A	64.9775	58.2237	81.9585	75.3654	127.822	84.2719	1.10833	28.8132
AAEL014349	CLIPB15	5.78101	5.44162	8.37076	8.44957	56.7061	36.9187	8.45812	3.99443
AAEL014140	CLIPB24	8.81507	11.2236	6.53375	4.98273	13.5557	10.8711	0.464783	4.80153
AAEL007993	CLIPB27	0.100158	0.083213	0.330551	0.13945	1.3016	2.27404	50.7676	10.8393
AAEL013245	CLIPB28	8.66673	8.3803	1.06834	0.9387	12.5532	11.1482	0.214443	3.27303
AAEL000099	CLIPB33	32.3274	37.8891	9.35553	10.6711	47.3277	46.4946	8.85333	19.6862
AAEL000028	CLIPB34	91.0975	104.975	72.3285	59.9826	244.979	235.689	8.32754	53.2031
AAEL000037	CLIPB35	10.6958	11.6691	16.5279	21.3634	69.91	71.076	0.364572	4.38974
AAEL005431	CLIPB37	8.3963	8.93238	3.9908	5.98534	19.5256	40.68	2.15919	3.75769
AAEL003632	CLIPB39	0.540412	0.236695	0.159126	0.210193	8.77276	2.05816	0	0.176654
AAEL006168	CLIPB42	14.2648	19.1207	3.79245	3.47222	12.7979	6.48173	0.757815	8.53239
AAEL006371	CLIPB47-76	0.170734	0.27381	0.265022	0.028232	0.369798	0.235831	55.6895	19.1842
AAEL005064	CLIPB5	26.9206	30.4815	51.3056	39.4817	53.7234	44.5243	1.87371	17.5188
AAEL003625	CLIPB8	23.1583	30.388	15.7682	11.5931	189.015	176.447	2.69887	10.2944
AAEL012713	CLIPC16	16.1263	20.3515	17.3854	13.3517	52.8421	55.5406	2.85629	7.66943
AAEL007593	CLIPC2	12.0473	11.828	15.1602	11.1321	14.2892	17.3198	0.801272	5.62634
AAEL007597	CLIPC3	32.1451	32.5579	27.4208	27.4853	53.1719	53.3022	1.74513	17.2105
AAEL004540	CLIPC6	16.7588	19.9208	20.2097	16.3197	39.555	34.5704	0.213504	8.40692
AAEL002124	CLIPD6	10.2698	12.3946	5.03705	4.35941	18.1117	13.8288	2.16265	9.16864
AAEL006576	clip-domain serine protease, putative	118.234	48.8815	82.7738	162.679	225.648	156.917	4.92113	19.3786
AAEL009722	clip-domain serine protease, putative	31.852	29.8239	38.3826	30.8352	68.8775	66.4749	80.397	38.4301

AAEL001098	clip-domain serine protease, putative	21.2483	26.2601	12.6787	18.0177	32.5023	38.3804	7.29759	18.1591
AAEL000030	clip-domain serine protease, putative	10.4351	13.5103	11.1045	7.91034	32.5071	27.5122	0.909712	7.2044
AAEL003279	clip-domain serine protease, putative	18.3982	14.4208	17.0161	12.7144	67.6623	32.9273	2.03807	11.3529
AAEL010773	CLIPE10	11.2541	11.7516	7.21337	9.80503	23.0114	16.4038	0.208505	3.80932
AAEL005644	CLIPE12	3.77805	2.85159	3.86003	3.30074	4.18158	4.5344	0.133905	3.16567
AAEL007949	clk2	10.5425	13.3352	6.99498	8.97504	9.17906	10.2471	7.15874	13.8612
AAEL017290	Coiled-coil domain-containing protein 72 homolog	292.587	288.041	387.732	307.959	340.434	371.331	221.887	247.523
AAEL005097	cold induced protein (BnC24A), putative	1048.31	1114.41	426.13	386.877	3166.16	3101.85	2118.04	1458.67
AAEL011090	complement component	96.8352	100.141	105.64	84.9751	104.412	96.729	29.2033	60.9137
AAEL004634	condensin	13.7287	13.8285	11.9505	13.5943	19.8893	20.6055	3.12629	8.58532
AAEL011049	condensin, XCAP-G'-subunit, putative	12.3764	12.6018	10.8935	13.4032	13.7758	14.3702	2.08152	7.9164
AAEL003160	conserved hypothetical protein	625.267	582.651	813.919	960.684	618.346	634.625	2015.03	1174.92
AAEL013127	conserved hypothetical protein	9.07598	19.7851	5.10721	7.96755	6.03369	7.51891	67901.9	39265.1
AAEL013126	conserved hypothetical protein	0	0	0	0	0	0	13.1325	0
AAEL006126	conserved hypothetical protein	0	6235.57	2.97402	4.12834	1.98609	2.33764	157.2	3505.79
AAEL003934	conserved hypothetical protein	8.10098	10.8311	8.03186	11.2177	11.9058	12.7914	0.24727	3.99485
AAEL003749	conserved hypothetical protein	36.2151	40.9495	64.307	47.1629	35.6316	37.7684	27.4804	25.2554
AAEL012518	conserved hypothetical protein	0.868134	0.583047	12.3295	36.0719	26.7975	21.3928	0.872873	0.743167
AAEL011810	conserved hypothetical protein	1.77257	2.55056	6.55472	3.68203	15.3251	5.19129	11.8691	6.96508
AAEL001577	conserved hypothetical protein	1.05625	0.135588	1.48437	0.545707	3.42778	1.42324	1107.92	468.795
AAEL004877	conserved hypothetical protein	24.299	23.683	34.2624	31.8422	31.6972	31.8263	4.35396	13.8559
AAEL007017	conserved hypothetical protein	63.5811	62.8863	140.101	118.012	43.8046	49.2072	27.5998	45.222
AAEL014377	conserved hypothetical protein	12.2699	12.7289	15.2823	14.6063	13.4871	12.2742	4.30034	9.76144
AAEL000776	conserved hypothetical protein	245.981	230.733	536.349	335.378	113.569	105.772	2.86539	135.005
AAEL001791	conserved hypothetical protein	28.2421	24.9921	32.8934	31.4396	17.011	16.9977	10.906	17.3743
AAEL003756	conserved hypothetical protein	15.0334	15.6385	20.0209	20.0288	13.5242	15.712	3.69558	10.0505
AAEL004151	conserved hypothetical protein	1927.58	2069.75	908.008	875.739	4893.6	4789	3261.76	2626.81
AAEL007356	conserved hypothetical protein	35.7603	34.7531	40.7764	34.914	39.6953	39.4075	11.0428	23.6552
AAEL013835	conserved hypothetical protein	0.165471	0.185236	0.111229	0.264086	0.150514	0.331725	1693.97	596.306
AAEL014645	conserved hypothetical protein	13.7166	13.9712	17.3184	14.7297	17.3724	18.7419	5.08728	10.923
AAEL010906	conserved hypothetical protein	36.335	38.0433	45.5504	44.4107	36.5039	38.9926	15.8345	27.3904
AAEL000311	conserved hypothetical protein	0.161458	0.069604	63.9117	937.945	2.43932	2.13054	0.305339	0.234931
AAEL001508	conserved hypothetical protein	6.16259	10.4126	7.32747	5.18172	14.2844	14.2006	0.284843	4.72286
AAEL007344	conserved hypothetical protein	0.552098	0.433085	2.27573	2.12009	14.4099	6.28653	985.166	218.498

AAEL004405 conserved hypothetical protein	6.34134	6.41239	8.73485	6.64009	7.93041	6.31504	1.47022	18.8178
AAEL011227 conserved hypothetical protein	28.7221	34.623	47.8287	45.1732	20.6193	25.7578	6.80212	27.6299
AAEL011321 conserved hypothetical protein	10.7639	10.6526	11.6018	11.0461	11.9464	12.4591	2.9387	7.65151
AAEL006862 conserved hypothetical protein	13.7297	12.6091	132.891	45.2256	17.054	16.6487	64.7113	36.4735
AAEL011286 conserved hypothetical protein	11.8499	12.0329	4.20643	8.01496	11.1946	10.4921	1.70124	6.2385
AAEL005453 conserved hypothetical protein	43.2637	44.7465	29.8478	39.4346	34.1696	34.037	7.40935	26.5337
AAEL003332 conserved hypothetical protein	18.0804	17.5764	23.9675	23.2422	10.313	10.8819	3.95348	12.3624
AAEL007173 conserved hypothetical protein	80.7534	81.9971	71.6563	110.077	45.5176	45.2726	166.081	108.694
AAEL004946 conserved hypothetical protein	35.3165	36.8448	46.1752	40.3776	37.2389	35.5179	20.4253	28.5825
AAEL000827 conserved hypothetical protein	0.294128	0	43.5819	326.443	0.132452	0.288223	0.170748	0.257216
AAEL004055 conserved hypothetical protein	21.5197	20.7356	24.936	24.0502	23.6646	24.0852	3.03178	12.7496
AAEL002597 conserved hypothetical protein	29.0313	31.1853	29.9259	31.8379	39.4282	33.758	186.413	87.4308
AAEL002711 conserved hypothetical protein	17.997	17.0441	48.5344	40.6506	4.35593	5.31089	1.13651	7.21751
AAEL003728 conserved hypothetical protein	1.62234	1.61437	1.53597	0.978556	30.5071	1.72771	148.057	30.7707
AAEL012181 conserved hypothetical protein	108.891	106.481	168.81	148.285	107.846	104.985	37.4664	65.2887
AAEL010789 conserved hypothetical protein	0.496996	0.22856	1.11242	0.462461	0.868558	1.06002	79.9358	27.7465
AAEL001997 conserved hypothetical protein	18.8108	19.3041	25.6222	22.153	41.8641	40.3963	6.07456	12.2897
AAEL005598 conserved hypothetical protein	38.8793	42.2727	44.1354	42.2634	46.4646	47.8369	26.9759	41.4471
AAEL009604 conserved hypothetical protein	136.998	146.716	246.184	207.561	108.136	109.39	377.419	222.45
AAEL011827 conserved hypothetical protein	27.2483	26.3785	25.0349	21.9933	31.4864	30.4533	5.50067	16.7331
AAEL008497 conserved hypothetical protein	18.4536	20.0065	19.6451	20.1573	14.071	13.2723	106.421	47.7267
AAEL007820 conserved hypothetical protein	69.9192	68.4292	76.66	79.2926	76.3987	74.1057	51.3626	55.4699
AAEL013735 conserved hypothetical protein	31.0474	33.094	34.1796	35.9328	25.0689	26.0494	18.0803	25.9814
AAEL009032 conserved hypothetical protein	18.655	12.3847	6.31717	20.2547	5.21982	5.55991	7.16347	7.7604
AAEL008054 conserved hypothetical protein	1.03323	1.49522	1.35929	1.55448	0.366335	0.581562	11.3188	2.31013
AAEL002443 conserved hypothetical protein	8.23721	7.45485	11.2635	13.3222	6.67571	6.96537	2.3428	7.81666
AAEL012460 conserved hypothetical protein	33.6589	35.2589	42.0484	40.4146	27.2835	27.9088	21.6509	28.2838
AAEL000397 conserved hypothetical protein	16.7382	15.2676	13.9103	15.4741	12.2178	12.4729	4.84256	10.7227
AAEL014434 conserved hypothetical protein	1.49664	0.400853	10.8603	4.94841	5.0664	2.12336	41.5812	15.6039
AAEL010996 conserved hypothetical protein	3.64928	3.61377	3.86386	4.21473	18.2446	10.5254	37.3182	15.1644
AAEL001509 conserved hypothetical protein	8.80063	8.34728	5.83138	5.87509	17.3704	10.8593	169.894	60.9261
AAEL011881 conserved hypothetical protein	50.3164	47.4872	44.1146	39.149	41.0341	39.3417	102.046	45.6587
AAEL004060 conserved hypothetical protein	318.818	370.526	297.835	240.296	501.683	517.82	660.028	554.499
AAEL007554 conserved hypothetical protein	23.2477	23.5775	30.6794	28.9037	28.4553	28.2622	6.53738	14.8368
AAEL011343 conserved hypothetical protein	0.19731	0.299159	0.256501	0.222713	2.61274	1.54373	435.331	171.171

AAEL011791	conserved hypothetical protein	13.1455	14.946	6.8602	6.56361	10.154	9.2656	0	9.82989
AAEL012462	conserved hypothetical protein	22.8616	20.6627	24.5525	21.5365	21.0545	21.2344	6.19262	13.7432
AAEL008428	conserved hypothetical protein	0.817982	1.67406	3.51757	2.50103	16.1993	32.7656	3302.9	1355.5
AAEL010877	conserved hypothetical protein	26.9828	19.9239	49.6921	47.932	14.7774	14.3061	2.04293	12.1935
AAEL013585	conserved hypothetical protein	1.75818	0.916616	76.3531	207.94	0.54196	1.07806	0.42497	2.75149
AAEL006617	conserved hypothetical protein	36.3124	38.0491	5.04568	2.19267	30.6605	30.644	17.6155	38.7642
AAEL014276	conserved hypothetical protein	14.0379	15.2565	20.5844	17.7981	8.71197	10.0482	0.469311	7.2161
AAEL002165	conserved hypothetical protein	0.502581	0.226191	2.94714	2.51064	2.59166	1.52678	32.3755	7.41499
AAEL002950	conserved hypothetical protein	18.8771	19.1175	48.5635	31.7719	12.9414	12.312	79.9801	46.4788
AAEL004699	conserved hypothetical protein	599.167	575.823	225.867	339.248	874.565	882.375	503.053	495.075
AAEL008139	conserved hypothetical protein	27.3287	24.9838	39.0645	33.2362	22.9999	25.6955	68.4538	33.6359
AAEL001385	conserved hypothetical protein	30.2518	30.1069	36.0524	29.5599	39.463	33.6926	103.661	46.5357
AAEL000436	conserved hypothetical protein	47.1935	55.7329	50.4738	36.2649	88.9758	82.4842	22.0173	37.6708
AAEL013887	conserved hypothetical protein	19.032	17.3504	27.315	24.8595	19.5289	18.1836	5.23989	11.3169
AAEL011420	conserved hypothetical protein	33.1117	33.6028	16.7022	20.7002	20.6892	20.8455	14.3618	21.5906
AAEL006681	conserved hypothetical protein	7.45457	11.2671	3.74817	5.0982	9.4467	8.10929	0.182867	3.20571
AAEL000115	conserved hypothetical protein	25.0241	23.605	26.4366	26.0026	41.38	39.3802	8.25136	15.8927
AAEL000165	conserved hypothetical protein	52.754	51.7868	112.994	104.077	187.175	159.884	3.34714	16.9327
AAEL000191	conserved hypothetical protein	20.6107	20.3395	28.544	28.8525	19.1581	20.3088	171.942	86.2069
AAEL000267	conserved hypothetical protein	9.98616	12.7151	10.8595	11.7342	10.4679	11.9673	0.883806	7.08154
AAEL000328	conserved hypothetical protein	35.636	29.6184	33.5141	33.6093	24.0057	27.7397	220.132	97.9836
AAEL000574	conserved hypothetical protein	51.9625	57.1138	46.7971	43.3783	33.9572	35.2271	29.6867	37.9058
AAEL000650	conserved hypothetical protein	49.3172	49.1538	17.9529	17.3608	71.7907	84.0302	3.1461	15.3007
AAEL000831	conserved hypothetical protein	0	0.070482	1.81847	18.1238	0	0.087974	0	0
AAEL000846	conserved hypothetical protein	0	0.093675	10.7137	73.7646	0.07151	0.077531	0.181371	0
AAEL000860	conserved hypothetical protein	13.5949	12.4117	18.8356	20.1644	10.9459	10.9371	0.817658	5.60506
AAEL000949	conserved hypothetical protein	20.2238	19.1603	35.6638	29.6219	17.4297	16.4694	5.3087	11.4834
AAEL000973	conserved hypothetical protein	19.7226	20.976	75.806	170.041	22.781	20.2464	0.844285	6.00465
AAEL001104	conserved hypothetical protein	2.57567	1.49093	1.03522	6.01168	4.51356	3.99085	100.435	39.2096
AAEL001271	conserved hypothetical protein	62.4424	70.9265	16.7043	19.3638	115.946	130.622	3.32105	20.6407
AAEL001323	conserved hypothetical protein	8.31441	7.02883	39.0404	141.916	40.1949	24.951	0.32453	4.71402
AAEL001355	conserved hypothetical protein	7.85982	8.40684	4.85423	17.9656	15.1552	13.7593	1.44122	2.71857
AAEL001399	conserved hypothetical protein	24.4005	21.3551	32.8558	37.0304	11.8217	11.8969	2.31811	13.9048
AAEL001425	conserved hypothetical protein	16.0879	16.9315	18.9764	18.7227	18.8845	22.7527	4.67829	11.1203
AAEL001598	conserved hypothetical protein	18.7672	18.2209	9.33811	15.9324	15.1633	13.9999	5.85056	11.8353

AAEL001650	conserved hypothetical protein	4.52319	0.141132	0.086738	0	0.331135	4.33631	36.6203	0.429969
AAEL001810	conserved hypothetical protein	59.8395	55.1257	64.0152	79.1486	79.6367	81.4322	35.3853	43.1809
AAEL001959	conserved hypothetical protein	46.7087	45.4751	52.1283	60.8984	31.6437	34.121	333.827	179.731
AAEL002203	conserved hypothetical protein	0.264391	0.140994	0.146033	0.412457	1.14745	1.09954	65.555	29.3615
AAEL002381	conserved hypothetical protein	177.039	192.308	355.878	293.799	116.962	114.758	58.1015	88.6637
AAEL002426	conserved hypothetical protein	14.7788	15.1936	14.3187	10.6899	11.6816	9.70073	381.952	208.113
AAEL002435	conserved hypothetical protein	68.7568	64.8774	72.5856	71.2068	62.6485	63.8273	50.7287	57.5474
AAEL002559	conserved hypothetical protein	14.5801	11.8238	9.03234	7.71297	20.1299	24.5371	0.463586	3.99359
AAEL002560	conserved hypothetical protein	8.92283	7.27344	4.32232	6.2144	9.2025	6.54326	1144.98	469.05
AAEL002793	conserved hypothetical protein	13.6585	13.3139	26.4212	76.176	12.2054	11.7443	9.09029	12.0434
AAEL002821	conserved hypothetical protein	21.6729	21.3443	24.2559	21.746	14.6191	15.2618	7.56133	16.4088
AAEL002917	conserved hypothetical protein	0.484226	0.405421	65.5105	8.27296	1.68888	1.12328	0.37501	0.11376
AAEL002963	conserved hypothetical protein	52.4995	56.6897	100.662	74.8579	42.9328	38.6966	309.279	131.582
AAEL002968	conserved hypothetical protein	49.2441	58.1633	44.6334	47.6588	53.5436	57.1486	0.677414	24.8608
AAEL003052	conserved hypothetical protein	7.10838	0.629384	1.72761	1.13675	0.551229	0.422266	0.239083	0.059915
AAEL003065	conserved hypothetical protein	25.0532	24.9632	40.9589	37.3306	20.1629	17.6212	8.30495	16.161
AAEL003087	conserved hypothetical protein	9.44996	8.81171	13.8222	13.662	8.9687	10.0102	3.36445	9.10841
AAEL003149	conserved hypothetical protein	11.5774	9.0665	13.0889	15.0388	12.874	11.869	2.16235	7.70263
AAEL003295	conserved hypothetical protein	7.11714	4.32947	2.40783	2.97486	0.871364	0.684045	1.56314	8.60003
AAEL003303	conserved hypothetical protein	182.041	190.578	247.903	171.169	219.265	209.609	28.8181	103.645
AAEL003406	conserved hypothetical protein	10.2991	9.53089	6.28541	12.1349	6.85669	6.59567	0.867755	4.99534
AAEL003428	conserved hypothetical protein	6.59209	6.90008	17.4443	14.3799	23.9039	18.5639	107.22	53.2868
AAEL003435	conserved hypothetical protein	37.7137	37.1771	73.4027	85.1046	15.7678	15.7225	15.6423	27.4363
AAEL003540	conserved hypothetical protein	28.6659	25.1973	19.4788	25.0116	34.105	32.5865	5.16574	17.4424
AAEL003559	conserved hypothetical protein	28.6534	29.3742	45.9503	38.3575	30.0212	31.654	5.9095	15.2434
AAEL003648	conserved hypothetical protein	13.4789	12.1365	11.4661	13.0683	6.31055	7.53072	1.24482	7.78867
AAEL003738	conserved hypothetical protein	1.88256	2.52363	0.91074	0.995344	22.6462	0.797577	34.3785	6.97844
AAEL003740	conserved hypothetical protein	1.86791	1.48049	1.45456	1.24901	29.3121	3.23359	72.6973	10.8174
AAEL003804	conserved hypothetical protein	18.9886	11.5116	12.7045	24.7893	3.22411	2.7513	0.337739	6.35696
AAEL003876	conserved hypothetical protein	103.515	63.5073	101.784	105.374	61.0923	57.2619	17.6242	45.5985
AAEL003986	conserved hypothetical protein	402.944	473.388	58.7084	72.7491	90.2836	99.159	740.896	579.276
AAEL004043	conserved hypothetical protein	1.12347	1.19896	0.737512	0.852692	1.06434	1.24661	86.6658	43.6778
AAEL004053	conserved hypothetical protein	46.2404	49.1689	75.6874	66.2222	49.7751	52.1184	13.4603	30.0617
AAEL004084	conserved hypothetical protein	18.1762	18.8418	31.7309	22.9059	10.9927	11.1309	1.59393	6.73619
AAEL004169	conserved hypothetical protein	304.69	302.466	384.065	380.778	259.767	270.517	1138.41	590.52

AAEL004212	conserved hypothetical protein	2.93184	3.06961	9.8044	15.4933	11.963	10.1244	36.4081	14.9283
AAEL004363	conserved hypothetical protein	51.5211	43.6346	40.4001	42.5255	43.0028	46.7945	23.1264	33.0563
AAEL004397	conserved hypothetical protein	14.5416	14.0931	26.62	23.1875	13.2697	13.4026	99.377	50.3195
AAEL004521	conserved hypothetical protein	86.3259	54.1996	37.9441	84.5334	61.0044	113.785	7.55186	62.0987
AAEL004547	conserved hypothetical protein	57.5469	58.3517	18.8363	21.4638	110.107	92.3772	1.09621	12.4858
AAEL004688	conserved hypothetical protein	24.4098	24.873	16.2465	18.9914	34.0866	33.6023	124.507	52.2658
AAEL004706	conserved hypothetical protein	29.4696	33.7163	52.7121	39.1114	23.6518	32.4836	14.9731	27.6972
AAEL004727	conserved hypothetical protein	13.9657	13.8893	29.4968	65.9931	13.7576	12.8963	45.8838	26.6699
AAEL004809	conserved hypothetical protein	65.2524	94.9892	28.216	46.9168	102.347	132.47	35.1761	23.9643
AAEL004845	conserved hypothetical protein	15.0608	13.5957	12.0327	12.5016	15.4412	9.61687	67.3454	32.6816
AAEL004862	conserved hypothetical protein	16.6753	17.3565	5.64112	23.6341	2.53886	2.42118	1.03729	13.5459
AAEL004976	conserved hypothetical protein	110.538	96.259	291.578	251.059	53.9552	67.096	2.70459	57.525
AAEL004987	conserved hypothetical protein	48.064	55.4388	16.04	16.5209	41.2036	39.7563	1.143	23.2577
AAEL005048	conserved hypothetical protein	7.40839	9.00624	12.9556	16.8355	14.3509	13.3854	56.0718	22.9269
AAEL005110	conserved hypothetical protein	8.56323	8.90652	11.5993	11.2194	6.3183	4.54585	0.133468	6.20362
AAEL005203	conserved hypothetical protein	10.1314	11.3074	8.73289	9.66917	24.2614	16.6677	47.1077	17.0159
AAEL005215	conserved hypothetical protein	73.9281	70.5824	58.9202	70.427	56.702	56.0236	23.691	43.3421
AAEL005224	conserved hypothetical protein	20.7564	19.234	25.8344	25.0849	12.8506	15.0866	5.02801	12.5114
AAEL005262	conserved hypothetical protein	37.0729	33.3593	71.76	133.247	28.8601	27.3014	123.098	72.2293
AAEL005309	conserved hypothetical protein	16.7285	15.6773	23.1584	18.283	14.6581	17.1457	2.83165	10.3951
AAEL005592	conserved hypothetical protein	7.79262	8.20033	13.048	12.4218	7.18462	6.17905	1.69718	6.69035
AAEL005704	conserved hypothetical protein	35.0296	35.3607	20.3548	21.5417	22.2623	22.6951	3.23975	16.6705
AAEL005716	conserved hypothetical protein	32.3651	34.9625	7.9231	15.5847	40.7325	44.4071	3.33473	16.5941
AAEL005736	conserved hypothetical protein	56.713	55.6233	57.1194	57.0479	44.4142	46.464	14.1083	34.7008
AAEL005739	conserved hypothetical protein	0.365321	0.233975	0.249943	0.230951	2.60774	1.57924	367.59	143.268
AAEL005756	conserved hypothetical protein	0.162818	0	380.218	551.984	0.051939	0	1.32154	0.90906
AAEL006041	conserved hypothetical protein	9.32839	13.0204	8.10203	7.91334	37.9146	19.4607	22.8369	10.631
AAEL006087	conserved hypothetical protein	51.4844	51.2153	41.4754	46.2757	53.1388	53.2976	16.8131	31.5782
AAEL006124	conserved hypothetical protein	29.1272	30.285	43.8232	37.1521	35.0793	36.2998	14.7776	22.2784
AAEL006208	conserved hypothetical protein	23.2925	21.1181	27.4979	25.221	14.1878	14.6995	3.30607	11.7674
AAEL006328	conserved hypothetical protein	0.505659	1.11785	27.4175	537.491	0.837128	1.11873	0.420484	0.413401
AAEL006364	conserved hypothetical protein	11.6294	11.6064	8.29645	17.5526	9.63909	8.50747	65.8375	27.891
AAEL006408	conserved hypothetical protein	0.105976	0	0.082159	0.09498	0	0.106055	16.2798	0.172494
AAEL006423	conserved hypothetical protein	0	0	0	0.356552	0.127865	0	15.2937	0
AAEL006480	conserved hypothetical protein	124.83	136.098	47.7601	74.011	102.259	117.659	275.197	123.74

AAEL006483	conserved hypothetical protein	0.849799	1.70358	4.3872	4.69276	17.0346	47.8386	2197.07	1158.75
AAEL006637	conserved hypothetical protein	22.0251	21.2764	24.282	19.8959	29.6408	26.7493	3.21755	13.8473
AAEL006660	conserved hypothetical protein	15.6232	13.2689	26.4524	24.7144	3.89049	4.7277	1.65534	7.96874
AAEL006663	conserved hypothetical protein	14.3932	15.2345	20.1529	11.7188	13.9812	15.2901	47.5756	18.0685
AAEL006670	conserved hypothetical protein	11715.9	9558.15	4.95376	17.5539	8.36086	5.47377	125.472	6293.62
AAEL006697	conserved hypothetical protein	12.7787	13.8685	14.9482	13.4141	20.8434	21.2694	4.82362	10.186
AAEL006884	conserved hypothetical protein	15.583	14.5724	21.4079	19.3898	14.3172	14.9331	0.103326	5.98848
AAEL006953	conserved hypothetical protein	0.210645	0.122251	0.114036	0.616394	1.89284	0.21284	307.769	378.177
AAEL007096	conserved hypothetical protein	0.041195	0.096872	0.306433	88.727	0.149029	0.162284	0.182565	0.135956
AAEL007114	conserved hypothetical protein	41.3288	33.555	31.5985	31.0121	66.7856	62.3432	185.575	86.9135
AAEL007167	conserved hypothetical protein	10.7358	11.202	26.4208	22.6746	9.59046	11.4141	2.84861	9.1744
AAEL007196	conserved hypothetical protein	38.8009	40.3502	66.5983	38.8592	48.0741	52.3357	12.7067	29.2616
AAEL007241	conserved hypothetical protein	24.2211	24.4825	25.0659	27.1874	29.6707	26.0907	15.5986	22.3686
AAEL007279	conserved hypothetical protein	17.274	17.595	32.8084	24.8728	17.1496	17.8794	3.87754	10.0515
AAEL007296	conserved hypothetical protein	37.6383	55.882	20.7905	21.4	19.3592	20.7679	28.9204	37.1515
AAEL007298	conserved hypothetical protein	44.9733	43.0184	18.1484	28.7202	23.362	22.3047	211.12	118.768
AAEL007329	conserved hypothetical protein	32.4864	34.1919	14.4327	18.5424	19.6795	20.4248	3.75148	20.0043
AAEL007349	conserved hypothetical protein	5.76924	5.66735	6.33442	9.36199	45.9046	32.3855	803.331	286.191
AAEL007403	conserved hypothetical protein	1.29369	0.963195	2.12597	1.8269	3.36304	2.20455	91.4684	42.0612
AAEL007447	conserved hypothetical protein	24.4921	24.087	47.4699	38.4602	10.3205	11.7168	5.56857	13.8771
AAEL007490	conserved hypothetical protein	225.257	279.172	229.35	196.402	307.696	336.787	986.781	681.067
AAEL007686	conserved hypothetical protein	102.811	95.4956	2.6799	2.199	15.9347	12.8791	0.485806	47.9299
AAEL007703	conserved hypothetical protein	2.40592	1.0436	2.85011	3.26623	1.96003	12.4757	12.6013	1.63321
AAEL007810	conserved hypothetical protein	18.527	17.8456	25.8774	25.543	11.5281	11.7673	0.94631	10.767
AAEL007997	conserved hypothetical protein	2.82241	2.00313	16.2961	55.6931	1.56284	2.46962	5.12649	2.90056
AAEL008274	conserved hypothetical protein	50.0507	57.6516	43.5781	36.8825	151.306	124.172	486.451	230.947
AAEL008275	conserved hypothetical protein	0.319274	0.224423	0.332033	0.164607	1.6804	1.44026	218.372	107.988
AAEL008320	conserved hypothetical protein	19.1327	18.4777	19.9224	20.5647	19.4362	18.8946	1.82546	10.2009
AAEL008578	conserved hypothetical protein	60.0993	58.5101	69.6821	69.5386	47.861	48.3196	9.43922	34.9716
AAEL008594	conserved hypothetical protein	60.1728	86.6042	30.2422	26.9036	54.3909	58.5696	64.2698	74.0084
AAEL008793	conserved hypothetical protein	32.7977	26.1843	26.8222	34.6397	19.4274	19.6313	107.603	51.7462
AAEL008833	conserved hypothetical protein	16.1212	16.6567	19.694	19.4093	15.2284	15.8411	5.36671	11.4116
AAEL008834	conserved hypothetical protein	23.7842	23.9793	30.3008	22.3714	24.3624	24.4085	7.06334	14.4256
AAEL008852	conserved hypothetical protein	36.6099	37.3808	34.6318	34.3265	69.8231	64.8488	15.4978	23.6827
AAEL008868	conserved hypothetical protein	75.7885	68.3343	113.58	71.4039	99.9236	89.8506	37.3701	42.3046

AAEL009023	conserved hypothetical protein	22.9039	21.8247	20.0195	22.9431	25.5248	23.3099	2.39841	12.5071
AAEL009201	conserved hypothetical protein	254.921	307.21	265.313	236.878	410.873	414.753	880.026	685.31
AAEL009248	conserved hypothetical protein	22.5658	17.9851	25.6514	25.2004	16.0579	16.0118	2.14678	13.0182
AAEL009278	conserved hypothetical protein	70.6105	81.4717	83.8941	73.3933	89.21	83.7968	92.5118	97.3796
AAEL009422	conserved hypothetical protein	77.777	72.064	35.339	53.9183	52.551	57.9714	57.7963	91.9393
AAEL009433	conserved hypothetical protein	0.071039	0.247429	182.618	641.905	0.317134	0.412889	0	0
AAEL009640	conserved hypothetical protein	3.55072	3.24765	3.97689	13.0656	2.50345	2.3261	0.122678	1.16302
AAEL009650	conserved hypothetical protein	22.718	27.0975	32.4351	29.297	32.592	34.5598	9.23443	21.092
AAEL009669	conserved hypothetical protein	16.0878	24.9729	3.37975	1.29927	46.6237	44.0054	0.502167	6.72512
AAEL009681	conserved hypothetical protein	13.1269	12.5157	15.0048	12.5262	14.859	9.633	105.394	40.7817
AAEL009936	conserved hypothetical protein	10.0434	9.95766	22.8114	9.20981	6.93734	6.73706	6.41155	7.94659
AAEL009964	conserved hypothetical protein	143.524	176.801	151.559	124.676	224.135	223.253	362.604	299.037
AAEL010047	conserved hypothetical protein	23.4955	20.0868	36.5705	40.1111	18.7714	16.8273	203.687	115.537
AAEL010095	conserved hypothetical protein	33.444	28.99	47.3837	110.39	31.0859	35.3961	163.086	95.9534
AAEL010247	conserved hypothetical protein	17.529	15.2134	19.3915	20.9009	25.3398	16.2082	51.0072	21.6833
AAEL010355	conserved hypothetical protein	12.5142	12.0641	14.2636	10.9182	12.3489	13.8326	2.46903	7.08339
AAEL010429	conserved hypothetical protein	1.77849	1.6048	1.16522	1.43648	1.3485	1.19078	4092.85	2608.06
AAEL010435	conserved hypothetical protein	0.283256	0.432704	0	0.239979	0.169472	0.184747	1909.93	961.813
AAEL010444	conserved hypothetical protein	9.44866	9.52156	9.78758	8.69854	12.5157	11.2973	0.352896	4.70949
AAEL010477	conserved hypothetical protein	0.231906	0.503578	0.462706	0.32481	0.584026	0.473289	128.051	66.2766
AAEL010558	conserved hypothetical protein	54.6455	49.496	118.72	68.0108	113.011	127.121	103.464	44.6497
AAEL010765	conserved hypothetical protein	226.373	207.76	208.982	203.723	201.01	209.883	811.671	466.762
AAEL010848	conserved hypothetical protein	0.067408	0	0.049193	67.6517	0.061347	0.066937	0	0.356662
AAEL010885	conserved hypothetical protein	14.3994	14.6028	17.1847	16.9476	19.9239	22.5083	2.46185	7.19433
AAEL010898	conserved hypothetical protein	2.11679	1.52882	7.3845	25.0793	3.52692	4.33085	1.32319	0.920605
AAEL010903	conserved hypothetical protein	26.1708	26.0646	34.9953	30.2447	27.8669	31.8939	8.54816	18.6929
AAEL010943	conserved hypothetical protein	4.94128	5.17593	2.90356	2.51191	8.17018	5.34307	0.159652	3.14956
AAEL011081	conserved hypothetical protein	26.3681	22.8657	39.1266	40.6457	21.5479	21.1162	71.9121	35.6132
AAEL011151	conserved hypothetical protein	52.3066	33.7698	56.1265	77.6074	73.6085	58.4052	94.3375	68.6305
AAEL011238	conserved hypothetical protein	0.238369	0.092236	150.631	463.402	0	0.157435	0.375283	0.279628
AAEL011334	conserved hypothetical protein	4.99707	0.107226	0.477075	0.477044	2.17652	1.92175	179.727	67.9401
AAEL011338	conserved hypothetical protein	18.2226	18.7307	3.56985	4.4672	4.37837	3.19243	2.84275	9.83237
AAEL011342	conserved hypothetical protein	4.18415	3.27231	4.45446	3.63037	3.65491	3.04011	23.4112	7.18247
AAEL011347	conserved hypothetical protein	4.42254	4.83524	5.97106	5.96097	10.4316	9.7316	51.6593	21.3938
AAEL011371	conserved hypothetical protein	14.1382	17.7442	30.7523	38.9749	34.5269	16.7768	39.0008	29.0739

AAEL011413	conserved hypothetical protein	18.1925	16.2134	19.31	19.284	16.162	13.5966	2.40348	11.2454
AAEL011551	conserved hypothetical protein	0.331246	0.209347	0.636262	0.383027	8.51765	4.49271	994.963	361.619
AAEL011715	conserved hypothetical protein	13.4632	14.3837	8.19638	9.02022	10.3292	11.893	0.130433	5.52817
AAEL011788	conserved hypothetical protein	7.9114	8.82714	12.5002	10.8536	8.30004	7.1074	0.276214	4.38993
AAEL011865	conserved hypothetical protein	30.5852	26.5076	38.6736	38.3529	46.3877	46.5444	113.974	59.4964
AAEL012023	conserved hypothetical protein	55.656	53.0129	38.852	40.4667	53.376	54.3435	28.4401	36.3602
AAEL012137	conserved hypothetical protein	17.2564	18.0262	25.9432	22.2094	17.686	17.9247	4.7992	12.2898
AAEL012235	conserved hypothetical protein	57.7685	59.9395	66.7408	55.4946	109.926	108.028	25.6257	37.9506
AAEL012262	conserved hypothetical protein	43.9944	43.6602	38.0506	34.8127	39.3828	38.6132	202.049	116.248
AAEL012382	conserved hypothetical protein	30.4754	34.158	49.2955	57.6974	26.5981	30.5776	1167.21	532.993
AAEL012571	conserved hypothetical protein	9.44608	12.2773	15.4572	11.643	80.1933	54.3232	1.62725	3.02664
AAEL012675	conserved hypothetical protein	15.3904	17.9471	17.1742	16.1438	25.8317	23.961	3.72662	8.66193
AAEL012677	conserved hypothetical protein	125.659	126.18	86.6314	116.641	102.695	107.815	11.093	61.2755
AAEL012764	conserved hypothetical protein	249.65	303.275	79.5219	52.8237	27.3716	31.7364	7.61775	105.291
AAEL012865	conserved hypothetical protein	170.908	170.976	188.669	180.654	141.046	142.56	50.5664	113.909
AAEL012876	conserved hypothetical protein	101.377	101.404	72.3498	79.967	25.414	25.983	3.14107	49.7313
AAEL012914	conserved hypothetical protein	47.6316	45.4723	51.3302	53.2184	32.2815	34.8647	39.4252	48.6784
AAEL013004	conserved hypothetical protein	5.84116	4.89711	9.06587	36.503	13.2867	14.5294	247.838	94.3645
AAEL013142	conserved hypothetical protein	23.4579	23.4569	21.8039	22.7034	27.2319	27.1467	2.04102	12.4103
AAEL013163	conserved hypothetical protein	7.91876	6.9134	5.2459	5.82862	26.617	11.8505	223.478	73.9902
AAEL013191	conserved hypothetical protein	7.90925	12.6495	8.52089	15.5226	12.7595	12.3908	5.02309	10.6017
AAEL013255	conserved hypothetical protein	30.4197	32.371	37.9331	40.1855	35.8115	36.655	92.8783	47.1699
AAEL013288	conserved hypothetical protein	73.4185	73.1111	98.64	94.8323	64.4209	62.3927	0.845475	37.2807
AAEL013291	conserved hypothetical protein	24.4315	21.98	32.1448	27.1882	12.1419	12.163	4.15496	13.2841
AAEL013295	conserved hypothetical protein	70.014	70.4657	54.4033	65.6007	60.5145	63.9398	2.03002	46.0885
AAEL013336	conserved hypothetical protein	5.15291	3.94914	5.60646	16.966	7.06825	8.61878	19.8077	14.584
AAEL013533	conserved hypothetical protein	1323.7	1357.74	656.875	590.468	4431.85	4602.88	2539.3	1905.23
AAEL013584	conserved hypothetical protein	2.88344	3.06426	1.01457	0.711505	1.50433	1.45928	12211.4	6781.29
AAEL013590	conserved hypothetical protein	19.1694	17.9013	24.4683	23.3825	22.9406	26.3249	5.09249	13.0089
AAEL013774	conserved hypothetical protein	1.53228	1.07915	1.0249	1.05644	18.8657	9.4383	4036.06	1851.69
AAEL013857	conserved hypothetical protein	413.346	599.109	1.32696	1.57818	21.8398	23.0199	10.1914	244.97
AAEL013885	conserved hypothetical protein	3.05263	3.15504	2.35151	1.69833	6.10528	20.7295	10584.9	3506.08
AAEL013889	conserved hypothetical protein	7.80042	8.21395	12.5266	14.2198	6.06173	4.54788	84.0148	36.7642
AAEL014283	conserved hypothetical protein	26.0143	24.2707	34.3777	35.3879	13.9314	12.2418	7.27542	13.2141
AAEL014484	conserved hypothetical protein	64.5035	55.9703	52.423	55.2726	45.7756	52.0452	285.651	169.056

AAEL014633	conserved hypothetical protein	16.1584	15.5248	21.4105	20.1144	16.8264	16.092	1.09953	9.01691
AAEL014874	conserved hypothetical protein	0	0	10.4069	37.9549	0	0.071301	0	0
AAEL014896	conserved hypothetical protein	0.359451	0.37252	0.258427	0.257016	0.766284	1.03884	103.19	38.8618
AAEL015256	conserved hypothetical protein	15.9926	24.9059	67.6264	73.8019	28.2423	16.9668	23.6537	83.7999
AAEL015543	conserved hypothetical protein	25.1897	28.993	39.3108	28.9279	27.5104	28.7214	5.56201	18.9489
AAEL015593	conserved hypothetical protein	1.29065	1.00554	1.71908	1.69288	1.80423	1.773	47.1698	17.55
AAEL003513	conserved hypothetical protein	0.152159	0.065121	83.7279	570.743	0	0	0	0.319099
AAEL010240	conserved hypothetical protein	46.3286	47.8987	20.6498	23.4891	69.5674	63.8842	0.348877	19.0058
AAEL010808	conserved hypothetical protein	11.6344	8.34314	16.5873	15.9433	4.74753	5.2392	0.510549	7.52435
AAEL013719	conserved hypothetical protein	0.064987	0	0.049115	31.919	0	0	0	0
AAEL009636	conserved hypothetical protein	181.08	180.442	34.7363	37.4002	31.0224	31.437	31.6679	94.045
AAEL002333	conserved hypothetical protein	0	0	0.049645	50.5497	0.061683	0	0	0.11415
AAEL014943	conserved hypothetical protein	190.985	216.308	251.603	183.081	206.376	199.259	141.531	154.366
AAEL004734	conserved hypothetical protein	25.0429	24.3104	40.4488	39.661	26.4183	26.879	5.97337	15.1417
AAEL005016	conserved hypothetical protein	16.1332	17.2986	15.8328	14.6213	11.2304	11.6798	5.98849	12.3117
AAEL007562	conserved hypothetical protein	31.7311	32.7077	104.764	85.3249	18.5026	19.7027	1.16066	15.921
AAEL003654	conserved hypothetical protein	19.9952	20.6944	12.4537	12.7845	23.7776	23.2415	2.61605	11.5972
AAEL012737	conserved hypothetical protein	42.4309	36.0172	51.4966	40.2486	57.2374	51.2228	14.5561	23.7325
AAEL013720	conserved hypothetical protein	0	0	0.057893	26.903	0	0	0	0
AAEL003355	conserved hypothetical protein	61.3177	56.4718	47.7507	60.3471	22.7357	23.7092	2.24633	39.5132
AAEL009263	conserved hypothetical protein	80.3659	56.1607	276.817	218.781	14.0622	17.4277	1.20887	27.7943
AAEL013577	conserved hypothetical protein	9.02348	7.61707	5.75443	4.83429	5.57541	6.06135	26663.2	15568.5
AAEL012646	conserved hypothetical protein	1.3189	0	0.326688	0.196929	0	0.514811	7.0388	0.193081
AAEL009280	conserved hypothetical protein	25.8602	25.1886	36.1954	35.2386	16.8685	16.5991	11.428	19.0794
AAEL007259	conserved hypothetical protein	28.4522	34.2061	24.2741	21.2973	21.035	22.8628	15.5361	22.3079
AAEL004049	conserved hypothetical protein	13.2068	11.5787	9.66414	9.77457	11.0064	10.2695	0.462825	5.13369
AAEL009639	conserved hypothetical protein	26.9526	29.0685	42.4113	35.3391	31.4757	28.9828	9.69202	16.5224
AAEL000408	conserved hypothetical protein	20.2675	18.3705	22.04	20.2271	26.2864	30.0328	4.17945	10.6297
AAEL004438	conserved hypothetical protein	73.8875	75.6538	108.097	77.5255	80.5452	86.5854	42.9603	47.241
AAEL001955	conserved hypothetical protein	38.1425	33.4939	30.5351	29.7306	26.2454	26.4971	19.0936	28.4654
AAEL012372	conserved hypothetical protein	12.1777	13.4793	15.1599	12.4654	12.6864	14.3288	2.3826	7.02878
AAEL001326	conserved hypothetical protein	1.2566	1.1389	2.96896	13.9601	3.21164	3.43626	3.40656	1.52042
AAEL001448	conserved hypothetical protein	56.9907	56.5354	41.1418	46.2977	40.0864	40.075	30.0263	44.0569
AAEL008449	conserved hypothetical protein	122.331	85.8079	0.516245	0.453639	0.727962	0.681366	1.45494	47.7048
AAEL008801	conserved hypothetical protein	2.32926	3.96397	3.54654	2.36665	11.9602	8.03758	1898.77	593.79

AAEL013905	conserved hypothetical protein	34.4525	32.2985	33.3077	39.1109	20.7683	22.0113	7.0453	20.6709
AAEL009778	conserved hypothetical protein	4.90106	10.1835	7.52852	9.73657	6.3773	9.69228	0.886019	12.7263
AAEL008485	conserved hypothetical protein	152.35	123.668	22.449	22.2731	23.0731	19.9967	4.76773	39.1745
AAEL007478	conserved hypothetical protein	1.191	1.76258	3.91526	2.91142	3.3154	2.49788	291.488	129.973
AAEL008942	conserved hypothetical protein	17.0356	18.1909	17.7372	16.7361	28.5288	24.861	4.0513	13.8698
AAEL007381	conserved hypothetical protein	16.7684	18.7699	25.4292	24.2751	56.2568	51.8916	1.306	9.52048
AAEL009397	conserved hypothetical protein	17.5888	19.0563	20.8609	17.9774	17.5958	18.7417	3.38979	11.6336
AAEL009611	conserved hypothetical protein	14.664	13.4122	18.0386	16.792	13.512	12.7953	4.77183	12.8361
AAEL008640	conserved hypothetical protein	0	0.091579	3.94808	34.2137	0.069608	0	0	0.130733
AAEL007236	conserved hypothetical protein	301.194	297.171	454.873	408.438	153.465	157.373	15.4571	158.664
AAEL002366	conserved hypothetical protein	17.1697	15.4402	23.738	23.1553	13.1145	12.6993	4.05228	9.27315
AAEL001029	conserved hypothetical protein	23.6088	26.4311	20.8537	20.6989	26.2452	28.426	5.32356	14.6246
AAEL001897	conserved hypothetical protein	9.36008	10.1548	6.37216	8.28383	11.3845	9.36488	0.644223	6.61558
AAEL011588	conserved hypothetical protein	0.428238	0.071575	0.315364	0.129643	2.04	2.32588	974.806	439.774
AAEL006886	conserved hypothetical protein	0.482292	0.589609	0.178846	0.388473	20.6602	1.20825	18.0685	6.73013
AAEL008059	conserved hypothetical protein	3.55859	2.02111	0	0	0	5.54345	8.43125	6.73758
AAEL006007	conserved hypothetical protein	3.80429	10.6956	12.7895	9.8648	75.4045	115.658	31852.1	14523.6
AAEL003483	conserved hypothetical protein	22.8438	43.1175	49.6241	43.1029	191.393	162.686	6.19435	14.2934
AAEL004455	conserved hypothetical protein	21.9911	20.0021	25.3118	20.2005	29.6926	28.8216	4.40589	12.9922
AAEL005669	conserved hypothetical protein	0.262389	0.297575	0.248725	0.239432	1.0269	0.761057	253.609	117.102
AAEL012012	conserved hypothetical protein	8.27242	7.89152	5.66163	8.81283	11.5726	10.8425	112.331	55.2469
AAEL012502	conserved hypothetical protein	22.7065	21.0683	24.1601	20.0192	25.6019	27.1843	474.6	253.892
AAEL006350	conserved hypothetical protein	13.4937	12.4302	17.8824	15.8926	14.3986	15.0947	0.113382	6.87393
AAEL001153	conserved hypothetical protein	0.054569	0	0.787717	54.0865	0.196667	0.053725	0.250481	0
AAEL010786	conserved hypothetical protein	11.3258	11.7682	6.42209	5.92579	21.7339	19.6582	0.14153	6.31518
AAEL005528	conserved hypothetical protein	103.47	105.751	94.3933	94.7433	130.329	131.572	50.9958	69.3433
AAEL004302	conserved hypothetical protein	16.5245	14.8636	25.3146	24.1982	9.23233	9.22064	0.462721	7.97704
AAEL010799	conserved hypothetical protein	0.39883	0	0.680349	1.13884	3.04507	2.87869	422.52	199.593
AAEL008153	conserved hypothetical protein	14.0137	14.7134	6.56097	5.98784	38.9673	39.7711	66.6404	30.291
AAEL007757	conserved hypothetical protein	7.12218	6.59396	13.5593	11.2298	3.05844	3.65807	0.779867	4.36409
AAEL012644	conserved hypothetical protein	4.80774	0.078204	3.45227	2.7626	0.67491	22.2353	9.67973	0.117824
AAEL009428	conserved hypothetical protein	26.4544	30.046	45.4848	43.1397	30.7444	32.9297	8.91667	16.7773
AAEL011190	conserved hypothetical protein	5.75578	5.53646	7.07346	9.6399	4.38983	5.77808	1.76898	8.25904
AAEL005216	conserved hypothetical protein	56.9009	64.1859	90.7109	82.8826	56.5399	61.0512	10.4719	24.9339
AAEL006892	conserved hypothetical protein	16.8372	15.733	30.8104	9.81024	17.7679	17.7012	0.379666	10.0906

AAEL002457	conserved hypothetical protein	1.61827	1.02408	2.10352	2.0851	3.23931	6.44672	299.747	141.825
AAEL002921	conserved hypothetical protein	11.4082	10.3678	12.7899	14.0005	13.8493	16.1304	1.69657	6.97438
AAEL010775	conserved hypothetical protein	1.21618	0.74481	0.561461	0.323415	0.203291	0.143714	343.634	130.033
AAEL009862	conserved hypothetical protein	0.394821	0.27806	0.329365	0.104842	0.899068	0.860654	355.636	183.615
AAEL005102	conserved hypothetical protein	27.5074	25.98	9.14937	11.623	14.1097	16.7415	200.696	105.749
AAEL009194	conserved hypothetical protein	0.456506	0.651035	25.7393	100.372	1.6684	0.973642	0	0.305368
AAEL002983	conserved hypothetical protein	40.5123	39.5164	30.2567	35.95	51.795	49.1936	22.4178	30.463
AAEL009879	conserved hypothetical protein	47.4015	49.4238	34.227	40.166	34.3365	34.2053	17.6816	35.3196
AAEL001875	conserved hypothetical protein	21.1797	21.1353	24.4166	24.8501	14.6926	16.3905	2.78303	10.9761
AAEL011832	conserved hypothetical protein	113.662	113.095	138.834	124.774	121.261	124.492	47.3717	91.7742
AAEL007444	conserved hypothetical protein	70.035	68.6163	60.6676	95.1544	233.67	221.513	192.397	114.362
AAEL008723	conserved hypothetical protein	70.7859	74.6159	74.1017	64.239	70.935	71.7999	832.173	397.654
AAEL001358	conserved hypothetical protein	18.9132	17.341	32.3191	29.8367	9.54242	12.8843	3.133	9.9316
AAEL000948	conserved hypothetical protein	0.334842	0.465987	0.879943	0.522938	10.7809	5.07702	759.469	273.428
AAEL013025	conserved hypothetical protein	25.6214	27.1558	19.9134	19.5458	40.6941	39.9546	0.365767	15.1103
AAEL001487	conserved hypothetical protein	0	0	0.051481	66.0139	0.062821	0	0	0
AAEL012176	conserved hypothetical protein	14.2049	15.6838	20.614	16.9801	15.8421	17.2714	2.76081	8.97322
AAEL005428	conserved hypothetical protein	0.565995	0.190829	0.427958	0.563574	15.1219	3.72253	603.75	234.767
AAEL009399	conserved hypothetical protein	17.7086	19.0827	21.9265	20.5512	15.4884	17.9773	3.85878	13.4425
AAEL008039	conserved hypothetical protein	7.65522	4.84817	7.40293	7.76839	36.5927	24.1557	1230.18	452.974
AAEL012725	conserved hypothetical protein	19.22	18.0172	15.2402	22.0749	15.2393	15.4677	1.52967	11.0577
AAEL011088	conserved hypothetical protein	4.77229	4.95013	39.9254	24.1897	6.60435	6.97027	68.7539	33.4104
AAEL007920	conserved hypothetical protein	27.3519	28.7734	42.9039	38.7846	29.5911	25.0599	209.886	96.077
AAEL007591	conserved hypothetical protein	0.153753	0.68875	0.20697	0	1.11651	0.76989	2707.99	983.005
AAEL011701	conserved hypothetical protein	16.3215	15.4516	22.8086	20.241	13.6386	14.8643	5.17855	11.3209
AAEL000200	conserved hypothetical protein	127.65	142.556	56.0788	55.5962	460.098	492.844	9.35507	53.1509
AAEL005401	conserved hypothetical protein	40.4921	38.1039	36.5504	51.7699	72.8944	77.9157	5292.83	2610.36
AAEL001156	conserved hypothetical protein	31.0544	30.2208	28.4204	24.2542	23.2124	26.4295	14.7396	21.4601
AAEL007059	conserved hypothetical protein	18.5848	24.478	4.84971	4.75944	7.38239	11.031	198.449	108.198
AAEL008953	conserved hypothetical protein	13.7723	14.1903	34.4389	24.0505	71.3743	22.4094	157.075	57.3169
AAEL012964	conserved hypothetical protein	226.935	226.455	165.152	207.243	282.609	287.884	174.733	196.306
AAEL002891	conserved hypothetical protein	64.8531	64.5632	84.0188	76.9576	31.1078	32.2312	9.04188	36.4445
AAEL007399	conserved hypothetical protein	17.7806	18.2076	22.0932	22.1783	21.4466	23.2322	1.42097	10.5085
AAEL005593	conserved hypothetical protein	19.4296	16.6969	70.8981	38.2011	171.759	164.216	13.071	11.3518
AAEL009492	conserved hypothetical protein	5.0925	5.73723	6.46227	7.72284	10.1858	8.67959	199.227	87.6258

AAEL005091	conserved hypothetical protein	2.10969	2.16514	1.23996	1.61925	1.36248	1.08016	45.9008	20.2108
AAEL010931	conserved hypothetical protein	55.8177	59.0426	114.3	32.4891	46.7762	48.3376	141.47	95.3415
AAEL005452	conserved hypothetical protein	9.26014	9.56152	20.9851	18.9381	11.4184	11.6285	1.11754	6.19173
AAEL007156	conserved hypothetical protein	21.1313	19.4004	8.04259	8.27014	14.8328	13.9159	3.81832	10.9704
AAEL002843	conserved hypothetical protein	64.6457	64.0209	90.026	85.5073	28.8795	29.8794	2.90499	34.8781
AAEL014147	conserved hypothetical protein	12.5626	10.6716	12.0085	12.4955	12.9305	13.4893	2.94693	8.08421
AAEL012489	conserved hypothetical protein	19.7588	18.9476	19.0326	20.0193	20.586	21.9315	3.61268	10.4639
AAEL005432	conserved hypothetical protein	0.425988	0.212212	0.550283	0.337672	10.9996	6.62976	1065.38	333.957
AAEL008724	conserved hypothetical protein	18.942	18.4963	29.772	26.9007	9.31737	8.21714	1.81025	10.3621
AAEL004509	conserved hypothetical protein	0.646783	0.594846	4.86065	2.22683	2.78183	2.65217	59.4168	22.7903
AAEL006990	conserved hypothetical protein	3.18157	2.63715	1.94349	1.5026	3.19867	3.89693	266.314	128.565
AAEL000226	conserved hypothetical protein	37.1466	34.0909	41.4514	38.2928	38.6553	39.3459	19.6622	28.7776
AAEL013503	conserved hypothetical protein	8.87533	8.41582	6.44091	6.44386	5.64672	6.19	2.1813	7.91456
AAEL000294	conserved hypothetical protein	2.64689	3.25753	4.07714	3.62843	27.795	21.7676	157.276	65.694
AAEL000350	conserved hypothetical protein	0.435361	0.130865	398.027	819.894	0.174473	0.456033	0.062301	0.212337
AAEL000362	conserved hypothetical protein	1.2339	1.40523	1.35073	0.969024	2.75502	3.03788	63.8283	23.1072
AAEL000461	conserved hypothetical protein	26.3957	32.1497	38.9117	46.7611	32.2868	32.1913	113.445	59.2642
AAEL000570	conserved hypothetical protein	72.4284	64.3917	50.5121	49.5641	73.2822	50.3641	59.8951	92.5567
AAEL000658	conserved hypothetical protein	4.17245	3.84352	2.26862	2.49721	3.84445	3.98816	76.2236	30.8853
AAEL000699	conserved hypothetical protein	7.85444	7.66487	10.3667	8.77339	10.8383	12.3148	1.70587	6.06046
AAEL000796	conserved hypothetical protein	0.556902	0.527143	283.519	1758.35	0.56685	0.662389	0.879771	0.938442
AAEL000868	conserved hypothetical protein	12.4701	11.5351	21.7307	20.312	7.92116	9.72276	1.30526	6.65509
AAEL000996	conserved hypothetical protein	18.3976	20.6741	17.4619	15.7157	28.8362	29.9243	7.51248	14.4604
AAEL001004	conserved hypothetical protein	75.1572	72.5028	128.083	120.053	56.6553	53.3767	28.0812	53.15
AAEL001264	conserved hypothetical protein	76.4135	78.1826	112.772	92.1457	69.0769	76.0462	173.904	97.5348
AAEL001390	conserved hypothetical protein	28.5806	38.4448	19.4499	17.0167	26.6265	34.9628	0.28488	10.7685
AAEL001476	conserved hypothetical protein	16.3148	18.9103	9.97555	10.0514	22.8602	22.7	13.6281	23.5927
AAEL001520	conserved hypothetical protein	43.4515	41.4812	69.6254	62.5124	38.2571	38.3243	11.9255	22.5494
AAEL001559	conserved hypothetical protein	29.7573	29.6102	8.40752	15.0127	10.1329	11.3557	0.156275	16.3738
AAEL001639	conserved hypothetical protein	0.553178	0.544446	3.48581	20.6479	0.988926	0.918725	0.575091	0.48324
AAEL001666	conserved hypothetical protein	159.673	153.995	374.152	396.651	49.6489	47.7713	1.83107	80.514
AAEL001793	conserved hypothetical protein	105.144	136.863	25.9273	21.0288	27.2813	24.3792	4.15775	89.9751
AAEL001913	conserved hypothetical protein	152.552	140.456	230.934	308.287	82.4552	81.0557	469.356	281.901
AAEL002142	conserved hypothetical protein	64.0921	60.8054	94.3612	60.2797	37.3692	39.6361	6.35076	23.8315
AAEL002163	conserved hypothetical protein	30.0808	29.8214	49.1329	65.9561	32.2009	28.3485	209.892	86.3093

AAEL002186	conserved hypothetical protein	31.8179	30.0254	33.1988	32.9342	30.0006	30.5629	9.13586	17.7131
AAEL002232	conserved hypothetical protein	19.1301	22.1164	24.5671	20.9595	25.7622	28.6122	2.53383	10.5127
AAEL002267	conserved hypothetical protein	0.079664	0.141434	0.433274	0.268118	2.97091	3.71759	761.233	309.059
AAEL002411	conserved hypothetical protein	79.7268	74.1276	115.123	104.291	72.8896	73.9304	561.072	327.639
AAEL002432	conserved hypothetical protein	0.02348	0.055201	0.035485	0	0.042363	0.022942	223.893	115.348
AAEL002495	conserved hypothetical protein	1.35477	0.992537	4.93101	3.17131	129.669	116.363	8075.6	3393.82
AAEL002529	conserved hypothetical protein	12.3375	14.8964	24.0217	20.9321	14.3665	13.5785	7.11659	12.972
AAEL002791	conserved hypothetical protein	27.0729	23.3542	28.9678	21.1648	26.0613	27.4185	76.5147	38.1951
AAEL002815	conserved hypothetical protein	300.444	344.157	7.39428	7.57111	22.2371	21.1601	18.9053	132.247
AAEL002890	conserved hypothetical protein	2.15799	9.87629	1.03604	1.09292	4.78175	3.7069	1.43957	4.32925
AAEL002900	conserved hypothetical protein	40.0913	38.7586	37.1141	38.6889	59.7653	57.4063	222.146	98.2008
AAEL002905	conserved hypothetical protein	34.82	33.7892	42.7275	46.7014	24.029	25.3056	14.9926	27.5153
AAEL003086	conserved hypothetical protein	65.7263	63.2734	131.734	123.548	50.6161	54.5026	528.324	331.84
AAEL003114	conserved hypothetical protein	13.2254	7.45286	8.9703	12.8351	21.1689	17.9571	171.066	54.0776
AAEL003525	conserved hypothetical protein	10.2354	9.35963	58.6764	406.147	11.3814	11.4501	7.28045	6.47382
AAEL003600	conserved hypothetical protein	0.071292	0	0.051055	0.088173	0	0.142602	5.74653	0.311549
AAEL003949	conserved hypothetical protein	16.3051	18.1319	17.7321	13.8792	25.2081	25.9337	3.4145	10.0115
AAEL003957	conserved hypothetical protein	254.985	262.313	224.014	208.564	192.473	187.649	1075.92	565.79
AAEL003992	conserved hypothetical protein	18.9892	20.606	23.7043	20.6934	18.0408	18.3332	7.75479	15.7223
AAEL004277	conserved hypothetical protein	0.179732	0.1569	0.132774	0.268872	0.522958	0.17468	47.9981	16.9049
AAEL004422	conserved hypothetical protein	16.1963	16.9899	12.7303	14.5611	18.5432	21.1396	3.15285	9.01512
AAEL004439	conserved hypothetical protein	25.457	24.3685	45.86	37.0587	24.8926	24.8268	2.8303	15.0902
AAEL004490	conserved hypothetical protein	44.3299	44.6187	60.2102	60.0064	35.1406	35.5926	22.9563	32.0641
AAEL004516	conserved hypothetical protein	0	0	0.305211	14.6533	0	0	0.192581	0
AAEL004553	conserved hypothetical protein	5.16406	5.81657	7.52696	3.68473	9.43526	10.4236	162.646	67.157
AAEL004657	conserved hypothetical protein	62.1272	58.9318	41.9424	50.4189	39.2763	39.9472	231.496	116.341
AAEL004665	conserved hypothetical protein	0	0.107244	0.068617	0.158149	0.08363	0	90.9727	38.7692
AAEL004817	conserved hypothetical protein	7.68728	5.93397	1.75787	3.93138	6.35759	6.5705	0.325996	3.5754
AAEL004873	conserved hypothetical protein	66.1991	56.9677	530.96	832.326	35.4598	41.178	26.8707	38.3751
AAEL004914	conserved hypothetical protein	76.559	90.0263	54.6668	50.8878	46.6431	47.1311	52.2585	64.5823
AAEL005171	conserved hypothetical protein	33.9218	33.4382	9.49748	14.5836	20.8994	22.5281	16.6204	23.2347
AAEL005288	conserved hypothetical protein	24.6347	21.1431	21.321	21.6631	9.53294	10.6762	0.192629	12.8135
AAEL005395	conserved hypothetical protein	31.0124	29.3997	50.3031	49.4961	20.7573	23.3653	5.10408	19.0459
AAEL005457	conserved hypothetical protein	103.949	145.718	39.3913	37.8235	47.5688	51.3852	103.314	113.946
AAEL005465	conserved hypothetical protein	19.344	16.3221	20.5371	20.5026	19.3288	18.0764	3.93921	10.5348

AAEL005606	conserved hypothetical protein	11.2452	16.74	100.289	41.2622	17.1994	9.16256	3.57718	8.43654
AAEL005777	conserved hypothetical protein	4.38652	5.43521	11.4766	8.54832	6.22236	6.13397	0.288265	4.43721
AAEL005813	conserved hypothetical protein	33.9639	32.4386	31.1436	38.6375	31.8128	32.2932	13.5284	23.8193
AAEL005884	conserved hypothetical protein	144.921	138.395	217.78	211.921	83.3673	89.7527	94.702	131.784
AAEL006098	conserved hypothetical protein	14.733	13.7783	9.35152	10.1628	19.118	22.3088	71.6546	34.8983
AAEL006336	conserved hypothetical protein	0.153232	0	2.00758	31.3761	0.161006	0.044068	0	0
AAEL006570	conserved hypothetical protein	24.2214	24.1854	19.6764	21.0607	23.554	21.2415	2.54395	10.9389
AAEL006701	conserved hypothetical protein	0.168249	0.68744	2.70523	2.02211	6.1165	1.94302	148.283	61.2624
AAEL007043	conserved hypothetical protein	56.6502	58.6185	82.2798	59.2494	56.2311	58.376	62.1254	51.8637
AAEL007058	conserved hypothetical protein	18.1336	15.4803	80.763	60.3368	15.5289	15.8368	1.55437	9.75479
AAEL007084	conserved hypothetical protein	29.3157	29.9048	34.9857	29.3515	55.1582	52.3653	14.634	23.0225
AAEL007339	conserved hypothetical protein	0.362303	0.249696	632.307	152.792	0.128624	0.41746	0.164385	0.733618
AAEL007370	conserved hypothetical protein	54.9506	53.0006	58.4939	48.8654	43.6744	50.0578	29.2269	46.1984
AAEL007592	conserved hypothetical protein	0	0.226894	0.20365	0	0.366142	1.01078	285.105	91.589
AAEL007697	conserved hypothetical protein	17.0996	17.7909	19.6189	18.4653	19.6911	19.5571	4.83875	10.7134
AAEL007701	conserved hypothetical protein	69.379	58.9149	75.2919	68.3976	45.7904	44.5808	14.916	36.6362
AAEL007773	conserved hypothetical protein	15.5167	13.6518	7.71312	4.89161	18.6971	20.3063	47.8159	15.5265
AAEL007780	conserved hypothetical protein	1.04202	0.13412	1.08845	1.17482	1.19199	5.66659	5.33993	0.383735
AAEL008073	conserved hypothetical protein	53.3905	50.6606	60.5202	37.6706	92.0389	99.3701	112.114	69.4075
AAEL008100	conserved hypothetical protein	38.2019	34.205	57.0939	51.991	28.0796	27.5131	18.8727	25.5638
AAEL008157	conserved hypothetical protein	1.39572	1.11521	2.08364	2.13945	7.61894	3.35319	63.4616	21.7383
AAEL008182	conserved hypothetical protein	73.2641	62.6771	387.832	322.461	36.7199	41.9601	1.71259	34.728
AAEL008669	conserved hypothetical protein	0	0	1.15981	32.7662	0	0	0	0
AAEL008829	conserved hypothetical protein	64.6692	65.3942	8.81938	22.3709	53.1489	52.0606	0.469994	24.5797
AAEL008843	conserved hypothetical protein	124.561	121.587	59.4073	77.7547	95.0182	107.119	2.02386	52.0949
AAEL008899	conserved hypothetical protein	28.9663	27.0264	32.0294	31.3954	30.6035	36.178	5.53018	11.0594
AAEL009198	conserved hypothetical protein	20.5807	24.5764	19.3727	23.6791	25.5123	25.9219	294.468	140.498
AAEL009235	conserved hypothetical protein	64.8729	63.8553	85.3797	76.1101	56.2571	58.8976	26.7423	40.5792
AAEL009374	conserved hypothetical protein	10.1815	9.66889	10.0152	10.2761	7.8525	8.61652	4.74877	9.96264
AAEL009401	conserved hypothetical protein	9.95814	10.221	3.58703	6.0159	14.2924	15.8812	0.864809	5.66559
AAEL009483	conserved hypothetical protein	32.026	26.4617	35.4061	35.0148	40.2097	38.6847	186.064	82.2819
AAEL009861	conserved hypothetical protein	43.4976	49.0728	29.6554	20.3679	305.819	256.88	30.6078	34.2328
AAEL009899	conserved hypothetical protein	45.5101	48.4785	26.0268	36.5948	216.542	193.569	1.79653	17.5947
AAEL009912	conserved hypothetical protein	15.8283	15.0077	18.8719	16.4365	32.7094	33.0501	4.12148	9.96071
AAEL009955	conserved hypothetical protein	1074.05	1230.33	39.2121	13.4469	237.534	215.061	20.3196	515.964

AAEL010062	conserved hypothetical protein	1.58704	0.288419	25.039	13.1181	3.52702	2.97653	1.09748	0.584495
AAEL010169	conserved hypothetical protein	2372.03	2491.55	1098.79	1025.49	6537.23	6764.12	2798.57	2423.58
AAEL010249	conserved hypothetical protein	122.833	136.239	113.292	115.676	109.218	108.832	14.418	76.9743
AAEL010311	conserved hypothetical protein	29.344	30.5274	26.7697	26.3017	19.9717	20.825	14.9904	22.1498
AAEL010529	conserved hypothetical protein	42.2388	58.6533	31.8033	38.5851	5.89398	4.48795	1.25293	55.3559
AAEL010687	conserved hypothetical protein	0.805907	0.469695	0.362607	0.34894	2.03549	1.17926	50.3745	19.2281
AAEL010707	conserved hypothetical protein	0.038914	0	0.719423	53.5959	0.035971	0.039144	0.091698	0.06804
AAEL010724	conserved hypothetical protein	19.2548	18.8506	54.4773	39.6041	50.1134	51.8119	256.015	128.757
AAEL010845	conserved hypothetical protein	17.2864	15.506	27.03	29.175	17.5566	17.4288	3.33464	10.6253
AAEL010920	conserved hypothetical protein	1.09345	2.3154	0.36334	0.170701	1.35587	1.96301	5285.42	2325.52
AAEL010956	conserved hypothetical protein	2.0979	1.7489	27.3692	4.61521	6.94509	7.05771	5.16244	5.17611
AAEL011180	conserved hypothetical protein	2.20289	2.308	2.59765	2.50215	59.2155	16.6367	3737.28	1219.67
AAEL011438	conserved hypothetical protein	7.14195	8.95078	11.0278	9.66289	11.2016	10.3438	1.53322	6.8867
AAEL011477	conserved hypothetical protein	9.36958	8.34115	6.09977	7.36121	10.0201	10.2052	1.0063	5.22743
AAEL011628	conserved hypothetical protein	1.29235	2.33623	0.217677	0.810996	0.880725	2.5278	6.98621	0.880451
AAEL011664	conserved hypothetical protein	16.4713	16.2706	26.9016	25.3488	11.6615	12.8895	1.6229	9.86238
AAEL011851	conserved hypothetical protein	14.6979	15.9789	9.3184	9.82344	19.4047	20.4982	3.3131	8.82124
AAEL011876	conserved hypothetical protein	28.3613	28.9182	20.9138	29.4983	27.3725	26.3876	10.392	18.8129
AAEL011960	conserved hypothetical protein	24.5209	23.7115	21.8781	23.2126	37.969	36.7475	8.59202	15.4834
AAEL012073	conserved hypothetical protein	8.86123	8.21216	13.7356	13.5614	8.99623	10.8833	2.18904	8.34851
AAEL012302	conserved hypothetical protein	2.6401	2.63913	1.56838	1.96313	3.29145	3.45186	38.5541	13.2674
AAEL012357	conserved hypothetical protein	103.383	107.682	29.9505	39.7012	139.206	111.461	1.34403	47.1554
AAEL012427	conserved hypothetical protein	200.573	193	267.747	294.465	102.579	110.155	66.232	129.091
AAEL012511	conserved hypothetical protein	109.583	137.988	40.9175	40.3096	39.3556	45.8275	113.067	118.834
AAEL012610	conserved hypothetical protein	32.0479	36.5316	98.6244	61.8436	33.0866	32.5125	154.428	82.1357
AAEL012618	conserved hypothetical protein	20.5723	20.9288	25.9805	19.9754	36.7211	34.8137	4.82161	11.4741
AAEL012742	conserved hypothetical protein	52.968	48.842	23.757	21.2142	25.943	25.0251	44.4169	50.6231
AAEL012747	conserved hypothetical protein	15.0639	14.6985	18.5417	17.8751	23.1015	21.2026	4.15743	10.8572
AAEL012838	conserved hypothetical protein	2.81433	2.49656	3.26186	2.64993	1.00163	1.09436	0.615768	5.03816
AAEL012859	conserved hypothetical protein	13.3605	9.1374	4.12915	4.26775	2.11909	2.12621	115.013	46.1076
AAEL012866	conserved hypothetical protein	1.3376	1.77868	0.636153	0.930648	2.45346	1.96268	1220.83	538.64
AAEL012891	conserved hypothetical protein	43.6504	26.2483	31.7548	32.4157	44.0905	48.6549	44.7479	32.7131
AAEL012993	conserved hypothetical protein	19.0692	22.8868	25.7707	21.7795	23.9345	22.0844	7.99999	14.7436
AAEL013075	conserved hypothetical protein	252.655	277.783	362.888	236.732	310.19	267.391	38.8356	142.383
AAEL013110	conserved hypothetical protein	12.8985	14.2377	35.3479	21.5356	13.373	12.5224	0.54227	10.8802

AAEL013118	conserved hypothetical protein	15.3067	26.4956	10.0646	9.80196	7.97806	9.61659	97017	53989.3
AAEL013176	conserved hypothetical protein	61.7536	50.2883	74.4688	66.169	87.2365	88.5172	22.6707	43.9274
AAEL013184	conserved hypothetical protein	0.68343	0.507688	6.20163	4.76444	0.896716	1.43195	59.1671	23.1503
AAEL013262	conserved hypothetical protein	7.53352	13.8006	36.5345	18.34	8.00805	9.63715	13.5533	23.6103
AAEL013304	conserved hypothetical protein	17.0351	27.9721	20.2586	40.3954	7.64894	7.31434	412.854	226.656
AAEL013310	conserved hypothetical protein	32.0077	27.8009	25.2575	26.5191	25.2542	22.7217	7.93353	18.7583
AAEL013429	conserved hypothetical protein	0.624177	0.450376	1.519	1.45207	0.968693	1.32215	41.8915	18.0629
AAEL013524	conserved hypothetical protein	4.70339	3.46099	80.1777	233.604	3.14288	5.15026	1.63266	2.61229
AAEL013615	conserved hypothetical protein	16.5985	14.742	11.2577	13.1264	11.2391	10.84	5.59384	11.2499
AAEL013852	conserved hypothetical protein	21.3989	18.4685	52.8716	52.7179	9.12441	8.85217	0.49512	6.80064
AAEL013965	conserved hypothetical protein	19.8281	20.545	19.1693	18.0215	21.6271	20.8316	6.39094	12.6009
AAEL014132	conserved hypothetical protein	0	0	0.064715	0.042984	1.40637	0.489098	42.2492	9.99861
AAEL014180	conserved hypothetical protein	26.0068	27.2217	12.2433	13.6495	38.6308	37.6981	0.30306	13.7546
AAEL014274	conserved hypothetical protein	1.02563	0.983391	8.16792	26.4909	2.53108	2.09222	2.31348	1.76752
AAEL014431	conserved hypothetical protein	0.094443	0.036827	0.790495	84.8415	0.078396	0	0	0
AAEL014513	conserved hypothetical protein	13.3417	11.1244	15.9293	18.7288	22.2117	22.9137	5.83324	11.2657
AAEL014657	conserved hypothetical protein	24.858	24.8123	37.371	25.2768	35.8033	35.6092	145.459	63.1849
AAEL014825	conserved hypothetical protein	23.3878	18.7049	34.2339	28.9366	18.8441	17.3048	1.56503	10.5454
AAEL014960	conserved hypothetical protein	12.9402	14.7381	11.9126	11.0953	12.5433	8.62136	71.1222	30.0057
AAEL015064	conserved hypothetical protein	549.896	547.29	586.608	559.759	362.566	374.574	305.704	392.809
AAEL000262	conserved hypothetical protein	64.611	63.4866	109.657	104.317	45.621	44.8571	15.68	39.7998
AAEL011966	conserved hypothetical protein	15.3986	15.9936	11.5934	11.0866	20.1849	22.0973	0.59949	5.14927
AAEL011862	conserved hypothetical protein	15.9166	16.4901	25.5203	24.0191	14.4919	13.6386	8.47604	14.3349
AAEL005848	conserved hypothetical protein	24.7017	22.3843	32.0726	31.9798	21.6017	21.0203	8.8788	16.4454
AAEL002446	conserved hypothetical protein	20.7479	20.1559	25.0034	21.7275	16.9139	15.4945	5.92437	15.8988
AAEL005342	conserved hypothetical protein	17.3568	13.5686	9.42049	9.84251	10.2936	9.26283	90.4557	44.3359
AAEL013300	conserved hypothetical protein	31.7826	33.6361	15.9542	17.9232	28.5168	31.1887	0.431533	20.496
AAEL014194	conserved hypothetical protein	39.7002	34.9584	37.3449	42.0137	35.6037	37.1407	13.8547	27.3055
AAEL000407	conserved hypothetical protein	44.6303	60.4747	46.6136	44.3384	43.2311	41.4135	41.566	66.8065
AAEL001511	conserved hypothetical protein	160.423	169.6	302.843	193.506	114.006	114.315	17.5397	88.964
AAEL013716	conserved hypothetical protein	79.785	77.3158	50.2901	48.165	55.7909	60.046	52.2725	57.8485
AAEL001561	conserved hypothetical protein	22.1197	18.8183	16.1907	13.6361	29.9922	26.7168	109.216	51.0015
AAEL004908	conserved hypothetical protein	0	0.083413	8.01264	61.2342	0.18457	0.142985	0	0
AAEL003750	conserved hypothetical protein	649.814	657.685	518.223	536.296	1100.46	1097.39	151.778	368.205
AAEL005843	conserved hypothetical protein	71.2375	66.8856	62.0049	64.8981	63.4537	0	32.4158	47.9056

AAEL000260	conserved hypothetical protein	14.8552	13.6559	22.4077	18.9118	16.4562	18.5299	1.43991	7.94785
AAEL006940	conserved hypothetical protein	70.6509	66.132	78.1635	67.5898	67.385	69.1645	28.2704	46.884
AAEL013197	conserved hypothetical protein	0.072686	0.168155	0.260533	0.242767	0.920618	0.214415	817.704	276.108
AAEL014318	conserved hypothetical protein	24.5273	21.8296	158.163	664.064	22.2667	24.8771	8.86151	15.2707
AAEL001085	conserved hypothetical protein	38.5867	37.3742	41.9776	33.9799	31.0697	38.2762	13.1015	47.0504
AAEL001325	conserved hypothetical protein	11.2673	10.8352	21.469	25.1207	34.3554	28.9536	0.499239	5.10906
AAEL009427	conserved hypothetical protein	53.1043	54.7476	48.0713	51.0458	48.9985	49.2157	9.95922	30.235
AAEL013987	conserved hypothetical protein	152.778	137.455	126.441	243.125	118.469	60.9019	297.589	194.925
AAEL000204	conserved hypothetical protein	27.5757	25.6621	36.2072	39.3731	26.5173	27.2677	170.898	88.6513
AAEL003864	conserved hypothetical protein	29.0176	24.7248	29.1754	25.9687	45.7449	50.681	7.94868	17.7684
AAEL012708	conserved hypothetical protein	19.331	19.5277	33.4202	32.4608	15.2075	16.4071	0.315357	10.8368
AAEL011205	conserved hypothetical protein	37.4016	30.3266	27.9798	23.3595	19.6286	19.2872	2.2763	19.7304
AAEL009165	conserved hypothetical protein	1.41651	2.85013	0.639139	0.738797	0.080378	1.4032	7076.4	4899.15
AAEL001247	conserved hypothetical protein	77.2151	83.0291	109.786	86.6091	116.527	112.171	256.568	138.308
AAEL010574	conserved hypothetical protein	85.4604	94.1587	66.8376	68.3332	77.7848	78.6909	80.6484	108.338
AAEL004042	conserved hypothetical protein	80.5925	95.3488	38.7459	51.0984	49.6826	49.7346	52.5055	73.7695
AAEL012429	conserved hypothetical protein	4.17503	4.04235	21.9834	7.0125	5.48813	5.01176	8.43743	7.53429
AAEL005270	conserved hypothetical protein	1.39885	0.805271	2.34458	1.57817	8.25023	8.20281	1536.78	599.913
AAEL002805	conserved hypothetical protein	21.4783	27.1815	11.4547	11.7	18.6736	19.6988	12.1956	19.4282
AAEL002157	conserved hypothetical protein	9.5529	8.39455	7.90413	8.12231	17.5318	17.6795	2.02109	6.88348
AAEL002195	conserved hypothetical protein	14.9505	13.8642	18.6695	17.7672	10.2747	10.9688	1.21474	7.68294
AAEL001293	conserved hypothetical protein	107.057	117.692	16.5382	70.9498	14.1175	12.0045	256.484	152.125
AAEL011017	conserved hypothetical protein	14.2329	15.9552	9.04173	15.3782	15.1224	13.4685	81.409	38.3487
AAEL001635	conserved hypothetical protein	18.8078	16.8565	27.9933	25.0912	15.3581	13.843	1.59725	10.8095
AAEL010843	conserved hypothetical protein	233	252.7	261.411	233.106	198.434	185.634	425.684	256.813
AAEL000837	conserved hypothetical protein	0	0.207368	53.7461	276.444	0.163604	0	0.276717	0
AAEL015577	conserved hypothetical protein	5.94681	10.3625	11.1575	8.92232	13.1048	14.71	334.683	115.743
AAEL000847	conserved hypothetical protein	41.5896	42.9595	28.6355	26.577	49.3868	47.4931	36.4243	43.7636
AAEL010874	conserved hypothetical protein	0	0	5.9055	80.3902	0	0	0	0
AAEL004436	conserved hypothetical protein	93.011	92.7955	14.068	18.8346	61.8942	62.9594	54.9231	70.6823
AAEL013267	conserved hypothetical protein	0.102935	0.198644	0.187059	0.144515	1.56359	0.945889	85.8547	38.7457
AAEL001569	conserved hypothetical protein	167.336	154.294	178.753	179.972	129.029	136.179	900.897	547.345
AAEL003511	conserved hypothetical protein	0.260097	0.220982	73.9442	1150.04	0.118723	0.194026	0.471035	0.570492
AAEL015595	conserved hypothetical protein	81.1926	84.4219	58.0957	67.058	85.3756	93.7279	259.259	104.52
AAEL008086	conserved hypothetical protein	9.18	11.7157	20.5712	12.8209	8.43038	5.77199	2.70066	10.3828

AAEL000276	conserved hypothetical protein	39.909	38.6721	28.5551	31.792	37.192	38.4678	18.9512	29.3611
AAEL001179	conserved hypothetical protein	0	0	0.298558	14.456	0	0	0	0
AAEL014214	conserved hypothetical protein	0.643336	0.565513	0.739758	0.718227	1.92753	0.801214	76.7368	36.8654
AAEL005874	conserved hypothetical protein	25.5305	21.0753	27.7816	50.2241	20.9492	20.0102	33.119	33.6936
AAEL007685	conserved hypothetical protein	39.9006	39.9348	47.1278	41.3194	27.8581	28.052	17.0113	24.5843
AAEL013816	conserved hypothetical protein	19.1817	17.6238	25.1738	23.383	11.2045	10.7769	6.35663	12.8778
AAEL003191	conserved hypothetical protein	10.6254	11.9314	18.1139	15.5419	12.016	11.0008	0.146191	4.85647
AAEL011330	conserved hypothetical protein	61.1921	53.6275	180.081	154.85	25.928	27.5833	1.72249	34.3251
AAEL011300	conserved hypothetical protein	91.0712	65.6801	0.251709	0.451062	0.489	0.316776	0.141663	36.593
AAEL008259	conserved hypothetical protein	62.9326	65.6259	23.5265	23.7082	96.1776	99.6014	25.53	36.4097
AAEL001447	conserved hypothetical protein	37.9636	37.3174	24.6941	23.885	33.3834	34.4399	14.1186	24.2642
AAEL005558	conserved hypothetical protein	95.1549	96.2595	45.0317	54.6011	91.977	92.2026	325.767	167.799
AAEL000102	conserved hypothetical protein	10.9439	12.487	19.3916	18.7345	19.8237	18.4648	80.1969	34.3398
AAEL000019	conserved hypothetical protein	4.76343	4.08605	38.6094	34.6815	106.651	108.621	5.90883	19.1931
AAEL008678	conserved hypothetical protein	55.4643	48.7179	117.495	109.401	38.1775	44.1602	1.19909	34.0874
AAEL010097	conserved hypothetical protein	977.487	871.946	1557.82	1392.4	281.124	280.847	11.2781	316.059
AAEL006407	conserved hypothetical protein	34.2451	27.8821	39.9814	50.122	17.1206	17.3295	1.80521	16.7088
AAEL006632	conserved hypothetical protein	16.164	15.642	37.6585	32.3489	8.23662	7.97269	4.00287	9.1684
AAEL002105	conserved hypothetical protein	10.5386	10.5972	20.3815	14.5551	8.78327	8.69105	2.94336	8.29274
AAEL005880	conserved hypothetical protein	23.1746	23.4776	32.5971	30.794	20.0069	24.2128	16.7458	24.582
AAEL002811	conserved hypothetical protein	6.99262	1.80709	4.32106	3.47412	6.21213	7.2441	2081.5	960.054
AAEL012860	conserved hypothetical protein	12.7046	17.4346	23.2352	11.974	10.5572	11.0672	291.489	141.298
AAEL013800	conserved hypothetical protein	12.3749	14.3909	18.1031	14.6945	16.8849	17.6334	50.6014	22.9402
AAEL003445	conserved hypothetical protein	6.37702	6.52537	13.7339	10.8032	9.85496	8.42344	61.1506	24.9121
AAEL001594	conserved hypothetical protein	13.0766	9.46178	8.05278	13.6316	4.19848	4.11334	0.697591	4.53753
AAEL000406	conserved hypothetical protein	0.054073	0.061883	0.059548	0.022979	2.61307	0.429676	32.6098	10.728
AAEL001301	conserved hypothetical protein	0	0.434249	0.225384	0.156505	0.168727	0.061379	701.698	404.629
AAEL004863	conserved hypothetical protein	8.57695	8.54027	2.9845	3.4865	27.5662	30.4234	0.445476	4.37623
AAEL007792	conserved hypothetical protein	0.183495	0.427666	0.290236	0.290589	1.38887	1.35923	74.8677	29.3255
AAEL005543	conserved hypothetical protein	0.178457	0.034912	0.088508	0.359771	8.81264	1.12063	206.605	43.2321
AAEL006847	conserved hypothetical protein	64.2531	60.3284	97.1241	96.4852	61.3087	60.5374	37.6507	45.0371
AAEL010944	conserved hypothetical protein	10.7396	11.519	17.8799	39.1617	12.8042	14.0889	0.830351	4.85633
AAEL013984	conserved hypothetical protein	7.91455	8.79546	40.9705	33.7685	86.1186	80.8474	0.390142	4.51013
AAEL007863	conserved hypothetical protein	15.9482	15.0823	20.4819	18.2602	17.4118	16.8051	6.20795	11.7999
AAEL010684	conserved hypothetical protein	36.7166	33.9314	66.9309	118.813	35.0369	32.4179	64.4726	38.6664

AAEL006143	conserved hypothetical protein	4.99039	4.11302	360.684	572.509	10.0827	12.6361	2.14059	3.09262
AAEL010242	conserved hypothetical protein	0	0	0	0.109799	0.113428	0.368621	6.95734	0
AAEL011955	conserved hypothetical protein	25.7766	27.331	35.9201	34.1568	27.2951	30.1386	6.8801	14.184
AAEL010345	conserved hypothetical protein	78.4153	67.4163	43.8079	43.7896	31.7011	34.2707	33.8765	56.856
AAEL010731	conserved hypothetical protein	26.1243	22.887	29.9508	30.2819	53.0911	55.2888	129.404	59.5741
AAEL001187	conserved hypothetical protein	42.8699	39.4719	57.1068	48.2843	45.0739	42.8822	16.1156	29.9213
AAEL010412	conserved hypothetical protein	20.2288	17.9381	49.1125	26.7906	22.97	20.8779	4.51494	14.323
AAEL015357	conserved hypothetical protein	3.45319	3.10553	0	0.094747	0	0.070271	121.511	56.6184
AAEL001998	conserved hypothetical protein	50.8181	39.0323	26.099	25.9814	102.24	90.4596	1281.41	588.757
AAEL013202	conserved hypothetical protein	8.83955	11.9109	12.5095	10.6654	7.8238	12.7027	3.90877	10.807
AAEL004786	conserved hypothetical protein	0.815006	1.17762	0.142864	0.145968	4.74807	1.16966	76.2036	28.0725
AAEL005602	conserved hypothetical protein	50.0789	37.6417	60.5457	56.9706	35.6088	43.4269	0.543194	27.5204
AAEL006011	conserved hypothetical protein	0	0	0	1.39084	2.29486	1.65645	1505.43	607.053
AAEL000944	conserved hypothetical protein	155.371	193.907	127.97	112.744	75.751	75.9291	134.911	147.773
AAEL008580	conserved hypothetical protein	75.9332	76.9904	87.5914	78.0589	65.082	70.7463	1.14649	35.5379
AAEL014039	conserved hypothetical protein	404.415	468.002	425.399	377.726	635.338	626.312	1098.31	740.22
AAEL001499	conserved hypothetical protein	0.069228	0.226022	0.310636	0.132229	0.283262	0.174473	74.884	32.196
AAEL013775	conserved hypothetical protein	3.33724	2.75599	3.89447	3.80444	38.1178	40.5854	13636.2	5592.98
AAEL010338	conserved hypothetical protein	77.4793	51.801	2.47859	2.36604	9.56629	9.25519	4770.84	2254.41
AAEL012650	conserved hypothetical protein	6.60588	0.228181	0.624621	0.452781	0.058515	0.634689	26.209	0.986629
AAEL015379	conserved hypothetical protein	30.2663	33.4853	47.8727	47.2838	35.1876	38.9536	115.939	60.9657
AAEL004980	conserved hypothetical protein	50.6206	44.22	70.046	68.9162	34.5334	35.6726	20.8632	32.6315
AAEL006754	conserved hypothetical protein	1.08782	1.18229	0.968501	1.03636	5.87109	2.53963	49.22	21.3768
AAEL007692	conserved hypothetical protein	62.1856	59.5749	51.6685	52.2652	42.0219	39.9893	3.01117	26.8392
AAEL011203	conserved hypothetical protein	0.43401	0.294183	0.090845	1.04569	3.86549	0.826887	46.8164	10.9869
AAEL013122	conserved hypothetical protein	0.079035	0.18364	131.12	546.117	0.286626	0.233671	1.29425	0.413464
AAEL012147	conserved hypothetical protein	21.0229	19.186	18.5342	17.3289	22.2066	22.1478	3.10195	10.9899
AAEL012932	conserved hypothetical protein	45.5488	56.9103	16.8355	15.4001	52.1151	51.2898	23.441	35.7509
AAEL004882	conserved hypothetical protein	17.1757	16.6743	27.8465	23.477	19.3976	19.521	5.70213	12.3763
AAEL012676	conserved hypothetical protein	17.0927	16.3865	24.5014	37.9531	5.75153	5.40633	4.56376	11.3067
AAEL001898	conserved hypothetical protein	62.1123	60.6622	33.4568	47.6468	75.7952	71.1696	24.9605	35.8515
AAEL001129	conserved hypothetical protein	39.2065	40.1475	66.597	36.1544	43.4575	44.7342	13.3506	26.6452
AAEL012684	conserved hypothetical protein	304.978	461.875	97.1905	115.978	151.704	147.038	454.694	400.706
AAEL005259	conserved hypothetical protein	5.3851	6.15431	7.51962	8.10841	10.8674	11.9478	52.0546	22.8352
AAEL015240	conserved hypothetical protein	28.0816	11.5989	17.1942	29.5685	17.7603	24.3228	9.11818	14.6435

AAEL005588	conserved hypothetical protein	15.2942	13.1263	0	0	0.08946	0	0	6.35559
AAEL013394	conserved hypothetical protein	48.6118	42.0464	62.379	64.6362	35.9628	38.6123	31.6425	37.2057
AAEL013813	conserved hypothetical protein	6.92477	7.20154	3.50901	4.62944	12.16	14.3662	0.141904	4.80224
AAEL010280	conserved hypothetical protein	43.7332	64.4719	9.04639	10.4573	9.13044	10.9921	14.4605	20.7769
AAEL007045	conserved hypothetical protein	10.8018	11.5807	22.4474	19.7123	5.19399	5.37204	0.57921	6.73596
AAEL008671	conserved hypothetical protein	58.1035	57.014	65.6368	64.1924	41.7466	41.7829	189.36	83.7251
AAEL001621	conserved hypothetical protein	0.089381	0.104631	0	0.07683	0	0.088086	324.967	311.729
AAEL014701	conserved hypothetical protein	41.8727	42.2231	53.0775	44.3912	49.4061	47.0236	13.6146	27.3931
AAEL009927	conserved hypothetical protein	12.4256	12.8423	11.6668	9.31412	33.0568	35.7057	0.408088	6.28126
AAEL011082	conserved hypothetical protein	12.8407	12.9853	16.4314	15.2927	14.973	17.31	6.10058	12.1524
AAEL013211	conserved hypothetical protein	51.6477	50.4235	77.1973	74.1903	64.716	65.8716	0.723607	26.3539
AAEL007075	conserved hypothetical protein	49.3126	53.1729	93.3385	81.068	65.0334	68.678	14.8011	34.0254
AAEL005962	conserved hypothetical protein	14.2775	14.2314	23.4402	14.6516	20.3173	20.5679	3.06322	10.344
AAEL013864	conserved hypothetical protein	18.1713	14.8206	0.701737	1.78431	1.54009	1.08706	0.503842	10.9344
AAEL003467	conserved hypothetical protein	26.4221	20.9051	75.6492	58.623	87.5469	74.495	0.670689	6.2212
AAEL006971	conserved hypothetical protein	1.49723	2.82502	2.17113	1.62215	9.2835	37.6244	0	1.43092
AAEL000401	conserved hypothetical protein	12.5244	9.93065	25.9286	19.1556	6.8136	7.68062	2.819	8.19934
AAEL000774	conserved hypothetical protein	11.1087	12.7166	6.96075	5.72923	23.4141	17.523	0.115551	5.05191
AAEL003688	conserved hypothetical protein	29.9372	25.4365	122.809	103.897	14.511	14.9364	11.1288	19.4294
AAEL011101	conserved hypothetical protein	94.1763	95.4197	67.7134	76.0082	95.951	100.985	17.8026	50.2271
AAEL000698	conserved hypothetical protein	13.9329	12.6645	16.2065	17.7453	5.12919	5.88184	0.050117	9.6971
AAEL007465	conserved hypothetical protein	0.935373	0.66341	10.3533	3.12359	1.07832	0.855102	0.280052	0.50731
AAEL008469	conserved hypothetical protein	48.3023	44.9302	64.0741	54.8353	96.1269	99.1104	23.0717	45.609
AAEL012635	conserved hypothetical protein	0.111572	0.065054	0.060765	0.070317	0.679408	0.697575	114.295	53.4159
AAEL001495	conserved hypothetical protein	119.117	125.09	134.198	139.872	154.554	153.459	10.4033	56.9859
AAEL003451	conserved hypothetical protein	16.5178	16.7226	28.4731	23.8238	19.6829	17.5104	1.10634	8.12914
AAEL000956	conserved hypothetical protein	39.0314	32.1176	151.815	77.1466	21.7025	21.3855	11.3087	21.1582
AAEL000691	conserved hypothetical protein	173.658	197.093	90.6769	107.917	315.932	295.647	27.3555	93.4735
AAEL010254	conserved hypothetical protein	21.9174	19.824	18.1817	19.5054	13.5704	14.0892	6.84764	13.5169
AAEL003457	conserved hypothetical protein	28.1459	41.1071	40.5423	44.2678	54.6273	46.2386	0.30538	7.58075
AAEL014366	conserved hypothetical protein	6.08921	6.64458	6.36413	23.8024	10.3257	10.6329	8.34463	7.48048
AAEL010081	conserved hypothetical protein	42.2956	34.5206	92.9955	91.1467	13.6006	14.7302	10.4292	17.2553
AAEL010253	conserved hypothetical protein	11.2533	7.79666	11.0792	11.8268	2.54978	2.2045	1.30749	6.12155
AAEL002898	conserved hypothetical protein	64.7078	74.1841	68.8696	74.1151	41.0941	44.0563	12.5821	44.0152
AAEL007328	conserved hypothetical protein	28.9631	29.4364	24.3416	25.57	37.1473	37.1427	12.9723	19.863

AAEL001722 conserved hypothetical protein	56.1169	64.6949	1.60735	1.40106	2.90329	1.8744	0.919568	39.2315
AAEL014771 conserved hypothetical protein	14.2401	13.1485	26.3389	22.6768	14.0041	13.2087	60.7694	27.931
AAEL009097 conserved hypothetical protein	67.5957	20.0833	2.47005	14.7804	5.97057	5.26919	0.265645	8.73125
AAEL011301 conserved hypothetical protein	33.1808	30.8648	51.1794	48.0292	27.2123	29.3232	5.54261	14.6368
AAEL008088 conserved hypothetical protein	26.794	28.0703	60.794	54.2841	22.0846	23.3183	3.80102	15.901
AAEL000459 conserved hypothetical protein	194.211	168.645	87.0874	104.978	104.826	107.718	165.179	161.739
AAEL008478 conserved hypothetical protein	49.1752	49.0675	17.2481	29.807	8.22252	5.19223	496.038	257.006
AAEL004920 conserved hypothetical protein	52.6163	56.6249	42.3508	34.683	100.716	101.898	15.3717	30.4306
AAEL008291 conserved hypothetical protein	50.5458	55.553	41.8615	38.0643	50.922	54.015	154.643	83.3682
AAEL000940 conserved hypothetical protein	0.725176	0.148278	2.30141	1.20591	1.67678	2.22224	1811.42	832.121
AAEL008176 conserved hypothetical protein	50.0161	61.8149	8.21726	6.19431	71.6551	84.2082	681.888	326.142
AAEL003542 conserved hypothetical protein	27.6903	25.6601	29.0099	28.8264	14.6768	16.6003	1.45513	16.868
AAEL006053 conserved hypothetical protein	1.39847	1.08578	0.925307	1.64528	2.90078	4.75119	2252.92	1187.7
AAEL009662 conserved hypothetical protein	15.3967	16.2234	9.55995	9.48273	22.824	22.1223	3.40047	10.1768
AAEL000476 conserved hypothetical protein	12.6488	12.0843	19.2594	21.1118	8.7917	9.13235	2.29178	9.63343
AAEL008419 conserved hypothetical protein	14.5837	15.912	14.5061	13.5944	13.6091	13.7873	4.40434	9.86717
AAEL007238 conserved hypothetical protein	8.7255	6.38832	9.31206	7.75058	6.82038	4.17332	77.4461	24.5239
AAEL000730 conserved hypothetical protein	35.5993	34.7522	10.7433	16.426	33.6286	33.7793	13.8405	21.9164
AAEL004846 conserved hypothetical protein	30.3136	28.4124	24.5229	27.7497	18.238	20.5316	24.8928	30.7746
AAEL009043 conserved hypothetical protein	11.4255	14.5422	5.48136	9.17026	3.24244	3.81975	85.0083	43.1067
AAEL009372 conserved hypothetical protein	38.8559	40.9965	51.8647	50.3727	49.005	44.7722	14.3077	28.1869
AAEL014054 conserved hypothetical protein	36.7961	33.6269	17.6471	28.3998	19.1581	18.0452	6.22233	22.4083
AAEL014876 conserved hypothetical protein	1.15113	0.572981	941.639	3038.58	0.582554	0.703059	1.15478	0.975255
AAEL004826 conserved hypothetical protein	8.09426	10.1236	12.3345	9.85372	2.45075	8.36438	2.43134	12.791
AAEL000923 conserved hypothetical protein	457.961	466.393	291.131	436.805	386.677	406.989	5.45712	212.081
AAEL004234 conserved hypothetical protein	166.189	166.337	322.146	282.925	108.975	111.094	80.3617	106.501
AAEL005312 conserved hypothetical protein	149.664	120.639	196.892	150.429	103.439	115.774	5.11294	91.7529
AAEL006207 conserved hypothetical protein	0.22798	0.073027	0.435705	0.324168	0.58431	0.471478	148.282	80.4913
AAEL014483 conserved hypothetical protein	12.4953	12.535	14.9413	16.8674	11.8062	12.7016	1.30738	6.42686
AAEL009177 conserved hypothetical protein	22.7669	21.9395	34.9412	29.5553	28.5603	29.4176	5.84529	13.3991
AAEL010061 conserved hypothetical protein	1.02611	0.897012	0.939016	0.713007	1.266	1.37841	46.0054	19.554
AAEL001369 conserved hypothetical protein	10.7472	10.6258	3.28594	5.31258	4.94657	8.28172	3.21071	8.26603
AAEL004026 conserved hypothetical protein	30.1908	29.2261	10.8126	16.2506	29.3425	31.0727	6.58774	22.5657
AAEL009393 conserved hypothetical protein	18.5942	17.4615	23.0927	29.9068	24.0152	23.2085	5.51263	10.9127
AAEL009985 conserved hypothetical protein	5.2814	4.26255	8.50638	13.4191	225.695	48.2574	35236.2	11501.6

AAEL002483 conserved hypothetical protein	27.2841	31.9439	16.079	14.1976	27.4758	26.5422	8.5522	20.9547
AAEL001315 conserved hypothetical protein	17.0438	17.8294	27.5004	24.7508	20.7917	21.9099	3.19433	10.3108
AAEL008177 conserved hypothetical protein	30.2286	30.0068	16.7739	24.3071	31.9764	29.8822	5.70145	16.8322
AAEL010535 conserved hypothetical protein	49.7785	52.2537	61.9696	53.8163	71.0636	71.8658	35.328	41.4991
AAEL007584 conserved hypothetical protein	75.2639	57.9588	76.1105	87.7165	51.0366	56.2277	1.00163	31.3133
AAEL014146 conserved hypothetical protein	11.5447	11.2024	12.5335	7.16302	14.2788	12.869	8.89115	17.364
AAEL007133 conserved hypothetical protein	37.35	33.4581	22.1819	26.9265	33.9617	35.6695	0.712651	26.6067
AAEL010407 conserved hypothetical protein	38.0213	34.3319	40.207	33.7725	13.0242	13.2547	2.40835	20.5698
AAEL002406 conserved hypothetical protein	52.2358	42.1184	145.247	120.973	129.467	87.3337	353.166	163.803
AAEL005013 conserved hypothetical protein	41.1226	42.6727	39.9607	63.2163	49.5718	43.0579	74.793	35.0532
AAEL001306 conserved hypothetical protein	25.7483	26.9852	187.761	790.694	41.5398	36.2048	0.782386	10.4514
AAEL002322 conserved hypothetical protein	16.2983	17.7783	11.2815	12.6052	23.5163	22.3349	3.35061	11.1233
AAEL007234 conserved hypothetical protein	31.674	31.5185	20.7228	26.0442	36.1703	35.4575	12.8359	20.9369
AAEL008644 conserved hypothetical protein	0.057108	0	11.5887	36.6324	0.051116	0	0	0
AAEL012628 conserved hypothetical protein	16.3149	15.0915	14.2481	17.9092	10.4621	12.0945	3.84897	10.0946
AAEL006549 conserved hypothetical protein	23.3657	23.9547	12.1683	14.0878	27.7883	27.2661	4.66275	13.6757
AAEL004856 conserved hypothetical protein	0.152624	0.087762	140.457	200.867	0	0	0.174077	0
AAEL011710 conserved hypothetical protein	18.8677	22.2246	29.1376	25.6096	13.9688	16.6909	6.80649	17.4594
AAEL001189 conserved hypothetical protein	21.9137	19.0917	81.7904	462.754	20.1818	19.5781	32.8618	20.6426
AAEL002734 conserved hypothetical protein	11.9334	10.8128	13.6019	12.4487	14.9288	16.9258	3.98124	9.04227
AAEL006322 conserved hypothetical protein	89.5454	87.2839	100.521	101.778	95.0987	90.119	195.982	113.497
AAEL005904 conserved hypothetical protein	77.8128	78.1014	124.734	123.976	38.6506	42.8951	14.7242	42.3395
AAEL009744 conserved hypothetical protein	10.6961	10.1489	17.5635	16.9192	9.14787	11.9258	0.481927	7.69107
AAEL002263 conserved hypothetical protein	0.575004	1.61947	1.66004	1.15501	12.0872	11.2517	4929.67	2797
AAEL006867 conserved hypothetical protein	46.1535	45.866	54.9184	51.625	40.8858	44.9504	22.6043	29.3379
AAEL002175 conserved hypothetical protein	33.1692	31.9219	27.0688	25.0338	74.0362	19.001	106.319	38.9296
AAEL003112 conserved hypothetical protein	0.040232	0	0.776021	5.98342	0.221955	0.161616	0.265103	0.445514
AAEL004655 conserved hypothetical protein	13.1389	14.1239	12.1686	15.0455	12.3376	10.9479	2.45894	7.52501
AAEL008365 conserved hypothetical protein	23.478	20.79	18.9744	32.473	44.1988	40.0112	1.36229	8.34624
AAEL011085 conserved hypothetical protein	54.4315	50.9358	43.258	51.9313	32.7312	32.5964	0.637724	26.0288
AAEL013548 conserved hypothetical protein	23.0084	23.9757	32.4943	28.9188	22.3423	22.5024	7.50128	16.0135
AAEL004842 conserved hypothetical protein	19.2068	20.1155	28.2201	22.6457	23.6771	23.0456	5.87695	13.6174
AAEL005265 conserved hypothetical protein	26.4776	25.4238	21.5458	27.0902	28.0898	29.7275	3.36252	15.569
AAEL006406 conserved hypothetical protein	0	0	0.455523	0	0	0	8.25316	0
AAEL004515 conserved hypothetical protein	39.6203	36.7652	74.7562	54.468	29.8185	32.0913	10.1052	29.5769

AAEL011296	conserved hypothetical protein	0	0	0.380225	0	0.971667	1.57457	247.269	84.8602
AAEL006069	conserved hypothetical protein	4.91509	3.70642	3.40157	1.82976	9.78799	7.34606	57.451	18.5956
AAEL006155	conserved hypothetical protein	41.3413	43.9486	45.7896	48.7229	36.0288	34.4833	21.1007	27.8871
AAEL005759	conserved hypothetical protein	0.755041	0.146054	1176.69	3192.21	1.48952	0.997763	1.14784	1.09658
AAEL005032	conserved hypothetical protein	23.5414	18.6097	13.3101	37.0863	24.4103	26.628	39.2452	20.4883
AAEL003944	conserved hypothetical protein	28.3905	32.4723	30.8436	29.3332	55.7772	55.7506	7.74946	17.8031
AAEL012100	conserved hypothetical protein	45.9014	46.0595	17.2221	18.7516	39.6228	40.6039	6.49942	23.3916
AAEL009274	conserved hypothetical protein	140.238	146.822	267.871	199.804	74.018	75.5212	108.735	124.509
AAEL007431	conserved hypothetical protein	131.624	141.862	148.939	129.883	174.129	179.812	53.4919	74.5272
AAEL000318	conserved hypothetical protein	0.070123	0	42.6795	476.398	0	0	0	0.119104
AAEL008112	conserved hypothetical protein	15.1253	15.7373	16.3852	14.9011	11.5228	10.9132	3.77314	9.75597
AAEL015080	conserved hypothetical protein	14.1657	13.7174	38.8589	28.8602	10.8385	11.37	1.43434	12.1806
AAEL008281	conserved hypothetical protein	0.062184	0.148199	0	0.109834	0.279803	0.242469	293.656	154.999
AAEL008034	conserved hypothetical protein	5.48114	6.76352	11.6607	28.9655	8.73971	8.04144	2.08878	2.91231
AAEL012399	conserved hypothetical protein	41.1699	37.1198	6.11462	12.1545	6.41108	8.02503	6.66919	33.2135
AAEL005404	conserved hypothetical protein	1.19211	1.11065	1.35686	7.36562	0.330464	0.341017	0.678387	0.956612
AAEL013635	conserved hypothetical protein	10.8042	11.6803	16.5837	15.0154	7.69842	8.24748	0.779701	6.76324
AAEL006890	conserved hypothetical protein	163.128	180.271	203.137	196.016	259.335	253.119	2.74202	81.626
AAEL008802	conserved hypothetical protein	724.057	661.36	238.934	206.356	256.905	218.641	58.8954	277.772
AAEL015013	conserved hypothetical protein	8.42944	9.33449	12.4044	11.2226	11.0167	9.07942	2.68683	12.7209
AAEL005822	conserved hypothetical protein	12.316	10.5295	21.6778	18.8439	16.9796	16.4776	96.2431	46.8731
AAEL007758	conserved hypothetical protein	7.81146	11.074	4.24795	6.73453	8.02158	9.21123	440.741	185.048
AAEL009155	conserved hypothetical protein	0.436791	0.101334	0	0	1.7189	1.27016	266.948	107.884
AAEL010611	conserved hypothetical protein	43.331	50.9929	44.946	36.1487	72.5429	61.5483	61.024	64.3587
AAEL000211	conserved hypothetical protein	50.3116	53.1844	52.157	51.0052	64.3326	67.8604	20.7926	42.4154
AAEL008894	conserved hypothetical protein	22.5417	23.5685	22.0661	20.5905	23.5599	22.1606	1.59327	12.2967
AAEL013725	conserved hypothetical protein	10.8385	11.2057	10.7095	10.5015	13.9161	15.3566	1.52049	6.73561
AAEL007117	conserved hypothetical protein	0	0	116.67	507.085	0.104477	0.114105	0.284603	0.212296
AAEL011109	conserved hypothetical protein	3.77022	3.455	18.8386	6.09272	7.17945	8.94167	3.286	3.13491
AAEL000984	conserved hypothetical protein	25.0472	27.4039	23.1221	21.761	25.4244	26.6346	1.54012	12.8959
AAEL009467	conserved hypothetical protein	17.4605	11.6708	10.9867	12.1103	7.12648	6.54916	457.659	171.282
AAEL010150	conserved hypothetical protein	10.5059	11.3708	10.0269	10.8358	13.8504	12.1051	0.92813	5.40654
AAEL014908	conserved hypothetical protein	14.2189	14.3119	11.7778	13.0775	19.5308	24.5403	5.21825	11.116
AAEL005353	conserved hypothetical protein	2.45835	3.39794	6.82864	3.67864	10.5755	9.36539	147.716	53.5572
AAEL004969	conserved hypothetical protein	41.3209	57.3103	7.24576	2.56639	24.4232	21.3952	0.978587	21.5308

AAEL007797	conserved hypothetical protein	15.212	14.6328	18.9876	18.0562	13.3383	14.8276	1.68854	6.276
AAEL008070	conserved hypothetical protein	3.06298	6.17125	6.22615	6.17074	7.33783	10.12	6.66714	0.639958
AAEL009500	conserved hypothetical protein	24.9514	21.3552	35.5051	39.913	12.259	12.459	8.68768	19.9856
AAEL002090	conserved hypothetical protein	96.686	95.2539	77.4627	89.1874	93.2259	85.8606	62.6337	71.5922
AAEL012031	conserved hypothetical protein	11.8887	13.4864	20.1244	17.6384	14.2206	13.7438	1.34067	7.18274
AAEL004798	conserved hypothetical protein	0.736245	1.06724	0.245263	1.10921	10.971	4.19995	3343.32	1032.35
AAEL006211	conserved hypothetical protein	22.3992	20.9116	39.781	32.5869	11.6261	12.4169	1.75327	9.4663
AAEL000496	conserved hypothetical protein	0.619175	0.396783	354.828	1526.66	0.480332	0.6014	2.76615	0.729852
AAEL000344	conserved hypothetical protein	0	0.081638	0.105024	28.6944	0.063797	0	0	0.119859
AAEL000500	conserved hypothetical protein	2.35117	4.98795	1.44902	1.41912	3.16072	5.02899	14838.4	8641.12
AAEL000782	conserved hypothetical protein	22.9505	21.858	27.5296	22.0244	24.5112	24.5593	9.75696	16.7052
AAEL000857	conserved hypothetical protein	36.1283	36.4313	36.7824	42.5712	26.9542	28.2964	8.34692	20.9649
AAEL000961	conserved hypothetical protein	137.543	136.762	9.18705	17.6993	119.051	115.279	1.04194	69.4186
AAEL002081	conserved hypothetical protein	121.313	137.128	227.151	163.048	129.453	117.015	77.9972	105.806
AAEL002578	conserved hypothetical protein	0.894645	0.374955	1.91048	2.15496	18.8607	11.9854	1880.53	692.492
AAEL004113	conserved hypothetical protein	17.2097	16.9029	16.2355	15.4587	15.9744	16.6945	7.39355	13.9621
AAEL004775	conserved hypothetical protein	10.9202	9.74046	10.6628	9.18648	6.99513	5.98614	1.05045	4.8941
AAEL004818	conserved hypothetical protein	32.0975	34.8914	30.3336	26.4185	48.1249	46.6262	4.3315	18.6693
AAEL004838	conserved hypothetical protein	42.4589	40.4198	57.5926	49.783	56.6528	54.2319	18.8065	27.275
AAEL005094	conserved hypothetical protein	1.42582	1.47437	4529.5	965.735	0.848395	1.47434	1.75862	1.34514
AAEL005163	conserved hypothetical protein	28.664	31.3222	37.564	29.6489	39.185	41.9607	5.99654	16.2635
AAEL005580	conserved hypothetical protein	20.1693	23.8194	15.6135	20.0576	20.8156	20.7781	16.4653	22.7547
AAEL005737	conserved hypothetical protein	6.2826	6.63502	10.3317	9.35072	8.58771	7.77107	0.296251	3.41172
AAEL006987	conserved hypothetical protein	0.727431	1.08206	0.497543	0.241683	6.87771	1.41966	60.3238	9.79847
AAEL007342	conserved hypothetical protein	4.9691	2.52927	33.616	33.2811	14.6642	18.9136	2273.12	844.841
AAEL008174	conserved hypothetical protein	4.97786	4.48128	5.39507	6.30236	6.46703	6.32353	47.9634	21.5726
AAEL008220	conserved hypothetical protein	18.7742	18.9428	12.863	10.3459	35.2171	35.5827	6.74018	14.6202
AAEL008278	conserved hypothetical protein	0.27968	0.263914	0.418874	0.531643	3.11516	1.42088	278.728	113.656
AAEL008716	conserved hypothetical protein	20.2724	22.4588	18.4946	20.5256	23.5465	22.4608	3.01184	10.5463
AAEL009226	conserved hypothetical protein	0	0	21.2925	8.24224	0	0	0.077771	0
AAEL011807	conserved hypothetical protein	15.7504	14.6009	18.3366	17.6103	14.3121	13.1371	1.9463	7.49943
AAEL012227	conserved hypothetical protein	119.601	120.684	135.044	111.902	160.011	155.03	52.6458	81.912
AAEL013051	conserved hypothetical protein	5.21798	4.21361	5.57176	5.99401	4.54146	3.18412	0.511495	5.71189
AAEL013494	conserved hypothetical protein	59.2984	73.0366	37.5319	33.5031	44.1417	52.5336	57.0889	71.8682
AAEL015288	conserved hypothetical protein	0.224426	0.086668	27.7869	203.839	0.204671	0.074215	0	0.124749

AAEL000144	conserved hypothetical protein	0.206943	0.136506	94.9901	456.745	0.072972	0.21332	0	0.179158
AAEL009114	conserved hypothetical protein	6.55371	6.23971	19.4843	16.3845	27.8975	26.3387	53.8235	22.0243
AAEL006883	conserved hypothetical protein	8.05353	10.3439	5.23937	6.99954	86.6465	14.8245	18.9646	13.9899
AAEL010001	conserved hypothetical protein	14.1122	11.168	15.2129	14.4494	13.424	13.584	3.87487	9.09004
AAEL009755	conserved hypothetical protein	13.5546	12.7969	17.4108	14.5743	11.9374	13.1644	3.51552	8.50003
AAEL003037	conserved hypothetical protein	13.8514	11.2106	13.117	9.14729	23.888	17.701	242.57	95.3758
AAEL006787	conserved hypothetical protein	35.1689	34.1254	52.9157	48.9185	31.8388	32.0419	2.97989	17.0736
AAEL007500	conserved hypothetical protein	1.00689	0.925618	1.26151	7.27707	2.12669	1.82476	1.08044	0.873165
AAEL012671	conserved hypothetical protein	137.182	138.788	69.5383	100.521	128.007	121.596	382.725	222.864
AAEL001651	conserved hypothetical protein	17.8052	18.0631	21.7331	21.0387	15.9288	15.3747	3.42326	10.511
AAEL005826	conserved hypothetical protein	1.35325	1.16245	1.20345	0.585738	3.79899	3.01598	35.8409	13.5942
AAEL013968	conserved hypothetical protein	39.8505	43.7023	34.9706	31.2001	75.5109	72.6666	10.802	25.6143
AAEL002467	conserved hypothetical protein	1.03048	2.28648	0.687604	0.516465	6.56492	15.7208	8343.24	2767.37
AAEL015122	cop9 signalosome complex subunit	3.89964	5.6433	7.07354	6.74362	5.4107	6.76244	1.92279	6.91006
AAEL006560	cop-coated vesicle membrane protein P24	48.2102	51.5086	15.0925	13.5946	18.4014	20.6578	33.9979	43.4791
AAEL000208	copii-coated vesicle membrane protein P24	129.653	170.933	42.1786	33.3374	48.3059	51.9821	195.393	158.419
AAEL013389	coproporphyrinogen iii oxidase	8.06525	9.03915	8.28615	6.36152	10.1228	10.9024	66.8127	28.1615
AAEL007327	cornichon protein, putative	6.71764	7.68103	4.4301	1.4329	10.5295	3.04794	11.187	5.69422
AAEL009249	coronin	1.41485	1.50202	1.07868	0.701717	1.86151	1.7184	58.6622	19.3874
AAEL011982	cortactin	11.1431	11.8281	15.7691	13.4405	12.2319	11.8599	72.8045	32.4868
AAEL007422	cp27, putative	32.119	32.1138	44.0883	38.2531	21.53	23.0229	10.3502	23.3381
AAEL001308	CRAL/TRIO domain-containing protein	86.7033	82.1847	3.71655	6.04693	42.8418	38.7729	2.2013	49.1889
AAEL010027	CRAL/TRIO domain-containing protein	4.92231	4.77064	35.5968	10.57	3.7745	3.44534	122.03	57.1296
AAEL003347	CRAL/TRIO domain-containing protein	7.73885	5.29152	74.0168	180.899	10.829	10.1661	19.3557	7.71238
AAEL006523	crk	27.1613	24.1649	43.4565	37.1217	13.8056	14.1859	8.01161	18.2189
AAEL010008	crotonobetainyl-CoA-hydratase, putative	22.1039	24.2944	26.0441	25.1296	29.1482	29.7569	9.05225	17.6114
AAEL011453	CTL14	1.69791	2.4815	3.77331	3.74298	79.2079	37.8532	2.08347	1.25673
AAEL011446	CTL17	35.2311	45.1961	10.5939	13.6355	27.7288	20.7437	0.477066	13.6996
AAEL002280	ctl2	33.1564	32.5185	9.32948	13.773	23.5403	23.127	1124.75	452.91
AAEL011407	CTL20	0	0.455867	0	0	1.59511	1.53816	51.6562	19.2501
AAEL011408	CTL21	16.0665	19.0594	28.4098	27.1236	35.8844	41.3034	0.410259	7.82736
AAEL017484	CTLGA4	10.3649	7.29883	10.6625	12.5127	1.4725	2.1665	0.495457	5.1103
AAEL005641	CTLGA5	28.5272	33.3462	22.6735	30.8637	100.135	93.7388	2195.88	792.114
AAEL009209	CTLGA6	29.5211	23.7145	24.9407	22.9263	26.4241	27.666	5.34436	14.3807
AAEL017265	CTLGA7	12.5282	12.8521	14.442	12.32	30.5506	42.3668	121.929	39.2994

AAEL011610	CTLGA8	4.19527	6.42063	3.63747	8.9475	46.5348	76.6135	0.694359	1.05351
AAEL000543	CTLMA11	16.9788	12.9912	7.54182	9.39568	14.3583	12.8223	2.91455	7.66042
AAEL011455	CTLMA12	12.8872	8.31682	6.39729	5.4294	92.1932	68.9649	0	6.52511
AAEL005455	ctp synthase	55.579	58.9281	40.4298	38.7202	111.154	113.418	25.6992	36.0747
AAEL007353	cullin	18.5196	17.7854	21.2896	21.7652	16.1782	17.3201	4.91943	11.3135
AAEL006271	CUSOD2	303.433	299.404	645.142	454.918	166.197	172.812	16.8768	121.368
AAEL001751	cyclin	22.2176	24.3514	18.2899	18.4526	42.5105	40.3848	8.68977	19.1536
AAEL000672	cyclin a	137.75	126.392	242.581	194.266	57.9898	63.2973	11.0865	78.9295
AAEL008256	cyclin A3, putative	25.3449	24.0316	30.4916	29.2952	23.3648	24.2082	0.555916	12.6205
AAEL010094	cyclin b	338.019	322.118	708.58	554.171	186.946	204.361	9.31968	179.155
AAEL009572	cyclin B3	102.459	99.1289	134.761	132.028	115.112	118.64	2.39916	46.2658
AAEL011648	cyclin d	15.1164	16.0246	9.6551	14.7242	7.18651	6.13517	0.347654	12.0864
AAEL009057	cyclin e	35.6556	33.9137	54.4688	47.7886	16.2185	16.4237	1.27861	19.8314
AAEL004865	cyclin g	202.035	196.623	186.758	202.904	131.475	142.593	394.09	231.052
AAEL004839	cyclin t	37.3554	33.8679	31.3597	33.7961	33.1959	32.582	13.9409	24.2645
AAEL004492	cyclin-dependent kinases regulatory subunit. putative	93.1914	91.5409	148.26	133.894	68.5762	71.6517	5.77023	43.1563
AAEL005232	cyclin-dependent kinases regulatory subunit. putative	25.8289	25.2507	28.8306	33.1108	13.1539	15.2596	2.03114	11.7149
AAEL003993	cyclohex-1-ene-1-carboxyl-CoA hydratase, putative	131.77	170.102	81.0017	75.4607	137.556	128.535	114.859	101.851
AAEL002031	CYP12F7	1.81684	1.37747	1.53748	1.37278	2.76702	4.0876	376.754	174.257
AAEL006827	CYP12F8	11.5042	8.75704	18.257	18.9318	5.93066	5.74234	0.606482	5.70963
AAEL014412	CYP304B2	0.062562	0.036255	0.065566	0.025188	0.201469	0.188253	116.136	48.7706
AAEL000338	CYP325E3	31.2847	21.0044	0	0.063949	0.056855	0.275533	0.058517	12.9241
AAEL012266	CYP4C38	17.6674	14.8519	25.6283	28.4905	13.73	13.5477	128.323	70.2566
AAEL007816	CYP4D23	5.5204	6.20772	2.20966	1.13239	6.09413	6.09078	512.837	198.095
AAEL007815	CYP4D24	0.106165	0.041223	0.025917	0.03005	0.064318	0	82.9578	39.5237
AAEL008345	CYP4G35	13.8995	18.2285	11.2794	11.7307	40.979	35.4167	0.131207	14.8766
AAEL004054	CYP4G36	151.421	149.829	98.6233	108.234	300.257	267.721	1.39499	57.4788
AAEL007830	CYP4H29	0.75334	0.721593	0.816287	0.385496	1.43289	0.971026	26.6773	9.49859
AAEL007812	CYP4H32	0.072767	0.212755	0.053722	0.061887	0.164036	0.250867	586.655	329.767
AAEL012492	CYP6AA5	1.41074	1.02317	3.44739	2.7503	5.35351	4.96031	147.781	49.2742
AAEL007010	CYP6AG4	2.53131	2.21809	45.0498	26.3583	103.869	114.828	69.1516	40.4331
AAEL007473	CYP6AH1	0.796102	0.963601	0.563043	0.356165	1.57843	3.05351	51.4397	22.4978

AAEL008889	CYP6AL1	6.71137	11.2683	0.601056	0.661148	1.44926	1.77013	0.269595	5.20081
AAEL014893	CYP6BB2	8.47306	6.56482	4.41405	5.46754	4.59992	4.99592	54.6166	16.4596
AAEL017539	CYP6BY1	1.99537	0.361093	0.62771	0.392902	0.349477	1.21493	48.8781	14.615
AAEL002046	CYP6CB1	6.8543	9.72356	122.581	78.8318	47.8242	53.2431	0.927473	7.96863
AAEL009127	CYP6M11	1.63277	1.13026	3.26138	2.7755	4.39662	9.18791	177.303	72.8693
AAEL009128	CYP6M6	3.30637	2.90231	1.91431	1.80929	4.23015	4.03711	82.6651	37.5655
AAEL009129	CYP6Z9	4.34591	3.58666	5.44622	5.2295	20.1334	14.3617	816.945	302.151
AAEL006795	CYP9J15	0.330211	0.213044	0.135593	0.266093	1.89236	1.21576	160.87	54.8601
AAEL006815	CYP9J16	1.04688	1.33294	1.60557	1.79551	4.04219	4.06774	57.3371	24.2331
AAEL014609	CYP9J26	0.214119	0.070888	0.220545	0.050977	0.309442	1.01102	68.4086	32.252
AAEL008846	CYP9J32	0.706573	0.600246	0.33629	0.383303	7.21887	0.463628	27.9002	11.4412
AAEL002638	CYP9J6	1.56747	2.36395	14.5669	18.6619	4.18232	4.30738	81.2144	36.3723
AAEL006811	CYP9J8	0.329365	0.301619	0.756777	0.469821	3.33609	1.72967	264.905	60.6484
AAEL014608	CYP9J8	0.218426	0.063585	0.318078	0.184143	0.718841	0.754165	101.39	19.0028
AAEL014605	CYP9J9	1.59336	1.26394	2.5175	2.70783	6.86864	4.79874	196.999	80.7686
AAEL006793	CYP9J9	1.71308	1.53974	2.79644	2.20983	7.64231	8.9236	424.676	232.746
AAEL014611	CYPJ19	0.73575	0.534352	0.459474	0.460509	1.27286	2.14302	76.5531	26.6706
AAEL007416	cysteine dioxygenase	23.8692	29.1534	12.3975	11.0564	22.4748	21.8491	332.444	203.782
AAEL015445	cysteine dioxygenase	2.04717	3.32505	0.415302	0.39046	0.705002	1.09787	19.9084	31.6458
AAEL008468	cysteine synthase	46.3433	45.0062	12.7429	11.3337	16.2529	14.7875	33.1506	50.1118
AAEL008467	cysteine synthase	0.532888	0.623114	0.160241	0.238531	0.244405	0.401751	79.9386	37.6727
AAEL000356	cysteinech venom protein, putative	0.512102	0.597796	681.618	2969.03	0	0.488371	1.21973	1.81428
AAEL005487	cysteinech venom protein, putative	0.940058	0.704485	1643.33	290.557	0.96161	1.74899	0.389118	0.796048
AAEL000302	cysteinech venom protein, putative	1.1926	2.46656	4563.27	1986.99	0.728446	2.58524	0.749649	1.46835
AAEL008473	cysteinech venom protein, putative	41.0158	35.8291	39.3909	115.255	119.058	95.3581	200.07	67.4867
AAEL000369	cysteinech venom protein, putative	0.122705	0.720531	1220.61	51.9831	0.44147	0.358461	0.535417	0
AAEL000361	cysteinech venom protein, putative	0.792137	0	1021.11	192.053	0	0	0.903256	0
AAEL000379	cysteinech venom protein, putative	0.473115	1.082	5.79577	100.97	2.16882	3.30169	0	0
AAEL005090	cysteinech venom protein, putative	0.479067	0.274717	2537.05	820.118	0.860471	0.46668	0.539813	0
AAEL000333	cysteinech venom protein, putative	0.343029	0	1198.65	237.045	0.150804	0.330751	0.179823	0.56297
AAEL000363	cysteinech venom protein, putative	0.167219	0	1100.67	76.1519	0	0	0.359702	0
AAEL000317	cysteinech venom protein, putative	2.33369	1.70076	3998.76	388.085	0.264764	0.288474	1.36617	2.12318
AAEL010017	cytochrome B5 (cytb5)	199.701	232.34	340.083	246.884	237.688	228.764	663.386	376.877
AAEL004450	cytochrome b5, putative	119.996	133.973	142.968	167.043	122.889	130.579	1204.5	502.38
AAEL012636	cytochrome b5, putative	0.171707	0	0.124467	0.435786	0.538642	0.92184	134.747	52.2226

AAEL014935	cytochrome b5, putative	86.825	148.673	191.804	154.85	108.96	99.5247	351.89	300.188
AAEL011281	cytochrome B561	24.3719	22.6191	16.7757	22.5299	14.5079	18.4545	4.05182	9.14052
AAEL004457	cytochrome c	148.392	180.429	202.433	147.758	187.682	184.886	335.946	282.697
AAEL014944	cytochrome c oxidase polypeptide	215.474	249.985	194.708	160.088	324.02	312.914	707.169	495.486
AAEL013009	cytochrome c oxidase subunit iv	82.1733	102.816	70.3649	64.4156	121.903	140.979	298.473	166.907
AAEL003234	cytochrome c oxidase, subunit VIA, putative	297.309	383.478	297.644	249.309	436.416	432.84	1104.04	707.724
AAEL002737	cytochrome c oxidase, subunit VIIC, putative	99.1718	140.595	133.407	99.099	186.82	157.924	407.125	220.225
AAEL011514	cytochrome c oxidase, subunit VIIC, putative	198.545	211.536	133.742	165.549	184.005	180.378	516.731	429.552
AAEL008880	cytochrome c oxidase,-subunit VIb, putative	234.403	259.865	219.594	203.726	386.777	407.513	1111.58	638.317
AAEL009183	cytochrome oxidase biogenesis protein (oxa1 mitochondrial)	22.1423	23.4238	27.5653	29.8307	32.0835	29.2469	127.433	56.2307
AAEL014614	cytochrome P450	0.203524	0.034251	0.193041	0.098615	0.607048	0.402308	37.7098	15.6706
AAEL015370	cytochrome P450	7.28038	7.56076	0.761055	1.64531	1.42966	1.55701	76.6752	32.2958
AAEL015641	cytochrome P450	1.11099	0.595207	0.520418	0.605089	2.21844	3.66563	67.0855	28.8645
AAEL006824	cytochrome P450	60.6235	71.1047	29.4064	26.6945	95.9986	96.4295	0.360214	12.44
AAEL014830	cytochrome P450	7.77319	6.66857	3.16202	2.54228	9.00243	6.17434	0.347678	4.42535
AAEL009018	cytochrome P450	3.21948	4.91639	73.4003	52.8735	35.6517	33.7572	0.344972	4.57752
AAEL001960	cytochrome P450	1.14492	1.2902	2.12671	3.34711	6.94104	5.1933	270.019	98.8194
AAEL011478	cytoplasmic dynein light chain	213.736	216.503	274.254	266.659	138.7	136.724	98.8227	133.86
AAEL002422	cytoplasmic polyadenylation element binding protein (cpeb)	211.059	222.829	32.7996	49.615	181.874	187.442	1.6968	110.696
AAEL002056	Cytoplasmic tRNA 2-thiolation protein 2	19.1305	16.5547	23.1633	16.7717	18.6003	20.6853	3.64581	9.6696
AAEL005833	cytosolic purine 5-nucleotidase	59.3161	73.8404	21.3525	22.2775	23.4645	20.6039	36.4052	51.4132
AAEL005336	d-3-phosphoglycerate dehydrogenase	109.403	172.111	14.5254	6.78718	66.68	57.5249	1330.1	800.954
AAEL006424	D7	2.70363	1.78793	1.32834	1.08198	1.63645	2.75188	10.5106	0.708489
AAEL008620	D7 protein, putative	165.787	187.961	16.9991	11.5484	63.4465	60.4579	4.15812	60.9514
AAEL006417	D7 protein, putative	0.031032	0	0.142973	0.054671	0.028115	0.152275	14.658	0
AAEL012674	d-amino acid oxidase	2.82478	4.14417	3.82219	3.31953	12.1707	11.7743	77.0995	35.5762
AAEL000164	d-amino acid oxidase	3.18816	3.49763	2.7444	3.25865	4.44634	4.95014	76.4738	28.1437
AAEL000213	d-amino acid oxidase	97.5972	91.8672	17.901	17.0391	18.1391	17.2985	23.0231	31.6921
AAEL012888	dcapl	8.77276	9.77244	9.29318	7.39002	8.46016	7.59439	66.1632	26.3658

AAEL007374	DCE	24.7154	33.2541	11.2422	13.0988	19.7912	20.4172	1.75262	9.00096
AAEL006640	DEAD box ATP-dependent RNA helicase	16.4717	14.9876	10.6341	10.1288	22.5644	23.5671	3.22125	9.52731
AAEL012015	DEAD box ATP-dependent RNA helicase	51.4155	47.4549	76.5022	73.27	43.6018	40.5212	0.77175	23.7174
AAEL005744	DEAD box ATP-dependent RNA helicase	40.0271	44.3008	47.3967	40.2711	141.379	146.858	13.552	25.6352
AAEL004978	DEAD box ATP-dependent RNA helicase	487.137	531.83	388.032	475.9	584.029	552.017	12.5774	249.988
AAEL014904	DEAD box ATP-dependent RNA helicase	14.4461	15.6057	19.9306	17.3243	44.6216	45.8805	4.14658	9.42224
AAEL001216	DEAD box ATP-dependent RNA helicase	228.798	235.638	131.365	138.911	301.638	302.452	73.7203	138.978
AAEL002083	DEAD box ATP-dependent RNA helicase	212.295	220.362	309.204	253.609	355.538	363.572	442.735	313.147
AAEL008500	DEAD box ATP-dependent RNA helicase	725.866	666.532	1104.51	1061.48	328.723	330.995	93.8866	341.059
AAEL010402	DEAD box ATP-dependent RNA helicase	81.8629	86.7919	60.9997	74.4027	87.8916	85.5883	22.498	51.8436
AAEL009285	DEAD box ATP-dependent RNA helicase	18.264	16.5698	21.2573	19.4196	33.7071	31.3016	4.76801	11.2943
AAEL008728	DEAD box ATP-dependent RNA helicase	12.6482	13.662	13.8852	14.6266	15.3007	16.1298	1.56964	7.47604
AAEL010787	DEAD box ATP-dependent RNA helicase	356.757	351.185	468.007	435.039	1813.63	1832.85	472.529	370.873
AAEL009913	DEAD box ATP-dependent RNA helicase	45.0245	49.2584	61.0887	50.7535	59.5929	58.9426	26.4477	36.128
AAEL013359	DEAD box ATP-dependent RNA helicase	380.21	400.73	301.669	263.081	1064.43	1015.4	706.527	502.224
AAEL010317	DEAD box ATP-dependent RNA helicase	30.4953	31.0544	31.0177	28.4662	32.2695	30.6939	6.55938	17.5586
AAEL001876	decapentaplegic, deca	19.5019	21.0469	20.2983	19.3012	21.7591	20.9437	6.83551	14.3351
AAEL003841	DEFA	7.71054	4.13138	24.1906	56.6106	194.688	646.266	63.1225	14.9364
AAEL003832	DEFC	6.03762	3.96583	13.8373	24.6677	72.6505	121.426	6.51415	2.80016
AAEL003857	DEFD	23.135	7.37591	24.2186	24.648	259.918	585.462	113.328	32.7201
AAEL003849	DEFE	0	0	0	0	2.52058	0	204.207	391.537
AAEL009996	deformed wings, putative	16.658	17.2286	23.9525	20.732	15.7532	16.7361	2.9849	10.1719
AAEL007213	delta(9)-desaturase, putative	18.5374	11.5549	117.09	34.2494	2.37055	2.05275	3.13664	6.39957
AAEL014287	deoxycytidylate deaminase	45.8105	45.5603	51.0702	32.622	33.1276	27.9097	2.75714	29.7717
AAEL011245	deoxyhypusine synthase	17.4733	16.6296	18.9391	13.5302	13.9305	15.5103	2.92657	9.57014
AAEL008857	deoxyribonuclease I, putative	1.15311	1.04323	1.04875	0.69469	4.82822	4.09362	1765.14	1104.73
AAEL001641	deoxyribonuclease I, putative	52.3525	50.9105	34.8455	33.0184	155.992	124.434	2.51916	32.9287
AAEL004092	deoxyribonuclease I, putative	0.191659	0.055572	0.070263	0	0.172335	0.328089	466.627	243.572
AAEL002042	deoxyribonuclease I, putative	7.29333	8.59009	4.59847	4.84941	9.642	10.0258	0.117653	3.60386
AAEL012418	deoxyribonuclease ii	23.284	20.9886	50.9153	43.8248	6.89206	6.64714	1.65477	10.7669
AAEL012629	deoxyuridine 5'-triphosphate nucleotidohydrolase	245.948	234.909	329.593	299.789	126.788	118.936	10.1882	150.604
AAEL005579	developmentally regulated GTP-binding protein 1 (drg 1)	45.5574	42.896	37.6243	33.8954	55.1396	54.0698	21.2797	32.1639
AAEL013847	diacylglycerol kinase, zeta, iota	20.5545	21.6007	18.8909	22.8026	24.4492	24.09	4.23183	13.3679

AAEL008878	diacylglycerol o-acyltransferase	82.6732	74.9277	87.1892	85.1329	60.0856	64.8791	539.921	251.664
AAEL013844	diazepam binding inhibitor, putative	121.238	127.425	40.1371	45.1421	81.56	70.5298	13.6321	79.0654
AAEL009214	diazepam binding inhibitor, putative	418.679	433.745	36.8448	69.9884	47.3923	46.3862	4103.43	1986.74
AAEL006836	dihydropteridine reductase	252.778	252.179	408.177	337.922	181.114	174.908	14.7996	119.346
AAEL000354	dimeric dihydrodiol dehydrogenase	13.4327	11.9514	33.5684	34.6004	7.41038	6.45804	0.420211	5.27692
AAEL000834	dimethylaniline monooxygenase	76.8082	79.7054	12.7592	9.26302	65.0359	42.3421	13.0746	52.686
AAEL000797	dimethylaniline monooxygenase	1.14653	0.756624	1.88399	3.31352	2.02939	0.213276	93.8112	41.7422
AAEL000820	dimethylaniline monooxygenase	1.08706	0.752681	0.239453	0.157388	0.407029	0.572995	92.9589	43.3976
AAEL015110	dipeptidyl-peptidase	0	0.045143	0	0	0.016217	0.154939	207.916	99.7666
AAEL010912	dipeptidyl-peptidase	72.0293	67.9231	175.256	168.586	36.8261	41.4027	17.6855	42.8016
AAEL015386	dipeptidyl-peptidase	0.126527	0.187911	0.170194	0.12202	1.25729	1.89834	653.577	359.279
AAEL003678	diphosphoinositol polyphosphate phosphohydrolase, putative	40.0133	40.7246	50.5482	42.7621	39.1113	36.7787	25.3992	33.4475
AAEL002016	disulfide oxidoreductase	26.8423	24.5335	29.0436	27.2356	21.1086	21.6861	11.0026	17.4043
AAEL006166	DNA (cytosine-5)-methyltransferase	21.2053	19.8748	21.0336	19.2955	17.0836	16.7328	6.77047	12.8462
AAEL005597	DNA helicase recq5	42.6887	45.1488	51.5052	48.9312	38.6963	38.3392	7.87464	26.3815
AAEL005858	DNA mismatch repair protein mlh1	11.1173	9.34327	13.0613	13.4759	8.59313	8.98277	0.801777	5.96787
AAEL011780	DNA mismatch repair protein muts	14.6126	12.7133	21.1446	18.8271	7.00662	7.29012	1.13811	7.12374
AAEL014856	DNA mismatch repair protein spellchecker 1	20.1768	19.2476	25.5759	27.9072	16.2291	16.8223	2.16056	8.89476
AAEL001175	DNA photolyase	23.3713	20.8034	30.1882	26.0514	20.5394	23.9896	5.2534	16.7251
AAEL008247	DNA polymerase alpha catalytic subunit	28.6977	24.4198	30.347	35.2462	22.8385	21.7906	2.01904	13.552
AAEL014178	DNA polymerase delta catalytic subunit	25.7945	24.0442	22.9106	25.1129	21.7748	22.3114	2.04952	13.6026
AAEL002785	DNA polymerase epsilon subunit b	20.1766	18.0828	23.9031	25.7408	7.55637	7.93278	1.63887	9.90413
AAEL010085	DNA polymerase epsilon subunit, putative	39.9565	37.0693	65.5885	58.1699	27.8752	28.9597	1.28975	20.0477
AAEL004562	DNA polymerase eta	11.3656	11.539	15.353	14.1557	11.6729	13.3558	0.870337	6.21359
AAEL006715	DNA polymerase iota	31.3969	33.3157	48.6482	44.3232	32.284	31.867	7.99601	20.676
AAEL005888	DNA polymerase theta	66.4237	66.5926	56.206	58.8479	50.0934	54.1063	1.50931	31.9039
AAEL004017	DNA polymerase v	53.728	57.5701	36.3214	30.3841	64.9549	60.6772	7.52462	29.8036
AAEL009851	DNA polymerase zeta catalytic subunit	12.3795	11.086	12.6061	12.258	6.53938	6.32112	1.99366	6.71913
AAEL014314	DNA primase	35.2935	32.4399	48.5278	46.2941	21.3536	21.9316	2.89366	16.898
AAEL004698	DNA primase large subunit	26.81	22.8314	28.3849	32.3687	10.9063	11.4883	1.85338	13.313
AAEL011772	DNA repair protein rad50	17.8431	17.9945	25.3535	19.2822	11.9089	13.2917	2.92016	11.2966
AAEL006080	DNA repair protein rad51	16.9901	17.9583	29.6663	25.4541	13.9466	14.7315	5.04365	10.8492
AAEL003893	DNA repair protein xp-c / rad4	22.737	20.9348	55.4492	53.8683	11.7718	11.5541	2.39652	13.5368
AAEL002407	DNA repair protein xp-e	72.6099	69.5607	63.2931	62.5313	92.9633	88.2868	35.1522	46.4696

AAEL008519	DNA replication factor Cdt1	50.9481	41.8993	112.977	75.2323	8.99705	9.68246	5.07188	26.3101
AAEL000201	DNA replication helicase dna2	13.0996	11.6416	19.2081	17.9166	6.57764	6.49685	0.788585	7.0202
AAEL007007	DNA replication licensing factor MCM2	55.6228	48.5228	61.6279	90.8505	21.8831	20.2226	5.24031	28.4276
AAEL011811	DNA replication licensing factor MCM3	48.9815	45.0334	53.1483	67.2878	13.7061	13.2854	5.00647	25.0417
AAEL010086	DNA replication licensing factor MCM4	45.112	79.6423	50.1676	46.195	89.704	58.9794	95.836	100.315
AAEL014524	DNA replication licensing factor MCM4	6.37336	6.63384	13.0242	15.1626	2.8449	2.80821	1.13502	6.4552
AAEL002810	DNA replication licensing factor MCM5	107.237	61.231	80.6554	79.3061	27.5616	27.7872	7.47875	91.1537
AAEL012546	DNA replication licensing factor MCM6	44.7204	39.9632	53.7582	57.7663	10.5274	11.1543	5.81464	23.6017
AAEL000999	DNA replication licensing factor MCM7	90.6975	87.8161	108.503	127.264	44.185	42.1451	8.38969	51.7741
AAEL002551	DNA topoisomerase type I	110.251	111.801	88.7431	102.611	90.3311	87.61	54.562	74.2353
AAEL012584	DNA topoisomerase/gyrase	110.96	109.298	74.3439	89.6259	111.387	108.492	37.2816	61.329
AAEL009395	DNA-binding protein smubp-2	20.9502	21.9	31.2838	31.1572	23.7282	29.3873	0.462533	10.1129
AAEL009404	DNA-binding protein smubp-2	1.82122	2.04642	2.84302	2.43784	12.2219	11.5736	72.7104	24.595
AAEL009411	DNA-binding protein smubp-2	31.7432	31.3586	50.351	42.9779	17.225	18.299	0.598095	18.3674
AAEL001831	DNA-directed RNA polymerase II	44.7645	44.0061	56.8451	44.195	65.5211	63.665	37.6973	43.9374
AAEL011087	DNA-directed RNA polymerase II	40.3545	36.3654	54.0339	49.1154	33.5206	34.8193	24.8285	35.38
AAEL008755	DNA-directed RNA polymerase II subunit	29.4093	29.6433	37.8564	33.2875	19.8579	20.8702	7.51292	17.3808
AAEL007496	DNA-directed RNA polymerase III, 62-kS-<br>subunit, putative	22.6467	24.1543	27.3637	24.9984	28.2856	29.7953	4.94679	12.538
AAEL003552	DNA-directed RNA polymerase subunit rpb6	96.5175	100.916	130.079	93.5209	97.8051	104.427	42.9374	66.1955
AAEL000677	DNA-directed RNA polymerase subunit rpb8	27.7281	31.6712	42.1906	32.2519	33.5042	34.1814	10.8603	24.1531
AAEL013114	DNA-J, putative	39.7524	42.5553	35.3139	23.8252	17.8787	17.2099	20.4486	30.5807
AAEL003588	DNA-J/hsp40	57.3401	55.3102	45.9893	48.2527	24.0506	23.0387	48.4335	57.0581
AAEL010227	dolichol-phosphate mannosyltransferase	35.6808	61.4313	18.6134	21.8851	11.3135	12.9167	34.5848	38.4285
AAEL002174	dolichyl-diphosphooligosaccharide protein<br>glycosyltransferase	76.8373	98.3491	34.1252	32.1971	36.1687	37.6734	66.0716	70.0486
AAEL000064	dopachrome-conversion enzyme (DCE)<br>isoenzyme, putative	43.8635	41.263	74.753	48.8827	68.0042	54.5936	1.5093	25.3984
AAEL000024	dopachrome-conversion enzyme (DCE),<br>putative	19.9686	18.0133	11.0386	4.84592	29.1445	22.4467	1.38035	7.63285
AAEL008171	double-stranded RNA-binding protein zn72d	65.6139	69.9151	68.0228	65.9499	62.7135	64.3477	47.9759	59.3309
AAEL011118	dual specificity protein kinase	15.1473	13.4943	22.4209	20.1556	15.0338	14.312	4.99384	10.981
AAEL010411	dual specificity protein phosphatase	21.6646	19.2086	32.3897	33.5449	22.5374	17.8984	63.5125	30.0908
AAEL001504	Dual specificity tyrosine-<br>phosphorylationgulated kinase	7.80199	5.14066	5.49851	5.97232	6.74037	3.47415	96.8004	43.1967

AAEL004437	dual-specificity protein phosphatase, putative	25.8763	24.6221	35.6525	29.609	37.4987	37.0732	6.13305	13.1263
AAEL007563	DUOX	31.563	31.7145	31.6735	42.9531	27.7258	30.0873	0.461091	14.6599
AAEL007288	dynamin	30.0559	30.3754	27.0272	30.0009	28.3283	28.7239	97.7776	50.2362
AAEL000026	dynein light chain, putative	17.7011	20.998	36.7759	34.9074	14.9619	17.3062	4.58025	13.09
AAEL005343	dystrobrevin	22.5311	17.2939	31.6011	32.5071	15.1046	31.4166	29.4692	43.0437
AAEL000903	e(y)2	42.6628	50.2772	98.9911	55.1326	55.7427	51.7584	37.0068	33.3545
AAEL001440	E1a binding protein P400	60.8681	0	0	76.1761	47.9384	48.7943	0	36.6516
AAEL000327	E78	10.9617	9.83027	16.2937	14.9745	9.17335	8.87194	0.381277	3.67123
AAEL000293	ebna2 binding protein P100	153.633	181.981	76.923	85.7017	95.1723	91.1904	137.96	150.113
AAEL002130	ecdysone inducible protein L2, putative	37.523	31.9218	19.0782	40.0413	29.6297	25.3638	68.5442	28.1699
AAEL010719	ecdysone receptor A isoform, putative	12.0852	13.2227	16.3607	14.0347	18.1425	17.9967	2.33662	10.3573
AAEL010717	ecdysone receptor isoform-B (EcRB)	21.6805	16.141	22.8889	19.8393	20.35	17.8379	11.2824	17.2937
AAEL000741	ecdysone-induced protein 74EF (ETS transcription factor E74A)	8.67705	8.5406	4.71581	21.2539	4.13139	2.5504	15.5852	10.2064
AAEL009600	ECR	22.1848	23.2184	27.6837	24.1961	25.9487	25.1209	7.59585	15.4368
AAEL006851	eIF3g	39.488	60.4902	45.5963	33.802	77.8756	98.6759	53.6403	43.4689
AAEL002334	eIF3-S6	151.758	159.153	142.702	121.537	305.514	304.592	142.816	144.537
AAEL011954	elongase, putative	0	0	0.352695	26.1444	0	0	0.475727	0.560659
AAEL013649	elongase, putative	1.70347	1.20543	3.84339	30.9565	0.741684	0.480662	6.04831	3.8398
AAEL007216	elongase, putative	45.1316	24.5867	3.46268	15.0594	6.98504	6.99604	2.68097	17.4913
AAEL008004	elongase, putative	25.8781	27.1612	102.203	243.323	18.6559	16.9852	12.3751	18.8256
AAEL011951	elongase, putative	0.098208	0.231908	61.1803	665.854	0.087747	0.23198	0.201682	0
AAEL003977	elongase, putative	44.5622	45.175	5.65454	3.44746	12.113	10.1727	1.77119	24.7996
AAEL009574	elongase, putative	35.784	30.4455	21.3547	28.7781	31.8807	32.6641	66.9763	30.8232
AAEL013128	elongase, putative	84.164	82.7007	42.084	50.3979	75.244	74.07	31.5944	49.9028
AAEL009571	elongase, putative	54.6974	51.6662	144.109	285.598	36.1158	36.2072	14.0949	31.9343
AAEL012402	elongase, putative	3.12057	3.44041	3.05007	15.7016	5.46431	5.699	6.78794	3.87744
AAEL002662	elongase, putative	41.2335	33.3222	0.517018	2.09781	1.99332	1.22842	0.139038	18.0494
AAEL002673	elongase, putative	43.9746	36.0031	0.769478	0.737519	3.06473	3.93924	1.02092	26.693
AAEL004947	elongase, putative	13.3666	3.20066	4.52838	115.841	0.388071	0.328473	7.43067	6.71044
AAEL009313	elongation factor -1 beta,delta	220.649	243.664	290.361	205.971	469.873	469.563	124.244	171.642
AAEL011288	elongation factor 1 gamma	471.768	506.396	374.241	321.961	1344.45	1302.54	567.638	467.551
AAEL000951	elongation factor 1-beta2	431.78	459.597	262.835	219.219	983.286	1047.04	497.136	515.418
AAEL014536	embryonic ectoderm development protein	27.2995	25.3814	36.7812	33.4984	29.1614	31.3995	3.77691	16.0416

AAEL009189	encore protein	25.3855	24.6274	23.3034	25.3712	25.9842	27.4695	1.47692	14.4386
AAEL003906	endonuclease iii	7.99057	7.68862	14.216	12.9584	10.1902	10.3618	0.183985	5.59062
AAEL014052	endoplasmic reticulum protein erp29	31.4283	29.1623	1.9209	1.52131	4.04628	4.04446	5.24771	16.9836
AAEL011121	endoplasmic reticulumsident kdel protein	12.7122	11.371	13.7891	13.6924	10.5502	11.7947	1.5042	6.24328
AAEL009629	endoU protein, putative	15.7731	15.5615	33.7748	26.4637	113.911	117.583	1.93704	10.3882
AAEL012057	enhancer of polycomb	35.8528	35.1266	58.2539	52.7475	23.0345	23.0703	5.27799	19.6228
AAEL013213	enhancer of zeste, ezh	10.6308	8.89475	15.5293	15.5767	5.56176	4.80996	2.32929	9.30013
AAEL009846	Enn protein, putative	18.9279	19.6752	31.1065	31.0815	14.1244	13.9119	3.06407	11.538
AAEL001668	enolase	215.312	212.197	281.961	257.278	210.859	215.178	406.187	248.603
AAEL000109	enolase-phosphatase e-1	495.353	526.155	1026.85	659.959	640.932	600.054	102.943	307.065
AAEL006342	epoxide hydrolase	6.06724	11.3218	5.13887	3.01483	21.852	18.9628	277.378	140.301
AAEL006354	epoxide hydrolase	6.49537	8.1212	12.4393	11.7642	9.88621	7.69347	0.428002	4.51876
AAEL005926	er lumen protein retaining receptor	60.1107	61.1044	38.0246	44.9655	88.9803	77.2655	9.73756	32.6373
AAEL008434	er lumen protein retaining receptor	20.8442	14.9346	13.4414	10.6532	11.3634	13.9365	30.4939	9.01028
AAEL002086	er lumen protein retaining receptor	77.1062	86.379	62.02	59.4767	56.5122	49.8027	91.2933	92.2428
AAEL007023	estradiol 17 beta-dehydrogenase	86.4968	82.1669	48.1737	57.0183	66.6694	64.0072	659.552	321.568
AAEL013546	estrogenlated receptor (ERR)	24.5703	25.1079	33.7029	31.7232	25.5202	28.1926	5.71914	14.4466
AAEL005651	ethanolamine-phosphate cytidylyltransferase	188.065	169.959	91.0703	88.4162	106.388	80.9381	126.126	122.026
AAEL010488	ets	7.46265	6.44713	9.64068	28.6281	9.67279	8.25231	14.2809	8.71792
AAEL007945	eukaryotic translation initiation factor 3 subunit	140.839	139.857	115.197	93.1772	310.839	311.721	220.818	157.074
AAEL012279	eukaryotic translation initiation factor 3 subunit 1 (eIF-3 alpha)	114.211	130.035	154.742	126.464	61.5575	56.4334	165.839	125.873
AAEL009101	eukaryotic translation initiation factor 3f, eif3f	166.428	166.721	123.561	93.7901	332.254	335.05	188.545	165.565
AAEL001916	eukaryotic translation initiation factor 4e	223.101	230.361	289.061	257.692	208.761	193.55	135.588	148.542
AAEL001864	Eukaryotic translation initiation factor 4E binding protein (4EBP)	56.1386	54.7477	129.209	113.129	162.667	153.511	127.015	55.9004
AAEL005796	eukaryotic translation initiation factor 4e type	82.2134	78.4378	128.887	111.72	68.8749	75.4516	39.7768	62.2813
AAEL006209	exonuclease	14.5995	13.9807	23.0324	17.6726	11.9764	11.2341	1.97264	6.87598
AAEL005851	exosome complex exonuclease rrp42 (ribosomal RNA processing protein 42	30.3428	27.6976	21.4005	25.249	27.9933	32.5998	5.84092	13.4737

AAEL009588	expressed protein (HR3)	58.8275	55.0406	2.44735	1.98053	0.579328	0.528008	53.2582	56.9984
AAEL001302	expressed protein (HR39)	21.9393	36.7577	68.9343	92.3365	19.8452	24.707	16.4766	23.0311
AAEL007212	expressed protein (HR96)	9.46787	10.0765	21.3855	21.3237	19.3231	16.2465	90.6624	36.7399
AAEL002184	f-actin capping protein beta subunit	36.926	36.8904	40.7434	37.2927	22.9841	22.3273	161.851	80.7628
AAEL003453	failed axon connections protein, putative	35.7359	36.7426	35.221	28.0808	33.6216	35.6657	18.1624	27.4464
AAEL007042	far upstream (fuse) binding protein	131.968	144.15	96.1248	112.448	107.196	102.41	67.9652	85.8865
AAEL003497	farnesyl-pyrophosphate synthetase	75.3569	79.6183	61.9289	76.0556	70.1021	69.8976	567.733	288.709
AAEL003579	FAS-associated factor 1, putative	24.8908	25.7995	30.0486	32.6848	20.2207	20.2508	17.1506	27.6618
AAEL004287	fas-associated protein	72.789	49.722	39.7709	45.9789	27.0605	33.7415	55.0954	39.6178
AAEL013808	fascin	54.9018	48.381	78.615	78.3361	27.2109	26.5089	3.97146	24.3162
AAEL004452	fast myosin heavy chain HCIII, putative	81.1228	86.1516	106.489	101.213	60.4399	66.8601	46.9715	57.4218
AAEL003203	fatty acid desaturase, putative	275.851	266.189	104.084	88.077	557.811	564.872	950.503	604.461
AAEL003831	fatty acid hydroxylase	0.380064	0.622139	0.769967	0.98622	4.14997	1.57248	427.183	162.032
AAEL001194	fatty acid synthase	148.921	123.799	110.689	132.013	231.6	243.598	78.0912	106.869
AAEL002228	fatty acid synthase	12.728	14.388	13.9416	13.2079	44.4437	38.5053	0.077849	4.21461
AAEL002227	fatty acid synthase	8.7376	8.31912	7.68674	7.82162	15.261	13.5556	0.125588	3.43601
AAEL003371	f-box and wd-40 domain protein	61.6336	58.65	95.718	80.9782	60.27	58.4684	27.6734	39.5451
AAEL011215	f-box and wd40 domain protein 7 (fbw7)	25.0293	24.8524	51.9059	53.3198	22.1662	24.0517	6.83589	14.9378
AAEL004875	f-box only protein	38.5058	38.2009	51.3939	49.0694	41.0941	38.117	13.0372	22.0594
AAEL007385	ferritin subunit 1, putative	426.873	438.235	653.399	586.325	586.517	550.719	16710.7	7091.37
AAEL010248	fibrillarin	56.1673	61.7061	44.9895	45.1794	50.7601	46.9025	7.19396	27.5032
AAEL000549	fibulin 1 and	0.191711	0.058313	0.331378	0.21373	0.635152	1.2164	145.32	60.9282
AAEL002539	fimbrin/plastin	13.0042	11.9885	10.6205	13.651	15.694	14.3958	324.643	126.295
AAEL011632	fk506 binding protein	28.9957	28.8303	62.4457	46.7805	35.0439	35.8262	7.81378	19.2977
AAEL004313	fk506-binding protein	152.224	224.999	3.82671	2.74099	117.456	110.144	150.767	176.767
AAEL008367	fk506-binding protein	42.7574	67.5628	17.2781	15.0457	24.3144	26.1062	37.2555	43.1144
AAEL007883	fk506-binding protein	170.508	197.203	199.859	174.543	128.204	125.327	179.33	174.587
AAEL008958	forkhead box protein (AaegFOXK1)	74.831	77.0082	104.486	96.5747	79.5901	79.9766	36.8177	57.4008
AAEL005731	forkhead box protein (AaegFOXL)	63.6585	52.4966	30.1331	31.1859	65.9127	61.5057	0.38283	27.183
AAEL009900	forkhead box protein (AaegFOXN2)	36.479	39.6099	52.0514	47.3065	42.4063	43.5304	1.79518	19.4693
AAEL014953	forkhead box protein (AaegFOXO)	61.3426	36.7483	68.249	61.3273	82.1431	63.5634	22.3293	52.6943
AAEL003162	formin 1,2/cappuccino	34.9295	32.3102	58.5297	51.0837	30.2772	29.5725	5.69638	21.0016
AAEL001946	four and a half lim domains	33.8771	32.6314	58.9568	35.8233	22.484	23.5853	51.6911	38.8111
AAEL009326	fragile X mental retardation syndromelated protein 1, putative	37.4508	36.794	36.6728	34.8979	25.4551	25.2305	15.7753	22.2764

AAEL008646	FREP10	10.5715	13.5298	8.82286	15.7202	18.0602	33.1618	23.3313	15.1322
AAEL007942	FREP14	0.138982	0	0.103623	0.476286	0.829964	0.971273	292.798	159.535
AAEL006704	FREP18	1.6172	0.068677	3.28838	5.30819	18.6785	31.7527	3143.42	1286.33
AAEL006691	FREP21	0	0	0.112994	0.173948	0.627629	0.824897	197.46	111.805
AAEL003294	FREP3	26.3632	24.7298	1.14684	1.6412	2.12542	2.4862	2.46422	12.2444
AAEL006699	FREP34	0.095404	0.1126	0.184732	0.255671	3.94625	1.60969	169.058	84.7983
AAEL011400	FREP36	19.5417	22.7783	13.7396	12.5416	35.9064	32.2341	0.154572	6.04485
AAEL011007	FREP37	0.377	0.31723	0.517572	0.55057	1.46562	1.96242	73.8202	34.3819
AAEL004156	FREP9	1.87661	1.94737	2.45431	3.85839	17.8931	13.4776	606.46	252.965
AAEL001158	fructose-1,6-bisphosphatase	94.3256	101.668	152.194	136.487	85.0668	85.913	16.0319	63.4679
AAEL005766	fructose-bisphosphate aldolase	361.398	355.971	852.5	657.591	218.441	224.21	453.717	376.351
AAEL006301	fruitless	28.4887	25.9774	38.8907	40.1611	43.4321	42.2452	10.163	16.9456
AAEL007889	f-spondin	17.435	23.6443	9.88091	8.47619	23.9324	23.2587	3.09162	12.0441
AAEL003413	f-spondin	40.2696	43.9648	13.5804	8.62217	59.8893	60.7908	1.62881	13.9819
AAEL011973	fumarylacetoacetate hydrolase	46.201	44.0554	53.3359	67.8606	35.416	40.1345	5.533	35.8919
AAEL004722	GABA-A receptor interacting factor-1, putative	42.5006	38.3412	44.3482	45.9543	40.4683	41.3333	127.716	65.1162
AAEL002781	galactokinase	22.5817	24.2607	22.6174	25.8049	37.2711	35.1161	182.319	87.8555
AAEL001607	galactose-1-phosphate uridylyltransferase	125.518	163.889	17.6239	15.0406	27.8816	25.2301	114.903	126.644
AAEL009842	GALE12	5.48747	6.07857	6.88209	14.0179	46.7361	12.2741	459.574	223.576
AAEL012135	GALE2	0.136293	0.079623	0.356722	0.311955	2.91866	1.86576	225.778	76.6082
AAEL003844	GALE5	28.3501	22.8863	35.2588	38.9559	147.17	120.563	641.786	210.874
AAEL005293	GALE8A	0.03921	0.047552	0.136661	0.197208	1.23489	2.08764	423.935	161.78
AAEL004522	GAM1	1.09613	0.048904	3.4255	2.89649	2.97939	29.1755	1085.42	696.837
AAEL015561	gamma glutamyl transpeptidases	2.0879	2.17959	8.20674	1.40396	0.887283	1.58647	3.00432	3.90681
AAEL014580	gamma glutamyl transpeptidases	14.5215	14.1601	20.375	5.38836	13.0255	14.6821	4.12007	9.58114
AAEL000271	gamma-glutamyl hydrolase, putative	21.5812	18.3133	12.7306	12.7661	41.1694	32.27	417.69	185.654
AAEL000759	gamma-glutamylcysteine synthetase, putative	30.9016	33.0472	29.093	24.5402	24.6109	23.1815	140.083	55.8172
AAEL012939	gamma-subunit,methylmalonyl-CoA decarboxylase, putative	45.0181	48.3994	70.7594	59.6578	39.5985	37.9131	138.417	56.2989
AAEL013903	gamma-tubulin complex component 2 (gcp-2)	14.3229	14.2554	15.0699	15.1469	15.3908	18.0827	3.02859	7.91531
AAEL013956	ganglioside induced differentiation associated protein	20.1691	7.74971	14.4487	10.5351	15.6242	19.6884	41.6739	19.6326
AAEL010218	GATA transcription factor (GATAa)	8.65539	10.0417	5.58819	8.29152	18.2321	12.2673	0	5.38319

AAEL006447	GATA transcription factor (GATAb)	13.9941	13.8034	15.7695	16.9167	19.3759	18.3476	1.39659	6.46368
AAEL010225	GATA transcription factor (GATAc)	0.75897	0	0	0	2.77775	0	128.448	35.9889
AAEL005947	Gch sequence factor-1, putative	117.773	131.761	98.8619	94.4672	73.2074	71.8661	64.9139	85.1101
AAEL006102	Gelsolin precursor	20.6938	19.684	18.6285	17.6314	72.7869	83.7732	2.21769	9.70993
AAEL006096	Gelsolin precursor	34.4736	41.2539	39.9548	37.2645	159.417	147.678	7.37279	19.8067
AAEL009773	geminin, putative	46.6115	44.9952	85.3845	58.7527	35.7267	33.5739	2.80691	21.8801
AAEL007474	geminin, putative	16.3438	15.5843	24.6254	17.2688	13.4942	12.7188	1.07619	7.30675
AAEL005925	geranylgeranyl pyrophosphate synthase	0	0	9.83949	47.5108	0.079781	0	0.149878	0
AAEL000856	germ cell-less protein	80.1847	96.2094	69.6913	142.495	145.598	149.959	21.4109	106.979
AAEL009055	gliotactin	2.63495	2.36214	2.11197	1.94937	1.11941	1.10889	50.0992	19.2493
AAEL013877	glucosamine-6-phosphate isomerase	82.3127	72.8013	140.168	122.101	30.6761	34.1517	413.624	231.166
AAEL009510	glucosamine-fructose-6-phosphate aminotransferase	8.35608	13.7614	9.24799	38.2702	7.58236	7.7845	355.495	149.422
AAEL005705	glucosaminephosphotransferase	29.0363	51.8802	12.6474	11.3944	13.8749	14.5026	22.8709	33.8174
AAEL004025	glucose dehydrogenase	34.2184	26.2606	28.4214	29.2234	32.5042	31.2791	216.714	98.8748
AAEL011809	glucose dehydrogenase	0.212167	0.150492	43.8469	17.6171	0.717809	0.39958	0.206984	0.247033
AAEL004028	glucose dehydrogenase	9.56958	6.93327	1.30409	3.61829	1.22862	1.00577	0.05962	3.21574
AAEL005769	glucose dehydrogenase	14.1875	19.107	5.62609	8.90387	26.8519	25.3995	2.89103	8.482
AAEL009207	glucose dehydrogenase	0	0	18.4634	85.0024	0	0.028049	0.063828	0
AAEL011808	glucose dehydrogenase	0	0	3.03456	28.6953	0	0	0	0
AAEL002292	glucose transport protein, putative	0.078359	0.368688	0.282226	0.291761	7.89886	1.03624	156.528	61.5323
AAEL006274	glucose transporter (sugar transporter	0.991036	0.745517	0.729482	1.08707	1.46924	1.48468	168.357	88.9837
AAEL001548	glucosyl/glucuronosyl transferases	6.78454	7.20274	4.18482	2.68176	6.74077	7.50307	263.623	111.231
AAEL014246	glucosyl/glucuronosyl transferases	47.6222	64.8569	154.077	111.765	29.6603	32.5514	19.011	21.1695
AAEL003076	glucosyl/glucuronosyl transferases	23.1568	28.7447	105.466	93.6971	11.6764	16.7555	261.364	130.819
AAEL001586	glucosyl/glucuronosyl transferases	5.91158	5.88704	30.4033	6.0931	8.71318	8.68912	80.3081	34.6562
AAEL002688	glucosyl/glucuronosyl transferases	6.14036	5.69174	9.50932	10.8027	4.21222	5.34769	87.1275	43.8776
AAEL000687	glucosyl/glucuronosyl transferases	1.08456	0.67963	0.305904	0.287151	2.59256	2.16078	125.333	56.7746
AAEL001816	glucosyl/glucuronosyl transferases	0.442943	0.130473	0.918842	0.899373	0.494131	0.925049	317.724	104.993
AAEL010464	glutamate dehydrogenase	79.6919	88.6478	63.9794	42.1885	99.1144	101.513	240.243	185.836
AAEL006834	glutamate semialdehyde dehydrogenase	289.285	348.112	150.499	303.976	190.009	186.636	337.485	351.009
AAEL001887	glutamine synthetase 1, 2 (glutamate-amonia ligase) (gs)	137.731	182.676	88.5924	122.008	94.8493	98.3895	2011.62	1076.99
AAEL013458	glutamine synthetase 1, 2 (glutamate-amonia ligase) (gs)	155.981	153.791	414.587	350.243	112.743	118.868	56.4536	99.0772

AAEL007201	glutamyl aminopeptidase	38.7937	39.193	47.4323	48.2631	42.8595	46.0986	412.823	223.014
AAEL012238	glutaredoxin, putative	60.3369	67.9763	72.2808	72.368	46.5401	49.958	1492.31	757.516
AAEL013980	glutaredoxin, putative	98.4046	102.239	129.432	100.594	80.8615	91.5295	355.897	252.037
AAEL014064	glutaredoxin, putative	38.3573	43.7298	40.8078	38.3568	29.4323	29.2416	307.818	142.655
AAEL009154	glutathione synthetase	24.0707	22.9615	26.6313	25.2463	20.4442	19.4	147.103	78.092
AAEL006239	glycerol kinase	132.36	119.098	207.959	223.656	35.4534	32.1714	11.4716	66.7951
AAEL007080	glycerol-3-phosphate acyltransferase	23.5699	23.3175	29.1919	34.8646	38.1631	40.1902	228.557	118.932
AAEL001593	glycerol-3-phosphate dehydrogenase	228.735	201.293	515.174	509.007	91.984	91.2918	320.326	306.696
AAEL012974	glycine cleavage system h protein	107.855	144.172	26.729	13.2081	96.8739	83.3312	6.58193	54.9516
AAEL000364	glycine cleavage system h protein	38.7223	67.1238	8.41185	7.10799	38.9383	31.6574	2.61035	23.0269
AAEL014426	glycine dehydrogenase	17.8362	43.2283	6.64839	0.830468	18.3785	13.7491	0.527208	15.9674
AAEL015143	glycine rich RNA binding protein, putative	203.053	208.58	196.991	225.974	154.925	161.467	34.8021	96.2012
AAEL000703	glycogen phosphorylase	227.753	203.634	267.088	355.012	53.2051	55.2973	35.2261	113.91
AAEL004221	glycogen synthase	76.9189	74.3687	103.251	101.24	53.5967	55.5335	23.2286	50.6323
AAEL014863	glycogenin	100.206	93.4828	110.936	148.992	71.4718	76.0995	19.7685	57.2469
AAEL003094	glycoprotein, putative	0	0.039447	0.567512	22.9632	0	0	0.153677	0
AAEL000559	glycosyl transferase	28.4276	40.8901	11.2672	13.9379	19.882	20.6427	18.7375	25.1062
AAEL007982	glycosyltransferase	11.693	9.37167	10.3779	9.29142	4.89257	4.37287	2.39565	7.03015
AAEL000004	glycosyltransferase	38.4754	41.3158	23.2973	20.0629	28.003	28.4261	15.9203	28.939
AAEL004532	glyoxylate/hydroxypyruvate reductase	31.1993	26.4969	97.5608	88.9421	19.8914	23.1363	183.567	100.028
AAEL003889	GNBPB1	8.81469	6.01901	4.11017	3.1353	55.8212	28.8139	4.10992	2.9058
AAEL009176	GNBPB3	0.056207	0	0.124017	0	0.049874	0	128.26	55.5693
AAEL009178	GNBPB4	0.881194	1.19836	7.6961	4.94994	3.15691	3.80633	1285.41	526.139
AAEL006230	gonadotropin inducible transcription factor	9.42713	7.97695	12.2949	13.2881	11.7393	9.8715	2.86979	8.30032
AAEL002145	gonadotropin inducible transcription factor	39.4393	37.8799	59.2359	56.4681	25.9155	28.5576	8.85571	17.2846
AAEL011325	gonadotropinleasing hormone receptor	29.1676	31.9452	14.1097	17.1622	30.7695	28.0331	0.57311	11.8488
AAEL003494	goodpasture antigen-binding protein	89.2534	92.3968	146.609	130.755	82.5748	83.613	38.989	52.7984
AAEL008126	GPRCIR	16.0812	14.6844	10.5344	12.3941	10.6121	10.0784	0.623165	7.6707
AAEL013160	GPRFZ1	16.9578	15.7754	22.8932	22.2939	13.625	14.6032	2.2061	9.58782
AAEL018033	GPRFZ5	11.2914	11.0867	9.4145	12.0179	7.18899	6.7159	0.775503	4.63628
AAEL015190	GPRFZ6	7.56664	10.3371	6.11089	8.93485	4.74792	4.03612	0.327427	4.54901
AAEL009983	GPRFZ7	17.0497	16.4991	27.3938	26.9304	15.6116	15.9642	1.85734	10.0068
AAEL013886	GPRFZ9	14.1968	18.0483	12.6455	18.8418	9.24516	11.2033	1.07938	8.54769

AAEL012796	GPRGHP3	9.61758	6.8837	16.2703	14.0765	3.57566	3.70296	0.096446	8.75383
AAEL010980	GPRMTH2	3.4506	3.02949	1.90271	2.06286	2.62548	1.76406	46.6332	16.9938
AAEL011521	GPRMTH6	15.6051	15.672	25.0898	20.4646	15.4124	13.2828	132.558	55.1099
AAEL000811	GPRMTH7	0.979932	1.65914	4.37361	7.158	2.93099	1.53187	78.8696	27.1764
AAEL017160	GPRMTH9	19.4485	20.0156	23.016	16.8358	14.2971	15.9842	106.147	48.0732
AAEL001606	GPRMTN	48.6319	46.7343	92.3892	81.7239	55.1819	52.6803	189.805	77.371
AAEL003448	GPRNNA13	0	0.09666	0.673938	1.28568	0.222948	0.160099	165.089	30.809
AAEL017549	GPRNNA14	0	0	1.49154	1.17642	0.329549	0.360702	167.987	31.269
AAEL007644	GPRNNA6	0.078036	0.030105	0.112429	0.084526	0.902369	0.334322	38.2004	12.871
AAEL003647	GPRNPR3	34.5109	32.4863	44.8309	43.0427	32.6688	33.6499	0.425861	16.1367
AAEL004396	GPROAR4	23.7731	20.667	14.2494	18.8781	20.0576	19.8273	0.40805	4.89764
AAEL006685	G-protein, gamma-subunit, putative	3.00396	7.70858	3.52017	1.07255	6.00563	4.73388	452.077	161.878
AAEL000246	GPRSTN	10.6888	11.3003	10.5306	9.87511	7.23146	7.93986	1.40429	6.49815
AAEL017544	GPRTAK1_3	0	0	0	5.36269	6.17318	0	0	0
AAEL012069	GPXH1	25.3448	28.7313	30.8929	31.4973	30.8872	22.3454	355.3	139.03
AAEL011571	Gr14	13.2669	11.7753	22.0871	16.9473	13.2353	14.3795	2.75842	7.74628
AAEL014285	growth hormone inducible transmembrane protein	47.2857	50.1337	62.2759	57.1961	53.9836	56.7029	130.731	66.6862
AAEL001061	GSTD1	897.43	772.011	1261.86	1332.29	338.924	372.961	130.369	411.846
AAEL010582	GSTD11	41.1254	38.1676	66.8557	67.7809	33.8708	34.6026	334.759	133.787
AAEL001054	GSTD4	0	0	0.071089	0	0	0	20.3691	6.21051
AAEL011752	GSTI1	24.1549	24.0124	25.4927	24.7266	31.0509	29.9601	25.212	33.4896
AAEL011741	GSTS1	6.67742	5.71894	14.0009	20.2659	15.3922	12.6177	1993.64	811.029
AAEL004229	GSTT4	12.1401	9.38805	5.71488	2.42175	24.0845	24.8458	259.511	102.005
AAEL000092	GSTX1	0.091411	0.314789	0.194914	0.224011	1.15751	0.63528	200.148	80.9878
AAEL002261	GTP cyclohydrolase i	107.745	123.706	153.748	153.901	70.3653	73.6661	3.31083	43.0055
AAEL006786	gtpase_rho	39.5036	39.8174	31.6276	33.5629	53.5136	53.3535	153.464	73.4124
AAEL013139	gtpase_rho	158.083	166.108	217.074	201.167	93.7775	97.9374	569.421	351.26
AAEL010506	GTP-binding protein alpha subunit, gna	51.8354	49.3861	56.7759	61.6767	33.2132	34.0133	27.867	36.3021
AAEL013123	GTP-binding protein alpha subunit, gna	0.148177	0.306317	0.207615	0.873003	11.4283	0.73013	502.312	159.98
AAEL008630	GTP-binding protein alpha subunit, gna	14.8817	16.2805	11.1397	14.2599	15.1081	15.0266	2.04737	9.08876
AAEL002160	GTP-binding protein	95.5662	98.9436	58.0927	56.1127	199.162	194.042	87.7416	93.3108
AAEL005634	harmonin, putative	12.5303	12.9142	12.5595	20.0816	9.84025	10.4248	0.219818	4.94449
AAEL014845	heat shock protein	105.715	106.251	138.75	133.543	141.772	138.087	38.9835	59.9355
AAEL011704	heat shock protein	23.6436	35.0353	34.8977	34.789	13.4525	17.6855	8.75952	28.0101

AAEL011708	heat shock protein	11.4459	18.3017	23.0814	20.3761	14.659	17.2465	6.29375	22.1229
AAEL014843	heat shock protein	161.368	142.846	296.657	205.745	85.7387	88.5654	80.5943	112.68
AAEL013350	heat shock protein 26kD, putative	0.417291	0.366119	0.383113	0.795889	2.53605	0.849699	33.7163	6.96623
AAEL004148	heat shock protein 70 (hsp70)-interacting protein	141.332	139.383	157.132	133.676	89.8208	84.8201	217.693	139.373
AAEL001052	heat shock protein, putative	112.753	132.78	153.572	137.356	128.101	122.287	66.0433	96.6186
AAEL007558	helicase	41.796	42.4683	41.2621	45.0229	50.9923	48.9226	20.4248	28.6352
AAEL003968	helicase	30.1413	27.8515	64.8474	55.0784	21.0452	20.4847	14.1456	21.903
AAEL006331	helicase	19.0331	17.3495	27.7898	26.6407	14.7972	13.4038	3.02399	8.35923
AAEL003950	helicase	30.1893	28.3105	97.5146	70.0614	21.4036	21.2755	0.684534	12.6438
AAEL010472	helix-loop-helix protein hen	5.83821	8.56157	10.7502	7.75201	7.09626	8.11078	0.076142	3.03418
AAEL008410	helix-loop-helix transcription factor	0.996237	0.617727	1.11798	0.818492	1.75704	1.88158	44.9634	16.4718
AAEL004868	hemomucin	5.4682	5.71403	4.79667	5.75586	8.51465	8.2994	23.7504	7.62198
AAEL010437	heparan n-sulfatase	27.6013	27.5453	46.1966	50.0781	22.8586	23.102	3.57054	18.2227
AAEL013538	hepatoma-derived GF, putative	81.3503	83.4447	93.5108	106.502	63.525	60.6624	25.6511	55.2574
AAEL005049	heterogeneous nuclear ribonucleoprotein	40.2482	41.8015	65.5364	55.509	61.1297	57.3564	0.493372	18.6652
AAEL005515	heterogeneous nuclear ribonucleoprotein	840.689	872.542	874.706	816.659	764.281	757.764	387.887	565.257
AAEL010467	heterogeneous nuclear ribonucleoprotein	155.813	166.295	63.2905	110.344	91.2327	95.0781	125.431	130.424
AAEL013990	hexamerin 2 beta	20.5019	16.3714	8.29182	8.74707	23.0857	21.0442	1.35914	6.62855
AAEL009387	hexokinase	94.0847	88.5151	99.784	127.029	119.066	108.398	465.102	219.13
AAEL013263	high affinity copper transporter, putative	0	0	0.118613	0.068873	0.375197	0.40772	259.973	126.439
AAEL001421	high density lipoprotien binding protein / vigilin	134.422	158.945	39.8115	40.0229	100.395	98.1461	119.492	122.626
AAEL011380	high mobility group B1, putative	114.793	112.141	45.4829	61.4081	103.348	93.0663	27.6419	52.394
AAEL011414	high mobility group non-histone protein, putative	712.419	680.358	1355.14	1029.93	437.543	453.825	312.521	487.82
AAEL009630	high-affinity cgmp-specific 3,5-cyclic phosphodiesterase	3.0317	2.46298	5.99876	6.71865	22.5756	19.2645	33.2083	11.8855
AAEL006177	histone acetyltransferase type b catalytic subunit	92.3097	85.8616	152.501	160.343	61.1474	65.1888	16.6577	52.3923
AAEL004586	histone deacetylase	120.138	114.27	212.317	175.983	105.927	100.841	128.116	95.1292
AAEL010638	histone H1, putative	37.9809	32.0816	136.247	113.352	15.2525	15.2301	0.448513	20.5065
AAEL002103	histone H1, putative	69.3417	73.0305	63.8021	67.0079	81.5138	78.5006	0.746958	36.135
AAEL007882	histone H1, putative	20.0886	20.5394	19.4256	18.6658	31.5285	31.8783	6.14773	12.1622
AAEL007609	histone h2a	698.976	688.322	851.177	816.732	411.188	405.701	132.756	405.487

AAEL003862	histone h2a	44.1695	44.0198	144.697	115.606	12.0547	17.6756	3.49712	21.3888
AAEL012499	histone h2a	207.897	168.855	857.674	746.175	49.959	56.0888	8.43925	104.726
AAEL003669	histone h2a	19.2038	10.0337	109.029	87.8368	10.9527	10.2862	0.560132	7.82025
AAEL015683	histone H2B	4.49822	4.83594	22.9975	12.6351	3.50827	2.49026	0	3.19773
AAEL015684	histone H2B	16.4742	11.7226	71.9551	55.706	9.75904	9.30525	0.199043	6.06962
AAEL015682	histone H2B	60.3395	41.9439	350.174	257.98	66.5495	54.0005	0.518904	28.4604
AAEL003685	histone H3	36.544	25.7887	107.954	93.9566	43.5937	38.1034	0	17.8676
AAEL003659	histone H3	131.983	98.9819	387.349	301.844	135.301	115.559	0.513265	58.868
AAEL011424	histone H3	310.883	278.031	609.18	578.034	144.873	136.068	9.21884	202.912
AAEL006783	histone h3 methyltransferase	31.2776	34.6021	52.856	43.9186	32.1132	35.2875	7.36357	18.1374
AAEL003673	histone H4	16.6874	13.2486	97.8446	78.018	19.2062	17.3831	0	7.71015
AAEL003689	histone H4	45.825	41.6225	183.515	158.772	77.7703	70.2513	1.55468	29.8723
AAEL013709	histone H4	2.82122	0	1.93907	0	1.49983	0.655327	6.49625	0
AAEL001764	histone-fold protein CHRAC subunit, putative	48.7546	45.392	56.9967	48.3663	30.1083	30.128	17.905	36.8493
AAEL000193	histone-lysine n-methyltransferase	74.3434	74.9104	131.31	114.317	57.7839	63.0851	11.6314	41.0142
AAEL002740	homeobox protein otx	13.3456	15.0058	12.4565	12.737	13.6941	16.396	0.137018	4.24862
AAEL002769	homeobox protein prospero/prox-1	14.9445	14.0447	13.8258	15.6116	13.1207	14.9475	0.896982	7.66991
AAEL009949	homeotic antennapedia protein, putative	19.0548	21.0334	28.8075	20.3452	11.6969	10.1527	0.882381	9.54075
AAEL013637	homogentisate 1,2-dioxygenase	32.8327	48.6646	6.71054	5.22653	15.2216	12.2206	1.28783	14.3372
AAEL004401	HPX7	219.367	206.154	17.6291	43.2112	94.305	97.9846	1.95119	114.061
AAEL004388	HPX8A	157.632	154.771	225.725	181.715	84.8077	84.3879	1.76198	89.3823
AAEL004390	HPX8B	743.736	609.752	230.614	451.652	396.572	390.362	7.70715	342.482
AAEL004386	HPX8C	385.531	341.083	155.745	259.284	317.875	318.677	3.91822	182.362
AAEL014598	huntingtin interacting protein	4.27348	3.64577	4.58315	5.07729	5.26852	5.13868	71.5841	29.4527
AAEL010449	huntingtin interacting protein	1.73682	1.58103	1.83827	1.94478	2.08815	1.8942	32.1952	12.4012
AAEL006509	hydrogen-transporting ATP synthase, G-subunit, putative	173.921	212.913	207.031	179.322	293.54	299.905	452.811	373.168
AAEL008835	hypothetical protein	14.0972	14.2645	15.9713	17.9494	23.8281	23.4698	0.40416	6.9524
AAEL008511	hypothetical protein	12.3372	14.9924	16.3221	15.6992	6.21462	6.74209	0.13999	8.90984
AAEL012637	hypothetical protein	18.9183	16.6524	26.0701	25.2479	19.2775	18.4295	4.067	11.6958
AAEL012815	hypothetical protein	108.075	105.577	128.844	142.243	82.6691	87.4366	23.6202	71.4923
AAEL009208	hypothetical protein	15.0474	13.7139	34.9891	21.656	14.0957	13.6572	18.0531	15.1506
AAEL013545	hypothetical protein	2.26476	5.23918	2.98714	3.4675	7.8382	9.21599	4.68536	12.0096
AAEL014877	hypothetical protein	0	0	85.2193	339.495	0	0	0.393603	0

AAEL003190	hypothetical protein	0.682168	0.39769	0.128944	0.595462	3.02207	2.29595	314.378	117.309
AAEL007012	hypothetical protein	15.0541	10.2136	30.3096	8.73956	5.03291	0.420931	0.96054	0.281692
AAEL013616	hypothetical protein	22.2196	22.8765	32.8944	26.8807	22.6921	22.2644	8.1035	18.4348
AAEL014522	hypothetical protein	25.3385	22.6203	31.8504	28.0864	22.3908	22.2497	5.45224	14.1816
AAEL011266	hypothetical protein	34.961	35.9123	49.5359	91.0167	69.1234	69.8012	21.9014	27.2218
AAEL005213	hypothetical protein	199.271	234.218	230.401	307.708	333.97	346.755	385.384	292.079
AAEL013916	hypothetical protein	8.84072	10.923	16.1078	15.012	8.50115	10.0572	0.155352	5.83789
AAEL001958	hypothetical protein	17.5674	19.3261	31.7943	28.9595	21.3123	18.6275	0.213737	9.82469
AAEL012050	hypothetical protein	2.45313	6.05632	4.66672	3.08672	4.26002	4.88602	0	6.04148
AAEL012398	hypothetical protein	0.736314	0.282133	0.190921	0.158592	0.830446	1.03886	161.222	64.59
AAEL002297	hypothetical protein	120.608	24.1057	56.3871	54.0072	60.5523	19.2767	20.5345	79.0977
AAEL007879	hypothetical protein	10.8265	12.6159	0.402052	1.84044	9.29075	8.85887	0.206568	6.97257
AAEL009890	hypothetical protein	18.1588	17.5672	19.4798	20.7121	17.9113	18.437	7.5596	14.545
AAEL007086	hypothetical protein	19.7269	16.306	15.0606	20.945	26.5716	18.9136	1.32107	8.82575
AAEL008101	hypothetical protein	6.66713	7.54015	12.0887	11.7601	5.13925	5.11503	1.47003	6.79952
AAEL008927	hypothetical protein	102.365	106.037	149.244	135.41	86.0365	77.5352	20.0632	56.8016
AAEL012119	hypothetical protein	26.4585	30.2907	39.7281	18.2393	39.3199	33.4011	17.495	21.6265
AAEL015044	hypothetical protein	10.5505	9.87511	12.5344	12.5655	11.6716	10.1674	1.76276	6.90313
AAEL004322	hypothetical protein	53.52	51.1508	24.2378	34.283	53.4978	55.15	13.2367	34.1069
AAEL009687	hypothetical protein	19.8403	37.7746	37.6543	44.2582	15.7977	19.7766	27.3587	41.0187
AAEL000005	hypothetical protein	27.7279	26.0935	34.4804	27.5257	30.8947	30.811	7.92174	17.4151
AAEL004384	hypothetical protein	12.9321	16.2233	16.5901	9.94771	15.8936	14.5098	252.067	121.883
AAEL008772	hypothetical protein	29.5566	28.9745	22.3297	27.1279	32.0641	34.5874	0.669487	19.9158
AAEL010705	hypothetical protein	0.655374	0.834383	948.379	2602.97	0.619247	0.564621	1.20899	1.22964
AAEL007417	hypothetical protein	30.9734	30.4854	43.4469	42.6945	22.2512	25.1763	16.724	24.0457
AAEL000861	hypothetical protein	0.763436	0.647683	0.647108	0.981756	1.62978	1.91502	217.399	98.6986
AAEL014991	hypothetical protein	25.1227	28.0235	23.8305	23.2745	20.5478	19.78	8.63146	16.3665
AAEL013659	hypothetical protein	114.906	103.322	148.904	141.851	46.9169	50.9608	0.925466	48.8728
AAEL002252	hypothetical protein	34.6037	24.3325	48.2975	32.3444	27.3365	16.4441	29.1547	22.708
AAEL011350	hypothetical protein	1.83902	1.17297	20.6476	5.39568	3.93381	4.64815	1.50074	1.49971
AAEL005916	hypothetical protein	40.1389	37.1668	20.1436	30.5137	24.6374	26.5837	0.335663	18.7889
AAEL013133	hypothetical protein	57.7439	55.9739	110.597	86.1328	67.1032	66.2643	150.878	84.51
AAEL005873	hypothetical protein	30.0849	32.9362	40.1602	41.8966	48.3419	47.1238	8.13828	25.1231
AAEL008027	hypothetical protein	39.2748	40.6805	41.8897	44.8201	19.4421	20.8003	16.8458	28.9694
AAEL000058	hypothetical protein	0.058168	0.099985	0.085544	17.2086	0.104847	0.057241	0.067594	0.150377

AAEL004894	hypothetical protein	0.074645	0.175527	82.7249	152.572	0.067984	0	0	0.13254
AAEL007841	hypothetical protein	40.996	40.3483	76.3747	69.3183	31.4009	30.1311	11.8552	26.1762
AAEL000702	hypothetical protein	30.8076	27.6538	29.8711	26.521	39.0296	42.9046	86.0969	38.2362
AAEL004591	hypothetical protein	49.292	82.1277	27.8244	28.0834	76.9878	98.2405	2.73713	19.439
AAEL014041	hypothetical protein	10.1582	9.51923	11.364	9.06598	12.9725	13.1788	0.468033	4.78262
AAEL010056	hypothetical protein	142.809	196.838	104.157	95.4382	83.2076	107.462	272.035	201.028
AAEL012858	hypothetical protein	119.667	116.716	8.66927	8.2576	73.6123	61.6964	3.48958	56.6768
AAEL013799	hypothetical protein	22.8461	24.0782	54.7326	42.414	12.2462	13.1993	0.478581	13.5095
AAEL010978	hypothetical protein	22.7563	22.4635	18.7164	17.1534	22.2777	23.0748	5.11312	14.111
AAEL008137	hypothetical protein	66.8723	59.076	153.533	151.696	16.4066	16.2483	1.51096	28.4417
AAEL003072	hypothetical protein	9.74593	4.47175	20.1549	22.9769	4.64705	3.89425	0.265139	8.60239
AAEL014047	hypothetical protein	5.97663	6.38203	6.17222	4.64292	7.31823	4.40257	0.915248	5.19872
AAEL008710	hypothetical protein	0.998944	1.02573	1.35278	2.58498	9.73919	4.8444	256.312	79.6645
AAEL000002	hypothetical protein	24.8478	25.9687	39.9746	81.1533	47.0689	23.0583	2.1628	12.1239
AAEL000172	hypothetical protein	54.5744	52.296	71.9907	58.8273	53.7561	54.5815	22.3532	40.797
AAEL000257	hypothetical protein	31.5691	31.3164	37.4232	34.2648	33.93	32.4408	8.5488	16.784
AAEL000657	hypothetical protein	70.2718	85.0113	165.269	198.906	78.1373	76.7406	10.5479	43.2371
AAEL000723	hypothetical protein	2.64334	4.4541	3.47301	4.57415	5.82952	4.8321	0.102674	9.05707
AAEL000739	hypothetical protein	114.928	110.199	152.466	148.223	102.237	100.27	1.30025	44.3078
AAEL000859	hypothetical protein	0.890602	1.30102	0.416815	0.513363	12.9899	5.69915	6764.35	2423.86
AAEL000969	hypothetical protein	32.5307	32.1279	19.2544	26.0244	29.9362	33.0373	9.98412	17.9914
AAEL001107	hypothetical protein	91.3948	127.537	251.973	182.435	131.119	207.724	1513.34	812.705
AAEL001471	hypothetical protein	26.5203	23.7254	21.1485	24.8188	52.2381	50.342	306.913	109.972
AAEL001519	hypothetical protein	0.566153	0.221752	0.304767	0.167835	2.31919	0.531229	33.371	12.181
AAEL001679	hypothetical protein	2.8793	7.00656	1.49191	1.32765	3.49406	14.763	17721.3	13779.2
AAEL001717	hypothetical protein	11.5828	10.9282	16.4187	13.6889	9.88193	13.682	3.3532	9.25485
AAEL001720	hypothetical protein	32.9198	34.4234	32.4188	34.4231	22.5669	24.7004	3.16974	16.966
AAEL001748	hypothetical protein	27.7212	28.1351	15.4124	18.3662	26.5726	29.7957	4.50504	17.8357
AAEL002162	hypothetical protein	50.0584	47.0338	22.1291	33.4377	43.8433	42.783	11.7757	28.2281
AAEL002283	hypothetical protein	43.5801	33.9389	104.991	145.341	47.2911	44.2736	1.0162	8.28317
AAEL002320	hypothetical protein	9.81817	8.46091	15.9114	14.7025	9.66138	9.31033	0.329062	5.4098
AAEL002369	hypothetical protein	22.3865	20.4095	32.4786	28.302	14.18	15.1655	5.29353	14.3905
AAEL002400	hypothetical protein	0.222484	0.199284	105.47	692.378	0.106273	0.298442	0.447195	0.123263
AAEL002586	hypothetical protein	26.6317	28.0823	75.6572	64.886	20.9423	20.7093	0.360058	18.9635
AAEL002757	hypothetical protein	0.94201	2.60912	1.38209	0.701356	10.5414	1.78399	18.6994	8.75428

AAEL002899	hypothetical protein	34.8597	34.609	38.9761	41.6553	86.653	38.6106	22.8622	42.5172
AAEL003008	hypothetical protein	15.4852	16.601	18.474	16.9713	22.8427	21.558	3.63187	9.32509
AAEL003197	hypothetical protein	0	0	0	0	0	0	120.367	57.8365
AAEL003261	hypothetical protein	2.71654	3.26052	5.09352	5.01441	7.64544	2.79953	52.3313	14.0189
AAEL003368	hypothetical protein	22.1686	23.181	26.6982	24.5841	23.1186	22.4791	66.258	30.2751
AAEL003667	hypothetical protein	6.99587	5.11673	5.30337	4.44462	9.20808	11.6479	249.395	108.537
AAEL003693	hypothetical protein	43.5704	42.0687	66.1261	40.6175	34.3822	34.6498	29.5794	35.1148
AAEL003783	hypothetical protein	8.09645	7.88079	13.7037	13.4251	6.25804	5.79666	1.38824	5.65456
AAEL004070	hypothetical protein	2.67327	2.47036	1.25155	1.69122	1.20731	0.85936	56.2546	25.6427
AAEL004336	hypothetical protein	36.5493	33.2082	61.0002	59.4267	23.0052	23.2179	5.46532	20.8618
AAEL004387	hypothetical protein	20.8391	19.0043	32.6947	27.5837	8.55119	8.0544	2.28052	11.6781
AAEL004454	hypothetical protein	97.0683	124.336	138.619	62.3207	249.005	161.209	139.132	105.864
AAEL004555	hypothetical protein	10.4381	10.4804	16.2384	13.7567	5.61968	4.78391	0.207244	4.73294
AAEL004593	hypothetical protein	6.61958	6.74893	5.65066	6.51955	13.0402	14.7222	0.286	3.73004
AAEL004695	hypothetical protein	12.0487	18.3785	11.6407	8.74289	10.6554	10.2164	30.2345	52.4132
AAEL004869	hypothetical protein	30.2767	30.3543	43.2822	37.818	35.2838	34.519	4.2191	16.3562
AAEL004912	hypothetical protein	0	0	0.515042	21.5965	0	0	0	0
AAEL005100	hypothetical protein	25.797	24.3706	23.0518	23.9198	16.0915	15.6132	7.10151	15.9354
AAEL005192	hypothetical protein	0.912911	0.348858	2085.58	1615.18	0.283405	0.307982	1.40632	0.537475
AAEL005256	hypothetical protein	29.6844	29.3157	24.5459	31.1047	31.2431	32.1586	3.4793	16.5181
AAEL005286	hypothetical protein	21.9844	22.4042	28.7741	30.6168	12.9963	14.9954	0.210369	11.7899
AAEL005331	hypothetical protein	47.0217	46.5705	31.7636	32.6181	231.646	90.2777	1465.17	537.887
AAEL005348	hypothetical protein	57.671	55.5354	56.8941	70.2702	57.0794	47.3089	10.1781	30.1812
AAEL005517	hypothetical protein	16.8527	14.3269	15.3963	16.1681	17.3329	18.2283	440.45	180.316
AAEL005679	hypothetical protein	13.659	12.7967	21.1866	14.4382	13.0397	15.7831	2.54108	7.51295
AAEL005940	hypothetical protein	0.013838	0.024794	0.057421	99.6824	0.045108	0	0.736668	0.416421
AAEL006078	hypothetical protein	9.78277	8.91691	19.2226	27.5062	6.80415	8.88079	0.702031	6.69994
AAEL006138	hypothetical protein	0	0	7.41814	9.68332	5.04166	5.30097	375.553	8823.48
AAEL006156	hypothetical protein	0	20.8536	0.398705	0.439689	0.774639	0.463937	1.17707	7.71862
AAEL006229	hypothetical protein	70.5367	70.3377	45.9166	58.551	56.9755	57.3652	7.28411	32.2575
AAEL006323	hypothetical protein	70.0454	56.1103	1.9351	3.38816	23.2558	22.7996	2.0416	20.0785
AAEL006555	hypothetical protein	28.0326	28.8047	11.2546	13.2483	23.2716	25.5565	2.06075	16.0156
AAEL006768	hypothetical protein	8.67235	8.75942	36.0871	67.4005	9.87124	9.68818	0.695345	6.95886
AAEL006931	hypothetical protein	68.2555	69.6768	88.4169	91.8659	65.574	65.9157	47.7195	53.6829
AAEL007295	hypothetical protein	10.5712	8.99138	13.9858	11.6637	13.7854	13.2178	0.505215	5.43453

AAEL007371	hypothetical protein	66.1601	60.4468	91.2854	77.6587	242.48	230.816	5.6813	34.1614
AAEL007571	hypothetical protein	26.5826	13.2826	69.3567	55.4084	5.1163	0.556945	0	30.6366
AAEL007610	hypothetical protein	1.41198	0.963255	0.904984	0.598436	0.734289	0.861482	16.6739	4.82048
AAEL007627	hypothetical protein	10.4667	9.05937	11.8138	13.6842	10.7219	11.2648	1.63375	6.88193
AAEL007726	hypothetical protein	13.8175	12.5284	11.2233	11.9671	8.27415	8.65274	0.564136	6.06438
AAEL008024	hypothetical protein	12.3702	12.2682	17.7246	27.4029	15.3371	11.9838	60.3591	23.8486
AAEL008106	hypothetical protein	100.78	99.4343	75.3291	70.8205	395.609	320.143	14.0355	30.0067
AAEL008280	hypothetical protein	0.057014	0.065782	45.5899	276.8	0	0.167237	0.128801	0
AAEL008446	hypothetical protein	0.162556	0.759367	0.120665	0.553787	1.16446	0.789077	17.924	5.17442
AAEL008520	hypothetical protein	43.3446	34.8738	69.678	47.3752	44.9489	51.2804	36.672	29.5655
AAEL008612	hypothetical protein	19.9329	17.6006	12.6153	17.1193	19.6058	19.7337	3.98753	10.981
AAEL008639	hypothetical protein	15.9719	23.7162	4.34717	5.24722	6.55329	5.70347	185.627	69.3764
AAEL008775	hypothetical protein	5.65314	1.14974	2.90412	0.530503	4.29915	14.466	0	0.093216
AAEL008792	hypothetical protein	28.2492	26.2165	45.01	38.3823	23.1632	23.134	3.47816	15.1167
AAEL008813	hypothetical protein	32.2352	34.0154	30.0413	27.6959	43.4892	57.0914	23.2864	36.3198
AAEL008830	hypothetical protein	42.3509	39.1385	44.046	42.9705	54.8173	50.7176	19.1845	29.5196
AAEL008944	hypothetical protein	9.6659	10.2807	10.9058	9.76526	11.1133	11.3558	0.359838	3.99307
AAEL009162	hypothetical protein	3.55848	0	10.3646	0	3.39666	0	0	0
AAEL009270	hypothetical protein	26.142	26.334	31.5842	28.2924	27.3207	25.1552	5.383	17.5452
AAEL009347	hypothetical protein	27.8028	25.8223	75.4158	44.1898	20.1366	22.7354	3.34879	13.5033
AAEL009403	hypothetical protein	1454.08	1558.41	646.053	631.242	3360.96	3450.2	3012.23	2333.03
AAEL009477	hypothetical protein	0.136198	0.071322	0.834764	0.375028	1.52696	0.709018	130.773	54.2937
AAEL009491	hypothetical protein	69.1833	57.7118	64.2182	92.4094	54.1609	66.4079	2.05848	32.8596
AAEL009519	hypothetical protein	594.703	578.735	464.002	434.873	525.306	587.904	16.0464	301.135
AAEL009612	hypothetical protein	19.6335	18.8836	27.9611	19.5131	47.3814	47.6871	9.82831	16.3209
AAEL009645	hypothetical protein	35.7938	39.5637	21.1451	18.8258	106.895	41.13	305.878	137.442
AAEL009730	hypothetical protein	9.96776	9.09568	10.8617	8.90712	12.248	10.7643	2.2011	7.15926
AAEL009756	hypothetical protein	32.2664	30.4822	38.8361	40.2884	34.867	30.5786	11.6269	20.3326
AAEL009901	hypothetical protein	81.769	81.9918	96.9745	102.355	61.6975	71.1812	9.49151	50.6414
AAEL009962	hypothetical protein	75.7533	57.1568	109.562	100.123	25.8729	26.8169	1.24696	47.2251
AAEL009968	hypothetical protein	14.1259	10.0605	25.9504	23.8888	9.07901	8.90864	91.5326	38.9132
AAEL009972	hypothetical protein	94.5158	83.2344	204.436	180.661	50.1788	46.7679	1.15813	61.6277
AAEL009989	hypothetical protein	5.88269	6.83308	8.15849	6.34708	8.06264	7.49234	126.979	59.8466
AAEL010010	hypothetical protein	14.1746	15.2184	21.8726	20.0505	17.3095	18.7162	1.9138	8.9713
AAEL010155	hypothetical protein	259.28	208.481	404.571	261.705	25.9766	29.621	5.39422	116.083

AAEL010229	hypothetical protein	90.6677	81.8729	78.0709	102.001	50.6291	58.0079	0.559518	55.3608
AAEL010416	hypothetical protein	0.056724	0.131109	0.454132	0.432924	5.13215	3.29915	319.347	130.256
AAEL010489	hypothetical protein	12.4535	11.8617	5.23103	7.12702	8.84715	8.9251	0.164269	6.15701
AAEL010652	hypothetical protein	215.645	237.973	412.259	291.128	103.7	158.271	481.572	205.967
AAEL010722	hypothetical protein	14.3817	13.7272	23.3192	20.9321	11.5047	11.5319	1.76755	8.15995
AAEL010820	hypothetical protein	26.4283	26.1531	17.5448	21.9862	21.0181	20.5657	0.890625	12.9856
AAEL010840	hypothetical protein	154.062	165.33	150.046	112.476	222.064	226.132	346.176	253.631
AAEL010899	hypothetical protein	53.4344	50.4407	17.0341	31.629	47.5242	48.1499	17.7796	32.5337
AAEL011262	hypothetical protein	22.6216	22.9795	27.2019	62.3589	3.29312	28.1741	27.8444	3.60136
AAEL011267	hypothetical protein	32.5181	30.3305	36.1467	34.0995	29.5588	31.8958	15.2774	22.2346
AAEL011362	hypothetical protein	9.11573	9.22361	12.6574	11.5939	10.3048	11.4256	2.08458	7.73148
AAEL011472	hypothetical protein	16.7872	18.1037	14.6759	12.2713	22.1197	22.8722	0.511169	5.9227
AAEL011532	hypothetical protein	19.1268	14.7695	25.6826	28.9106	13.3713	16.338	0.605675	6.31351
AAEL011847	hypothetical protein	38.3674	36.8729	31.73	29.5492	32.2551	32.249	8.32028	21.0089
AAEL011918	hypothetical protein	36.5527	41.1228	50.7162	40.5329	40.4456	33.9648	23.8575	33.0882
AAEL011998	hypothetical protein	1.55954	1.95658	0.750806	0.599957	6.60073	1.64886	222.207	93.9285
AAEL012085	hypothetical protein	4.94792	6.70983	6.38506	6.32739	7.90685	8.55074	96.4885	33.9792
AAEL012236	hypothetical protein	17.0301	15.9387	22.6424	21.0896	16.4037	16.9634	1.71341	8.21447
AAEL012244	hypothetical protein	24.4309	21.2662	24.5192	33.1179	28.3101	18.3931	121.184	61.1469
AAEL012322	hypothetical protein	242.462	267.309	423.313	346.707	160.496	167.929	882.773	460.324
AAEL012437	hypothetical protein	46.2699	49.4547	57.5896	60.5597	53.0011	52.6921	5.30202	26.1012
AAEL012445	hypothetical protein	60.3676	63.4035	95.1296	89.4409	74.5836	70.4064	10.8552	35.5968
AAEL012505	hypothetical protein	3.17245	1.35746	2.14466	1.90843	13.5598	11.8177	152.139	34.8393
AAEL012639	hypothetical protein	4.34456	0.128785	1.15652	0.443224	0.403032	0.330406	20.9884	1.88112
AAEL012976	hypothetical protein	11.7434	7.27627	14.5349	18.9271	2.55785	1.86848	0	9.88812
AAEL013317	hypothetical protein	20.5071	18.3986	24.0017	21.8221	22.1725	23.5836	0.913753	9.56412
AAEL013402	hypothetical protein	81.3703	58.2847	94.7044	88.7895	50.8231	55.1008	37.2129	38.9613
AAEL013578	hypothetical protein	6.49102	6.27045	7.49064	7.50915	7.05353	6.56396	1.40177	6.62703
AAEL013731	hypothetical protein	52.7212	49.4141	56.712	45.4913	62.8271	59.9555	34.9629	41.8085
AAEL013734	hypothetical protein	66.3571	67.1161	140.454	92.7629	58.6745	68.1344	0.383803	44.2507
AAEL013874	hypothetical protein	23.4392	21.2511	10.6765	19.7872	35.4561	14.9356	10.3486	29.5187
AAEL014128	hypothetical protein	95.8581	92.2027	2752.88	5350.38	37.1835	82.6639	6.05498	26.4185
AAEL014376	hypothetical protein	9.23557	7.87391	7.50975	7.4529	15.9848	16.435	0.89848	4.84117
AAEL014812	hypothetical protein	64.966	69.7416	38.1342	57.1042	58.4911	66.9375	29.0313	57.5928
AAEL014821	hypothetical protein	38.9322	49.7965	25.776	19.2119	25.4476	25.8878	1182.59	658.617

AAEL014827	hypothetical protein	19.0491	19.8967	28.6922	25.5994	20.4198	20.7807	8.0805	13.9585
AAEL015118	hypothetical protein	0	4.01132	0	6.16128	0	0	0	0
AAEL015306	hypothetical protein	19.5891	56.1731	21.3268	4.06994	14.2927	15.3152	11.2231	17.2512
AAEL015447	hypothetical protein	0.142891	0.181119	0.409368	0.485899	5.01283	2.1145	321.093	165.669
AAEL015606	hypothetical protein	49.0558	56.664	0.805206	0.883637	0.571363	0.992812	5.32558	25.8643
AAEL013784	hypothetical protein	10.7499	7.21608	12.6748	10.5788	8.59393	9.66476	46.5073	18.9206
AAEL009060	hypothetical protein	9.18478	7.51661	10.235	8.84258	8.57359	8.93362	2.88842	7.57209
AAEL003110	hypothetical protein	0	0.021973	2.23721	71.29	0	0	0.043652	0
AAEL009529	hypothetical protein	15.2671	13.5178	26.9486	22.046	15.9608	13.5366	132.425	64.6653
AAEL007449	hypothetical protein	41.8861	39.7522	12.2327	25.8654	22.1847	24.1349	13.4752	23.0192
AAEL001322	hypothetical protein	23.4583	21.5677	41.7352	37.6459	15.2969	15.5996	8.22653	15.7709
AAEL006968	hypothetical protein	189.91	198.966	282.619	241.418	214.358	226.029	4.91461	102.632
AAEL014126	hypothetical protein	0.949758	0.386984	1604.63	516.149	0.147426	0.092365	0.728608	0.414273
AAEL000167	hypothetical protein	22.3596	23.7185	12.6244	15.063	18.2317	20.1231	13.5058	19.8209
AAEL001357	hypothetical protein	26.5083	27.1897	36.7332	24.9102	24.0022	24.4853	3.22023	14.8562
AAEL000638	hypothetical protein	3.83623	3.40904	4.37494	9.64919	6.36257	5.63661	37.202	6.19147
AAEL011515	hypothetical protein	8.4795	8.42008	15.0722	12.1337	4.37906	6.32677	1093.64	456.382
AAEL003403	hypothetical protein	26.6496	27.4173	33.5415	34.0158	16.401	16.45	3.05191	13.2397
AAEL007264	hypothetical protein	0	0	0.491435	21.3193	0	0.061602	0.138659	0.101657
AAEL009787	hypothetical protein	10.0208	10.1442	4.48962	5.48026	7.18817	8.27752	0.227531	4.57793
AAEL007338	hypothetical protein	110.024	112.843	36.0641	53.5489	63.4327	56.6599	15.3693	64.139
AAEL005447	hypothetical protein	15.6891	19.0631	32.5371	28.1631	15.2555	17.6972	2.37287	8.5271
AAEL004315	hypothetical protein	27.1652	31.0753	35.1848	29.777	31.4766	31.2697	0.350045	11.4808
AAEL008050	hypothetical protein	19.2534	6.46978	0.854803	1.03148	18.1885	9.88404	1.25671	0.759334
AAEL001350	hypothetical protein	20.1887	20.7697	24.0929	19.6179	13.7431	13.3961	6.93496	16.1248
AAEL003816	hypothetical protein	3.57172	3.13401	8.58479	9.48151	19.1587	94.5958	1705.98	696.941
AAEL003821	hypothetical protein	1.04947	0.719978	0.47451	0.55183	1.30111	12.8613	102.933	62.3712
AAEL004421	hypothetical protein	174.649	174.887	140.652	190.841	161.585	156.986	380.734	261.82
AAEL011523	hypothetical protein	12.6719	13.0934	13.1983	8.31416	11.5768	9.50064	2.22373	7.73707
AAEL008785	hypothetical protein	255.876	229.243	104.874	106.582	242.148	247.47	18.4894	98.543
AAEL002865	hypothetical protein	176.126	178.236	68.9132	56.2445	112.932	141.98	620.441	386.689
AAEL000615	hypothetical protein	23.126	23.9741	33.1223	23.2346	37.925	42.147	2.31314	11.1382
AAEL011066	hypothetical protein	75.3894	83.5297	142.175	114.523	67.882	66.6939	79.0063	75.643
AAEL004462	hypothetical protein	63.4504	65.3494	32.4745	55.2047	49.6112	49.2562	1.05678	37.3134
AAEL008686	hypothetical protein	22.189	21.8589	67.7568	50.6558	18.3459	14.5852	19.7449	17.8216

AAEL000319	hypothetical protein	0	0	1.56285	68.4858	0.439638	0.479213	0	0
AAEL008704	hypothetical protein	12.6006	14.2269	28.3446	20.371	6.41666	6.83024	0.344778	7.49739
AAEL005474	hypothetical protein	108.119	105.281	82.2074	80.949	96.9813	86.3151	3.22535	45.5974
AAEL002470	hypothetical protein	31.1056	34.0718	20.532	47.21	34.2329	35.1023	29.7574	21.462
AAEL011240	hypothetical protein	64.9427	65.1334	69.5374	74.6731	75.2357	69.95	35.243	44.684
AAEL008938	hypothetical protein	42.3706	43.718	64.0457	54.264	44.2408	44.908	19.5037	29.5638
AAEL002496	hypothetical protein	30.7323	31.7403	36.0082	33.7118	27.0221	29.5673	12.5493	20.9937
AAEL012453	hypothetical protein	31.6599	31.8907	27.7112	31.2579	26.908	29.0502	0.499427	13.9661
AAEL014166	hypothetical protein	10.0057	11.6419	1.55969	2.29835	3.71371	5.3941	52.7994	21.4472
AAEL004537	hypothetical protein	57.7768	59.1484	48.6354	51.3719	13.4509	14.8609	2.06544	34.7392
AAEL005179	hypothetical protein	4.37771	3.99912	12.6211	41.0564	5.13713	5.32344	197.218	76.5801
AAEL010708	hypothetical protein	56.4323	54.3115	39.4194	48.1775	39.1039	42.5028	4.38309	37.341
AAEL012754	hypothetical protein	33.6837	33.7247	44.4861	38.651	0	34.9398	24.562	25.2304
AAEL002281	hypothetical protein	16.6678	17.4775	16.262	18.3781	23.1223	24.9331	0.237128	8.16382
AAEL011297	hypothetical protein	8.01465	5.68971	35.9573	370.602	0.287601	0.105193	0.789152	4.28483
AAEL013395	hypothetical protein	169.631	157.993	88.4087	148.861	106.899	103.496	7.73544	55.7004
AAEL009709	hypothetical protein	18.2475	16.8734	16.5179	17.7678	13.6049	13.7632	0.155943	9.51748
AAEL007805	hypothetical protein	36.924	36.2206	26.782	29.9297	37.5227	38.8131	6.64024	19.4693
AAEL003361	hypothetical protein	14.4841	13.1443	22.8214	19.8937	9.03682	9.08759	1.07733	6.07231
AAEL005132	hypothetical protein	7.38146	8.00682	13.8721	9.58698	9.78243	11.296	3.48017	10.3862
AAEL013710	hypothetical protein	0.764706	0.429097	0.782197	0.567658	0.389997	0.815217	41.035	17.3805
AAEL000551	hypothetical protein	121.278	157.333	18.8623	9.9352	107.805	115.006	23.5196	97.6714
AAEL008453	hypothetical protein	14.6977	15.7342	20.0063	18.3782	10.9269	13.3036	0.921265	10.2945
AAEL014852	hypothetical protein	13.7826	20.4463	3.58022	8.27575	3.25214	3.16473	2.38299	7.6336
AAEL003797	hypothetical protein	10.9956	8.11944	9.64178	12.7278	5.06064	4.43809	0.189923	6.08936
AAEL009459	hypothetical protein	0.223668	0.788771	7.56309	63.3132	23.3973	33.6422	28.4757	0.493649
AAEL004658	hypothetical protein	4.94585	4.26006	20.5547	7.36503	7.88514	9.68343	53.7237	27.2234
AAEL011980	hypothetical protein	11.5869	7.27122	10.6585	21.1622	16.9011	10.2423	767.562	344.388
AAEL004316	hypothetical protein	20.4899	20.5777	22.1285	24.3339	22.2459	23.3181	0.218407	9.39399
AAEL001274	hypothetical protein	64.369	35.403	59.4215	70.7326	22.3075	41.8347	0.497512	6.18522
AAEL000995	hypothetical protein	12.8654	17.3426	27.4148	29.2755	9.47599	12.5335	0.937501	4.67137
AAEL008470	hypothetical protein	23.5035	22.6733	78.3862	40.6591	25.5013	20.8694	155.485	80.2415
AAEL011119	hypothetical protein	18.3663	9.61858	27.2754	13.9956	42.5364	25.4713	0.72738	6.64213
AAEL003766	hypothetical protein	13.9151	14.0239	7.0519	41.6558	17.5921	15.7597	0.36317	4.40235
AAEL002778	hypothetical protein	45.8805	40.6466	36.9474	48.1634	11.401	11.2433	3.43381	22.6995

AAEL006046	hypothetical protein	36.8762	37.3959	40.0549	41.7847	19.8965	20.421	0.787423	16.768
AAEL007963	hypothetical protein	35.8714	42.1869	6.41315	6.80643	13.4265	12.1623	6.00879	19.291
AAEL006662	hypothetical protein	8.58602	13.2259	19.3911	18.8475	89.5667	125.488	0.580174	0.676
AAEL010593	hypothetical protein	43.6902	44.6107	87.6285	68.3616	24.5065	23.8173	1.10653	34.1745
AAEL008799	hypothetical protein	0.913958	1.45678	23.9	292.805	0.687415	1.06733	0	0
AAEL006545	hypothetical protein	27.1836	24.1187	33.4899	33.3515	26.6332	24.7471	14.1404	66.2956
AAEL008892	hypothetical protein	5.34138	7.38846	4.06943	6.63816	9.47525	8.9562	0	5.18238
AAEL014937	hypothetical protein	49.3688	136.646	116.756	77.7686	628.746	610.065	0.838782	36.6044
AAEL005755	hypothetical protein	167.971	206.639	147.453	123.155	224.217	206.111	238.318	233.036
AAEL010420	hypothetical protein	13.6605	15.1669	16.4167	13.1925	4.04439	4.37669	0	10.0347
AAEL003927	hypothetical protein	3.44964	5.91099	0.35406	8.26057	0.185732	0.305774	0	3.99568
AAEL000310	hypothetical protein	5.18935	5.16262	5.30636	13.2426	2.59093	2.02044	57.0645	26.2802
AAEL001062	hypothetical protein	2.38881	1.44589	2.51134	1.04903	3.14988	1.2835	37.1112	10.3497
AAEL001174	hypothetical protein	0.164372	0.037076	20.8518	229.543	0.022097	0	0.077005	0.128342
AAEL001362	hypothetical protein	114.179	116.132	215.665	186.579	113.204	96.2492	125.873	98.1263
AAEL001460	hypothetical protein	55.053	59.7334	64.317	62.4987	50.3251	49.6962	17.3784	32.8745
AAEL001558	hypothetical protein	21.4626	25.2606	61.0973	42.279	13.4087	14.736	16.1225	19.488
AAEL001815	hypothetical protein	18.5841	20.2106	18.3715	35.3337	19.0174	15.932	3.61883	9.20407
AAEL001873	hypothetical protein	0.081922	0.182109	119.904	665.226	0.280716	0.081838	0.193151	0.285204
AAEL002036	hypothetical protein	55.8474	54.6835	21.5207	37.5379	65.9101	66.4681	1.04932	30.1941
AAEL002402	hypothetical protein	38.1663	32.2348	53.8694	30.8022	30.5487	34.5931	9.4178	4.67819
AAEL002474	hypothetical protein	9.45814	9.23547	10.5996	23.4851	6.9577	7.14564	0.219455	3.50409
AAEL002480	hypothetical protein	37.3752	36.7123	45.2795	44.9774	41.6842	41.1691	9.48207	25.0219
AAEL002482	hypothetical protein	126.201	90.7763	0.991949	1.20014	0.609121	0.871813	4.2998	30.7674
AAEL002545	hypothetical protein	18.7583	15.1928	31.3164	24.1568	19.4671	10.8935	0.913412	31.9733
AAEL002592	hypothetical protein	117.804	105.037	6.70924	9.1935	11.0438	13.5619	1.21074	80.9506
AAEL002652	hypothetical protein	282.454	294.727	523.082	411.496	273.962	264.514	2.76416	152.585
AAEL002706	hypothetical protein	35.5007	32.6533	49.2929	41.5247	32.307	29.2013	11.8986	23.1728
AAEL003299	hypothetical protein	19.2196	16.6132	23.1274	18.8611	128.05	100.241	0.492181	5.60678
AAEL003590	hypothetical protein	28.2485	32.3623	33.32	31.6129	55.3123	64.3642	2378.14	1182.58
AAEL003596	hypothetical protein	0.031538	0.03611	0.255147	0.267544	1.60154	0.895972	142.376	70.2414
AAEL003656	hypothetical protein	46.5338	55.0251	32.5886	35.6429	62.4804	64.6421	3.25992	16.2154
AAEL003671	hypothetical protein	56.4882	58.5899	47.2875	47.4021	59.1408	57.8165	24.2745	39.2618
AAEL003764	hypothetical protein	0.351232	0	0.341137	0.498453	10.0258	5.15396	1725.67	714.497
AAEL003768	hypothetical protein	29.586	29.3403	22.7194	25.9642	30.8496	33.386	6.35536	14.0778

AAEL003773	hypothetical protein	53.7182	49.4616	113.51	91.2142	36.0634	32.9757	21.1189	36.0878
AAEL003843	hypothetical protein	0.618946	0.477245	0.151781	1.41571	4.47042	31.3224	21.5576	13.3541
AAEL004149	hypothetical protein	495.58	511.522	721.741	666.696	899.777	907.523	1703.45	1002.14
AAEL004230	hypothetical protein	9.8876	10.2159	3.15456	2.59316	26.5696	25.9735	64.839	29.5326
AAEL004272	hypothetical protein	36.5489	36.0861	47.9376	50.0027	31.506	32.1742	3.29224	17.0594
AAEL004624	hypothetical protein	7.04527	6.00191	4.1893	4.82403	9.20854	8.68407	0.223268	3.94587
AAEL005004	hypothetical protein	2.03807	0.681539	1.18559	0.555777	26.273	9.8465	0	0.472327
AAEL005212	hypothetical protein	130.078	95.8865	2517.2	4751.07	49.7067	56.499	3.55599	33.2937
AAEL005236	hypothetical protein	434.642	429.535	266.364	313.836	383.721	444.503	235.683	298.364
AAEL005281	hypothetical protein	20.8742	20.2538	28.6829	30.2807	13.8702	13.7492	1.14346	8.00347
AAEL005393	hypothetical protein	19.0604	19.3027	13.1027	19.3155	11.0396	12.3398	10.7368	20.0364
AAEL005421	hypothetical protein	50.8893	50.6368	70.0261	58.984	41.3205	40.0728	17.2429	33.1325
AAEL005506	hypothetical protein	6.01482	7.36497	9.41394	9.53124	3.3584	6.49854	0.279169	4.3855
AAEL005509	hypothetical protein	5.87257	4.87568	3.56943	3.06913	14.8641	10.6239	38.9807	12.9428
AAEL005938	hypothetical protein	50.301	42.2437	75.9706	48.448	23.5744	25.1448	0.833204	26.2976
AAEL006204	hypothetical protein	46.4936	47.5939	56.8855	47.1178	39.242	38.1858	16.3846	28.9171
AAEL006546	hypothetical protein	14.6161	10.2182	11.7387	18.2599	14.9814	3.61558	17.1579	3.25548
AAEL006750	hypothetical protein	14.3528	14.2346	20.5668	18.6783	12.9342	13.755	2.59795	8.60689
AAEL007000	hypothetical protein	9.31132	8.9541	6.4854	4.59432	7.43708	9.79061	0.243618	3.74973
AAEL007003	hypothetical protein	0.206319	0	61.4687	654.569	0	0.204135	0	0.716019
AAEL007199	hypothetical protein	12.7816	15.977	45.4316	55.4002	19.2768	18.4676	103.912	42.6793
AAEL007249	hypothetical protein	13.3674	13.0988	11.8009	13.2758	9.97935	11.7514	0.515838	6.7595
AAEL007460	hypothetical protein	13.2136	14.0228	13.4257	13.1734	16.2356	17.1103	0.689243	6.59481
AAEL007552	hypothetical protein	0.029342	0	1.6402	18.272	0	0	0.068462	0
AAEL007567	hypothetical protein	46.7032	46.0862	69.2967	51.6442	84.4504	78.2802	31.1354	31.8006
AAEL007775	hypothetical protein	3.37886	11.359	6.24687	6.5615	9.43248	4.26777	0.174792	2.03859
AAEL007813	hypothetical protein	51.4469	47.5155	76.4577	70.3997	48.5224	49.7041	6.5964	29.9491
AAEL007960	hypothetical protein	70.0341	76.4846	89.1632	79.9759	91.1363	93.5667	14.9715	38.7064
AAEL008405	hypothetical protein	12.7446	12.8645	13.9272	12.8769	14.4012	10.8121	43.9077	15.6836
AAEL008727	hypothetical protein	123.852	126.14	131.546	125.178	139.488	144.155	101.554	100.846
AAEL008936	hypothetical protein	5.1613	6.49687	12.1442	8.22324	102.581	71.3304	0.090685	1.79887
AAEL009070	hypothetical protein	63.7055	65.7265	104.154	68.9324	113.225	122.835	39.2689	54.2321
AAEL009262	hypothetical protein	30.4161	28.5278	49.039	39.9032	15.5636	15.5934	10.811	18.6591
AAEL009381	hypothetical protein	51.3611	50.0748	74.7766	65.5572	39.8981	40.477	15.6964	32.861
AAEL009654	hypothetical protein	53.1365	49.054	43.3137	46.0416	44.8594	46.9064	0.745096	25.413

AAEL009746 hypothetical protein	0.466471	0.288446	7.71798	142.268	0.138942	0.150351	0.070145	0.207803
AAEL009987 hypothetical protein	13.6635	14.4228	17.7518	16.7497	13.3461	13.9359	0.173631	6.66428
AAEL010282 hypothetical protein	15.0799	25.6506	61.8326	40.5728	20.6282	26.191	0	15.8978
AAEL010643 hypothetical protein	31.6206	22.7025	56.821	34.5705	28.0342	29.4187	14.6533	29.2294
AAEL010646 hypothetical protein	27.1304	28.6616	35.1717	42.7214	10.8756	12.0474	0.386718	13.8714
AAEL010755 hypothetical protein	40.6813	39.095	45.2323	47.4572	21.2997	21.6671	18.8695	25.4588
AAEL011138 hypothetical protein	51.3129	54.8834	30.2306	45.4717	58.1789	63.7755	13.4198	25.3965
AAEL012233 hypothetical protein	151.741	155.349	181.175	179.877	244.078	223.545	26.781	79.9246
AAEL012415 hypothetical protein	17.7138	18.9207	25.3064	27.406	7.52089	6.09533	108.1	53.0697
AAEL012416 hypothetical protein	21.4564	19.8749	29.3928	26.8452	16.5476	17.4113	5.61924	12.3097
AAEL012598 hypothetical protein	21.5845	18.0708	23.2361	23.3896	24.6151	25.3603	73.8797	31.6749
AAEL012630 hypothetical protein	2.53607	2.67505	4.58021	5.22691	6.98463	4.32635	60.8605	26.4867
AAEL012738 hypothetical protein	13.8995	11.926	25.3833	18.506	19.7961	17.5665	43.2284	16.2786
AAEL012855 hypothetical protein	134.665	135.662	16.3699	14.4643	108.256	94.3018	1.35125	45.3989
AAEL012928 hypothetical protein	5.49702	2.931	0.467945	9.49004	0	0	0	2.14351
AAEL013131 hypothetical protein	44.8963	44.5716	78.7857	65.0106	46.2809	48.8322	27.3381	34.6045
AAEL013738 hypothetical protein	22.1551	22.3721	88.3495	54.1487	5.67179	5.9254	0.845374	14.0267
AAEL013919 hypothetical protein	14.4279	13.9002	18.4735	17.0655	20.7453	18.303	3.7336	13.1643
AAEL014185 hypothetical protein	401.877	409.204	336.214	303.777	572.535	587.897	190.533	287.541
AAEL014380 hypothetical protein	10.6752	11.1803	5.35199	6.46498	13.24	13.7753	0.117542	3.63342
AAEL014694 hypothetical protein	18.4692	21.7983	11.6716	10.299	25.2993	24.0849	3.52768	9.86553
AAEL014828 hypothetical protein	13.636	12.9053	8.59805	10.5131	16.4293	14.2996	66.6687	29.4588
AAEL015128 hypothetical protein	34.074	35.3538	13.4207	17.4192	38.0971	38.0573	0.168893	16.9043
AAEL015319 hypothetical protein	71.4832	71.589	89.6045	90.1008	53.2404	58.1657	51.7731	55.6309
AAEL015451 hypothetical protein	7.83457	0	0	0	0	0	0	0.845134
AAEL007855 hypothetical protein	19.4494	18.3982	18.2147	18.0457	10.9614	10.3983	0.256797	9.99333
AAEL000309 hypothetical protein	7.64192	7.63636	4.38289	4.22402	11.4082	11.0004	0.327888	5.59035
AAEL009476 hypothetical protein	0.906974	0.781909	0.817519	0.855729	20.4799	4.45541	640.899	270.173
AAEL004539 hypothetical protein	12.0276	10.3131	12.605	7.09708	5.72023	0	0	0
AAEL011884 hypothetical protein	0.16262	0	122.53	142.575	81.8078	114.422	0.386084	0.28569
AAEL001284 hypothetical protein	7.18879	7.53738	6.48212	20.19	10.4874	9.06355	58.1933	35.4419
AAEL001135 hypothetical protein	65.1591	33.7578	22.6394	40.0979	19.7539	42.4932	21.6438	50.0045
AAEL005868 hypothetical protein	0.218762	0.12824	0.370208	0.426736	2.09644	1.99756	581.036	244.955
AAEL006037 hypothetical protein	15.4182	14.6066	10.5083	13.9933	6.04913	6.36878	0.601322	8.61326
AAEL014972 hypothetical protein	11.2252	25.8953	25.9693	43.6177	72.7723	79.8099	0.677377	6.21065

AAEL005922	hypothetical protein	84.137	78.8176	48.3128	64.5953	36.9892	37.5323	0.639422	47.0875
AAEL007870	hypothetical protein	13.8145	7.81067	24.4426	22.2464	18.045	20.4222	0.599982	4.17411
AAEL007872	hypothetical protein	0	0	0.071584	0	0.091822	0	62.4268	30.0478
AAEL015046	hypothetical protein	11.6396	10.4212	17.1176	16.3497	9.26143	9.48214	3.35694	8.42012
AAEL014451	hypothetical protein	37.5498	37.5932	37.9324	39.7411	78.9836	46.2294	10.6029	19.3283
AAEL004831	hypothetical protein	10.3543	12.357	6.46055	8.59778	10.3638	10.5584	0.223457	6.42224
AAEL010325	hypothetical protein	0.412553	0	0.149588	0.52992	0.543217	0	7.37783	0.362914
AAEL012987	hypothetical protein	45.9103	48.8865	48.2506	52.3184	30.0818	30.695	24.9693	35.9351
AAEL002612	hypothetical protein	3.61006	1.81224	1.40942	0.432421	9.16882	33.541	20.5554	9.05182
AAEL006803	hypothetical protein	56.1586	56.0447	90.5657	84.6385	53.3256	61.5369	3.08352	26.8248
AAEL011852	hypothetical protein	36.4771	29.7759	53.588	55.922	51.1209	48.5938	28.9851	35.3423
AAEL015217	hypothetical protein	3.95795	4.22719	3.13486	3.5128	3.36931	3.41352	55.3999	24.1869
AAEL006349	hypothetical protein	0.063276	0.076011	76.5232	42.3457	0.290214	0.251036	0.138162	0
AAEL014335	hypothetical protein	6.22516	5.15536	9.73407	18.6951	9.7803	5.08519	63.8799	28.155
AAEL002104	hypothetical protein	21.3965	21.4578	44.1294	35.7569	10.8376	11.6716	2.22652	11.6372
AAEL006191	hypothetical protein	27.758	25.9039	15.8721	24.8407	16.926	17.3613	4.22609	43.0604
AAEL012940	hypothetical protein	0.059523	0	0.210925	0.342566	0.518516	0.911735	151.299	70.2857
AAEL009148	hypothetical protein	30.9343	27.6537	46.263	45.5162	12.2024	11.2626	99.6129	48.6913
AAEL003376	hypothetical protein	34.6237	32.9825	47.5212	37.8577	25.7184	30.9241	15.3222	30.7236
AAEL004498	hypothetical protein	445.534	502.371	266.382	237.314	1236.37	1296.23	236.778	329.425
AAEL005357	hypothetical protein	71.1429	62.0661	45.0443	56.3722	31.4629	33.3833	26.1892	37.8054
AAEL004787	hypothetical protein	29.3958	26.9769	31.6064	33.7671	39.4639	39.1203	3.65931	14.545
AAEL013447	hypothetical protein	8.68139	9.57723	25.4238	47.1216	10.1868	9.54198	1.23185	4.54029
AAEL008675	hypothetical protein	24.4824	17.0758	0.068441	0.358244	0.354381	0.855795	0	8.49452
AAEL003186	hypothetical protein	0.150336	0.167998	0.112308	0.250319	1.03209	1.03006	131.546	64.9177
AAEL011075	hypothetical protein	19.7229	23.1628	36.0513	38.1984	16.9655	16.6076	0	10.2267
AAEL009408	hypothetical protein	28.1676	30.9687	22.5588	22.5382	23.2708	24.228	7.31716	22.0814
AAEL012570	hypothetical protein	75.1184	140.597	25.498	11.8722	60.4445	60.4115	599.988	444.813
AAEL003924	hypothetical protein	64.3605	65.7284	44.7115	47.235	71.8483	68.6283	68.8097	72.1453
AAEL006130	hypothetical protein	12.2995	15.3923	7.56703	5.74112	23.9864	37.1247	142.638	49.159
AAEL002685	hypothetical protein	11.0893	3.62495	14.0481	22.7403	5.80733	15.7835	0	0
AAEL010690	hypothetical protein	37.6782	37.3884	44.1139	33.8387	39.7739	37.7339	28.3788	46.7268
AAEL013706	hypothetical protein	9.20244	14.5476	3.91486	5.50859	5.43756	6.90095	0	33655.8
AAEL006904	hypothetical protein	9.32124	10.7142	12.0395	11.9275	25.5505	16.4459	149.268	46.6075
AAEL004489	hypothetical protein	39.9491	41.9808	63.309	53.137	35.0237	36.4795	11.8524	23.4408

AAEL001328	hypothetical protein	19.1197	24.3904	13.0053	12.6892	41.8759	36.8152	0.900884	10.5696
AAEL001529	hypothetical protein	0.935828	5.96071	0.431158	0.539731	0.619639	0.945695	0.17253	0.623702
AAEL004713	hypothetical protein	40.6841	42.7786	24.801	26.8593	48.1124	47.072	1.0005	11.1586
AAEL000915	hypothetical protein	0.111213	0	0.288304	0.457816	0.675255	1.13903	391.286	151.409
AAEL006493	hypothetical protein	0.107343	0.379053	0.895464	0.282774	1.99572	2.43283	369.816	131.084
AAEL007850	hypothetical protein	13.6419	10.5238	14.9492	13.4939	21.0873	20.3588	3.10975	9.25384
AAEL010634	hypothetical protein	160.553	126.741	38.7268	51.0038	320.748	332.267	2.49961	32.9847
AAEL006015	hypothetical protein	36.5591	39.8293	77.1233	59.1193	14.8327	13.6253	7.10358	20.9557
AAEL014542	hypothetical protein	43.5942	34.1059	93.7672	84.0034	21.6951	20.7201	18.4856	25.3594
AAEL008415	hypothetical protein	15.2542	17.109	17.3746	17.7135	16.1558	16.68	0.202005	9.61606
AAEL014268	hypothetical protein	2.26809	1.83509	0.283008	0.601769	0.513174	0.681588	139.878	34.2713
AAEL005007	hypothetical protein	38.0291	42.9068	44.8862	44.0898	30.995	31.4133	14.9493	25.2992
AAEL002235	hypothetical protein	27.7098	27.2201	31.3441	31.4293	24.2082	24.0873	10.1154	17.6422
AAEL012178	hypothetical protein	6.80611	7.83491	14.2564	12.1565	3.29251	3.76305	0.443247	5.30694
AAEL004792	hypothetical protein	80.0235	73.0835	44.1638	55.4801	113.482	113.153	15.0484	59.3024
AAEL005214	hypothetical protein	28.5351	28.4245	35.0422	32.2435	32.4894	32.9323	12.7965	20.9233
AAEL009352	hypothetical protein	21.2666	16.6353	0	60.4389	17.0292	17.6244	7.23686	11.446
AAEL013827	hypothetical protein	118.873	114.661	22.3267	25.0761	99.7496	103.601	11.6483	53.2253
AAEL002573	hypothetical protein	1.54213	4.08303	0.769117	0.825696	4.01019	2.47584	315.908	113.849
AAEL000607	hypothetical protein	15.4908	14.1831	14.22	14.2216	40.8588	23.7886	216.425	88.6752
AAEL006962	hypothetical protein	36.5649	37.2961	27.5375	20.3015	108.95	123.84	1.34466	17.1992
AAEL006392	hypothetical protein	42.9246	38.3485	39.5972	34.9577	42.32	47.6468	0.74675	18.2986
AAEL013484	hypothetical protein	10.1436	17.1947	40.415	34.6686	450.44	370.423	208.477	56.8353
AAEL015247	hypothetical protein	0	0	0	0.057664	0.306413	0.730329	42.9912	17.0216
AAEL003932	hypothetical protein	16.8721	15.8812	14.137	15.3423	17.6043	17.7872	5.94485	12.6933
AAEL014599	hypothetical protein	21.2062	24.8069	1.53971	1.91376	9.02983	5.39372	2.12215	8.98973
AAEL012504	hypothetical protein	58.593	56.2443	99.0937	73.1101	28.4933	34.6807	121.087	84.0583
AAEL000727	hypothetical protein	22.3625	23.454	26.7263	27.1818	21.4677	21.6608	6.74575	13.6905
AAEL008808	hypothetical protein	24.7395	18.9261	22.0182	19.6864	18.807	23.0748	1.02832	12.8406
AAEL007553	hypothetical protein	47.6054	40.6914	34.0158	45.048	68.9998	41.1376	151.547	116.573
AAEL002923	hypothetical protein	16.3339	6.68343	1.21024	3.18605	7.59338	4.65461	21.7888	12.9961
AAEL001392	hypothetical protein	5.92389	9.01583	12.5493	27.8438	21.8202	94.2669	0.80163	29.5922
AAEL011385	hypothetical protein	19.9844	20.3745	34.8601	28.1829	14.5997	14.3345	6.87764	13.6763
AAEL001927	hypothetical protein	27.2018	26.1202	44.233	37.374	18.1326	18.8763	19.5519	26.0117
AAEL008797	hypothetical protein	0.094597	0.437031	1.7265	94.4905	0.825232	0.567082	0	0.08168

AAEL000968	hypothetical protein	2.69097	3.24962	14.0281	8.94017	1.00404	0.594576	0.097373	3.17975
AAEL008395	hypothetical protein	10.0866	10.242	14.7816	12.7592	13.012	12.4533	1.18843	6.01278
AAEL007837	hypothetical protein	28.0635	25.222	61.4168	58.9824	20.4866	22.6241	6.13683	14.9069
AAEL009256	hypothetical protein	11.6865	8.72399	14.1211	10.7857	5.89166	5.67531	1.71887	8.03172
AAEL006233	hypothetical protein	10.1386	12.8226	4.30466	1.80909	12.8893	2.10207	62.3947	35.7328
AAEL005554	hypothetical protein	11.0287	14.2522	16.0533	15.3754	18.8167	18.2148	0	9.77796
AAEL003080	hypothetical protein	29.4913	36.411	26.2524	27.4575	21.1034	24.3369	14.8973	22.983
AAEL013311	hypothetical protein	15.4951	13.1924	30.9609	21.0786	16.8973	13.8412	1.17043	5.13675
AAEL011728	hypothetical protein	0.195399	0.453231	119.076	6.81969	0.356536	0.193419	0	0.342987
AAEL002059	hypothetical protein	23.8676	25.5447	39.8829	36.948	16.5201	18.8717	8.42543	15.0411
AAEL002327	hypothetical protein	10.7268	11.7886	23.8747	21.1361	14.8166	15.6094	75.9534	32.2932
AAEL003848	hypothetical protein	0.647911	0.496579	0	0.369372	0.767346	1.24481	332.383	147.213
AAEL007391	hypothetical protein	0	0	0.585318	0.136908	1.02058	2.38576	226.907	94.6543
AAEL001454	hypothetical protein	32.1358	28.2511	31.5263	31.3084	24.5364	26.7552	7.12966	17.289
AAEL000322	hypothetical protein	9.13352	9.10625	10.2291	19.8699	6.91659	8.26935	1.92769	6.95608
AAEL007479	hypothetical protein	26.5686	31.7877	78.4479	48.6875	25.4655	21.9635	8.61041	21.5702
AAEL010211	hypothetical protein	19.0016	17.7363	22.1783	21.4237	8.42081	8.73108	3.19654	9.45975
AAEL007053	hypothetical protein	13.3844	12.035	15.2024	15.4016	14.2392	15.0665	0.149927	12.0958
AAEL002473	hypothetical protein	351.18	309.827	470.966	548.476	144.118	171.274	3.64848	122.091
AAEL000565	hypothetical protein	51.5482	52.8157	31.8882	42.5201	39.0244	39.5326	11.1788	31.314
AAEL005623	hypothetical protein	0	0	0	0	0	0.166766	10.5631	2.03144
AAEL007440	hypothetical protein	0	0.055507	9.51281	28.8948	0.042586	0	0	0
AAEL011254	hypothetical protein	14.1613	14.626	13.3805	14.0922	49.2291	25.3553	72.1757	33.4935
AAEL003531	hypothetical protein	17.0976	16.1472	20.7592	18.6644	16.5274	16.5845	6.94098	17.0093
AAEL008617	hypothetical protein	18.3487	18.8932	9.83533	14.197	20.2062	19.6834	4.57342	10.109
AAEL004851	hypothetical protein	508.282	522.1	255.077	234.103	1425.89	1501.84	964.217	860.992
AAEL010818	hypothetical protein	246.227	266.257	281.849	232.463	593.827	588.353	453.522	357.752
AAEL007206	hypothetical protein	9.92344	15.3084	30.3386	35.0957	15.6479	13.7312	81.872	33.4852
AAEL001939	hypothetical protein	32.5275	31.9156	80.6852	43.9001	26.5347	27.1694	5.68714	21.1046
AAEL000604	hypothetical protein	91.5385	83.1082	211.434	227.667	58.9174	63.4411	36.1465	63.9231
AAEL008729	hypothetical protein	10.0226	7.57806	12.696	11.5983	8.79468	8.19244	0.467198	8.37594
AAEL010699	hypothetical protein	24.5725	25.3841	63.0048	67.0566	19.6543	21.0657	0.185123	12.8079
AAEL013634	hypothetical protein	26.3734	30.9572	17.079	17.6564	19.2999	17.7766	7.52303	14.7035
AAEL006556	hypothetical protein	13.6824	10.0422	33.9726	25.9772	6.68159	5.32837	0.193878	5.44075
AAEL011398	hypothetical protein	40.617	41.2144	49.5779	34.3471	48.0274	44.983	1.05622	15.5929

AAEL001186 hypothetical protein	7.59987	8.23884	5.57205	6.00036	10.5687	10.9987	49.1856	21.2413
AAEL000429 hypothetical protein	10.8196	11.5552	11.2149	12.4196	9.81478	11.0424	1.26857	6.89829
AAEL010914 hypothetical protein	10.1102	9.56867	13.0412	11.9041	5.88056	5.63476	56.7902	21.9723
AAEL006825 hypothetical protein	1.52398	1.57814	3.45795	1.95209	0.866507	1.00389	0.289198	9.73863
AAEL006146 hypothetical protein	9.51614	10.5733	16.5225	14.4584	11.9063	12.7215	2.44129	7.5633
AAEL015453 hypothetical protein	6.73421	4.47453	6.9647	8.27172	5.94408	5.93197	0.362437	4.08482
AAEL005332 hypothetical protein	109.519	116.968	56.9445	58.0349	359.829	268.506	186.543	147.37
AAEL004718 hypothetical protein	8.37708	9.96829	17.7594	13.784	4.68702	5.19998	1.18162	5.58135
AAEL012856 hypothetical protein	67.1987	43.6549	6.10951	11.4933	142.01	89.8425	4.46184	11.0596
AAEL009811 hypothetical protein	17.8107	16.7545	20.9549	15.8547	13.1497	10.3784	19.7368	28.6054
AAEL010271 hypothetical protein	47.0041	52.2053	26.8739	33.3373	48.5287	56.0666	18.0875	33.6691
AAEL000537 hypothetical protein	16.7846	0.236893	37.2964	6.92048	12.9569	0.098637	0	14.8314
AAEL002093 hypothetical protein	17.0717	16.3999	17.852	18.1245	14.5601	16.7813	2.4312	7.28764
AAEL009150 hypothetical protein	45.9188	46.0018	71.1961	65.7294	41.2446	45.3153	579.166	270.428
AAEL004356 hypothetical protein	28.3847	27.2487	44.2391	36.2144	23.2628	22.9663	9.97914	19.1614
AAEL006983 hypothetical protein	17.6167	18.516	35.1468	24.3299	19.511	20.1752	1.57526	10.6783
AAEL003404 hypothetical protein	320.256	303.184	580.313	354.616	158.246	163.277	3.42066	105.958
AAEL002920 hypothetical protein	7.25512	0	0.336836	0	0.323923	0.70679	1.37658	2.91097
AAEL012602 hypothetical protein	33.1635	31.5824	17.7107	26.0498	34.6025	32.5735	13.9065	23.3012
AAEL003159 hypothetical protein	24.9006	26.3771	36.8095	34.013	29.6918	29.6948	3.60301	13.6945
AAEL003842 hypothetical protein	8.48317	6.97995	3.61784	3.50506	10.2519	9.18581	48.1872	18.2676
AAEL009973 hypothetical protein	33.2849	30.8105	74.4642	72.5057	21.8564	23.3141	0.771339	22.6973
AAEL002492 hypothetical protein	20.6285	20.474	4.74441	6.86113	58.3839	29.2518	54.9431	20.4978
AAEL000252 hypothetical protein	41.7767	46.8784	13.5836	18.5841	37.9327	44.631	0.200601	4.95428
AAEL001949 hypothetical protein	19.548	22.9721	15.5495	12.7979	20.5473	22.6892	0.578082	4.63766
AAEL002889 hypothetical protein	133.993	2.50556	2.27457	1.52828	0.817833	4.76359	503.993	37.3961
AAEL007676 hypothetical protein	35.4784	31.4326	49.1674	47.0703	53.0137	55.93	2.41211	18.8818
AAEL000079 hypothetical protein	25.9473	25.5067	34.3238	30.3065	28.6395	29.0076	1.51859	14.0334
AAEL000365 hypothetical protein	46.4081	46.4838	32.9716	34.9825	42.3371	40.9653	13.0622	27.5903
AAEL010770 hypothetical protein	11.5853	11.6765	11.9934	11.3459	18.5187	16.8297	1.51764	8.56004
AAEL004140 hypothetical protein	58.951	55.1162	54.0537	45.6759	57.1091	56.6455	353.998	166.135
AAEL013293 hypothetical protein	2.97354	3.82022	1.79573	3.29131	4.51496	4.557	1.77583	6.56474
AAEL005501 hypothetical protein	26.3734	22.9253	34.3165	53.1409	5.63183	7.12033	4.20463	13.667
AAEL002205 hypothetical protein	85.9976	86.9731	46.2819	58.3849	75.5498	68.7867	26.6144	54.3415
AAEL006761 hypothetical protein	6.61145	6.21351	31.9505	58.8597	3.90701	3.69989	0.444243	3.85351

AAEL013204	hypothetical protein	8.73033	10.6625	23.3985	18.6665	14.7389	15.1222	0.05859	5.72671
AAEL009840	hypothetical protein	0	0	0.297607	0.043007	1.54911	0.924509	111.18	46.2283
AAEL007014	hypothetical protein	0.321632	0	79.4661	596.35	0	0.306825	0.349369	0
AAEL008586	hypothetical protein	26.2537	31.8825	30.5233	26.1282	14.0607	15.0065	11.2046	18.2883
AAEL012018	hypothetical protein	98.1165	102.789	73.669	81.6836	110.885	117.158	2.06426	49.7859
AAEL000786	hypothetical protein	70.6978	75.7554	24.7144	14.88	85.0177	78.8279	1.98802	31.8309
AAEL005172	hypothetical protein	23.0897	23.0165	67.2405	41.1222	21.7719	23.903	238.742	91.4707
AAEL005498	hypothetical protein	85.5723	88.4844	123.939	124.595	79.5533	90.6377	37.2838	57.9816
AAEL007368	hypothetical protein	18.4073	18.2643	30.5232	26.3659	18.3924	19.0937	4.16345	9.67464
AAEL003005	hypothetical protein	27.5209	23.7053	30.8761	46.0759	29.9003	31.9077	9.31365	16.1116
AAEL005295	hypothetical protein	9.54927	9.22924	14.799	12.8728	6.26101	4.63837	0.403918	3.99392
AAEL008019	hypothetical protein	2.57475	1.9512	2.25782	3.27551	3.57116	4.8324	81.1311	39.8406
AAEL007905	hypothetical protein	42.6699	44.2814	22.8052	28.6829	42.4629	42.7084	5.81816	24.3177
AAEL010014	hypothetical protein	0.049802	0.050523	0.0939	0.090159	0.850408	0.555254	57.6865	23.869
AAEL005153	hypothetical protein	25.2085	33.5419	15.2062	23.166	32.6783	35.2473	0	12.8261
AAEL007663	hypothetical protein	29.6719	32.0855	22.6548	25.1099	47.749	52.4291	14.3989	25.7479
AAEL012878	hypothetical protein	10.3377	13.3624	46.2826	28.3325	8.64463	8.5562	3.65526	6.97841
AAEL004274	hypothetical protein	32.9833	34.587	50.3495	42.297	27.4333	28.065	8.36177	19.5485
AAEL000353	hypothetical protein	0.21015	0.343593	55.4157	22.152	0	0	0	0.183085
AAEL000539	hypothetical protein	21.9316	38.3621	42.5177	38.5037	56.5262	48.594	124.532	46.3527
AAEL003878	hypothetical protein	18.9692	19.366	29.0568	21.702	26.5504	23.2234	1.44918	11.7953
AAEL004104	hypothetical protein	24.3833	19.3531	28.0153	23.1073	13.7976	12.8412	0.292094	10.5794
AAEL004136	hypothetical protein	12.5395	11.6317	14.0118	12.2341	8.82705	10.0723	0.333859	6.30946
AAEL004318	hypothetical protein	29.8946	32.0643	24.3949	23.2433	33.2967	36.5641	0.429142	17.2557
AAEL004934	hypothetical protein	13.3283	13.1461	14.9709	13.3517	16.8077	15.7349	3.47912	8.386
AAEL005117	hypothetical protein	93.7422	107.929	13.9211	16.1136	37.135	33.065	14.8498	66.0674
AAEL006602	hypothetical protein	97.7	81.1542	2.61359	1.85991	11.7886	9.58764	25.0667	44.6029
AAEL007166	hypothetical protein	162.108	142.556	151.411	160.424	231.025	232.653	42.0347	96.0027
AAEL007611	hypothetical protein	2.62898	2.55218	2.99601	3.63019	2.90587	2.94161	53.9524	24.3805
AAEL007867	hypothetical protein	40.7958	38.2789	47.215	48.7612	44.4474	43.2355	19.3163	29.2593
AAEL008433	hypothetical protein	5.6439	9.08336	5.04334	7.04962	9.66228	7.40881	0	6.4628
AAEL008441	hypothetical protein	1.12917	2.68944	4.38176	4.85525	41.3782	13.8563	6308.31	2607.72
AAEL009047	hypothetical protein	48.4029	47.1474	53.7727	57.3115	29.9591	31.4867	28.5515	35.6938
AAEL009346	hypothetical protein	4.89704	5.34389	5.23182	13.8753	11.9338	11.4023	58.6261	25.2848
AAEL009504	hypothetical protein	27.7807	26.1267	63.7637	44.0629	19.8663	18.7454	0.308132	13.0523

AAEL009725	hypothetical protein	12.1449	11.6506	13.7583	12.7173	11.6282	12.5907	3.38522	9.01773
AAEL011143	hypothetical protein	27.0688	26.8361	40.5282	34.7676	22.9516	22.5724	5.87269	16.1888
AAEL011351	hypothetical protein	16.4201	17.8349	19.3177	20.6098	22.9264	24.4694	0.213382	9.56589
AAEL011525	hypothetical protein	10.7072	7.76774	14.3461	6.7915	14.9747	3.64	38.6365	30.5014
AAEL012127	hypothetical protein	50.4462	79.7827	58.7152	90.283	56.0906	57.339	80.6338	47.4744
AAEL013292	hypothetical protein	18.7443	18.233	24.233	20.2137	26.4678	28.724	6.41561	15.3305
AAEL015289	hypothetical protein	0	0.024063	0.051908	8.99526	0	0.020849	0	0
AAEL007875	hypothetical protein	132.182	134.482	118.395	137.265	83.2812	80.2647	1.97821	71.7221
AAEL008399	hypothetical protein	21.3069	22.1156	31.448	28.3069	19.7671	19.7358	2.03783	11.3967
AAEL006149	hypothetical protein	23.2625	23.3143	36.6366	34.2049	19.6774	19.9605	10.7274	18.0145
AAEL006279	hypothetical protein	43.7516	40.3718	40.2127	48.6434	14.9882	17.3973	8.20726	24.4781
AAEL014338	hypothetical protein	40.5368	30.3943	45.1308	40.3163	32.2224	35.5378	0	5.22262
AAEL005115	hypothetical protein	40.3026	36.4866	106.061	94.4864	30.7146	33.3446	0.516481	18.5443
AAEL011319	hypothetical protein	4.87662	10.1005	35.1352	42.4746	176.236	158.212	37.6711	12.3645
AAEL011598	hypothetical protein	1.34554	1.50514	38.7764	24.9276	1.79596	1.4392	166.049	79.8333
AAEL009234	hypothetical protein	36.8307	32.0984	53.8915	53.2218	25.9429	27.2251	1.51563	19.5931
AAEL009188	hypothetical protein	8.35678	9.8314	10.3524	9.64559	19.8675	24.5502	130.785	50.3795
AAEL006569	hypothetical protein	7.46689	9.37875	4.64252	7.04834	9.20025	8.92692	15.8024	22.4221
AAEL010394	hypothetical protein	30.0751	47.3954	25.221	29.546	46.1417	39.7925	48.1921	40.9796
AAEL007680	hypothetical protein	12.4892	8.55429	8.5238	19.9623	53.726	55.1141	11.7095	18.5916
AAEL012516	hypothetical protein	31.0482	30.7299	38.6273	34.513	33.4605	32.4936	8.93734	19.035
AAEL009542	hypothetical protein	12.8219	10.5431	10.2339	7.83432	13.5107	15.2248	2.30765	7.06423
AAEL011582	hypothetical protein	22.6627	22.8385	37.5025	32.3565	11.8834	11.086	0.169493	9.05408
AAEL009074	IAP1	88.6934	86.3576	71.0882	72.2922	123.868	101.916	376.425	185.28
AAEL006633	IAP2	28.943	28.2102	15.3092	20.2304	26.1513	24.5321	15.1446	21.7545
AAEL014251	IAP5	23.6061	19.7428	49.781	39.6849	15.0359	17.4467	4.76864	21.614
AAEL006876	igf2 mRNA binding protein, putative	120.132	115.801	176.167	169.444	119.043	125.576	5.32469	44.5253
AAEL001965	imaginal disc growth factor	106.606	137.01	30.1454	23.1803	134.795	133.455	22.6023	42.0521
AAEL002023	imaginal disc growth factor	196.54	218.911	321.492	288.632	247.633	254.94	34.9142	116.717
AAEL000141	immunophilin FKBP46, putative	22.1172	20.8501	31.7705	25.7318	8.94827	8.51351	1.39301	10.7011
AAEL005390	importin	26.9061	25.4489	19.6176	22.1786	18.6458	19.3482	8.05362	16.686
AAEL004035	importin 11 (imp11) (ran-binding protein 11)	87.4487	83.3961	105.852	99.1877	49.3788	50.6922	11.4146	47.4047
AAEL009080	importin 7, putative	121.703	130.912	81.3938	74.8831	215.311	197.899	44.8473	87.4167
AAEL004441	importin 9 (imp9) (ran-binding protein 9)	34.6633	30.1155	39.6124	46.0076	31.6549	32.8429	8.50302	20.3458

AAEL011657	importin alpha	93.9355	98.9146	88.778	76.0892	125.408	121.655	69.1927	79.3684
AAEL012960	importin alpha	163.158	156.765	195.48	250.409	76.2268	80.1708	7.07287	93.8678
AAEL013275	importin beta-1	61.2004	61.5081	104.073	93.5028	45.8532	47.3304	15.3761	34.6471
AAEL007521	importin beta-2	54.2312	54.8052	104.567	57.3192	58.5726	62.5062	31.7831	35.6226
AAEL010698	importin beta-4	98.7004	101.636	52.761	61.5889	85.5571	85.9532	37.8366	63.3241
AAEL003650	Inhibitor of growth protein, ing1	15.4589	15.3258	23.4054	24.0182	9.8217	9.53178	5.77947	11.7732
AAEL003835	initiation factor 5a	546.966	593.935	415.574	359.468	868.797	856.762	550.243	509.117
AAEL003193	inorganic pyrophosphatase	93.7373	99.4389	98.0005	68.5411	98.9228	97.9687	167.337	108.812
AAEL009273	inosine-5-monophosphate dehydrogenase	37.4352	39.2605	58.6114	45.0283	31.3398	29.853	14.8977	28.1121
AAEL014719	inosine-uridine preferring nucleoside hydrolase	9.94722	9.14077	4.71362	32.2091	5.93419	5.92344	306.814	125.387
AAEL002176	inosine-uridine preferring nucleoside hydrolase	3.25347	4.56979	4.51627	15.4921	4.72295	4.5993	7.31408	5.85523
AAEL006485	inosine-uridine preferring nucleoside hydrolase	0.193437	0	0.787837	2.86295	0.044055	0.19083	6.22804	0.157544
AAEL010766	inositol triphosphate 3-kinase c	102.064	93.7339	194.391	154.206	56.0574	50.2046	7.98484	52.4945
AAEL000273	inositol triphosphate 3-kinase c	21.9027	21.0354	24.053	32.4807	35.4746	29.4297	117.861	59.9705
AAEL007457	insect origin recognition complex subunit	53.7801	54.8321	70.1229	67.6225	50.7151	51.9693	29.4879	39.6828
AAEL014250	insect replication protein a	23.5835	22.4041	19.5996	25.5806	10.8582	9.23072	6.49559	18.3226
AAEL011038	integrin alpha-ps	0.80419	0.628498	2.4057	1.13397	4.39588	1.17946	53.7719	17.193
AAEL012466	integrin beta subunit	16.9829	17.0801	17.7035	11.2789	13.0463	11.6868	127.491	40.0506
AAEL006048	interleukin enhancer binding factor	42.8635	48.4974	52.8985	37.0041	42.5919	43.2165	14.9942	32.7718
AAEL015636	interleukin enhancer binding factor	27.3201	30.7262	40.4595	28.4922	27.4521	30.0167	9.59549	21.5804
AAEL005221	internalin A, putative	63.3643	50.6635	99.6232	178.322	98.5417	85.4915	1420.74	591.057
AAEL014847	inx2	120.579	116.319	42.5127	35.4925	46.7122	48.2288	357.191	216.207
AAEL006726	inx4	136.859	141.069	50.7492	67.9682	117.133	114.701	1.99687	63.2131
AAEL008588	inx7	0.109676	0.065515	0.300805	0.246125	1.64743	1.6128	313.684	157.222
AAEL008117	ion channel nompc	0.135573	0	0.083384	0.053539	1.92326	0.214245	61.0802	24.4409
AAEL006144	isopentenyl-diphosphate delta isomerase	22.1501	25.2672	20.4807	21.0999	28.434	27.3615	13.0594	19.7385
AAEL008634	jnk	61.3924	57.1	105.636	85.949	48.6838	47.0022	16.6977	38.231
AAEL003505	jun	6.53344	6.63351	12.9349	15.6976	50.8311	17.3794	105.897	46.6271
AAEL005178	juvenile hormone esterase	20.448	26.8372	3.27862	3.16071	23.4952	17.3227	0.54271	10.9928
AAEL014438	Juvenile hormone-inducible protein, putative	0.420158	0.62122	1.40469	0.740118	20.693	0.772409	38.5418	16.712

AAEL014435	Juvenile hormone-inducible protein, putative	0.428282	0.644431	0.347976	0.330251	6.34742	0.503258	2.91924	3.02341
AAEL003954	Juvenile hormone-inducible protein, putative	0.744004	0.688132	7.46576	11.2597	11.298	3.16582	161.494	66.5164
AAEL001833	Juvenile hormone-inducible protein, putative	4.75278	4.6931	3.87416	3.1676	62.4581	8.4229	20.3381	11.295
AAEL004242	Juvenile hormone-inducible protein, putative	5.83936	6.85622	16.1297	9.37287	7.0236	8.19505	48.6034	19.6706
AAEL000516	Juvenile hormone-inducible protein, putative	1.7252	2.46616	4.26386	7.04244	16.9184	10.6533	154.349	74.2024
AAEL014440	Juvenile hormone-inducible protein, putative	64.5477	62.3378	56.6307	64.281	44.4787	44.3435	1.39332	23.9662
AAEL005827	katanin P80 subunit	23.8493	24.6435	56.6772	49.0706	16.0602	17.6248	5.54281	14.7353
AAEL010118	kelch repeat protein	21.8877	21.6883	23.4785	20.3651	21.275	20.8238	4.31775	14.5816
AAEL009227	kinesin heavy chain	7.50437	6.45684	11.5618	11.8176	7.74758	7.28783	0.641595	4.96268
AAEL008542	kinesin heavy chain subunit	68.8912	68.272	87.5652	67.6	60.2194	59.9203	59.3114	54.7109
AAEL002714	kinesin-like protein KIF23 (mitotic kinesin-like protein 1) (kinesin-like protein 5)	23.4614	22.0171	33.1006	31.285	23.594	23.5343	3.12611	16.47
AAEL004235	kinesin-like protein Klp10A	55.7554	54.8519	85.5507	80.6238	58.121	0	30.6494	35.7277
AAEL008928	Kir2A	37.0257	35.4586	44.8294	35.1387	59.7013	61.659	121.265	61.0628
AAEL001646	Kir3	0.258039	0.037834	0.093597	0	0.995048	0.161842	88.6362	23.0123
AAEL008879	kynurenine 3-monooxygenase	15.0749	13.5212	14.5676	14.1644	21.7683	22.2004	418.341	140.879
AAEL009295	lachesin	12.6401	11.8979	24.4895	12.0325	6.42767	6.68593	7.73532	9.71721
AAEL001895	lactosylceramide 4-alpha-galactosyltransferase (alpha- 1,4-galactosyltransferase)	0	0	0	0	43.6213	0	0	0
AAEL001900	lactosylceramide 4-alpha-galactosyltransferase (alpha- 1,4-galactosyltransferase)	0	0	0.093512	0.054194	0.195515	0.272008	35.9491	11.1037
AAEL000219	lactoylglutathione lyase	12.8254	12.0542	12.4892	12.0782	12.065	12.7936	150.68	79.8198
AAEL000335	lamin	150.529	139.084	180.232	161.613	84.5063	86.6263	1.62243	81.3561
AAEL007803	lamin b receptor	15.9185	15.1309	19.8459	20.103	7.48231	7.78286	2.85374	10.6709
AAEL003658	laminin beta-2 chain	37.844	37.8437	32.6081	24.9741	21.8308	22.4578	11.9871	18.0007
AAEL010930	l-asparaginase	1.43733	2.00919	2.40933	0.944674	1.6434	2.51602	448.077	202.921

AAEL009932	lethal giant larva, putative	20.3559	20.0545	17.58	21.6083	28.8633	28.0533	117.795	54.4845
AAEL013349	lethal(2)essential for life protein, l2efl	0.642211	0.838433	0.709225	0.203246	6.46319	3.05464	33.3609	7.02029
AAEL013341	lethal(2)essential for life protein, l2efl	14.5119	13.7281	13.3442	12.4877	13.6032	13.5303	47.1204	17.5411
AAEL013338	lethal(2)essential for life protein, l2efl	2935.19	2623.24	9631.26	8440.01	326.508	369.467	41.0152	1054.51
AAEL013346	lethal(2)essential for life protein, l2efl	0.471355	0.931034	0.098453	0.517149	2.71075	1.81212	16.8676	4.7028
AAEL006026	leucine rich protein, putative	7.87012	8.49432	10.5636	7.46233	12.2093	13.2572	1.95964	6.36896
AAEL012092	leucinech repeat	17.7348	16.2272	6.19334	4.31568	21.7405	17.7688	133.385	63.8928
AAEL003720	leucinech transmembrane protein	54.7892	51.9678	38.312	37.8179	44.1212	44.1037	3675.6	1691.46
AAEL003713	leucinech transmembrane protein	11.0282	11.8389	8.28326	9.53054	11.8712	11.5755	83.318	39.9165
AAEL002615	leucinech transmembrane protein	1.60956	1.24601	2.2492	2.8472	10.6703	6.24407	124.948	47.4416
AAEL003597	leucinech transmembrane protein	0.333844	0.305598	10.6153	3.3332	4.67008	3.94023	90.8998	39.0084
AAEL012093	leucinech transmembrane protein	0.163146	0.02135	0.120864	0.135009	1.94307	0.73239	108.537	43.2198
AAEL002295	leucinech transmembrane protein	2.63236	2.33436	1.93972	1.8481	7.52257	7.05402	782.402	273.232
AAEL002978	leucyl aminopeptidase, putative	178.404	161.201	233.3	240.156	190.679	204.209	1206.96	616.961
AAEL011058	lim domain binding protein	32.3014	36.3675	53.2734	44.1054	31.4304	36.4764	7.81909	18.8203
AAEL013438	LIM domain-binding protein 3, putative	4.63854	4.29118	8.4805	7.97567	2.87795	1.75751	74.4212	28.5463
AAEL005667	LIM domain-binding protein, putative	14.3844	13.4555	21.3623	20.8973	12.9223	8.40406	139.034	54.1755
AAEL007055	lipase	0.117894	0.277524	0.525746	0.100637	5.79304	1.60597	432.082	147.67
AAEL011008	lipase	1.60644	1.15786	4.99739	20.5999	0.63532	1.51821	0.110921	0.674663
AAEL012350	lipase 1 precursor	21.8829	12.58	33.9314	11.6856	41.8337	59.883	41.2332	47.1269
AAEL014921	lipase 1 precursor	0.25219	0.228429	0.140433	0.123095	0.182186	0.149322	629.387	193.958
AAEL012340	lipase 1 precursor	0	0	0	0	0	0	67.4409	13.315
AAEL009309	lipid depleted protein	27.667	26.7378	32.3702	27.4994	36.1533	36.0104	4.78266	14.7428
AAEL005951	lipid storage droplets surface binding protein	183.505	159.772	113.995	116.048	315.946	294.323	5.83978	73.9631
AAEL006820	lipid storage droplets surface binding protein 2 (lsd2)	775.59	722.011	753.56	605.718	520.78	522.812	387.589	595.822
AAEL009766	lipoamide acyltransferase component of branched-chain alpha-keto acid dehydrogenase	23.2793	25.2227	8.80154	8.85262	25.0018	22.6505	6.06072	17.4409
AAEL009957	lipocalin-1 interacting membrane receptor (limr)	38.075	38.8136	47.6615	42.561	34.4623	32.4311	16.6149	23.3523
AAEL007704	lipoma preferred partner/lpp	13.7652	13.3254	12.3089	11.4484	13.9741	12.7867	48.9165	21.9963
AAEL008058	liprin alpha (lar-interacting protein alpha) (synapse defective protein 2)	33.1536	32.1054	66.9474	59.4064	12.9707	15.625	14.0717	23.6779

AAEL005149	liprin-beta1, putative	15.6862	13.7167	12.5704	14.1849	19.6437	17.3584	68.6743	27.8918
AAEL014706	LL5 beta protein, putative	14.7932	14.2391	30.4796	26.9713	8.83921	9.23786	2.18296	7.88911
AAEL012014	l-lactate dehydrogenase	2.15642	11.308	21.2515	6.89874	2.85935	1.3279	897.377	175.615
AAEL009232	long-chain-fatty-acid coa ligase	3.93148	5.52626	2.47134	2.52389	2.44409	2.50297	211.995	114.215
AAEL008687	loqs	34.4708	39.6666	72.5269	45.0513	39.7449	40.6161	41.5833	36.048
AAEL003165	low molecular weight protein-tyrosine-phosphatase	46.4865	44.9787	64.3292	65.196	28.6652	31.7171	42.824	47.2607
AAEL007657	low-density lipoprotein receptor (ldl)	7.68316	10.9595	5.66621	5.56677	9.22538	12.9055	0.200916	3.33289
AAEL009869	low-density lipoprotein receptor (ldl)	3.98236	3.11438	18.533	8.41883	4.09613	3.032	0.721669	2.0952
AAEL014222	low-density lipoprotein receptor (ldl)	110.127	113.332	45.4334	63.576	145.817	133.015	0.87243	50.8158
AAEL008564	low-density lipoprotein receptor (ldl)	36.4738	37.6855	16.4485	25.1319	46.2583	49.6775	19.5492	31.9264
AAEL012086	LRIM1	54.6044	53.4414	41.9107	34.5163	179.53	169.117	3.79405	18.006
AAEL001401	LRIM10A	402.227	413.16	50.6314	48.4446	200.563	213.9	10.6294	169.749
AAEL001402	LRIM10B	445.818	405.052	75.4226	51.2845	416.896	389.869	8.86778	133.833
AAEL007103	LRIM15	23.8501	25.8797	29.3009	52.1484	42.7062	43.9663	1.08345	11.7685
AAEL010125	LRIM17	85.3542	100.279	10.7327	15.7758	52.4653	45.9736	2.73799	21.2047
AAEL012763	LRIM24	13.0912	13.1803	8.55752	9.91154	26.7454	23.5078	0.427928	4.85802
AAEL010128	LRIM4	25.4566	36.9645	10.5547	6.43243	41.2486	36.1922	0.175555	7.8148
AAEL001417	LRIM7	6.90047	7.94295	9.35754	9.21428	17.912	17.2779	0.149968	3.43228
AAEL001420	LRIM8	447.426	542.204	91.6844	70.4811	666.955	657.446	9.99511	124.259
AAEL001414	LRIM9	423.112	424.191	59.4114	47.3375	163.76	191.044	10.1816	102.907
AAEL005241	LST2	17.4267	19.352	93.3137	37.7929	24.0892	21.7853	22.6266	17.7449
AAEL003664	lupus la ribonucleoprotein	83.7687	88.2619	101.197	77.0949	74.916	72.6278	16.7194	51.6686
AAEL004134	lupus la ribonucleoprotein	8.81079	11.3609	16.8425	10.1655	29.1363	18.0696	3.77065	13.3484
AAEL010841	lupus la ribonucleoprotein	44.9021	46.5954	24.0477	38.2488	38.7538	39.6775	9.62576	26.7431
AAEL003723	LYSC11	14.4454	15.3887	11.1041	14.3521	61.7785	103.655	677.037	273.312
AAEL009382	lysine-specific demethylase NO66	23.6506	23.7175	24.3638	22.6105	31	33.526	2.43616	11.775
AAEL004933	lysosomal acid lipase, putative	0.045687	0.160881	0.067308	0.039146	0.081514	0.266782	645.099	319.908
AAEL005752	lysosomal alpha-mannosidase (mannosidase alpha class 2b member 1)	168.347	173.924	61.9106	89.0171	14.1388	14.1399	862.643	744.892
AAEL011357	maintenance of killer 16 (mak16) protein	26.6495	33.5674	41.5017	32.4374	54.4353	56.364	7.24413	18.4594
AAEL010046	maintenance of ploidy protein mob1 (mps1 binder 1)	45.4138	45.1933	41.8781	42.6468	34.242	37.2619	7.03772	22.9609
AAEL008166	malate dehydrogenase	148.069	165.597	183.507	156.999	157.287	149.625	215.398	176.939
AAEL005790	malic enzyme	62.2745	44.8182	60.9718	82.2314	306.397	301.382	191.186	78.0268

AAEL002069	malonyl coa-acyl carrier protein transacylase	13.9393	13.4566	12.0236	10.4703	16.5508	18.0012	4.71099	10.1184
AAEL012554	maltose phosphorylase	0.585632	0.615581	0.029148	0.121207	3.07718	0.291093	46.7778	17.9774
AAEL012555	maltose phosphorylase	6.2038	6.48983	0.937421	0.742757	0.697314	0.479763	0.269175	5.52473
AAEL014537	maltose phosphorylase	1.9673	2.47293	0.26274	0.321484	0.831696	1.05786	148.082	30.8162
AAEL010901	mannose binding lectin, putative	62.3652	72.232	34.9352	34.8611	37.6635	37.8915	69.6257	71.8043
AAEL011912	mannose-1-phosphate guanyltransferase	22.8688	44.1963	11.093	10.8995	11.7345	13.1252	26.2441	25.704
AAEL001331	mannose-1-phosphate guanyltransferase	37.5194	74.8376	12.8958	13.3602	12.4082	14.0197	101.982	69.6759
AAEL015438	mannose-6-phosphate isomerase	33.3829	49.3697	15.9061	14.9042	14.3909	15.4261	20.0807	27.9989
AAEL004389	mannosidase alpha class 2a	6.93666	7.54174	4.01312	3.9636	8.56533	7.84949	0.264026	3.6357
AAEL001442	map-kinase activating death domain protein (madd)/denn/aex-3(c.elegans)	20.6699	15.1431	23.0585	24.6494	14.8975	15.2466	0.584299	9.92408
AAEL002655	matrix metalloproteinase	3.57769	1.33109	0.569203	0.704479	30.6538	5.68184	0.067279	0.399499
AAEL005666	matrix metalloproteinase	6.2108	5.77433	9.72828	11.5324	4.67948	4.81698	153.901	58.0183
AAEL002661	matrix metalloproteinase	0.778239	0.385641	0.342388	0.120465	13.0941	0.856118	54.4344	6.17347
AAEL010797	Med21	33.1104	32.9633	88.3265	75.0262	22.548	21.4868	15.6357	27.8719
AAEL004195	membrane associated progesterone receptor	42.3077	39.9489	64.9428	53.3917	70.8996	69.3534	207.084	115.448
AAEL005699	membrane protein tms1d	37.7844	31.5206	30.123	32.1248	23.1976	23.5001	144.801	75.6954
AAEL001279	merozoite surface protein, putative	46.9443	46.0718	19.4212	25.2462	43.1815	40.995	37.234	14.2654
AAEL014341	metallocarboxypeptidase, putative	11.7912	11.6339	15.6671	14.365	14.5267	15.5305	2.07483	6.57564
AAEL010073	metalloendopeptidase	18.1681	19.5642	26.603	25.1392	28.0764	27.4191	7.08311	16.1001
AAEL011542	metalloproteinase, putative	0	0	0	0	2.51546	12.3764	17.3306	29.5963
AAEL013150	methionine aminopeptidase	10.3723	11.2579	15.3593	15.7499	19.3809	19.4509	2.84913	7.97723
AAEL004738	methionine aminopeptidase	69.262	71.1821	76.8386	64.5504	89.934	85.7616	39.7667	45.8212
AAEL010751	methylenetetrahydrofolate dehydrogenase	111.595	89.3268	110.369	109.823	101.021	34.2451	28.4758	69.1867
AAEL006085	methylenetetrahydrofolate dehydrogenase	81.6184	125.598	22.1863	8.87554	44.0674	38.6961	29.2471	63.8098
AAEL001134	methylmalonate-semialdehyde dehydrogenase	77.9979	97.6857	48.4047	33.6924	84.9941	78.6223	26.5258	50.3773
AAEL013967	Methylmalonyl-CoA carboxyltransferase 12S subunit, putative	17.6711	25.3885	14.2545	11.6163	38.8801	36.7415	6.25903	12.7993
AAEL006435	mevalonate kinase	35.6647	32.7298	38.9462	33.5958	35.7427	36.492	11.5546	24.8602
AAEL003192	mfs transporter	4.90772	4.05594	2.15254	2.23712	2.13991	1.53716	157.002	77.8248
AAEL013141	mfs transporter	312.813	295.945	711.412	343.17	143.197	161.339	4.36	173.317
AAEL012183	mfs transporter	162.983	162.936	358.538	185.978	78.7404	86.3603	2.69002	80.0847
AAEL000800	microsomal dipeptidase	40.5207	39.8759	69.6101	81.0867	61.8812	66.2908	262.386	134.223
AAEL006829	microsomal glutathione s-transferase	101.544	146.376	102.618	101.454	92.9161	92.0677	897.3	449.951

AAEL010157	microsomal glutathione s-transferase	0	0	0.291403	0.112523	0.615596	1.06878	206.324	87.0409
AAEL000947	microsomal signal peptidase 23 kd subunit (spc22/23)	151.126	171.812	63.7859	54.918	58.9494	63.5027	180.57	165.119
AAEL005712	microtubule associated protein xmap215	68.7166	65.5093	81.5153	75.792	57.98	56.2771	12.2183	38.0118
AAEL001605	microtubule binding protein, putative	69.663	72.2519	109.708	90.2856	47.9127	48.483	39.498	58.1156
AAEL013242	microtubule binding protein, putative	8.23001	8.37948	24.7704	20.3913	11.9084	10.4995	2.66688	7.6976
AAEL004176	microtubule binding protein, putative	56.7481	57.2938	63.5732	58.2998	60.5766	55.2122	208.305	113.837
AAEL002771	microtubule binding protein, putative	33.3713	33.2023	23.5323	24.3864	31.7402	30.4146	9.7996	21.1776
AAEL006911	microtubule-associated protein	20.2525	18.7328	23.9697	22.12	24.0245	24.9839	94.3486	35.9672
AAEL008657	midline fasciclin	164.16	151.347	95.283	159.253	106.539	103.972	12.8842	58.4458
AAEL006752	Misexpression suppressor of ras, putative	37.8226	35.4283	46.525	36.4166	18.6253	19.3934	12.9081	26.6158
AAEL009391	Misexpression suppressor of ras, putative	40.8836	42.6577	34.9773	29.805	29.1496	30.534	8.6015	30.0443
AAEL000593	mitochondrial 18 kda protein (mtp18)	17.2925	18.85	25.237	22.6402	13.6979	17.2902	3.1085	11.2866
AAEL004067	mitochondrial benzodiazepine receptor, putative	19.4223	18.8919	15.3315	12.613	30.4784	21.3113	431.382	202.011
AAEL007046	mitochondrial brown fat uncoupling protein	17.3247	11.8886	120.124	86.9085	14.5153	12.8689	0.436074	10.8449
AAEL006262	mitochondrial carrier protein	49.0336	41.92	68.0095	57.2211	45.2038	46.7257	27.4629	33.6797
AAEL008494	mitochondrial carrier protein ymc	173.445	198.544	26.578	35.765	122.812	95.5769	4.22694	97.9054
AAEL010002	mitochondrial import inner membrane translocase subunit tim17	41.3436	47.2454	37.7595	38.637	61.6388	58.2161	25.7226	38.1805
AAEL007001	mitochondrial import receptor subunit tom40	48.9529	55.9556	58.9061	44.6055	105.352	102.589	37.4386	45.2173
AAEL008128	mitochondrial inner membrane protein translocase, 13kD-subunit, putative	42.4363	42.6788	39.4939	23.9373	65.5395	63.3796	41.3062	33.3849
AAEL002523	mitochondrial inner membrane protein translocase, 9kD-subunit, putative	62.0828	57.1496	106.913	99.3077	53.5463	53.1912	43.4202	55.7681
AAEL005693	mitochondrial NADH:ubiquinone oxidoreductase B16.6 subunit, putative	12.0123	14.7033	15.8559	9.52526	25.9615	12.7299	43.4073	31.6566
AAEL009100	mitochondrial oxodicarboxylate carrier	21.3979	18.5773	8.97577	9.88154	8.45935	8.25558	94.8389	44.4532
AAEL011195	mitochondrial phosphate carrier protein	236.779	266.169	291.401	240.652	595.236	585.773	460.263	347.594
AAEL013643	mitochondrial transcription factor A, putative	25.7966	25.9568	31.6482	28.5687	23.2856	25.805	10.2387	18.041
AAEL011376	mitosis inhibitor protein kinase	18.5484	19.798	28.2252	24.7234	14.0195	15.555	3.06327	10.8001
AAEL006140	mitosis inhibitor protein kinase	21.8486	23.2802	8.37875	14.0568	8.16304	8.50987	7.25172	14.3758

AAEL011596	mitotic checkpoint serine/threonine-protein kinase bub1 and bubr1	14.2186	11.9932	42.1267	36.0386	16.7848	18.0249	2.00447	7.91396
AAEL008316	mitotic spindle assembly checkpoint protein mad2	14.1799	12.102	42.2316	33.4376	6.55077	7.63568	1.40281	7.39865
AAEL014329	mixed lineage protein kinase	50.2924	48.0406	42.096	40.5442	42.2287	41.8053	25.4954	37.8696
AAEL004120	ML1	77.7553	106.332	17.5121	7.87103	75.7556	68.0478	3.891	34.7942
AAEL006854	ML13	35.4011	33.6966	33.8784	29.0609	27.639	27.0075	6.35291	16.8773
AAEL009557	ML17	0.59142	0.231877	746.298	311.119	0.52756	0.568301	0.884657	0.675291
AAEL015138	ML18	0	0	0	0	0	0	27.9542	6.77578
AAEL009760	ML21	1.82914	0.117391	3.92581	2.8758	8.87323	36.3369	852.833	441.688
AAEL015136	ML6	2.52322	2.0149	0.403741	0.378159	3.34672	6.96449	13474.1	5842.49
AAEL004823	MnSOD1	79.1252	88.9277	76.7392	72.1745	110.886	110.737	55.5407	71.4777
AAEL010576	modifier of mdg4	673.723	643.456	846.067	789.737	594.347	603.602	118.312	378.143
AAEL007915	moesin/ezrin/radixin	43.3621	44.7754	71.6461	66.9475	50.0282	51.0961	115.318	59.5371
AAEL011727	Molybdenum cofactor sulfurase 2	0.09922	0.03334	48.254	3.15544	0.025904	0.028143	0.03162	0.094344
AAEL014272	molybdopterin cofactor sulfurase (mosc)	7.43677	7.4591	5.2611	5.64594	5.86862	5.68844	135.567	46.5855
AAEL007640	molybdopterin synthase large subunit, putative	8.51838	14.3923	8.84364	18.3316	7.06448	6.3656	25.7396	6.97076
AAEL000417	monocarboxylate transporter	16.2562	14.0918	12.1953	12.2311	11.9181	13.5774	328.469	171.306
AAEL003524	monocarboxylate transporter	54.5564	38.1553	13.1502	14.3537	14.9778	14.48	402.451	198.175
AAEL005513	mothers against dpp protein	38.2453	35.3894	93.887	77.6869	24.0563	23.2723	16.1865	23.8426
AAEL004813	M-phase phosphoprotein, putative	21.887	24.384	25.4826	20.5991	41.8138	43.6397	12.0823	18.477
AAEL011522	MRAS2, putative	12.3445	13.941	0.523472	5.34922	1.74551	1.43817	0.795656	8.60524
AAEL012990	mrg-binding protein	6.79009	6.05242	5.64218	7.34571	5.49094	6.06159	3.69773	8.79927
AAEL006887	mrna turnover protein 4 mrt4	16.6837	21.0945	18.5327	13.9483	27.4605	23.0967	5.26574	10.6967
AAEL009613	mRpL36	52.6107	51.1814	41.555	37.0696	44.7127	51.5217	26.615	42.5972
AAEL006386	mRpL39	19.2684	21.2682	30.6511	23.7418	25.2085	25.8124	9.2388	15.6083
AAEL006117	mRpL7	55.8673	55.2342	60.317	45.7135	80.5228	88.164	39.3511	48.4981
AAEL001280	mRpS15	2.3055	1.38817	4.10269	3.41509	2.36301	2.2254	1.37955	5.84357
AAEL009608	mRpS16	41.2507	38.4189	38.7094	37.2302	42.8084	45.5289	23.4643	33.5717
AAEL001615	mRpS18C	25.6956	23.1272	29.732	29.3471	31.7732	32.9408	7.71879	16.6413
AAEL003801	mRpS5	26.8169	27.0851	36.2346	33.7162	40.6825	41.0448	15.5588	21.8369
AAEL012323	MSF1 protein, putative	32.6867	33.783	37.4448	32.2413	38.2466	34.2164	17.5842	27.1573
AAEL013880	MSF1 protein, putative	70.3	60.7379	103.041	95.382	150.058	140.75	296.535	150.569
AAEL001667	multicopper oxidase	6.73547	2.31247	0.817263	0.863511	7.31783	2.78242	3197.78	1188.25

AAEL007415	multicopper oxidase	59.3306	37.0638	0.471837	0.586975	1.88135	2.06894	0.175381	23.5667
AAEL007802	multicopper oxidase	0.350366	0.209007	1.03561	0.612839	0.656561	0.678524	92.4796	29.8668
AAEL006583	multicopper oxidase	25.5326	16.127	0.180354	0.256961	0.375338	0.209553	0.329958	9.07502
AAEL012189	multidrug resistance protein 1 (atp-binding cassette C1)	2.41994	3.85747	2.17967	1.80898	19.6673	4.28413	11.4115	8.65813
AAEL011510	multiple inositol polyphosphate phosphatase	0.067641	0	0.02517	0.116391	0.247298	0.235541	90.0928	32.8365
AAEL003670	myelinprotein expression factor	100.017	105.234	57.9696	58.307	80.0677	80.8425	71.8993	78.8251
AAEL008701	myoinositol oxygenase	32.4512	27.9261	21.1047	26.6852	64.0021	51.7628	1.68913	8.7741
AAEL001411	myosin heavy chain, nonmuscle or smooth muscle	34.6135	33.0628	48.816	36.7187	29.4119	27.5098	140	64.7276
AAEL011905	myosin i	8.94301	8.92057	6.92525	7.04144	20.167	19.941	726.719	333.661
AAEL012631	myosin i	14.2888	11.0813	37.3439	26.3537	30.5996	27.5869	247.354	105.842
AAEL009991	myosin iii	1.57632	2.08815	1.65033	3.75465	3.53624	2.74271	93.9616	36.8663
AAEL007632	myosin light chain kinase	8.2714	8.10254	17.1867	15.5924	6.28369	5.465	0.362303	4.00266
AAEL008921	myosin regulatory light chain 2 smooth muscle	77.8398	76.8026	93.6635	89.8891	63.1177	67.8593	340.762	176.517
AAEL004227	myosin vi	14.5882	14.6363	9.58427	10.4822	17.8728	16.011	407.856	159.256
AAEL008610	myosin vii	0.063758	0.033577	0.194391	0.056067	2.01605	1.31294	162.608	59.1962
AAEL011436	myosin xv	9.43632	8.08139	14.2202	14.5866	5.10882	5.49541	1.11797	5.60511
AAEL013729	myotonin-protein kinase	22.7599	21.885	37.0197	36.1639	20.1665	20.4348	7.49155	13.8751
AAEL006022	myotubularin	20.099	17.616	20.9203	19.5472	20.5456	20.5173	5.86	11.3868
AAEL000053	myotubularin	13.5835	11.745	26.4031	17.3862	11.7503	10.9624	0.712311	6.7391
AAEL007672	myst histone acetyltransferase	18.6144	18.7139	30.8556	27.5791	23.3295	21.6191	5.25001	10.8799
AAEL009406	n(4)-(beta-n-acetylglucosaminyl)-l-asparaginase	26.8086	24.9351	10.4056	10.2864	23.7503	26.3574	340.906	169.2
AAEL012062	na+/k+ atpase alpha subunit	103.293	96.049	140.396	122.507	134.137	130.56	505.057	239.451
AAEL001146	n-acetylgalactosaminyltransferase	1.41108	0.818491	3.14085	35.5791	1.81827	1.14877	18.536	7.33589
AAEL001151	n-acetylgalactosaminyltransferase	10.0185	9.28861	10.8087	44.1442	8.58194	8.30827	36.2789	17.663
AAEL004538	n-acetylgalactosaminyltransferase	155.565	187.546	235.224	248.102	102.249	132.915	112.481	113.019
AAEL012823	n-acetylgalactosaminyltransferase	4.10431	4.0628	4.29326	15.2096	4.40961	4.36727	5.43865	4.20637
AAEL001122	n-acetylgalactosaminyltransferase	4.94731	2.14759	3.85217	5.33221	1.52847	1.07189	78.3879	32.6782
AAEL001121	n-acetylgalactosaminyltransferase	6.53123	6.61588	6.67681	15.0674	8.18584	9.881	85.5043	41.578
AAEL000654	n-acetylgalactosaminyltransferase	9.85266	10.913	9.99588	13.1847	11.6579	12.5447	199.865	90.8103
AAEL000639	n-acetylgalactosaminyltransferase	1.91533	1.21002	2.37918	18.925	1.76037	2.09256	163.699	71.2229

AAEL013897	n-acetylglucosaminyltransferase vi	35.5837	35.0852	46.1237	46.4375	21.8069	21.7632	12.373	22.1861
AAEL011899	N-acetyllactosaminide beta-1,3-N-acetylglucosaminyltransferase, putative	0	0	0.017882	0.066305	1.46739	0.504429	31.5315	11.3379
AAEL005709	n-acetyltransferase separation anxiety	65.2596	70.3452	103.027	90.6533	71.7655	70.1628	26.5323	47.442
AAEL011157	NADH dehydrogenase, putative	65.3167	63.4268	65.6598	59.2272	87.3149	84.7989	100.717	101.167
AAEL002825	NADH:ubiquinone dehydrogenase, putative	75.3573	103.457	80.462	69.6905	117.269	114.08	247.225	154.873
AAEL002881	NADH:ubiquinone dehydrogenase, putative	328.462	373.628	284.511	240.353	410.504	399.483	788.903	499.267
AAEL014673	NADH:ubiquinone dehydrogenase, putative	5.18326	13.9949	9.4527	6.83909	6.80869	9.84524	7.19335	10.2373
AAEL014689	nadph cytochrome P450	22.4661	19.8374	23.2794	23.8261	41.5929	30.9004	86.3314	37.5184
AAEL003349	nadph cytochrome P450	26.6752	28.4157	31.5191	27.8098	53.8607	45.7782	120.141	58.0559
AAEL000477	nadph fad oxidoreductase	15.8977	16.5252	20.7373	19.0077	12.6968	10.8347	6.52429	12.6904
AAEL000746	nadp-specific isocitrate dehydrogenase	112.626	127.956	110.554	105.183	59.6355	59.6059	668.813	406.465
AAEL009651	nascent polypeptide associated complex alpha subunit (nac alpha)	121.554	108.768	74.5136	53.9054	253.583	260.753	69.9436	89.1358
AAEL011711	nascent polypeptide associated complex alpha subunit (nac alpha)	74.8211	85.8163	69.073	45.5408	194.704	182.453	48.4519	66.5408
AAEL009779	NBP2b protein, putative	19.6996	20.9925	21.7501	19.6422	22.1293	24.7628	14.4905	21.3799
AAEL009579	NBP2b protein, putative	12.1315	12.7841	17.4417	14.8462	12.4256	10.4749	3.38464	8.70739
AAEL007289	NBP2b protein, putative	41.7435	40.7033	56.1722	51.3966	54.1207	51.4677	13.0308	29.8407
AAEL002375	NBP2b protein, putative	36.3281	36.9216	53.5451	42.4116	61.5492	60.3647	21.4643	29.0296
AAEL010803	ncd	12.7184	13.4008	38.2143	33.9353	11.6382	11.9202	2.63813	7.93199
AAEL000558	neural stem cell-derived dendrite regulator	9.74997	9.44835	14.554	12.5694	7.89373	6.96348	0.594691	5.14577
AAEL011396	neurogenic locus delta protein	22.9419	21.9921	39.13	40.1063	14.3229	14.4968	3.33757	11.0671
AAEL013689	neuronal calcium sensor, putative	5.97119	5.40212	8.63153	6.62211	6.45322	7.0557	66.8603	27.6043
AAEL003469	NHP2 protein, putative	49.4765	55.7536	78.1666	68.6232	68.7688	77.7243	17.5286	34.6357
AAEL004923	nicotinic acetylcholine receptor, putative	5.4638	7.52894	2.94393	2.63111	7.41234	5.44561	0.139585	3.21239
AAEL007226	nidogen	35.4868	44.8555	0.612889	0.408934	1.60944	1.35526	1.10868	16.1536
AAEL009531	niemann-pick C1	0.041389	0.177109	0.040206	0.069821	0.959611	1.23633	360.97	146.528
AAEL014038	NIF3-like protein 1, putative	15.1471	17.7823	14.3751	15.43	13.4458	13.843	9.3139	15.2474
AAEL000206	ninjurin A, putative	0.117898	0.269132	0.262276	0.302672	2.56627	1.74941	391.588	168.926
AAEL012261	nitrate, fromate, iron dehydrogenase	18.3557	17.3071	17.3231	16.7477	21.7212	23.3976	93.4098	48.5586
AAEL000111	nitrilase, putative	49.8627	62.3356	69.0704	65.9349	36.2655	37.734	1.3289	27.8569
AAEL004071	nitrogen fixation protein nifu	59.3959	59.8197	91.4835	83.8346	92.144	78.3664	211.83	117.785
AAEL011272	nmda receptor glutamate-binding chain	52.8189	52.5874	51.1235	54.5046	42.8372	43.405	346.946	169.031

AAEL004307	n-myc downstream regulated	48.0031	50.7247	49.6123	37.6885	51.2409	48.3838	129.923	67.5096
AAEL007158	nnp-1 protein (novel nuclear protein 1) (nop52)	29.4902	30.3506	31.3597	26.8034	47.6385	47.3356	6.42468	17.9268
AAEL003039	nonsense-mediated mrna decay protein	71.313	74.4176	55.2851	49.1156	301.052	329.127	55.826	63.9036
AAEL014051	nop14	57.8417	55.0664	74.6972	60.8989	62.3109	63.6867	3.20262	31.6726
AAEL012107	NOS	55.3667	53.7436	63.3981	70.3875	34.3938	40.5056	0.832016	22.6339
AAEL002039	NADPH oxidase(NOX5)	2.04523	2.14717	1.14439	1.26172	1.41199	0.81588	0.327787	2.24347
AAEL002733	npf	0	0.675118	0.418555	2.08795	1.61466	2.9626	721.949	311.782
AAEL006743	n-terminal acetyltransferase complex ard1 subunit	102.097	98.8779	117.718	108.684	112.25	109.27	80.0647	83.3806
AAEL003908	nuclear pore complex protein nup107	29.1566	29.2331	28.6992	34.828	18.534	20.8224	5.34379	17.4601
AAEL001861	nuclear pore complex protein nup154	33.3379	32.3584	40.088	31.482	32.0741	33.0139	6.16059	17.5727
AAEL004368	nucleolar RNA-associated protein	20.9339	20.7558	11.2703	15.1469	25.7252	26.1161	3.93443	11.1426
AAEL006710	nucleolysin tia-1	49.705	49.0144	42.2553	47.0202	43.4313	41.8973	6.03278	26.4469
AAEL005635	nucleoporin	37.0153	40.9592	36.2937	34.0754	41.3174	42.7049	12.8102	26.7123
AAEL002455	nucleoporin, Nup153, putative	37.9515	40.6332	49.3234	44.5563	39.1548	41.9237	12.1509	24.5658
AAEL006311	nucleoporin, putative	25.8083	25.2348	26.6246	24.339	22.4825	25.3148	14.1028	21.5424
AAEL012359	nucleoside-diphosphate kinase NBR-A, putative	1138.54	1281.13	1176.32	1019.57	1243.27	1366.9	774.149	919.535
AAEL005567	nucleosome assembly protein	19.232	22.4465	32.5055	20.2257	144.013	149.842	54.7188	35.9371
AAEL008143	nucleotide binding protein 2 (nbp 2)	12.9125	12.2925	6.95542	7.10861	11.1123	15.395	46.7643	20.2116
AAEL010360	nucleotide binding protein 2 (nbp 2)	4.78704	5.49271	3.31816	3.25353	7.22863	6.84306	39.5939	13.7575
AAEL009305	numb-associated kinase	11.1119	6.84039	16.7036	93.3576	13.4181	12.4012	39.5619	16.8014
AAEL002587	OBP11	67.4634	89.6221	35.6489	28.7266	68.084	68.7546	0.531701	13.9893
AAEL003315	OBP16	0.126386	0	4.73677	251.348	0	0	0.282208	0
AAEL004342	OBP18	62.4827	55.7448	6.80429	1.53996	34.1525	28.0992	1.0586	61.4537
AAEL005772	OBP22	929.738	851.179	2.88632	2.53915	13.7589	13.8333	28.5007	410.165
AAEL006393	OBP28	6.02268	4.68457	1370.45	8195.96	3.54569	3.99973	3.44732	4.15763
AAEL006387	OBP29	0	0.06833	4.32349	207.365	0.260635	0.113467	0.269031	0.100719
AAEL006396	OBP31	1.22378	1.0762	801.636	6180.29	1.03255	0.839342	1.11391	1.23735
AAEL006398	OBP32	0.465222	0.066982	208.635	1363.01	0.051792	0.226145	0.134911	0.203044
AAEL006385	OBP33	1.24619	0.677214	451.59	3755.38	0.525891	0.229338	0.548998	0.827667
AAEL009597	OBP40	0	0.085936	5.57775	91.8412	0.135692	0	0	0
AAEL009599	OBP41	0.819555	0.59375	623.98	3069.44	0.159819	0.818863	0.994055	1.39372
AAEL010718	OBP44	0	0	97.8061	264.077	0.19862	0.107922	0	0

AAEL010714	OBP45	0.35321	0.101166	512.997	1257.62	0.160078	0.174161	0.796733	0.747317
AAEL010872	OBP46	1.41833	1.06446	741.51	7483.99	0.874944	1.16696	2.56034	0.958491
AAEL011491	OBP52	0.960615	0.831825	3.06592	151.756	1.0775	1.63617	0	0.598592
AAEL000821	OBP6	0	0	0.058506	12.0017	0	0	0	0
AAEL000833	OBP7	0.260088	0.119742	39.4241	247.858	0	0	0	0
AAEL004783	Oda	406.507	383.413	639.508	576.038	205.12	221.475	1187.16	681.395
AAEL007450	odd skipped, putative	1.86167	1.95169	0.870364	0.921731	2.07685	1.48206	31.3097	9.68656
AAEL002626	Odorant-binding protein 56e, putative	1.66292	0.880666	502.32	2403.41	5.81298	6.15902	2.06198	1.02787
AAEL000839	O-fucosyltransferase, putative	31.1453	27.1668	35.0005	27.4662	19.8199	21.1768	6.5133	16.0215
AAEL003318	oligopeptide transporter	0.70568	0.755406	0.293984	0.384334	1.54801	1.95767	2852.03	1219.17
AAEL008381	oligopeptide transporter	62.1016	64.1908	15.7961	67.1546	32.4159	31.2004	1.18616	34.2956
AAEL008865	oligoribonuclease, mitochondrial	16.183	18.446	18.4121	16.4691	6.30384	8.94108	5.958	15.0884
AAEL004228	oligosaccharyl transferase	72.4394	76.0117	57.3452	52.8951	48.5444	48.9672	46.6986	57.5042
AAEL002804	oligosaccharyl transferase	61.589	85.8448	19.6967	18.9325	31.7474	32.3262	59.4612	57.2296
AAEL002183	oligosaccharyl transferase, subunit, putative	196.413	260.057	73.1311	64.9464	108.691	116.854	287.173	248.651
AAEL012141	Or43	9.41111	9.6182	12.022	12.6318	8.63188	9.63534	0.175176	3.15411
AAEL010339	orf 140; significant similarity to ORF next to beta-keto adipate succinyl CoA transferase from P. putida, putative	20.0189	19.9691	24.346	21.3524	21.66	21.499	14.5704	21.538
AAEL007693	organic anion transporter	1.26012	1.46476	2.4219	1.08904	2.43894	1.45028	78.1013	28.3973
AAEL007691	organic anion transporter	4.1844	4.66807	17.454	6.55258	7.64746	5.83083	150.855	62.6859
AAEL001808	organic anion transporter	33.1204	29.4126	35.2066	30.5167	12.1702	13.2593	4.20374	11.2282
AAEL012444	organic cation transporter	19.5238	16.4812	20.1309	16.9013	19.4541	21.0945	219.252	111.391
AAEL004328	origin recognition complex subunit	22.4915	24.9391	36.9877	35.0029	16.656	15.3297	1.6175	12.9943
AAEL010080	origin recognition complex subunit	31.8398	29.9466	67.9393	55.3259	23.7751	24.068	1.83126	15.2029
AAEL011358	origin recognition complex subunit	11.679	11.5852	12.7099	15.9483	7.38581	8.28622	0.784894	7.02755
AAEL005289	ornithine aminotransferase	48.2796	51.8851	66.971	54.8949	61.6898	55.2329	551.974	231.857
AAEL007878	ornithine decarboxylase	11.3725	12.7091	1.58096	1.48014	5.14313	4.45939	0.530119	5.09653
AAEL000044	ornithine decarboxylase	2.23024	3.95745	35.4802	38.9154	9.85664	11.3003	245.991	95.8519
AAEL007880	ornithine decarboxylase	232.883	180.268	125.778	114.687	245.306	330.626	3011.48	986.562
AAEL011309	orotidine-5'-phosphate decarboxylase, putative	55.2906	59.4353	68.4759	51.4409	169.822	161.325	37.1163	47.2609
AAEL004571	o-sialoglycoprotein endopeptidase	11.704	11.6691	15.1848	13.4944	15.7016	18.9487	1.24715	6.41155
AAEL000442	osk	698.809	780.898	979.768	882.422	301.426	324.736	10.094	405.088

AAEL002076	outer mitochondrial translocase subunit, putative	24.4716	28.292	32.3705	25.2477	43.2555	44.1149	64.4136	75.3775
AAEL007669	oxidoreductase	0.130537	0	0.283089	0.164187	0.301967	0.330068	83.3565	31.6761
AAEL010677	oxidoreductase	27.9582	23.8878	45.8384	44.8466	20.0335	24.0254	4.42361	14.1739
AAEL005852	oxysterol-binding protein 1, putative	57.0882	58.3203	55.6979	58.4477	41.2826	41.1801	303.177	162.502
AAEL001706	p15-2a protein, putative	79.5879	75.9436	99.7077	75.7158	74.1714	73.2227	32.4074	51.4528
AAEL008736	p31A, putative	112.661	114.83	145.819	131.468	107.727	112.108	53.7467	83.5012
AAEL001235	palmitoyl-protein thioesterase	8.72066	7.10397	13.1819	18.8856	7.19889	6.29533	75.5315	27.693
AAEL007151	paramyosin, putative	42.8784	39.7808	73.4614	49.0899	41.6272	38.6694	146.105	90.1475
AAEL000692	partner of sld5	25.7561	29.4415	34.2906	32.5697	16.0273	15.0933	6.94445	17.7293
AAEL010403	past-1	43.7499	46.4722	42.232	34.0315	52.4478	50.5779	189.246	77.9012
AAEL000339	paxillin, putative	23.7985	23.9203	35.2895	35.2758	31.423	30.5564	117.229	58.6528
AAEL003928	pdgf/vegf receptor	8.22012	7.37422	3.93364	3.52491	12.1221	10.0414	64.7043	27.3894
AAEL012280	pef protein with a long n-terminal hydrophobic domain (peflin)	24.6068	25.3633	27.611	23.0489	19.1006	17.8609	125.395	50.7983
AAEL013279	peptidyl-prolyl cis-trans isomerase (cyclophilin)	498.676	550.973	324.076	288.477	142.374	158.244	1032.61	743.342
AAEL011758	peptidyl-prolyl cis-trans isomerase f, ppif	721.669	765.706	756.384	654.111	740.34	767.404	782.296	696.705
AAEL006066	peptidyl-trna hydrolase	46.0471	53.4371	62.3357	50.3906	55.1591	56.9655	14.2334	28.3773
AAEL004861	peroxisomal integral membrane protein Per8p, putative	12.255	12.3124	18.8692	19.4152	17.2782	12.6199	0	7.58663
AAEL002913	peroxisomal membrane protein 70 abcd3	19.327	20.2346	19.6127	31.7091	23.7189	22.3027	178.933	88.2354
AAEL006491	peroxisomal targeting signal 2 receptor	27.9945	29.9387	27.4913	29.5888	35.4566	40.5992	12.8745	21.7527
AAEL000081	peroxisome assembly factor-2 (peroxisomal-type atpase 1)	51.0941	50.3831	71.9004	51.5547	77.5639	77.4478	28.1728	36.8915
AAEL010171	PGRPLB	0.555355	0.318166	1.12966	0.848477	3.58619	4.24024	472.865	100.586
AAEL011608	PGRPLD	39.4244	40.2241	74.3706	62.9444	37.9599	47.0603	5.57977	25.3584
AAEL009474	PGRPS1	17.5785	23.1403	7.24944	8.2743	35.5475	69.8153	1481.35	527.785
AAEL003032	phd finger protein	28.1337	25.5773	34.9764	33.4842	22.4437	24.9415	9.80854	17.8295
AAEL011124	phd finger protein	21.9575	21.5662	31.6689	30.934	17.1089	17.5943	1.88371	14.8424
AAEL002867	phenylalanyl-tRNA synthetase alpha chain	47.997	54.7602	30.5198	28.7102	59.2683	57.7293	23.8403	29.7611
AAEL003454	Phocein protein, putative	30.9076	29.2672	45.271	28.3414	20.5558	22.0891	35.0971	29.3954
AAEL000264	phopholipase d	8.61057	8.78867	16.1025	11.5041	15.2009	14.6705	100.364	44.6685
AAEL007019	phosphate transporter	153.78	159.424	125.137	119.008	186.454	183.531	109.219	114.855

AAEL007322	phosphatidate phosphatase	15.738	16.802	10.7789	20.8094	22.5365	16.5093	86.4744	42.8671
AAEL013170	phosphatidylcholine-sterol acyltransferase (lecithin-cholesterol acyltransferase)	103.285	109.378	30.7319	43.1158	40.7129	42.5961	19.5888	57.1026
AAEL011264	phosphatidylethanolamine-binding protein	16.7352	12.4827	12.3689	12.2506	10.3502	11.1986	375.573	173.872
AAEL011260	phosphatidylethanolamine-binding protein	0.179815	0.210601	152.984	75.3935	0.488774	0.442822	0.490237	0.374319
AAEL015262	phosphatidylethanolamine-binding protein, putative	0.890608	1.01677	533.19	289.593	1.13945	0.857679	2.24873	1.01561
AAEL015260	phosphatidylethanolamine-binding protein, putative	4.56871	3.47358	4.63373	5.01656	2.2424	3.19356	129.582	36.2567
AAEL013596	phosphatidylinositol 3-kinase regulatory subunit	31.9831	31.4804	11.4634	16.0795	28.0098	30.2388	10.8193	17.7555
AAEL013863	phosphatidylinositol-binding clathrin assembly protein	58.4137	62.006	54.9168	56.244	53.1997	49.5447	33.8618	43.5877
AAEL008393	phosphatidylserine synthase	24.051	18.8508	20.3682	38.0508	23.2136	23.1198	26.6229	20.3869
AAEL001472	phosphatidyltransferase	18.3287	19.2064	17.1854	19.435	15.2001	15.1335	2.55966	10.0417
AAEL006667	phosphatidyltransferase	21.0006	19.3962	4.70589	6.31474	19.9817	22.2305	83.5226	40.7016
AAEL000006	phosphoenolpyruvate carboxykinase	119.651	150.39	95.9946	67.3562	20.3706	23.4375	27.7679	148.308
AAEL000080	phosphoenolpyruvate carboxykinase	109.305	142.351	53.6267	46.8836	12.6575	20.3965	1286.37	562.732
AAEL001480	phosphoglucomutase	54.3254	54.6554	5.02677	8.10727	21.3773	22.7065	24.6693	35.1029
AAEL010369	phospholipase b, plb1	0.4285	0.551029	11.9852	33.6603	0.152883	0.273383	4.91036	3.30449
AAEL010661	phospholipid scramblase 1, putative	24.7504	29.3189	31.1921	32.6541	48.6376	46.2439	217.036	95.899
AAEL007677	phospholysine phosphohistidine inorganic pyrophosphate phosphatase	8.72001	9.05537	9.20558	8.62096	11.0534	10.3843	2.64778	10.4529
AAEL009497	phosphomannomutase	20.6136	35.6036	18.8905	15.5823	8.00095	9.45118	30.4432	24.0843
AAEL005384	phosphoribosylformylglycinamidine synthase, putative	39.5544	68.0387	22.1541	17.7982	40.1496	36.3107	43.4337	40.7494
AAEL001100	phosphoserine phosphatase	111.462	105.63	14.3454	9.59931	15.35	15.3783	2130.75	1220.34
AAEL010326	Phosphotriesterase-related protein	0.517238	0.174596	0.895983	0.715569	0.504877	0.690628	18.6846	4.52497
AAEL002415	pinch	16.2592	17.6764	20.3537	37.5831	16.9194	15.5214	35.8973	20.0153
AAEL007823	PIWI	54.4756	53.1369	45.8779	54.1393	68.481	67.3946	18.0078	32.9797
AAEL008076	PIWI1	16.8913	22.0863	5.43526	7.20044	29.4142	24.706	0.389168	7.8615
AAEL008098	PIWI2	11.5765	10.9183	10.2594	10.5799	25.8082	28.6992	0.117931	6.20421
AAEL013692	PIWI3	18.359	21.7979	4.30335	7.79984	23.2575	19.2209	0.118669	10.7216
AAEL007698	PIWI4	42.8019	36.6511	85.1713	71.7498	34.9862	34.008	14.8203	25.1894
AAEL013227	PIWI6	61.3582	57.0362	44.768	39.2823	49.2551	47.5314	20.503	40.9563

AAEL011662	plasma glutamate carboxypeptidase	0	0	0.123948	0	0	0.033054	198.407	109.652
AAEL011658	plasma glutamate carboxypeptidase	42.7304	44.9761	77.008	67.7125	42.3604	40.9572	17.2441	28.8269
AAEL011815	poly [adpbose] polymerase	25.1127	26.3374	23.0222	25.7179	28.0328	26.7359	12.157	19.3227
AAEL006249	poly(a) polymerase cid (pap) (caffein-induced death protein)	101.061	100.301	154.839	131.649	72.5627	70.9229	10.9556	49.7925
AAEL002550	polyA-binding protein interacting protein, putative	56.8396	56.3388	100.41	91.7077	41.6816	40.6807	33.5301	44.4558
AAEL010318	polyadenylate-binding protein	659.54	745.005	527.111	512.165	857.436	965.01	803.417	715.7
AAEL014098	polycomb protein	23.3322	22.9897	25.2988	28.1039	15.7731	17.1974	6.99673	14.8542
AAEL012568	polypeptide of 976 aa, putative	29.8109	29.5844	35.5804	36.4859	48.1169	44.6757	5.61195	18.591
AAEL004686	pont	21.8222	22.3133	31.4841	29.8842	20.3488	19.1164	8.67331	16.7315
AAEL004814	potassium-dependent sodium-calcium exchanger, putative	0.493794	0.286952	0.474563	0.355744	0.247295	0.320988	84.2317	34.6409
AAEL004821	potassium-dependent sodium-calcium exchanger, putative	18.9315	20.0257	14.3067	18.865	26.787	24.8354	0.295207	10.5088
AAEL004094	pou domain	10.4572	10.5159	13.6476	12.3915	8.79946	8.28258	0.117834	5.96466
AAEL011763	PPO3	18.2385	19.2507	0.8439	0.925506	4.1877	4.11761	2.50916	8.08382
AAEL013492	PPO5	92.8369	84.5286	0.239332	0.485359	1.76963	2.16931	13.081	49.6011
AAEL000851	predicted G-protein coupled receptor (GPCR)	2.15171	3.36176	3.31997	3.60899	8.9969	4.5863	92.1739	35.9037
AAEL006720	predicted protein	10.6377	10.8903	11.8945	11.1884	7.44133	6.51761	0.123952	8.10358
AAEL007751	predicted protein	87.2793	78.9235	134.776	136.172	64.7743	64.674	322.875	158.312
AAEL014511	predicted protein	44.1299	37.4346	64.4407	55.7601	37.2599	44.3947	26.6354	37.7136
AAEL004467	predicted protein	99.3317	100.488	134.78	101.475	64.6138	61.3223	57.7993	70.9642
AAEL000647	predicted protein	1.43077	0.517038	1.51875	1.45883	2.56082	5.13802	1497.82	709.36
AAEL001996	predicted protein	27.0846	26.9871	41.6445	37.1731	30.4313	29.8543	6.29801	18.5277
AAEL002580	predicted protein	20.6868	21.2936	15.3287	24.5715	84.8565	89.4552	0.995648	6.90524
AAEL004484	predicted protein	183.362	177.314	337.337	271.377	186.022	184.425	67.1322	131.357
AAEL009219	predicted protein	0.019456	0.041807	0.081013	0.047792	1.94241	1.48492	143.364	61.6031
AAEL003180	prefoldin subunit	58.3286	57.7668	45.2661	46.828	62.1629	58.4411	39.4909	49.8232
AAEL002337	prefoldin, subunit, putative	79.3092	86.2753	74.476	67.898	84.124	82.0553	47.4658	62.1348
AAEL009959	pre-mrna splicing factor prp8	38.8903	35.2527	33.1234	37.6244	27.0519	27.7714	20.6478	28.4871
AAEL004523	preprotein translocase secy subunit (sec61)	335.983	498.068	123.331	124.244	102.368	109.942	188.249	286.61

AAEL010716	preprotein translocase secy subunit (sec61)	43.559	74.0907	16.0745	15.7664	36.6735	36.1584	94.0614	75.6853
AAEL002196	procathepsin L3, putative	20.7485	8.72391	0.461364	13.4924	1.78467	1.09738	0.939187	4.20321
AAEL013353	profilin	292.541	305.022	515.484	337.136	208.376	211.911	712.517	423.415
AAEL011271	programmed cell death 6-interacting protein	36.3713	33.499	35.9305	33.4912	48.2631	43.055	110.19	57.7601
AAEL005910	programmed cell death protein	32.8178	32.3659	35.8687	31.5057	38.955	33.8455	139.713	63.4532
AAEL011803	prohibitin, putative	8.72274	6.84747	9.77885	13.8617	17.5365	18.8783	208.789	79.574
AAEL012545	proliferating cell nuclear antigen	166.203	161.17	205.918	213.576	53.2825	57.1303	28.9997	89.0635
AAEL012312	proliferation-associated 2g4 (pa2g4/ebp1)	147.039	157.811	35.8708	46.9281	144.929	133.145	61.3776	113.864
AAEL013431	proline oxidase	25.917	26.4594	35.4587	32.2357	23.9231	24.2772	1.63768	16.3186
AAEL012351	prolylcarboxypeptidase, putative	0.069499	0.122726	0.104587	0.121294	0.06229	0.337299	265.196	127.822
AAEL009038	prolylcarboxypeptidase, putative	87.9953	84.144	6.23165	7.27946	40.2144	36.2033	7.55372	46.7493
AAEL012561	prominin (prom) protein	14.5303	16.2182	27.4335	17.49	20.5684	20.0177	301.078	115.854
AAEL010725	proprotein convertase subtilisin/kexin type 4, furin	11.4523	11.2378	20.8779	64.2097	7.13736	6.99616	7.16065	8.1339
AAEL012099	protease m1 zinc metalloprotease	6.23376	5.80158	3.82266	3.68396	6.04979	4.36897	104.896	38.0573
AAEL003212	protease m1 zinc metalloprotease	0.046605	0	0.016944	0.039338	0.256768	0.162692	100.327	31.257
AAEL013899	protease m1 zinc metalloprotease	0.206535	0.160089	0.55257	0.155395	0.610265	0.800558	676.217	346.024
AAEL008155	protease m1 zinc metalloprotease	0.01727	0.009854	0.136118	0.064893	0.490842	0.442605	141.68	65.72
AAEL012778	protease m1 zinc metalloprotease	0.274658	0.296106	0.471389	0.4258	3.42181	3.73185	880.061	453.529
AAEL012217	protease m1 zinc metalloprotease	53.0158	52.058	118.498	109.605	44.6278	43.8743	1.14098	25.9634
AAEL012783	protease m1 zinc metalloprotease	2.03101	0.789959	0.474782	0.451966	2.38835	3.62551	1914.78	1100.17
AAEL012776	protease m1 zinc metalloprotease	0.356933	0.415662	0.405362	0.179858	1.00459	1.51362	1109.83	533.812
AAEL012781	protease m1 zinc metalloprotease	1.32079	1.41484	1.38174	2.4322	1.53283	1.54064	147.246	78.0077
AAEL014671	protease S51 alpha-aspartyl dipeptidase	14.8724	12.2407	9.35929	12.8526	6.47941	6.29104	167.484	60.0119
AAEL015036	protease S51 alpha-aspartyl dipeptidase	12.672	14.5154	9.06885	10.993	7.39183	7.79043	484.046	244.882
AAEL009898	proteasome subunit alpha type	68.0213	67.1989	50.2106	50.8902	54.2304	58.3757	152.636	84.4998
AAEL010829	protein arginine n-methyltransferase	11.6876	13.4146	16.6036	13.6685	12.522	13.6992	2.16549	6.84232
AAEL003179	protein arginine n-methyltransferase 1, putative	162.05	161.302	330.592	265.976	200.434	199.589	24.6642	85.4206
AAEL008319	protein disulfide isomerase	72.3882	68.6256	76.9024	73.844	49.4231	50.9544	55.5152	62.0545
AAEL000641	protein disulfide isomerase	596.977	895.167	155.552	190.747	243.05	250.177	625.053	653.309
AAEL001432	protein disulfide isomerase	408.352	466.619	205.196	213.661	189.436	191.788	252.377	317.437
AAEL010065	protein disulfide-isomerase A6 precursor	115.649	158.174	82.3817	79.5993	68.3382	91.2913	234.714	164.305

AAEL000421	protein farnesyltransferase alpha subunit/rab geranylgeranyl transferase alpha subunit	21.8601	23.3892	42.6328	37.5289	18.5969	20.4611	2.07717	14.0362
AAEL001108	protein kinase c	16.7702	17.639	15.6285	17.6677	20.58	17.669	0.575406	6.44192
AAEL002722	protein kinase C inhibitor, putative	15.159	18.324	22.6687	41.242	23.3788	25.2446	25.2867	44.6447
AAEL005131	protein kinase c-binding protein nell1	10.9079	10.5809	0.266029	0.343692	1.28494	1.39144	0.079054	5.86578
AAEL007634	Protein KRTCAP2 homolog	74.4477	94.8854	39.7402	34.6016	27.2502	29.515	46.012	54.0186
AAEL008595	Protein maelstrom homolog	162.703	160.112	66.4626	98.4425	155.437	160.636	2.24519	84.4514
AAEL004410	protein phosphatase	10.926	10.1361	11.1496	11.1929	11.5133	11.2829	0.991623	4.78761
AAEL007171	protein phosphatase 2c	34.4976	31.9704	53.0941	47.5894	26.6321	27.7058	1.22802	18.6753
AAEL001441	protein phosphatase 2c gamma	105.804	101.085	153.886	148.201	89.9162	88.9067	39.6032	64.078
AAEL014042	protein phosphatase pp2a regulatory subunit b	54.9086	68.5062	62.8517	61.4903	64.507	49.4772	69.6253	79.6806
AAEL004709	protein phosphatase type 2c	46.2861	46.1227	23.3133	26.7413	26.0199	27.0959	357.74	188.386
AAEL004262	protein phosphatases pp1 regulatory subunit	7.055	8.26379	20.0271	18.0878	7.10739	6.55975	0.557172	5.05951
AAEL004040	protein phsophatase-2a	75.9303	78.1186	116.867	91.1769	57.1229	58.3659	37.5521	52.8962
AAEL008863	protein regulator of cytokinesis 1 prc1	27.44	26.1218	66.7297	75.1692	13.4991	15.097	3.41841	16.9451
AAEL001969	protein serine/threonine kinase, putative	11.019	11.3755	4.91408	4.44303	8.85416	20.5126	29.5262	23.1141
AAEL012659	protein serine/threonine kinase, putative	24.7943	22.552	76.6662	53.1612	23.2739	18.7591	0.691132	17.0073
AAEL013989	protein translocation complex beta subunit, putative	137.742	234.644	44.9954	42.0559	65.2612	70.405	126.47	159.693
AAEL008777	proto-oncogene tyrosine-protein kinase abl1	22.8291	22.2556	19.5154	23.7174	22.253	22.5107	3.63799	11.8301
AAEL004663	pseudouridylate synthase	18.5483	17.3234	27.0913	23.3849	25.2543	23.182	5.4695	11.0188
AAEL004142	psidin	21.6925	21.3209	24.319	23.0305	29.2899	28.7405	5.38985	14.0199
AAEL013733	Psq-DNA binding domain protein, putative	21.227	21.2643	29.0074	25.8946	18.3785	18.903	0.248356	11.9838
AAEL005065	ptpla domain protein	25.118	29.0165	11.8997	18.4474	16.965	20.3985	19.1037	33.512
AAEL008391	ptx1 protein	20.9295	20.3193	19.2037	20.0904	14.4088	15.5918	3.77779	9.28736
AAEL001735	pupal cuticle protein 78E, putative	6.46767	7.68858	3.42074	5.76499	0.914828	2.18756	0	8.16791
AAEL013515	Pupal cuticle protein, putative	204.404	208.77	169.639	167.387	421.189	406.043	0.560392	54.0922
AAEL003606	purine biosynthesis protein 6, pur6	89.7708	158.506	74.832	45.418	76.6978	61.5164	30.3147	70.757
AAEL002269	purine nucleoside phosphorylase	137.139	213.455	56.1998	37.446	84.2747	79.9805	110.352	148.695
AAEL009601	pyridoxine kinase	39.5338	33.6299	44.1468	39.5152	141.586	120.568	697.165	343.327
AAEL005422	pyrroline-5-carboxylate dehydrogenase	32.2065	33.3697	36.7783	36.5923	19.9222	21.9496	12.347	20.4513

AAEL013831	pyrroline-5-carboxylate dehydrogenase	23.9192	21.6801	26.1908	25.3422	15.6219	13.7012	7.06648	15.1694
AAEL007033	pyrroline-5-carboxylate reductase	33.6081	34.3918	22.9034	29.6	51.4619	45.6195	148.499	76.7199
AAEL008366	pyruvate dehydrogenase	23.7188	26.9762	23.6613	23.6045	26.6456	24.9704	12.1968	21.0273
AAEL007375	pyruvate dehydrogenase	95.3412	96.8935	68.7471	55.3	59.8703	60.3944	412.813	249.429
AAEL014913	pyruvate kinase	13.5188	8.88456	3.65212	7.42955	3.04459	6.36953	32.6721	10.6066
AAEL000016	Q0C7C4	31.8054	30.2701	32.1556	27.1342	43.0571	41.6531	9.63018	22.4662
AAEL014561	Q16G12	12082.6	9396.17	4.01591	477.779	2.42096	2.75949	84.4884	3881.73
AAEL013027	Q16KE2	9290.84	5539.29	10.9921	1416.74	1.53095	1.67082	90.6437	4641.81
AAEL010993	queuine tRNAbosyltransferase	9.65076	8.82629	4.98202	6.0719	13.731	14.2881	1.47586	5.57029
AAEL012054	Quiescin-sulfhydryl oxidase4, putative	86.9949	105.094	220.652	39.8836	60.0159	56.1091	55.6799	71.8545
AAEL007845	rab5	106.089	103.886	55.3105	73.1331	84.0487	92.5622	253.293	141.476
AAEL009495	rab6-interacting	24.4099	23.668	32.0761	33.0438	21.0611	19.9405	7.60247	15.8036
AAEL007176	rabkinesin-6	39.716	40.5618	47.078	46.9089	28.5445	28.3918	1.57757	23.3807
AAEL009732	rac gtpase	55.9996	52.4055	51.3437	49.6889	36.0522	36.7557	163.06	86.1069
AAEL010342	racgtpase-activating protein	14.4981	14.3943	21.1098	21.2516	12.3339	14.1141	1.67129	8.11556
AAEL013069	Rack1	1015.16	1035.07	388.098	348.684	3088.58	3277.16	1413.84	1330.24
AAEL011307	RAD51C protein, putative	4.59649	3.69034	8.88606	10.7323	2.65961	1.99347	0.610999	4.37059
AAEL012151	ral guanine nucleotide exchange factor with ph domain and sh3 binding motif, ralgps	32.0584	33.5628	48.1196	41.8698	30.7401	30.9263	10.4899	21.5163
AAEL009287	ran	247.275	271.319	187.249	178.366	288.034	285.903	206.222	197.342
AAEL007858	ran gtpase-activating protein	19.0639	21.2049	26.5305	22.1334	24.1923	23.2481	6.69846	12.8204
AAEL009884	ran-binding protein	38.1954	35.8988	32.9092	32.7602	25.6478	26.6683	12.9104	23.5637
AAEL005998	rap gtpase-activating protein	23.717	28.2391	30.1808	28.4883	24.9615	24.5606	11.1809	19.7122
AAEL011870	rap55	451.612	428.47	534.086	496.92	261.84	256.565	51.7791	251.443
AAEL009377	RAS protein, putative	78.3499	92.7455	133.18	101.027	62.8125	63.0627	79.3096	77.2968
AAEL005069	raslated protein Rab-1A, putative	164.316	175.764	149.565	148.139	119.362	119.607	350.968	207.073
AAEL004902	raslated protein Rab-2A, putative	36.7768	39.5393	57.3618	54.4377	38.0524	37.3285	148.948	81.986
AAEL008029	raslated protein, putative	16.6746	13.3521	13.946	13.3506	10.3017	11.1326	0	7.69062
AAEL011892	receptor for activated C kinase, putative	50.6207	45.8913	182.427	120.444	57.5213	61.332	23.8788	36.0548
AAEL002404	receptor protein tyrosine kinase	242.678	315.534	79.2679	92.2494	73.3717	84.0064	194.454	220.064
AAEL007123	receptor tyrosine kinase	14.0088	11.7714	12.1611	14.7828	16.2779	16.5074	0.534945	4.36004
AAEL001623	REG	111.601	115.802	130.379	115.643	163.285	176.493	88.7507	98.2036
AAEL008575	regulator of chromosome condensation	74.5934	73.421	121.932	118.323	22.3349	23.4509	34.2322	57.3172
AAEL007799	regulator of chromosome condensation	237.445	256.888	151.166	151.606	151.792	152.417	32.4843	140.592

AAEL007624	REL2	4.58942	4.29575	4.65917	4.42451	20.6604	12.6199	43.2877	18.0231
AAEL007291	replication factor A, 14kD-subunit, putative	55.564	59.9672	74.2933	71.0677	49.6707	48.1208	16.2503	36.9185
AAEL009465	replication factor c / DNA polymerase iii gamma-tau subunit	33.0973	32.4517	35.0297	38.0444	31.8608	28.6538	3.08687	17.5658
AAEL001324	replication factor C large subunit, putative	21.293	21.6289	22.3588	22.507	21.1191	19.7507	3.98026	12.5058
AAEL006788	replication factor C, 37-kDa subunit, putative	26.9307	25.1135	20.2267	23.9369	19.5027	21.0132	2.67218	14.0783
AAEL006223	replication factor C, 37kD-subunit, putative	29.3361	26.5514	27.4447	28.35	16.6791	17.7016	10.54	20.1761
AAEL010422	replication-associated histone mRNA stem loop-binding protein, putative	61.7623	60.8013	68.0697	65.4396	38.4281	36.5048	3.48337	29.8934
AAEL011279	requim, req/dpf2	35.201	30.0342	60.2677	47.6513	17.7973	19.2166	7.13895	21.4696
AAEL001126	rest corepressor (corest) protein	68.6966	72.234	81.0613	70.7982	69.325	71.7581	42.8955	58.2889
AAEL007725	reticulocalbin	30.6594	34.1348	12.2101	10.4143	13.6931	14.6074	11.6098	19.8472
AAEL000713	reticulon/nogo	129.478	220.722	78.7689	77.4784	50.3926	50.4208	52.3751	102.201
AAEL006045	reticulon/nogo receptor	3.27866	6.67477	5.60071	5.12541	2.16263	0.239516	0	6.79266
AAEL010910	retina aberrant in pattern, putative	14.9765	13.9757	34.4316	34.3754	12.6761	14.5754	0.155197	7.81963
AAEL009882	retinoblastoma-binding protein 4 (rbbp4)	122.274	109.915	230.098	221.595	53.4058	55.2203	31.5457	74.8273
AAEL007926	retinoid-inducible serine carboxypeptidase (serine carboxypeptidase	0.669591	0.568895	0.2233	0.577783	2.81953	2.10541	1478.66	614.652
AAEL009291	retinoid-inducible serine carboxypeptidase (serine carboxypeptidase	201.057	217.564	33.1182	27.8357	77.7904	84.5459	56.7802	129.037
AAEL006542	retinoid-inducible serine carboxypeptidase (serine carboxypeptidase	404.942	528.493	6.5766	7.51211	8.20055	7.34788	23.1681	223.633
AAEL007581	Rfc5p, putative	17.7944	18.9624	24.2515	21.7152	12.9044	15.3783	1.18169	9.72095
AAEL013752	rfx5	13.9026	13.6045	8.24337	9.49413	13.891	13.7751	0.197942	7.23318
AAEL012658	rgs-gaip interacting protein gipc	13.7904	11.0249	4.10177	7.0012	2.015	2.16349	209.789	90.4079
AAEL008881	RHC18, putative	25.4099	25.9585	17.8374	21.0513	12.2375	13.6816	12.7744	21.6654
AAEL000509	rho gtpase activating protein	14.61	14.8359	25.5227	16.0095	12.4123	12.1024	2.91346	8.86121
AAEL009157	RHO GTPase activator, putative	21.5828	21.0758	18.5142	20.4228	19.9817	20.4982	7.09168	13.4201
AAEL012996	rho guanine dissociation factor	69.5959	68.9125	73.5304	63.5868	72.3169	71.0818	274.232	147.918
AAEL013487	RHO guanyl-nucleotide exchange factor, putative	26.5515	25.0717	42.3986	34.2502	31.0306	30.6551	6.34714	16.7185

AAEL003587	rhogtpase	12.3754	12.7818	4.78363	5.78016	10.3716	9.78907	75.1653	37.0059
AAEL003510	ribonuclease hi large subunit	40.4358	41.6984	51.2634	54.5387	30.4374	30.903	4.08411	18.5214
AAEL004119	ribonuclease p/mrp subunit	8.26659	7.72332	14.2586	12.8345	4.72361	3.9085	117.217	48.9611
AAEL003716	ribonuclease UK114, putative	83.1737	111.078	46.2743	48.3834	102.992	101.413	2.10631	32.3535
AAEL003492	ribonuclease, putative	98.7161	94.9198	76.2168	68.281	102.681	114.892	1019.28	574.563
AAEL011089	ribonucleoprotein	75.9156	76.5705	64.4807	77.7667	57.0555	53.1044	30.6711	48.7649
AAEL010644	ribonucleoside-diphosphate reductase large chain	69.1678	59.2798	296.306	239.063	15.9271	14.8554	20.8078	48.486
AAEL010691	ribonucleoside-diphosphate reductase small chain	273.238	235.954	443.156	232.561	45.8988	42.4291	70.42	171.236
AAEL013071	ribophorin	142.458	183.787	48.7417	50.3612	77.0917	75.9478	124.611	134.938
AAEL015572	ribophorin ii	47.8034	61.6965	19.4604	17.4981	26.9717	27.023	36.1474	48.5316
AAEL010521	ribophorin ii	52.1583	74.6475	24.2228	21.6024	27.0754	28.9048	46.6459	44.5565
AAEL014449	ribose-phosphate pyrophosphokinase 1, putative	25.8975	36.0159	19.9867	16.424	34.4476	28.8324	4.30765	17.4824
AAEL004178	ribose-phosphate pyrophosphokinase 1, putative	242.884	295.961	164.387	115.758	297.134	255.552	48.9379	162.049
AAEL001037	ribosomal RNA methyltransferase	17.3927	15.5793	16.0802	15.9525	35.7154	32.9535	5.00775	13.4212
AAEL011282	ribosomal RNA small subunit methyltransferase b (sun)	47.5271	50.6309	57.3621	45.045	62.1787	61.642	8.66721	28.5413
AAEL003555	ribosome biogenesis protein	18.6912	18.9336	24.6113	21.6574	21.3154	22.0083	3.47774	9.85191
AAEL001169	ribosome biogenesis protein bop1 (block of proliferation 1 protein)	19.681	20.966	22.2384	17.2591	35.7749	37.4797	6.2493	12.8913
AAEL012185	ribosome biogenesis regulatory protein	37.9015	40.0198	51.3424	44.9975	33.3863	32.3093	8.84275	26.0331
AAEL010505	ribulose-5-phosphate-3-epimerase	34.1549	37.0179	52.843	39.839	32.6474	36.2307	34.9148	41.6126
AAEL004691	ring finger	215.144	208.03	308.255	275.569	210.627	221.426	223.277	190.526
AAEL006839	ring finger protein	29.9654	29.787	48.0742	40.9924	21.3499	23.2005	15.5262	24.3211
AAEL005114	RNA and export factor binding protein	75.0715	79.2828	31.7604	45.0252	42.5413	45.4745	35.7731	60.1391
AAEL011251	RNA binding motif protein	29.1789	26.7537	34.4876	29.5119	22.6673	22.8659	11.4005	19.1046
AAEL013982	RNA binding motif protein 4,lark	108.866	114.061	50.8256	62.591	48.8166	50.0208	58.726	82.5552
AAEL000674	RNA m5u methyltransferase	21.7638	23.2526	21.4056	20.599	27.13	30.4302	4.56057	13.5853
AAEL014831	RNA polymerase II subunit Rpb10, putative	38.8354	44.5954	66.1164	55.9718	51.578	54.3718	34.5778	43.6491
AAEL013457	RNA polymerase small Zn-binding subunit, putative	109.412	120.765	90.7703	101.399	95.9254	123.463	50.0045	59.3839
AAEL012171	RNA recognition motif protein split ends	66.1224	61.7269	25.3075	63.0207	38.501	47.1039	31.6329	51.8837

AAEL013869	RNA-binding protein	35.5555	35.0308	33.6834	35.2812	55.6039	54.0985	14.7455	25.675
AAEL007013	RNA-binding protein precursor, putative	14.8478	13.4328	22.0369	23.9339	15.8771	16.4004	0.58784	6.09954
AAEL008572	RNA-binding protein, putative	12.3419	13.0788	12.9573	13.9754	16.0012	15.2782	2.36189	8.53336
AAEL003572	rnase h (70)	14.5352	14.8236	4.66057	7.50618	18.5964	16.3242	4.13494	9.58467
AAEL003382	Ro ribonucleoprotein autoantigen, putative	76.248	71.9883	35.7682	56.1331	54.0858	49.1142	3.08403	35.5189
AAEL010904	rothmund-thomson syndrome DNA helicase recq4	11.6305	10.0625	21.0192	18.8094	7.37844	7.52339	0.660897	5.6715
AAEL002534	RpL10	1725.89	1849.92	771.133	649.591	5300.84	5204.66	3673.87	3042.1
AAEL013221	RpL10a	1174.95	1244.29	477.916	430.419	3276.01	3250.24	1950.3	1565.37
AAEL012944	RpL11	1358.41	1410.29	652.881	567.603	4053.3	4010.05	2147.93	1802.11
AAEL014562	RpL12	836.372	912.33	396.867	338.76	2213.92	2118.21	1561.31	1178.98
AAEL009825	RpL13a	1507.78	1593.38	597.5	538.429	3756.5	3862.94	2495.41	2135.67
AAEL011447	RpL14	1696.36	1773.9	695.299	643.591	4500.42	4575.38	2685.87	2376.12
AAEL012736	RpL15	1142.37	1177.61	408.098	421.752	3015.72	3004.56	1900.68	1615.91
AAEL008481	RpL18	1415.41	1476.73	637.974	570.704	3789.06	3723.89	2772.09	1953.47
AAEL006785	RpL18a	1599.28	1663.93	686.185	596.674	4141.28	4175.21	2999.61	2453.04
AAEL012733	RpL19	697.567	776.023	375.516	353.033	1815.55	1781.67	1411.92	1095.56
AAEL007715	RpL21	1207.91	1229.68	474.818	461.479	2878.35	3016.44	2081.7	1735.21
AAEL007771	RpL22	2248.19	2370.73	631.699	773.031	4953.09	5053.43	4488.05	3244.7
AAEL013097	RpL23	704.232	731.627	374.975	312.261	2342.49	1966.46	1389.19	1166.95
AAEL015006	RpL23	108.868	114.26	41.7977	53.6783	170.825	372.301	255.343	107.59
AAEL008329	RpL24	1279.4	1314.83	598.836	560.242	3462.53	3469.62	1879.79	1720.35
AAEL012681	RpL24	168.733	165.102	161.806	132.566	625.284	605.743	263.77	200.367
AAEL005722	RpL26	1055.92	1103.73	524.298	476.621	3384.02	3396	2017.31	1605.64
AAEL005817	RpL26	1434.68	1532.17	649.176	606.803	4206.26	4235.68	2527.38	2078.65
AAEL011587	RpL27	22.108	11.9965	31.5281	16.3742	59.3172	55.5501	60.7358	11.3443
AAEL008353	RpL28	1505.57	1587.72	775.091	675.612	3605.22	3681.79	2630.07	2044.39
AAEL005085	RpL30	332.967	353.882	147.134	138.199	813.098	849.967	526.141	501.188
AAEL005220	RpL30	28.1158	30.6194	6.78442	8.22304	81.2801	68.9443	58.0286	15.2009
AAEL006698	RpL31	1712.17	1737.12	688.839	685.992	3937.55	3776.95	3226.49	2471.42
AAEL003396	RpL32	1463.01	1546.03	624.177	577.377	3574.73	3528.62	2497.42	1977.58
AAEL009341	RpL34	1192.36	1219.23	551.784	474.469	3331.93	3352.5	1999.22	1818.22
AAEL000823	Rpl35	1398.51	1384.4	609.695	560.199	3613.17	3577.45	2302.91	1993.74
AAEL000010	Rpl36-1	1391.08	1402.17	732.526	700.181	3263.91	3108.21	2497.61	1706.84
AAEL002639	Rpl36-2	50.1601	67.9647	38.065	39.8869	105.554	138.939	84.0186	112.885

AAEL005451	RpL38	2296.97	2500.32	1063.4	1003.51	5927.66	5892.95	4298.29	3308.15
AAEL000701	Rpl4	23.7354	26.7091	28.0591	20.8005	33.0913	33.3254	16.4686	22.898
AAEL009994	RpL4	968.814	1024.35	409.891	367.755	3125.03	2959.61	1952.14	1395.37
AAEL003942	RpL44	1276.19	1297.64	466.981	448.732	3909.86	3968.95	2258.92	1819.31
AAEL008188	RpL6	1462.56	1550.76	593.135	566.355	3763.84	3682.03	2844.44	2059.92
AAEL012585	RpL7	977.015	1017.49	504.382	439.075	2937.41	2930.57	1622.87	1361.38
AAEL005629	RpL7a	1083.37	1108.15	475.614	443.143	2725.92	2745.57	1955.22	1520.35
AAEL003352	RpL7ae	244.14	278.323	343.195	295.076	416.798	434.381	35.0284	159.791
AAEL000987	RpL8	1363.24	1506.24	511.134	489.952	4050.87	4133.13	2018.63	1754.25
AAEL007699	RpL9	1355.92	1410.73	574.067	540.224	3690.03	3615.45	2542.72	2005.65
AAEL010821	RpLP0	825.681	859.013	324.835	327.061	2283.17	2425.42	1077.19	1027.64
AAEL003530	RpLP1	1361.43	1464.61	595.805	543.788	3155.87	3328.25	1775.99	1749.26
AAEL014583	RpLP2	3761.47	3909.42	859.075	1035.29	9032.27	9094.61	4975.28	4683.18
AAEL002047	RpS10	1207.55	1223.78	550.635	498.32	3082.05	3081.37	2258.65	1825.8
AAEL002372	RpS11	1632.37	1716.88	779.981	728.773	4079.29	3996.54	3020.17	2345.6
AAEL010299	RpS12	1466.55	1490.48	571.161	569.662	3941.63	3958.98	2724.43	2166.34
AAEL003582	RpS13	914.724	1029.81	428.726	402.317	2263.42	2275.55	1716.81	1354.63
AAEL005266	RpS14	1268.35	1260.08	512.002	456.344	3483.83	3459.41	2915.16	2021.39
AAEL011656	RpS15	1846.99	1861.91	777.811	739.458	4895.32	4978.98	3228.49	2524.15
AAEL003427	RpS16	1644.91	1725.29	785.48	747.733	4391.75	4365.24	3483.83	2639.24
AAEL004175	RpS17	1344.2	1388.87	657.329	584.927	2998.55	2963.68	2341.01	1859.55
AAEL010756	RpS19	1835.5	1880.66	911.639	842.976	4696.55	4635.23	3458.43	2541.34
AAEL008582	RpS2	36.3681	39.6466	44.8725	38.7387	52.3805	50.3542	17.8366	25.803
AAEL010168	RpS2	641.105	682.014	302.862	294.353	1811.36	2089.21	834.586	779.376
AAEL009506	RpS20	1024.99	1112.51	617.158	576.602	2682.18	2870.85	1472.47	1369.95
AAEL004503	RpS21	2417.4	2573.87	1012.66	993.733	5593.95	5592.29	4644.32	3794.44
AAEL012686	RpS23	1159.2	1239.3	633.032	565.944	2846.15	2833.41	2311.76	1846.96
AAEL014292	RpS24	224.84	200.253	109.657	81.5982	556.727	634.095	361.62	368.992
AAEL014903	RpS24	601.174	703.713	234.495	253.673	1438.04	1386.49	1095.34	762.147
AAEL000068	Rps25	1107.26	1065.3	446.594	477.93	3088.69	3179.96	2166.17	1710.63
AAEL010573	RpS25	203.851	283.638	84.8787	69.8515	523.44	431.188	341.29	232.522
AAEL002832	RpS26	1518.12	1601.72	668.136	702.48	3582.75	3643.67	2408.18	1988.24
AAEL006860	RpS28	1074.05	1142.49	521.462	516.76	2511.29	2555.42	2025.85	1599.77
AAEL007824	RpS29	2625.53	2832.45	1309.48	1240.07	5142.37	5455.14	5200.21	4280.85
AAEL008192	RpS3	999.127	1040.17	411.705	378.882	2880.79	2863.4	1581.72	1251.35

AAEL009653	RpS30	1055.16	1099.15	521.122	467.173	2584.23	2581.19	2199.81	1659.37
AAEL013158	RpS3a	252.062	284.61	108.306	105.494	708.584	737.918	372.81	318.411
AAEL005901	RpS3a	970.496	967.461	404.826	345.051	2647.35	2486.47	1332.55	1186.95
AAEL013625	RpS5	1229.45	1230.77	526.086	502.247	3038.09	3006.08	2104.15	1801.37
AAEL000032	RpS6	720.992	757.658	309.064	302.258	2199.83	2081.76	1445.02	1010.1
AAEL009151	RpS8	1527.02	1636.1	632.124	608.77	3530.39	3551.38	2464.45	2021.06
AAEL008103	RpS8	1394.88	1437.97	472.57	430.963	4088.21	4063.45	2217.79	1777.69
AAEL001759	RpS9	1093.73	1197.49	595.424	565.591	3396.69	3485.49	1811.67	1553.36
AAEL013694	RpSA	271.602	299.315	126.215	90.4488	916.21	931.768	356.446	352.732
AAEL009061	rrm/rnp domain	47.4265	46.923	32.8298	49.2001	22.1809	20.0728	21.0616	29.3208
AAEL012874	rrp4	14.6001	12.9871	13.6992	14.0702	20.1542	19.0769	3.94398	10.1176
AAEL004243	S1 RNA binding domain protein, putative	14.8321	14.5294	12.7377	12.821	14.4843	15.7966	2.70894	8.45694
AAEL014734	saccharopine dehydrogenase	8.30972	14.1378	3.9515	0.515736	1.82497	2.2331	275.943	166.668
AAEL001176	s-adenosylmethionine decarboxylase	104.405	116.325	53.8737	50.9571	120.726	102.991	500.062	290.298
AAEL013129	salivary gland secretion, putative	1.6899	1.24785	577.456	3765.93	0.445661	0.952463	0.538876	1.37993
AAEL000149	sap30	61.9912	60.0124	74.78	73.0411	49.2298	51.7687	13.3782	41.1382
AAEL003046	saposin	284.439	248.642	136.208	153.681	217.394	203.369	698.687	395.638
AAEL010028	sarcosine dehydrogenase	47.1066	55.8244	7.86217	5.80693	13.5201	13.1372	1.57964	34.0995
AAEL014936	sarcosine dehydrogenase	26.9058	46.5273	8.1207	4.4951	8.6297	7.92158	0.903247	10.5807
AAEL014931	sarm1	7.04721	6.27644	10.1874	6.84824	23.4681	17.5483	66.8734	25.5646
AAEL009192	SCRASP1	17.777	16.8872	19.2361	22.7402	35.104	39.0331	0.745117	7.76252
AAEL009420	SCRBQ1	2.27818	2.3072	3.03469	2.82686	4.69859	3.20737	46.6039	20.6352
AAEL009423	SCRBQ2	33.5173	31.125	26.3829	28.6985	26.5387	25.1925	149.129	59.1912
AAEL009432	SCRBQ3	0.279239	0.395811	0.185322	0.091817	0.519512	0.853334	461.449	208.451
AAEL001307	SEC14, putative	412.146	386.766	1.26448	1.55288	5.22352	1.90758	103.258	218.826
AAEL008271	SEC14, putative	0.063781	0	0	0	0.231406	0.626504	190.553	108.617
AAEL010344	SEC14, putative	78.401	52.7348	1.10837	6.09346	4.43408	4.24482	3.1932	34.2433
AAEL005471	Sec61 protein complex gamma subunit, putative	456.827	636.727	168.793	184.126	159.482	176.211	450.413	492.641
AAEL002158	secreted ferritin G subunit precursor, putative	209.774	184.874	392.876	368.558	209.765	218.257	907.494	484.864
AAEL004335	secreted ferritin G subunit precursor, putative	7.54104	9.24355	1.0464	0.752892	0.9246	1.20469	305.452	124.75
AAEL007383	secreted ferritin G subunit precursor, putative	310.554	375.127	326.672	318.239	371.269	387.496	16059.6	7356.27

AAEL000090	secretory carrier-associated membrane protein (scamp)	49.0012	49.6046	55.6941	47.6938	49.4771	48.5844	271.493	135.475
AAEL001937	selenium-binding protein	23.5332	21.2935	7.40883	7.96341	20.1877	17.5505	144.563	72.2927
AAEL007113	selenophosphate synthase	45.2019	66.4582	24.0801	20.7989	54.2831	50.3399	320.61	185.59
AAEL008849	selenophosphate synthase	218.866	232.871	322.558	271.912	85.1328	81.3351	231.85	207.637
AAEL005523	Selenoprotein G, putative	55.86	70.2792	54.8854	59.7851	35.412	34.3192	160.304	89.6632
AAEL008952	sentrin/sumo-specific protease	38.7313	39.4971	54.1737	51.5367	40.9705	40.5985	13.5583	27.6978
AAEL001372	sentrin/sumo-specific protease senp7	54.3465	56.2662	70.9811	65.7603	50.4498	48.1817	23.0656	35.7695
AAEL005493	septin	51.6779	51.5903	70.6053	64.6811	44.6627	44.0529	13.1346	26.2349
AAEL007432	serine collagenase 1 precursor, putative	3.73197	2.20515	0.587295	1.05913	1.18195	1.35285	5474.64	3665.14
AAEL002510	serine hydroxymethyltransferase	102.466	149.523	72.2251	52.8486	140.98	140.921	19.9531	81.3765
AAEL000706	serine palmitoyltransferase	45.1478	45.2223	58.4055	60.544	54.7795	52.7369	117.903	58.8016
AAEL002600	serine protease	56.5899	43.4128	61.0943	52.1192	216.07	196.99	5.44131	14.4909
AAEL007969	serine protease	4.44386	4.95497	4.01906	3.79445	23.6628	11.6293	0.822483	2.0366
AAEL010867	serine protease	24.862	28.4453	41.4619	36.6508	80.4763	84.1247	10.0216	16.5361
AAEL006586	serine protease	4.9837	1.59273	31.9088	71.0854	13.8059	10.7288	0.700185	0.620734
AAEL006568	serine protease	68.0579	48.3432	28.2067	56.5499	55.8898	41.2645	1.20052	12.8344
AAEL002593	serine protease	45.5838	40.8605	79.4487	78.2747	127.187	147.03	2.45784	22.1451
AAEL002610	serine protease	46.2829	21.3018	17.1647	18.0638	328.886	166.656	2.97038	6.82088
AAEL010866	serine protease	18.1086	17.4042	11.7516	11.4238	19.4009	19.7255	1.25589	7.28967
AAEL005753	serine protease	8.1279	8.41404	4.94646	5.73754	13.6265	9.62793	0.528708	5.18716
AAEL014980	serine protease inhibitor (serpin-2), putative	34.9374	35.8279	43.4731	40.1541	93.8638	95.7965	2.12066	14.1633
AAEL007106	serine protease, putative	10.5217	11.0405	5.24654	4.96343	9.04725	7.59097	0.530703	5.26045
AAEL006434	serine protease, putative	11.7441	11.158	5.61395	5.97147	52.0446	34.9575	1.41578	5.98632
AAEL010340	serine/arginine rich splicing factor	146.525	161.839	123.329	119.784	83.5508	88.9994	91.4183	109.496
AAEL002569	serine/threonine kinase	30.0794	29.003	36.6012	34.9492	24.245	24.7299	1.56553	15.8517
AAEL013390	serine/threonine protein kinase	51.7456	52.7685	83.1542	75.4838	56.3323	59.4918	7.78834	33.2288
AAEL003896	serine/threonine protein kinase	24.7219	24.1592	44.9311	40.523	24.9177	26.7109	6.00874	14.4894
AAEL009880	serine/threonine protein kinase	13.7881	14.3596	24.7158	20.7946	10.6384	12.9803	1.32966	8.95051
AAEL006712	serine/threonine protein kinase	48.009	41.6922	58.0997	65.0177	22.8761	25.1887	3.42483	28.3857
AAEL007544	serine/threonine-protein kinase chk2 (cds1)	40.1035	37.8459	43.2148	42.9361	50.0294	48.5423	1.22799	18.8366
AAEL007617	serine/threonine-protein kinase vrk	28.8391	30.1515	24.4641	22.5819	43.6574	46.8761	7.6276	19.1217
AAEL009244	serine-type enodpeptidase, putative	1.9736	0.26631	2.18524	2.13716	7.32693	21.4919	1333.73	686.862

AAEL001674	serine-type enodpeptidase, putative	0.554676	1.37939	0.618967	0.298141	0.376425	0.612492	3327.14	3271.28
AAEL001701	serine-type enodpeptidase, putative	0.587986	2.79461	1.16936	0.839399	1.60839	3.58443	1931.05	1861.62
AAEL002360	serine-type enodpeptidase, putative	0.574677	0.196946	0.085618	0.195371	0.424052	0.289248	1416.17	315.466
AAEL008769	serine-type enodpeptidase, putative	4.86315	4.93077	6.09795	6.03783	4.72871	5.64819	520.425	629.112
AAEL008782	serine-type enodpeptidase, putative	0	0	0.052272	0	0.323959	0.281406	17.1735	3.4453
AAEL014188	serine-type enodpeptidase, putative	1.52568	2.39672	21.659	8.00597	65.9631	203.168	22510.3	6507.16
AAEL011929	serine-type enodpeptidase, putative	0.157318	0.213959	0	0	0.08263	0.568386	364.427	140.353
AAEL006902	serine-type enodpeptidase, putative	0.348888	0.475295	0.340424	0.396655	0.682074	1.69464	2418.77	918.026
AAEL003060	serine-type enodpeptidase, putative	9.66325	12.9423	23.9898	25.7298	343.821	1028.19	63292.9	31369.3
AAEL008780	serine-type enodpeptidase, putative	0	0	0.053808	0	0.068015	0	60.0512	73.312
AAEL001690	serine-type enodpeptidase, putative	1.20844	2.52847	0.886466	0.76429	1.48354	3.84656	5887.58	5018.15
AAEL001703	serine-type enodpeptidase, putative	2.52066	2.847	11.5919	9.9323	164.997	315.226	16361.2	7398.97
AAEL009843	serine-type enodpeptidase, putative	0.065149	0.388406	0.191223	0.22106	0.597605	1.42834	1395.91	745.446
AAEL006598	serine-type enodpeptidase, putative	0	0	0	0	0	0	95.8172	18.9193
AAEL008784	serine-type enodpeptidase, putative	0.151654	0.351016	1.50262	1.63221	3.69486	8.31753	1042.77	435.322
AAEL008567	serine-type enodpeptidase, putative	0	0.103834	0.723914	1.59787	4.26847	7.0456	94.255	29.6868
AAEL001693	serine-type enodpeptidase, putative	1.1227	0.986759	0.758019	0.584029	1.02221	1.58966	5621.47	3218.89
AAEL002347	serine-type enodpeptidase, putative	0.475438	0.401555	0.25666	0.059295	0.488038	1.78208	2424.52	1015.72
AAEL006594	serine-type enodpeptidase, putative	0.823603	1.04684	0.564051	0.827877	0.984821	4.99986	6717.48	2252.34
AAEL012378	serine-type protease inhibitor	10.2152	9.90028	25.1541	10.5674	29.733	33.252	27.8766	17.1581
AAEL005037	seryl-tRNA synthetase	69.0668	85.1923	48.2628	39.4552	78.5691	80.1905	44.4147	57.444
AAEL010414	set domain protein	30.4885	30.6154	48.6728	41.8587	34.3042	33.6969	2.66023	17.1316
AAEL010809	SET, putative	68.7265	65.5659	93.7964	67.2573	63.8294	60.1521	20.9171	43.4905
AAEL011182	sex-determining protein fem-1	33.8152	31.928	52.5007	21.5395	29.3559	29.1586	31.2935	32.2077
AAEL000584	sex-determining region y protein, sry	31.8848	31.6639	43.5577	43.9794	27.929	32.2497	0.374043	14.7014
AAEL008739	shc transforming protein	29.1074	28.7119	46.2286	41.6838	21.1633	21.7037	10.6534	18.659
AAEL007578	shk1 kinase-binding protein	16.1815	17.9503	19.5662	17.649	21.0744	22.7732	1.57431	8.58221
AAEL008159	short-chain dehydrogenase	2.25045	3.74744	1.19204	1.10789	2.76803	2.73634	664.783	261.328
AAEL010290	short-chain dehydrogenase	26.0576	23.1767	54.9486	56.9166	20.235	21.5996	0.707209	9.85406
AAEL008152	short-chain dehydrogenase	41.4982	37.7982	56.1259	55.363	16.4199	18.3837	328.521	165.152
AAEL009625	short-chain dehydrogenase	9.89981	12.4436	17.8828	17.6609	27.3798	21.4024	226.273	116.609
AAEL008016	short-chain dehydrogenase	15.9086	19.4148	8.66451	6.0925	11.1435	10.1164	120.405	45.807
AAEL003139	short-chain dehydrogenase	0.796527	1.12201	2.17216	11.5322	1.57836	2.26992	4.92116	5.732
AAEL006224	short-chain dehydrogenase	48.7666	49.6706	63.3718	59.6372	76.4838	70.839	233.108	131.976
AAEL002493	short-chain dehydrogenase	11.339	12.2733	14.2619	12.052	7.82119	8.92481	6.0559	11.6274

AAEL008227	short-chain dehydrogenase	23.5122	19.2683	7.58536	9.60257	10.8503	9.32738	3.23419	13.4114
AAEL003961	short-chain dehydrogenase	18.8704	17.7093	44.6397	42.0551	16.2605	16.0773	0.636785	10.1618
AAEL006318	short-chain dehydrogenase	0.725497	1.161	1.03135	0.506961	0.450162	0.734987	104.763	52.9964
AAEL014526	sideroflexin 1,2,3	58.5364	76.9284	33.9898	25.75	117.58	126.223	34.0001	66.6592
AAEL009810	sideroflexin 1,2,3	36.8142	34.8797	53.9759	59.0201	38.2412	30.5837	25.122	31.4964
AAEL004603	signal peptidase 12kDa subunit, putative	47.0098	55.3273	18.3096	19.4848	25.5312	27.5704	40.2154	52.1152
AAEL011989	signal peptide peptidase	114.777	139.069	35.7246	42.8314	31.9507	35.895	113.267	121.53
AAEL004125	signal transduction protein lnkalted	36.4978	35.6921	26.6647	29.0806	41.864	44.9909	6.8828	19.9731
AAEL007252	sin3a-associated protein sap130	26.9232	27.0666	16.8143	23.0914	12.4792	12.2168	12.5629	19.7175
AAEL000888	single stranded DNA binding protein	47.8322	45.9302	74.2576	96.2254	48.4868	49.6828	36.5081	66.2852
AAEL013286	single-strand selective monofunctional uracil DNA glycosylase	22.5567	34.4721	30.9169	26.8153	58.0086	56.433	12.4588	18.7484
AAEL000840	skeletal muscle/kidney enriched inositol 5-phosphatase	14.0553	13.9821	14.8913	20.7422	26.8037	24.9478	123.669	63.9733
AAEL009160	skp1	182.034	188.433	283.87	254.891	174.965	180.035	262.754	183.12
AAEL010651	skp1	1.45205	5.8835	14.9794	20.2551	1.47962	9.90181	0	2.9674
AAEL002774	slender lobes, putative	13.029	10.3037	5.75584	6.11593	10.9652	9.13765	88.7807	38.8297
AAEL012935	slingshot dual specificity phosphatase	48.3883	43.1937	58.0378	48.6614	47.9945	49.3789	2.5963	20.7661
AAEL008719	Sm protein G, putative	54.2533	58.5234	68.4963	64.52	48.9228	52.6294	25.2808	40.3841
AAEL004589	small calcium-binding mitochondrial carrier, putative	81.788	77.2108	163.824	139.942	76.5565	63.5289	54.4093	55.2911
AAEL001382	small nuclear ribonucleoprotein sm d1	27.8326	39.8332	34.5522	26.7652	25.0916	25.4754	11.8414	21.1335
AAEL001363	small nuclear ribonucleoprotein Sm D1, putative	51.1238	48.6857	45.4442	43.2899	41.2837	53.0967	20.7056	46.3688
AAEL009914	small nuclear ribonucleoprotein sm d2	49.1135	51.7306	60.6396	53.5776	40.5904	44.5977	10.9721	29.3222
AAEL011433	small nuclear ribonucleoprotein sm d3	84.6456	86.9676	112.954	98.7108	70.1282	68.1923	44.345	63.9932
AAEL003985	small nuclear ribonucleoprotein, core, putative	34.804	39.0289	38.0103	36.5735	31.4947	33.967	17.9537	38.4631
AAEL002377	small nuclear ribonucleoprotein-associated protein	64.1095	69.1607	70.486	67.0452	51.9454	52.7107	22.6353	40.6974
AAEL003509	smap1	27.7607	25.5179	29.5742	22.9675	19.2113	20.9261	57.1507	23.6702
AAEL013510	smaug protein	98.7953	95.396	198.968	139.07	70.1156	76.1467	7.93118	54.2253
AAEL006939	smaug protein	38.9884	42.3846	74.8401	53.3698	29.1904	31.2103	3.9346	21.3007
AAEL013544	snail protein, putative	20.0122	20.326	27.0728	23.222	16.8522	21.1273	0.874141	9.5225
AAEL007661	SOCS	10.71	8.3663	15.1693	14.2016	7.47631	8.57054	1.15117	5.28237

AAEL011069	SOCS	23.2732	24.5358	52.9613	76.511	25.9	26.9463	6.35823	13.4371
AAEL010738	sodium bicarbonate cotransporter	8.39695	9.94101	5.86504	5.58453	9.40194	10.606	113.412	45.2918
AAEL009863	sodium/dicarboxylate cotransporter, putative	46.32	44.8346	34.175	39.3262	50.487	46.5519	153.598	71.8889
AAEL001503	sodium/hydrogen exchanger 3 (nhe3)	1.74753	1.79447	3.36002	1.92811	3.3075	4.37398	108.744	51.4587
AAEL010145	sodium/potassium-dependent atpase beta-2 subunit	58.9061	61.1871	129.892	102.542	47.2558	54.7187	209.007	128.45
AAEL003626	sodium/shloride dependent amino acid transporter	4.61557	3.69412	12.7641	2.6362	25.7026	21.7055	1.48571	1.69836
AAEL003638	sodium/shloride dependent amino acid transporter	0.676134	0.900031	1.1769	1.7002	12.7614	2.58538	0.43256	1.05002
AAEL003624	sodium/shloride dependent amino acid transporter	25.9631	19.9357	198.8	97.4625	24.7268	25.6631	0.52098	10.5344
AAEL006995	sodium/solute symporter	2.35226	0.175501	0.619799	0.361123	0.382176	0.920033	7.49307	0.055218
AAEL006139	sodium/solute symporter	34.0407	30.8872	19.0685	26.7928	26.0318	29.8594	138.326	73.0751
AAEL001835	sodium/solute symporter	1.01246	0	0.358562	0.867818	0.335291	0.510119	5.65887	0.3778
AAEL010650	sodium/solute symporter	40.2572	40.6136	17.9633	22.4721	19.0908	19.3805	216.003	126.226
AAEL002555	sodium/solute symporter	3.28189	4.65821	3.78475	1.90599	3.37921	2.64387	1.71626	19.5352
AAEL008915	sodium-and chloride-activated ATP-sensitive potassium channel	6.35502	6.78769	5.56494	7.10298	7.30977	7.61172	2.06942	8.27096
AAEL012440	sodium-bile acid cotransporter	26.0535	25.7354	12.1618	15.4164	23.9629	21.6597	2737.03	1174.3
AAEL012522	sodium-dependent phosphate transporter	1.16257	1.14026	1.05824	0.537533	0.593988	0.665364	16.1	3.52408
AAEL001656	sodium-dependent phosphate transporter	19.9956	17.2022	25.6194	23.6256	13.1061	14.9755	656.942	290.839
AAEL009749	sodium-dependent phosphate transporter	24.1717	22.1204	11.1972	22.7875	32.3605	31.8584	1.3696	12.4414
AAEL005484	sorting nexin	20.0065	21.189	23.433	16.0765	21.575	21.0295	4.30465	13.4816
AAEL008020	sorting nexin	22.7891	23.9506	10.0911	14.9012	30.7448	30.3467	17.7378	24.4386
AAEL003758	sorting nexin	19.7776	20.4548	25.518	20.8171	21.9504	19.0245	132.929	47.8066
AAEL012364	spermatogenesis associated factor	104.094	104.077	71.3569	75.1242	62.1312	70.5656	176.969	87.8812
AAEL000836	spermatogenesis associated factor	6.18706	6.44988	3.82819	2.9917	11.7319	13.2599	196.982	88.871
AAEL010071	spermidine synthase	40.2077	48.4916	37.9795	34.331	52.7277	53.214	6.09129	30.5125
AAEL001378	spermine synthase	34.3123	37.8323	19.8141	20.4805	28.6523	28.4781	7.09981	21.9312
AAEL005068	S-phase kinase-associated protein 2 (skp2), putative	16.8032	13.8536	20.6501	21.5541	10.341	9.32856	4.68973	10.8566
AAEL013047	sphingolipid delta 4 desaturase/c-4 hydroxylase protein des2	21.683	19.5599	11.6221	13.5544	22.4532	17.8521	251.038	88.3586

AAEL006381	sphingomyelin phosphodiesterase	0.109746	0.093996	0.058758	0.045558	0.123254	0.054028	217.19	77.6245
AAEL013717	sphingomyelin phosphodiesterase	0.644242	1.56864	0.340971	0.420382	0.63025	0.800256	2959.98	1717.94
AAEL002413	sphingomyelin phosphodiesterase	20.1913	20.9569	3.08482	3.46539	8.7135	8.8711	2.5763	12.7446
AAEL003188	sphingosine phosphate lyase	15.7623	14.3469	27.3132	23.3273	14.8532	14.2232	228.786	117.818
AAEL004710	spingomyelin synthetase	20.1808	19.4704	35.9046	18.3018	14.7948	15.7662	23.8838	12.0572
AAEL002709	spliceosomal protein sap	42.825	48.0298	32.8204	27.4749	44.0442	40.7983	16.1312	34.4524
AAEL013795	spliceosome associated protein	32.4408	29.9325	35.1117	27.2576	39.2404	39.4043	13.1814	20.9581
AAEL002818	splicing factor u2af large subunit	64.6853	67.773	60.611	54.2564	50.4669	48.3129	34.3728	50.4778
AAEL003916	splicing factor, putative	38.2549	36.1509	42.421	40.2886	31.0341	31.704	19.0032	28.8806
AAEL004010	splicing factor, putative	72.8953	79.3405	70.2411	67.3437	69.3707	66.55	65.4798	70.1203
AAEL000499	SPZ1A	42.8037	43.2974	53.5505	56.1555	32.32	37.3213	0.585263	24.9404
AAEL013433	SPZ1C	15.6307	18.3101	8.28646	10.6646	18.1798	15.0233	0	5.0641
AAEL008507	srpk	27.7567	29.6933	41.6072	37.3054	37.0395	38.1641	11.7277	18.9698
AAEL007765	SRPN10	53.333	50.161	54.4526	54.0977	102.03	49.8842	2580.87	948.826
AAEL003686	SRPN11	46.8575	49.0328	66.0103	53.7558	144.048	136.892	3.34451	22.7916
AAEL005673	SRPN2	42.4996	45.8273	42.961	44.4598	134.484	133.044	4.17783	20.4761
AAEL014078	SRPN2	6.64393	7.03336	6.34658	6.87781	19.0399	15.7865	0.551747	4.41461
AAEL002720	SRPN20	45.4156	52.4514	42.3872	36.347	117.019	107.064	1.9728	16.7124
AAEL005665	SRPN3	41.2036	44.6428	52.6474	49.8444	71.8973	65.6837	2.57187	19.5905
AAEL013936	SRPN4	55.9887	56.784	69.4773	66.3411	182.651	158.58	4.54609	22.732
AAEL014141	SRPN5	17.3482	17.9319	16.7084	17.6062	18.5667	14.8692	1.47714	9.86984
AAEL011777	SRPN8	16.9036	16.817	18.7718	17.8016	46.2281	42.4653	1.57357	7.46817
AAEL008364	SRPN9	76.484	76.4856	106.074	99.4998	87.6808	75.7459	3.87512	39.1773
AAEL006748	ss-DNA binding protein 12RNP2 precursor, putative	48.7806	51.7071	42.6736	41.1695	41.3547	46.3301	26.6693	35.2335
AAEL012840	stathmin	60.0534	57.2034	121.607	104.429	47.8405	51.0835	1.93555	20.4889
AAEL007470	staufen	26.5828	27.6226	37.737	0	21.9476	22.8279	11.8902	18.8137
AAEL003645	stearoyl-coa desaturase	11.5994	10.3587	11.8893	16.2098	32.3734	17.5062	1082.24	415.836
AAEL008484	steroid receptor-interacting snf2 domain protein	18.5641	16.9773	7.10197	8.55039	24.2895	23.1973	8.44844	14.3323
AAEL015661	sterol carrier protein-2, putative	0.789721	1.5287	0	0.859567	1.44988	1.31596	8645.5	4207.62
AAEL002687	sterol carrier protein-2, putative	24.4773	22.4429	18.0421	18.7272	20.8982	18.4286	321.668	172.553
AAEL012697	sterol carrier protein-2, putative	0	0.171877	0.105735	0.121974	0	0	634.934	242.179
AAEL000135	sterol desaturase	0.360982	0.803823	0.177752	0.142737	0.657945	1.05167	523.261	310.708
AAEL012425	sterol desaturase	2.26547	2.51151	0.337238	5.16782	0.845064	0.711922	7.84386	10.9032

AAEL001204	sterol o-acyltransferase	91.8086	90.1806	121.703	73.4847	91.4279	92.3454	118.359	89.7339
AAEL000798	sterol regulatory element binding protein cleavage-activating protein (srebp cleavage-activating protein) (scap)	24.9064	27.4485	21.4331	23.3805	21.0582	21.4676	5.84111	18.445
AAEL013913	stretch regulated skeletal muscle protein, putative	6.75086	1.68203	8.91204	11.3299	29.1442	3.49753	19.1722	6.0039
AAEL003449	structural maintenance of chromosomes smc2	11.9503	11.7019	16.8355	14.4211	12.0849	11.6603	2.27444	7.35904
AAEL010485	sugar transporter	1.44167	1.70384	0.651223	0.614255	16.1631	21.1263	74.2955	23.1983
AAEL013489	sugar transporter	5.14293	6.01919	5.54293	3.45575	7.81623	6.36328	0.57463	4.9084
AAEL010479	sugar transporter	4.35333	4.04363	29.9458	42.9717	30.4349	37.4795	598.1	245.371
AAEL008232	sugar transporter	2.65769	1.6248	10.2101	9.50887	4.48435	3.02967	53.7	19.2492
AAEL014207	sugar transporter	7.87056	7.7945	6.97217	7.52748	5.40751	6.88663	1.53134	5.80837
AAEL003808	sugar transporter	15.4161	13.7484	46.159	53.7569	19.3461	19.0567	233.1	132.635
AAEL007136	sugar transporter	52.2079	57.3168	32.7057	31.2651	45.6071	44.5906	27.3049	33.3816
AAEL003899	sugar transporter	9.52912	6.82927	2.00691	2.57462	6.00579	5.76597	163.766	79.9014
AAEL012655	sugar transporter	21.734	25.3597	78.4376	40.6446	24.7134	25.015	159.708	80.6092
AAEL007126	sugar transporter	6.30649	0.231826	1.19839	0.77702	1.61979	2.44073	13.9365	0.52324
AAEL007128	sugar transporter	1.21453	0	1.66053	1.68333	1.23468	2.08116	5.85711	0
AAEL012041	sulfate transporter	5.60649	3.56679	3.77651	4.17146	4.59375	4.66076	244.725	86.5531
AAEL006344	sulfotransferase (sult)	19.2921	24.1603	10.7241	9.65216	14.2751	11.2337	0.986503	8.39003
AAEL006353	sulfotransferase (sult)	10.2142	11.1349	6.7784	11.8433	1.98369	2.60187	163.711	88.4089
AAEL015305	sulfotransferase (sult)	16.1013	17.1775	8.11995	4.34524	8.01527	5.74139	3.58381	8.64507
AAEL015099	sumo ligase	88.4945	90.3609	105.618	92.8548	80.6595	83.336	35.0359	62.0781
AAEL000091	sumo-1-activating enzyme E1a	48.409	44.5318	69.4602	68.8979	26.271	28.3701	19.2607	30.4754
AAEL000274	superoxide dismutase	57.6633	53.9556	99.7114	72.8836	48.8077	45.4408	95.9439	54.1644
AAEL003105	supervillin	28.9669	24.3403	61.9453	61.8294	24.1196	23.7884	98.1785	50.2
AAEL013408	suppressin	39.9397	38.9604	29.9002	33.7998	52.7501	52.8081	22.472	31.2307
AAEL006566	suppressor of ty	33.7292	36.8271	46.4025	31.3117	41.4191	54.734	34.603	32.7428
AAEL008235	surfeit locus protein	9.31414	5.07674	8.05138	6.62419	9.8508	10.8588	25.6147	7.71438
AAEL004371	SWI/SNF related matrix associated actin dependent regulator of chromatin subfamily B member 1	29.162	27.5789	27.5577	26.1182	26.8307	27.6748	11.4164	19.8262
AAEL007315	symbol, putative	21.5056	23.8317	7.65736	7.6196	7.77862	8.94628	4.75411	15.8053
AAEL002437	symplekin	18.7777	17.3093	19.6909	21.2949	16.8079	15.8621	7.21311	13.4407
AAEL012098	synaptic vesicle protein	19.5198	20.0234	26.6925	31.2942	10.9179	12.2243	0.387544	10.7675

AAEL005849	synaptic vesicle protein	20.2466	1.37436	31.3927	57.5473	13.7669	39.5152	54.0707	1.99738
AAEL005533	synaptic vesicle protein	2.61584	0.300176	0.131366	0.05067	0.257424	0.3724	7.34023	0.731917
AAEL015283	synaptic vesicle protein	0.362178	0	0.314333	0.1523	0.589046	0.784903	83.8672	39.2893
AAEL013884	synaptic vesicle protein	20.3573	15.518	2.90162	2.87867	7.0001	6.51319	64.4606	30.391
AAEL013571	synaptobrevin	77.444	75.3326	67.8216	57.9884	86.8892	83.5656	428.146	194.223
AAEL005479	synaptogyrin, putative	10.6021	10.3099	12.7959	12.2302	12.0245	14.0313	0.349072	7.08072
AAEL009169	synaptotagmin, putative	21.848	19.1407	18.234	16.9768	27.9826	28.616	130.047	62.3128
AAEL007282	syntaxin binding protein-1,2,3	24.9884	21.9548	28.0078	25.6617	18.2888	18.8992	117.684	58.5906
AAEL005391	syntenin	51.4411	53.7764	59.8112	56.3994	51.3783	50.2656	232.831	120.286
AAEL014209	tangerin	6.96909	17.9556	9.50677	9.52847	10.6648	5.35406	27.253	8.74815
AAEL005276	target of myb1 (tom1)	14.2809	14.3443	9.62668	9.62994	15.1869	12.1335	146.287	67.5687
AAEL010341	TATA-binding protein, putative	35.0707	40.2699	48.2837	43.7726	38.5104	38.8132	10.3772	19.6254
AAEL013653	tata-box binding protein	47.5162	51.6451	54.5234	52.0862	52.8983	53.2598	11.2119	27.8986
AAEL000661	t-cell specific transcription factor, tcf	12.4402	14.3046	17.4427	15.9585	9.05274	10.8579	0.402481	7.3822
AAEL003872	Tctp	659.165	701.631	416.891	343.842	2535.46	2385.58	1073.35	793.077
AAEL000428	Tdo	11.4114	13.2723	6.25428	10.6761	44.3346	35.1923	330.98	135.725
AAEL000087	TEP22	19.9072	27.4194	7.91387	9.24408	16.2656	18.8046	1.11661	9.34807
AAEL008607	tep3	18.4613	16.9573	22.3535	17.2962	90.4102	63.3978	7.84752	5.93337
AAEL002250	terminal deoxycytidyl transferase rev1	10.749	9.25992	15.9367	15.3568	5.26587	5.5754	0.802971	5.08752
AAEL014017	testis-specific serine/threonine kinase 22c	12.4475	12.6979	12.841	13.3642	10.3975	11.429	0	7.77254
AAEL003210	tetraspanin 29fa	107.013	117.582	91.2698	99.1308	106.021	110.053	454.434	250.779
AAEL012532	tetraspanin 29fb	1.91024	0.366334	1.13081	1.2137	4.60214	3.9224	1147.7	564.461
AAEL005536	tetraspanin 29fb	1.1668	0.558015	1.47587	1.3141	8.11	8.54956	1086.3	415.918
AAEL012528	tetraspanin, putative	0.673131	0	0.38379	0.06377	0.809185	1.16901	38.9045	15.5343
AAEL005137	tetraspanin, putative	19.0852	17.4591	53.8197	45.657	20.1951	23.0424	1.1868	10.9346
AAEL005545	tetraspanin, putative	0.71243	0	0.103207	0.1195	1.5488	0.698958	29.1633	11.0463
AAEL010967	tetraspanin, putative	16.2564	14.8103	23.2952	37.9931	10.0052	11.0308	1.96273	8.30675
AAEL013875	tetraspanin, putative	107.298	104.398	101.821	103.387	213.348	221.058	1419.13	542.926
AAEL010102	tetraspanin, putative	5.92796	7.24745	4.18138	4.04291	8.2398	11.5506	0.959791	5.59068
AAEL008743	Tetratricopeptide repeat protein, putative	5.09344	5.45438	7.4055	5.90456	10.9662	10.5344	1.6829	5.86163
AAEL006462	TFIID subunit TAFII55, putative	19.8367	18.9095	24.0612	20.1082	20.6511	20.823	7.19677	13.0825
AAEL013218	thiamine transporter	0.200581	0.142669	53.6818	35.3708	0.107917	0.273745	0	0.271003
AAEL010777	thioredoxin (TRX), putative	363.448	341.839	480.422	405.463	310.394	334.2	663.037	371.554
AAEL009708	thioredoxin M, putative	24.6724	27.7191	25.1468	22.7764	23.3016	22.3897	122.128	62.9363

AAEL002886	thioredoxin reductase	142.284	146.741	117.641	117.837	139.098	142.242	483.769	236.531
AAEL000435	THO complex, putative	15.8525	15.1904	14.7076	15.4204	13.6094	14.0038	5.72411	11.8797
AAEL000049	three prime repair exonuclease 1, putative	17.9356	21.7742	3.30137	2.41052	9.65681	7.36327	171.168	75.3182
AAEL003443	threonine dehydrogenase	16.2994	26.8493	1.15195	0.551075	51.18	57.8421	0.963277	18.062
AAEL014055	thymidine kinase	71.8416	70.1648	96.3027	96.5843	46.3369	38.6684	53.4258	68.1728
AAEL001246	Thymidylate kinase, putative	47.893	34.8269	90.9882	73.0773	12.5005	12.9439	6.81305	41.3298
AAEL011653	thyroid hormone receptor interactor	51.6506	46.2962	57.0709	65.4661	21.7707	22.2738	1.86347	25.6707
AAEL006411	TIM	11.14	10.4063	5.80156	4.11205	20.9354	17.4771	91.2574	43.5688
AAEL010450	TIM10	147.911	71.3273	196.978	161.742	183.297	174.568	101.934	82.923
AAEL009518	timeout/timeless-2	16.9725	17.6028	20.9687	24.2732	18.0629	17.4874	3.39894	9.84247
AAEL013525	Timp-3, putative	245.337	154.204	11.1751	15.5837	25.8395	23.8652	56.9302	82.3301
AAEL014072	tip60	17.1836	16.2506	17.3536	15.615	19.0453	20.1558	3.02002	9.20408
AAEL000967	tmc7 protein	8.60999	9.63266	8.78413	10.1335	8.60919	8.6115	52.9143	22.9938
AAEL017403	TMOF	14894.6	10702.9	11.6904	1347.84	1.51613	1.67406	110.266	8572.03
AAEL006649	tnf receptor associated factor	14.6344	12.7204	0.631409	0.2495	1.83006	1.38775	3.45745	8.31495
AAEL001771	toll	4.46405	4.9024	0.788625	1.60468	2.72448	2.92502	0.353739	3.93658
AAEL013441	TOLL9A	1.97802	1.44199	0.764569	1.20404	2.17955	1.08355	141.476	57.0731
AAEL011734	TOLL9B	6.40549	5.31182	12.3813	9.80417	2.50739	2.34013	159.882	73.1356
AAEL004112	TPX2	1025.42	912.16	1824.5	1617.74	419.561	405.576	676.161	638.276
AAEL002309	TPX4	12.2847	16.8804	48.093	56.3703	45.3366	19.3679	1149.41	375.587
AAEL003912	trafficking protein particle complex subunit 6b	25.6942	26.9818	31.2105	26.5995	30.9746	39.3125	17.2494	29.9102
AAEL009389	transaldolase	137.744	138.554	178.893	152.046	107.791	107.698	104.633	112.422
AAEL001496	transcription elongation factor s-ii	77.1902	68.9999	76.0823	73.8544	59.3824	57.7053	28.1831	49.1281
AAEL001321	Transcription factor dp	42.935	38.9263	42.4816	48.3897	38.1037	37.9239	14.8613	25.6158
AAEL013151	transcription factor IIB, putative	28.0501	30.9446	41.5498	38.3026	26.8067	28.671	4.90224	15.1898
AAEL009798	transcription factor IIIA, putative	17.7959	21.1065	36.3135	29.1291	13.7455	13.9463	5.04319	18.9818
AAEL011858	transcription factor IIIA, putative	7.03652	6.72734	8.96014	7.5873	10.7767	10.1305	1.7908	6.22059
AAEL005975	transcription initiation factor	54.4169	54.9402	75.2219	68.3375	37.1618	37.1695	14.9593	34.6981
AAEL012202	transcription initiation factor IIE, alpha subunit	15.0585	15.7577	22.9074	19.0132	12.9082	12.8957	4.42643	10.4105
AAEL012265	transcription initiation factor RRN3	63.1688	66.5663	41.1268	45.2174	88.1092	68.3982	11.297	36.3334
AAEL015458	transferrin	121.788	141.191	18.4925	94.6397	159.421	167.22	8.49976	29.7798
AAEL015639	transferrin	72.9878	80.4789	7.34868	15.3261	76.7894	75.854	5.00444	19.5414
AAEL011641	transferrin	1.18615	0.60216	0.787966	0.600374	12.2625	7.14767	1018.71	342.49

AAEL004434	transketolase	291.652	323.038	424.795	434.486	299.455	291.484	208.882	226.152
AAEL003661	translation initiation factor	82.2104	94.2871	107.231	88.359	189.387	190.754	43.9102	59.9197
AAEL013828	translation initiation factor eif-2b	9.86336	11.5601	9.67397	7.20034	13.051	15.4517	48.1433	20.2146
AAEL003285	translocation associated membrane protein	146.127	191.098	36.6723	30.2769	51.6814	58.9917	357.226	295.12
AAEL010032	translocon-associated protein, beta subunit precursor (trap-beta) (signal sequence receptor beta subunit)	321.9	461.037	121.401	111.887	160.381	160.75	256.478	317.453
AAEL013320	translocon-associated protein, delta subunit	261.002	347.299	79.783	79.1061	85.3625	87.5771	257.622	258.983
AAEL007441	translocon-associated protein, gamma subunit	267.042	389.475	72.9298	65.2034	93.1449	99.7372	266.021	335.496
AAEL009995	transmembrane protein, putative	67.2529	64.1845	43.2893	38.0644	39.5101	45.2428	33.8881	47.0583
AAEL003573	transportin	42.1497	45.2815	22.2008	24.1477	41.0368	41.9188	20.8029	31.0883
AAEL002287	trans-prenyltransferase	14.7717	14.1918	13.5198	32.7483	19.3722	17.3101	8.20089	9.80252
AAEL006446	trehalose-6-phosphate synthase	78.2962	82.5113	63.0133	121.242	148.348	150.224	17.4936	29.0995
AAEL000567	Tret1	44.6442	66.1458	99.6711	148.303	211.246	222.522	22.8064	19.443
AAEL014551	triacylglycerol lipase, pancreatic	43.501	49.2549	3.07633	2.83939	11.1029	10.8213	2.40803	22.4855
AAEL014553	triacylglycerol lipase, pancreatic	2.3948	3.12788	13.6699	10.8148	3.01856	2.22434	0.155101	7.17893
AAEL005991	tricarboxylate transport protein	80.7359	86.5481	76.4989	71.3026	136.841	137.266	87.8766	89.619
AAEL002542	triosephosphate isomerase	359.977	361.791	522.369	463.658	121.821	121.279	343.9	264.593
AAEL011745	Trisn small nuclear ribonucleoprotein, putative	15.8775	16.1486	14.7274	15.294	7.49202	7.34204	5.12354	11.514
AAEL009156	trna (guanine-n(7)-)-methyltransferase	18.7792	20.4571	13.3056	14.8052	20.861	22.4558	6.93814	12.6584
AAEL003071	tRNA pseudouridine synthase D	30.1523	36.0985	17.9461	13.7542	103.232	93.0541	10.52	22.7432
AAEL016671	tRNA-Ala	22.6677	8.02336	13.1972	21.6695	0	3.92116	43.1555	13.8081
AAEL001171	tRNA-dihydrouridine synthase	15.1147	16.5448	13.9886	12.523	41.1871	38.6356	4.98609	11.1845
AAEL016814	tRNA-His	36.7912	42.9547	65.1874	56.2161	45.4083	47.8578	6.42869	16.4572
AAEL007029	tropomodulin	21.8217	21.2237	55.0933	36.1415	16.4656	15.3342	120.721	66.6629
AAEL013703	trypsin	0	0	3.20943	3.31584	19.8349	17.6631	0	88.2007
AAEL013715	trypsin	0.143033	0.910218	0.154925	0.357481	0.584241	2.54228	0	1704.94
AAEL010196	trypsin	2.30674	4.19875	1.37661	1.37358	1.4024	4.33416	17806	8779.25
AAEL008085	trypsin	0.504785	0.582922	1.07427	2.33645	7.42601	7.07983	3875.96	1597.48
AAEL010195	trypsin	0.355082	0	0	0.368776	0.161068	0.176152	696.602	266.7
AAEL013623	trypsin	0.08631	0.495938	1.6214	0.881309	4.17182	10.3503	1096.96	562.736
AAEL013707	trypsin	0.322072	0.553116	0.174497	0.067768	1.02101	2.94639	0	1066.95

AAEL013713	trypsin	5.76708	12.6375	3.77006	4.19893	2.72285	3.26327	0	22930.5
AAEL006425	trypsin	1.46023	1.06787	5.41523	3.90067	28.2279	50.7602	2707.07	1240.36
AAEL013712	trypsin	4.0789	9.11699	2.41374	2.48384	2.87362	3.22734	0	15001.7
AAEL007601	trypsin	0	0	0.049531	0	0	0.205295	236.152	217.822
AAEL010202	trypsin	0.730288	1.88981	0.346838	0.706844	0.43584	0.776664	8464.44	4708.23
AAEL007818	trypsin	0.598704	1.03844	25.159	28.9581	806.878	1856.55	2194.61	1484.11
AAEL008093	trypsin	0.202416	0.570643	0.417135	0.962202	3.77817	3.18265	1781.45	749.114
AAEL012852	trypsin	1.07288	0.648667	0.100251	0.058749	0.832815	0.421474	342.899	147.512
AAEL006903	trypsin	0	0.202985	0.251217	0.144234	0	0	1371.29	558.189
AAEL006121	Trypsin, putative	17.6879	16.7097	18.2658	20.8345	22.529	27.1024	0.688476	9.86687
AAEL013714	trypsin, putative	9.60776	16.0204	5.32964	8.58474	8.53029	12.6547	0	41018.2
AAEL015528	Trypsin, putative	0	0	0	0	0	0	39.506	5.22161
AAEL015104	Trypsin, putative	0	0	0	0	0.141327	0.076719	10.9723	2.13964
AAEL006123	Trypsin, putative	11.2496	13.527	28.5486	23.5881	16.657	17.0331	0.249067	6.43772
AAEL006378	trypsin, putative	0	0	0	0	0	0	9.88522	1.41965
AAEL006384	Trypsin, putative	0	0	0	0	0	0	37.2052	8.74427
AAEL013629	trypsin-alpha, putative	0.0444	0.364145	0.391268	0.304633	4.04393	3.42729	583.673	244.702
AAEL008079	trypsin-alpha, putative	0.519259	0.17422	0.21492	0.156182	3.66016	1.71715	567.197	204.52
AAEL006368	trypsin-beta, putative	0	0	0	0	0	0	15.7254	2.01389
AAEL008080	trypsin-eta, putative	4.25989	2.96547	2.28153	2.4075	21.6952	28.3333	472.894	183.012
AAEL008097	trypsin-eta, putative	4.77716	6.41805	1.57951	1.53128	7.54535	5.91044	454.568	175.224
AAEL006179	tubulin alpha chain	7.64198	5.83242	9.00915	9.33737	2.06887	1.17328	1.23503	5.71464
AAEL006642	tubulin alpha chain	903.46	797.877	1330.56	1093.77	380.404	394.092	1048.8	875.655
AAEL004172	tubulin alpha chain	70.4916	68.0643	105.199	122.157	35.8332	39.2082	1.44186	33.6338
AAEL002851	tubulin beta chain	764.125	724.505	1060.47	934.633	477.911	446.508	543.2	542.988
AAEL005084	tubulin beta chain	39.606	40.3843	21.0517	15.5588	38.7183	42.2111	16.4157	32.2882
AAEL005052	tubulin beta chain	2.10748	2.76663	3.90876	2.72427	28.4794	21.4868	79.4373	33.9488
AAEL002848	tubulin beta chain	52.863	49.4375	3.70432	3.06938	6.88388	7.8642	253.354	72.6905
AAEL004440	tubulin-specific chaperone e	9.88373	8.64112	18.6106	21.131	8.86757	9.18862	74.7775	36.0014
AAEL014247	type i inositol-1,4,5-trisphosphate 5-phosphatase	17.138	15.055	9.13766	12.2568	14.4661	12.4862	0.872245	8.97505
AAEL008963	tyrosine aminotransferase	51.9278	63.4627	16.5358	15.2732	44.445	32.9396	0.748307	25.9454
AAEL007971	tyrosine transporter	31.446	26.4114	40.3515	43.6636	28.3761	29.2624	1.51532	19.9485
AAEL017826	U1	10.7718	37.9981	55.6523	36.3592	60.3519	33.8703	33.0355	33.1627
AAEL017860	U1	10.8578	4.13046	12.8151	2.89031	3.4431	7.40753	0	0

AAEL017607	U1	0	7.56105	0	0	0	3.35223	0	0
AAEL017799	U1	7.19551	8.19372	5.10634	17.2781	0	55.1209	0	32.999
AAEL014167	U1 small nuclear ribonucleoprotein A	19.25	21.2718	28.2674	26.7068	23.4831	23.8441	5.2429	13.4714
AAEL017631	U2	0	1.91077	1.24256	0	0	0	0	5.81772
AAEL012656	U2 small nuclear ribonucleoprotein a	12.5286	9.2132	10.6035	11.0238	7.28106	8.65251	4.25664	11.5872
AAEL006713	U2 snrnp auxiliary factor, small subunit	88.3743	99.07	120.954	122.053	66.8433	60.1099	49.8759	63.5911
AAEL017659	U3	8.00391	5.44501	9.35129	19.9817	8.88804	11.1108	7.03534	16.1947
AAEL005263	U3 small nucleolar ribonucleoprotein protein IMP3 (U3 snoRNP protein IMP3), putative	29.2754	30.3043	35.0767	33.297	37.9093	40.4449	12.3978	22.6446
AAEL006063	U3 small nucleolar ribonucleoprotein protein imp4	27.6563	28.8898	31.9082	26.7843	38.547	41.3828	5.73952	19.1609
AAEL004997	U3 small nucleolar ribonucleoprotein protein imp4	39.0026	42.5809	53.3388	46.4576	77.6261	77.9712	9.17393	25.1168
AAEL000422	U3 small nucleolar RNA-associated protein, putative	23.2349	22.5254	25.9908	20.4722	46.3814	48.3969	4.0631	12.1827
AAEL017678	U4	0	0	0	0	0	19.0143	0	0
AAEL017674	U5	156.598	116.144	173.687	120.892	377.15	410.402	139.187	278.203
AAEL017888	U5	0	0	77.6394	66.3027	125.665	185.379	64.2809	97.9397
AAEL017805	U5	0	0	58.2295	0	0	26.4827	0	0
AAEL011187	U520	118.384	107.343	128.587	122.987	125.007	125.379	16.8383	54.1904
AAEL000766	ubiquinol cytochrome C oxidoreductase-subunit 6.4kD-subunit, putative	186.472	295.594	172.633	196.763	357.569	366.067	690.3	572.666
AAEL007868	ubiquinol-cytochrome c reductase complex 14 kd protein	125.862	135.563	125.73	102.551	155.55	149.875	278.865	215.187
AAEL000178	ubiquinone/menaquinone biosynthesis methyltransferase	30.2815	32.3406	38.0779	36.8392	28.4887	27.8816	13.2033	22.0296
AAEL000795	ubiquitin	836.537	799.148	1067.72	1082.93	860.449	867.136	848.497	845.5
AAEL003877	ubiquitin	74.3399	65.5094	57.201	57.8971	93.1308	71.0859	734.725	335.873
AAEL012112	ubiquitin conjugating enzyme 7 interacting protein	8.23843	6.98222	6.43783	7.72873	6.15675	6.06021	0.224931	4.78193
AAEL010583	ubiquitin conjugating enzyme 7 interacting protein	20.0023	19.8987	26.1341	23.6347	17.8201	19.0955	0.2999	12.2737
AAEL012599	ubiquitin ligase E3	38.4857	39.5073	22.0205	24.0953	36.7082	35.0485	12.3291	26.0498
AAEL012603	ubiquitin ligase E3	22.5327	23.8389	14.6533	15.3382	19.1658	19.5281	5.8357	13.1853

AAEL011287	ubiquitin specific protease	55.0913	58.0085	60.5647	52.4211	61.5653	61.4141	4.9231	26.0592
AAEL005038	ubiquitin-conjugating enzyme E2	134.653	125.74	200.724	164.52	87.0011	96.3687	96.4491	104.631
AAEL010798	ubiquitin-conjugating enzyme E2 g	172.462	180.219	201.654	175.383	116.89	120.748	78.7809	135.273
AAEL009770	ubiquitin-conjugating enzyme E2 i	139.236	138.489	210.327	182.335	126.42	115.974	134.269	114.34
AAEL006073	ubiquitin-conjugating enzyme E2-230k	41.1353	42.7289	43.2773	37.8018	34.3671	37.1789	7.79758	20.6278
AAEL008837	ubiquitin-conjugating enzyme E2r	48.8138	49.8974	74.7223	63.7545	58.7843	60.9584	21.2751	31.1672
AAEL005968	Ubiquitin-fold modifier-conjugating enzyme 1	49.6551	52.7927	28.257	28.257	33.4731	36.3292	54.5668	58.529
AAEL013363	ubiquitously transcribed sex (x/y) chromosome tetratricopeptide repeat protein	17.1097	15.9388	8.51507	9.50737	9.44095	9.64861	6.9619	13.4675
AAEL008656	UDP-galactose transporter	61.4911	90.1075	20.5499	23.6091	34.8588	37.6807	60.2493	68.201
AAEL011242	UDP-glucose 6-dehydrogenase	18.8716	21.7526	20.2749	21.3243	26.4219	24.2702	62.0713	26.7562
AAEL005903	UDP-glucuronosyltransferase, putative	6.9033	3.78271	11.1336	24.2181	9.1019	8.79628	27.0354	17.658
AAEL001896	UDP-N-acetylglucosamine: polypeptide-N-acetylglucosaminyl transferase, putative	94.4893	90.5842	154.156	127.196	120.269	115.304	73.8706	77.7329
AAEL015238	UPF0729 protein	38.8415	35.8629	36.3217	40.0004	65.2413	65.3408	185.616	96.4281
AAEL012973	uracil phosphoribosyltransferase	17.5172	19.6497	25.1111	21.8484	21.2922	19.6337	7.53658	13.2888
AAEL009099	uridine cytidine kinase i	25.3985	26.584	20.2504	17.1868	21.1537	22.2724	13.1567	25.5086
AAEL000395	USP	39.1233	39.829	39.7505	38.8261	46.5762	45.059	17.9059	27.8487
AAEL005617	utp-glucose-1-phosphate uridylyltransferase 2	61.87	58.9966	145.534	169.036	88.1972	81.6403	41.537	49.203
AAEL008309	V-1 protein, putative	34.2	53.3189	56.188	35.5205	94.4846	82.5833	52.8742	56.231
AAEL000291	Vacuolar ATP synthase 16 kDa proteolipid subunit	196.163	193.894	236.293	206.105	177.392	192.048	1718.42	939.238
AAEL012113	vacuolar ATP synthase proteolipid subunit	49.9024	54.0354	58.8002	58.2329	65.9549	71.3225	588.994	300.486
AAEL011025	vacuolar ATP synthase subunit ac39	54.5166	55.5565	31.969	36.6808	65.7049	71.8765	422.093	222.685
AAEL015594	vacuolar ATP synthase subunit c	10.1353	8.30098	23.2653	16.8147	7.45373	8.80989	63.2901	28.5664
AAEL012035	vacuolar ATP synthase subunit e	82.772	83.1683	100.342	94.4843	73.2459	79.2132	746.115	410.904
AAEL002464	vacuolar ATP synthase subunit f	62.771	62.4053	60.7378	53.1708	52.6164	57.9743	549.902	333.925
AAEL012819	vacuolar ATP synthase subunit g	18.3779	17.102	23.2299	20.1102	17.0743	20.753	417.077	197.804
AAEL006516	vacuolar ATP synthase subunit h	34.1106	36.0606	31.7651	35.6771	30.5842	33.8544	276.734	149.602
AAEL010819	Vacuolar ATP synthase subunit H	51.9829	56.2579	46.9092	47.4295	50.2229	59.2286	557.182	313.216
AAEL007777	vacuolar ATP synthase subunit S1	60.7339	58.5537	75.6982	70.571	67.8147	73.1421	451.624	247.204

AAEL009104	vacuolar protein sorting-associated protein (vps13)	7.23148	7.85922	4.58995	7.13688	10.4637	9.35577	59.4232	28.2219
AAEL010508	vacuolar protein sorting-associated protein (vps13)	7.36298	6.74533	4.88389	5.57501	8.19148	7.18463	51.7312	22.2802
AAEL006390	vacuolar proton atpases	0.206573	0.079932	0.618983	0.523602	3.70569	3.7781	381.13	191.69
AAEL014053	vacuolar proton atpases	29.6764	32.0726	29.6614	34.7796	30.3089	29.5453	0.98855	13.1788
AAEL004486	valacyclovir hydrolase	12.8321	11.7189	19.9932	17.3332	15.3133	13.1497	193.618	97.3892
AAEL013093	valacyclovir hydrolase	4.00193	4.02242	2.0415	4.02899	2.57523	7.94743	13.6031	1.90159
AAEL006023	Vanin-like protein 1 precursor, putative	9.45427	9.52595	8.63993	11.2531	6.74802	5.90548	175.06	75.0626
AAEL010747	vasodilator-stimulated phosphoprotein	26.797	27.09	10.7779	17.608	34.8932	34.8309	7.96688	15.5543
AAEL006563	Vcp	6415.2	6034.95	3.94251	9.45168	2.47309	2.24234	142.462	2937.61
AAEL000793	venom allergen	0.060181	0	0	0.100269	0	0	11.5341	0
AAEL001749	ventrhoid transmembrane protein, putative	26.4776	23.5702	6.78709	11.7558	19.041	17.8764	5.05111	12.2526
AAEL005879	vinculin	18.8093	20.2426	18.0922	19.4152	16.295	16.8555	86.9578	36.5851
AAEL014223	vitellogenin receptor (VgR)	86.1687	105.539	43.0886	50.6493	121.541	111.07	0	59.2462
AAEL006966	vitellogenin, putative	9.0168	11.2601	14.1876	14.5606	18.8776	17.5995	144.8	70.8584
AAEL010434	vitellogenin-A1 precursor	0	0	4.78064	9.61987	3.62803	3.54284	284.744	7567.78
AAEL001872	voltage-dependent anion-selective channel	386.877	400.781	456.054	364.941	289.064	295.952	700.798	478.174
AAEL013277	voltage-gated sodium channel	11.8078	17.3627	8.02759	10.6788	8.612	10.6329	0	33151.7
AAEL011639	WAP four-disulfide core domain protein 2 precursor, putative	29.1543	37.0544	13.235	8.05812	29.7694	41.4447	0.682999	40.6099
AAEL003975	wdpeat protein	13.6396	15.5641	14.7762	12.7917	21.9262	22.4031	2.91452	9.17687
AAEL011535	wdpeat protein	10.8432	9.00598	9.6633	4.76734	12.1641	11.4807	3.34802	11.488
AAEL004063	wdpeat protein	14.6277	14.2282	19.4345	14.5278	32.4287	30.612	3.40798	9.65167
AAEL003539	wdpeat protein	35.8128	36.3076	51.2887	46.7774	25.7361	27.3203	13.6966	23.433
AAEL002802	wdpeat protein	17.2409	15.8762	24.7109	22.889	13.5384	14.0291	4.01466	9.14871
AAEL005041	wdpeat protein	20.7343	22.2337	16.8917	15.3921	32.8885	31.3533	7.64986	14.0052
AAEL010013	wdpeat protein	26.5512	25.9985	29.2401	29.4895	22.1671	23.3419	6.66448	16.2011
AAEL016999	white	25.5842	24.383	28.8232	29.0951	23.434	23.9288	1.09818	14.1881
AAEL013892	wiskott-aldrich syndrome protein	9.72792	10.2761	17.5357	18.0014	6.85449	6.85408	96.6119	32.8504
AAEL004320	WOC protein, putative	62.9318	72.9898	115.697	99.7205	59.2573	73.2505	80.5317	81.0811
AAEL004558	wuho	20.583	18.3986	25.7752	17.6336	19.0489	17.1946	5.82775	11.8317
AAEL007892	xaa-pro aminopeptidase	0.73742	0.697663	0.492829	0.379067	1.49625	1.22246	555.068	256.051
AAEL009764	xaa-pro aminopeptidase	9.30025	10.2076	7.87383	8.07018	12.6749	13.5468	258.924	115.707
AAEL008893	xaa-pro dipeptidase pepd/pepq(e.coli)	29.2614	28.5895	30.5609	37.578	21.4381	21.9066	307.862	169.478

AAEL010077	xpa-binding protein 1 (mbdin)	28.6089	29.4597	39.7148	32.9235	38.8463	39.6454	7.60364	20.5113
AAEL013821	xylulose kinase	40.0747	39.9637	5.32105	3.3587	13.2958	12.6151	16.2501	26.9551
AAEL006830	yellow protein precursor	236.535	172.774	280.062	242.297	106.925	109.99	2.3411	101.373
AAEL014001	yellow protein precursor, putative	19.5063	15.2823	42.0526	42.9911	13.7259	11.3232	0.247344	7.16594
AAEL008609	zinc carboxypeptidase	2.1512	2.81424	0.307258	0.275405	0.849825	1.71354	792.265	436.177
AAEL003781	zinc carboxypeptidase	4.73787	3.96898	3.31853	4.05631	4.1625	4.26085	69.7198	28.543
AAEL001863	zinc carboxypeptidase	312.067	276.378	74.7141	92.1887	262.994	334.839	13329.9	6037.16
AAEL003424	zinc carboxypeptidase	0.115213	0	0.112067	0.097507	1.13674	2.06023	149.032	47.2863
AAEL008600	zinc carboxypeptidase	0.07852	0.22699	0.146863	0.170639	2.61319	1.89183	148.613	70.8379
AAEL001839	zinc carboxypeptidase	0.223616	0.525831	0.23111	0.460135	0.321721	0.263161	69.7134	15.0542
AAEL001840	zinc carboxypeptidase	43.6701	45.5297	30.3398	41.8008	9.25998	8.79685	753.214	366.465
AAEL011328	zinc finger protein	32.4338	33.9113	19.8456	29.0511	26.0402	27.5817	10.0252	21.5494
AAEL002285	zinc finger protein	12.3608	13.4867	14.7683	11.8025	9.44031	13.6236	3.58454	10.2782
AAEL002451	zinc finger protein	6.91229	6.6843	6.7442	7.93232	7.20561	7.70287	0.235766	3.92499
AAEL009508	zinc finger protein	61.4798	59.4416	34.7304	47.654	38.6703	39.4991	4.11267	32.9683
AAEL002120	zinc finger protein	11.7523	13.0956	13.871	13.4081	8.24376	8.91186	2.03484	7.12738
AAEL007427	zinc finger protein	116.87	112.879	64.075	89.0094	73.1744	73.5665	4.47111	56.846
AAEL014034	zinc finger protein	41.5561	38.6321	77.7973	73.4725	34.2299	36.678	11.9442	24.401
AAEL000974	zinc finger protein	10.4548	10.1262	16.8285	14.5174	7.72793	7.07485	0.887104	7.51441
AAEL003009	zinc finger protein	49.5385	47.1842	52.4742	54.6815	37.923	40.3281	167.881	80.6894
AAEL008579	zinc finger protein	17.087	17.5264	15.263	16.4364	13.0884	14.1162	2.46703	7.73369
AAEL006609	zinc finger protein	14.69	11.3611	12.7239	13.1814	83.2784	50.7604	47.1489	22.4949
AAEL011904	zinc finger protein	25.5375	27.073	31.5829	31.264	20.6017	20.9463	12.0432	19.7876
AAEL012039	zinc finger protein	7.95965	7.84829	10.7043	10.424	13.5409	13.6811	0.298759	3.56799
AAEL012224	zinc finger protein	31.7072	27.574	39.3352	38.9291	19.7329	13.5109	22.6821	28.9508
AAEL004815	zinc finger protein	29.9121	30.5258	34.1442	30.2074	29.3652	28.7264	9.69208	17.2492
AAEL010501	zinc finger protein	17.7337	17.713	22.7426	19.5253	22.9492	21.6404	5.71697	11.4281
AAEL007468	zinc finger protein, putative	10.286	10.8134	15.3781	12.8936	11.5868	10.8168	2.44309	8.26388
AAEL001968	zinc transporter	78.8552	92.4864	59.5997	71.5316	25.4206	29.7587	44.93	72.0539
AAEL014762	zinc transporter	75.2093	70.1722	46.6038	46.7026	30.5506	36.4998	1126.83	540.1
AAEL017268	uncharacterized protein	34.5995	65.6331	13.8819	19.0807	18.0494	29.6005	28.1477	29.9126
AAEL017349	uncharacterized protein	537.98	603.679	309.222	399.453	264.415	325.477	469.877	475.566
AAEL017320	uncharacterized protein	0.407171	0.68932	7.3147	3.7075	2.4122	2.97298	189.222	96.3036
AAEL017502	uncharacterized protein	0	0	0.119664	146.768	0	0	0	0.295997
AAEL017407	uncharacterized protein	6.89859	12.5221	22.2355	23.73	10.588	11.9013	213.237	102.254

AAEL017443	uncharacterized protein	0.919705	1.03073	0.603134	0.585621	1.24456	0.90921	20.0951	6.25175
AAEL017146	uncharacterized protein	19.6778	11.2055	20.4646	9.47485	13.7339	13.2239	17.9262	28.2184
AAEL018037	uncharacterized protein	39.2491	34.1102	88.5434	71.0478	18.2942	19.2352	8.86734	23.2418
AAEL017385	uncharacterized protein	79.7074	81.3279	86.8986	78.752	70.3762	68.3422	44.343	64.9085
AAEL017144	uncharacterized protein	146.876	134.908	45.5254	51.8514	397.803	373.237	7.28154	17.0808
AAEL017467	uncharacterized protein	0.024146	0.206031	62.4241	335.249	0.086151	0.117016	0.362918	0.076718
AAEL017471	uncharacterized protein	0	0	0.534352	41.3375	0	0	0	0
AAEL016984	uncharacterized protein	447.87	430.432	681.493	584.116	131.169	137.834	756.275	503.863
AAEL017029	uncharacterized protein	57.502	61.4911	61.5295	51.5868	42.0671	38.6956	2.06167	42.1276
AAEL017054	uncharacterized protein	24.9154	23.4485	27.213	25.6748	16.3856	16.3586	9.59268	19.4289
AAEL017055	uncharacterized protein	4.95891	4.61477	5.70228	5.80029	4.40659	3.57234	0.064207	3.42214
AAEL017061	uncharacterized protein	0.911904	1.63465	1.78195	1.45088	3.79767	3.45431	230.838	136.533
AAEL017066	uncharacterized protein	321.734	337.666	530.058	453.254	458.208	466.796	417.241	335.043
AAEL017071	uncharacterized protein	1.72315	1.33091	0.985404	1.43657	2.70924	2.15237	75.004	33.4988
AAEL017082	uncharacterized protein	81.1417	82.1844	141.856	108.744	48.9616	49.7552	33.3234	56.5661
AAEL017089	uncharacterized protein	36.5801	34.939	17.2982	13.9926	18.3052	19.4941	202.293	100.58
AAEL017113	uncharacterized protein	48.9425	59.6005	34.0526	32.3121	64.9953	61.3071	13.5997	34.7303
AAEL017117	uncharacterized protein	0	0.103027	10.7894	475.962	0.129866	0	0.343367	0.251243
AAEL017168	uncharacterized protein	39.4268	41.1754	49.3474	38.3554	40.8355	38.1974	1.55511	22.9707
AAEL017179	uncharacterized protein	177.548	153.339	308.419	307.746	97.4454	109.515	3.84641	97.1788
AAEL017186	uncharacterized protein	11.5132	11.6701	30.0268	20.6634	5.00861	4.57193	1.39772	6.6036
AAEL017231	uncharacterized protein	1054.41	1190.29	914.151	760.363	948.343	1209.14	4917.27	2296.47
AAEL017258	uncharacterized protein	150.762	149.862	159.771	136.51	150.279	160.405	91.2704	108.785
AAEL017269	uncharacterized protein	128.729	102.555	243.679	232.534	55.9542	61.2558	4.49969	84.1543
AAEL017280	uncharacterized protein	30.134	29.6427	33.0664	31.3947	32.8439	33.637	14.5004	21.4456
AAEL017284	uncharacterized protein	4.1472	3.38643	1.42775	1.46513	4.12058	4.12582	169.138	68.6971
AAEL017291	uncharacterized protein	0.051164	0	0	0.042709	0	0	58.4813	26.2354
AAEL017329	uncharacterized protein	18.1509	16.9252	23.9813	38.1473	4.78791	5.35888	4.20648	10.5763
AAEL017334	uncharacterized protein	43.0674	1.03829	2.75874	1.85916	0.817861	20.2139	177.148	20.7884
AAEL017368	uncharacterized protein	91.962	94.797	73.8007	69.2092	85.5368	80.3338	66.0544	75.4679
AAEL017380	uncharacterized protein	32.1549	9.79262	23.8532	11.9188	417.249	174.555	1.8195	2.72489
AAEL017391	uncharacterized protein	58.3795	55.8071	42.6715	53.0009	45.5655	46.9643	13.9931	31.968
AAEL017395	uncharacterized protein	85.1884	85.1169	109.569	109.537	83.4129	83.2287	42.1756	60.0787
AAEL017413	uncharacterized protein	17702.6	16218.5	8467.18	11015.2	12940	15811.1	69543.8	34084.1
AAEL017438	uncharacterized protein	113.241	122.734	163.471	174.653	73.7677	70.8838	27.4413	59.5133

AAEL017451 uncharacterized protein	272.96	367.824	330.075	276.007	80.3489	83.808	5.85446	173.235
AAEL017455 uncharacterized protein	172.639	161.165	379.411	343.133	202.407	287.578	33.4343	99.2256
AAEL017457 uncharacterized protein	0.601374	0.62224	0.14962	0.403672	0.297512	0.77708	5043.51	2305.54
AAEL017473 uncharacterized protein	56.1546	55.628	62.7538	46.6417	54.505	56.9968	158.731	78.801
AAEL017499 uncharacterized protein	37.8122	38.2706	47.856	28.4439	36.2245	33.1372	40.191	31.1427
AAEL017514 uncharacterized protein	0	0	0.318357	0.123391	15.4085	1.49337	157.885	85.3355
AAEL017545 uncharacterized protein	285.129	250.395	515.349	394.687	149.68	148.121	189.677	184.215
AAEL017566 uncharacterized protein	20.0663	19.1216	14.9792	14.6954	14.898	15.6403	8.38098	14.6738
AAEL018032 uncharacterized protein	15.0412	13.9957	4.07371	6.80198	16.1454	15.336	60.6668	28.0377
AAEL018044 uncharacterized protein	0.340419	0.257842	0.184874	0.235176	1.12639	1.22866	46.6888	18.8929
AAEL017358 uncharacterized protein	177.956	123.32	378.276	303.764	7.46537	18.0909	1.79391	70.599
AAEL017251 uncharacterized protein	50.0269	46.4902	34.4714	40.8019	28.2901	30.2491	183.937	86.7293
AAEL017171 uncharacterized protein	9.36958	9.21081	18.999	79.1366	7.38683	5.9403	6.58	6.34723
AAEL017245 uncharacterized protein	6.94856	8.29117	22.1385	27.599	64.5168	57.9872	119.256	34.8655
AAEL017228 uncharacterized protein	37.3827	41.0169	98.0394	69.3781	112.386	100.815	234.267	147.778
AAEL017045 uncharacterized protein	16.2774	12.8929	10.7299	29.2169	9.45357	19.6824	0	1.70692
AAEL017425 uncharacterized protein	28.3536	23.1173	10.9683	13.5679	24.3186	12.3799	71.3467	27.8808
AAEL017198 uncharacterized protein	2363.83	2583.83	1053.28	1029	5395.91	5676.49	4539.97	3960.45
AAEL018031 uncharacterized protein	44.4652	45.3957	54.4724	51.3612	38.8651	40.3784	0.843808	13.8875
AAEL016960 uncharacterized protein	28.7772	29.2068	18.5037	21.7175	21.6693	20.9456	9.56199	16.8287
AAEL016962 uncharacterized protein	43.2847	44.2409	47.6953	47.6335	45.6503	46.126	14.5495	27.467
AAEL016982 uncharacterized protein	277.729	269.206	408.572	362.443	192.28	198.072	78.7002	185.385
AAEL016986 uncharacterized protein	61.0192	73.0207	62.1773	50.2128	80.845	65.1847	21.2067	46.6116
AAEL017022 uncharacterized protein	2.03158	2.40061	8.07923	9.60506	42.5458	43.7315	11831.4	4580.51
AAEL017075 uncharacterized protein	192.241	214.802	79.8473	85.0814	640.094	610.037	76.8832	136.292
AAEL017096 uncharacterized protein	2969.03	2993.94	1030.55	1022.69	6175.17	5975.41	3146.89	2739.13
AAEL017098 uncharacterized protein	1073.46	956.799	1813.54	1191.02	318.726	328.261	12.9188	616.392
AAEL017119 uncharacterized protein	607.49	728.123	270.25	325.984	1365.86	1615.05	13594.7	7295.45
AAEL017140 uncharacterized protein	7.84027	8.23959	8.15785	7.79213	9.60789	9.52363	80.9975	34.7632
AAEL017194 uncharacterized protein	41.7928	42.7037	54.7981	50.4367	35.3568	36.5622	7.40977	24.298
AAEL017197 uncharacterized protein	0	0	20.4214	1101.85	0	0.134699	0	0
AAEL017240 uncharacterized protein	61.8835	61.1618	71.7032	71.8722	50.6265	53.3882	16.512	37.275
AAEL017257 uncharacterized protein	0	0	137.687	587.944	0	0	0	0.488442
AAEL017317 uncharacterized protein	21.7346	10.2755	18.2759	16.883	14.6499	21.5307	10.4869	1.41571
AAEL017321 uncharacterized protein	76.1893	82.2699	67.241	78.4758	70.4939	70.1495	8.56926	48.8626

AAEL017337	uncharacterized protein	14.1553	17.0393	24.2888	17.506	22.7132	20.7775	0.445946	7.63146
AAEL017386	uncharacterized protein	17.4406	18.2791	32.2734	61.9993	7.69615	5.75483	53.0768	38.9922
AAEL017424	uncharacterized protein	20.9876	21.4453	44.2755	32.1976	20.2655	21.7133	7.70041	15.0357
AAEL017497	uncharacterized protein	90.2047	89.0154	74.6906	74.2665	102.388	100.388	19.2128	50.8626
AAEL017994	uncharacterized protein	1.46509	0.943043	4.38551	16.7445	2.54845	2.69302	0.093846	0.497504
AAEL018040	uncharacterized protein	12.0032	10.3341	15.3693	18.1673	23.6451	22.4166	0.720069	4.67186
AAEL017501	uncharacterized protein	939.27	771.472	0.261286	0.303021	0.33597	0	16.9795	486.211
AAEL017521	uncharacterized protein	22.7778	22.261	27.7357	24.2605	23.2052	22.1075	0.620645	13.1232
AAEL017226	uncharacterized protein	0.053516	0.101652	0.140535	0.163205	2.40608	2.36843	334.659	142.948
AAEL017437	uncharacterized protein	124.364	137.83	67.9145	74.9282	155.249	149.288	36.7684	76.3026
AAEL017543	uncharacterized protein	21.8998	19.0716	16.0082	17.9972	16.5823	17.494	9.18633	15.7819
AAEL017367	uncharacterized protein	94.1808	75.3883	148.676	192.808	16.3462	18.6446	10.9567	44.7707
AAEL017421	uncharacterized protein	110.021	116.032	70.3063	52.8287	134.401	129.371	23.7942	66.1583
AAEL017513	uncharacterized protein	31.5233	49.8467	20.9979	33.5769	46.806	38.6187	36.5477	24.7753
AAEL017485	uncharacterized protein	28.7523	24.8791	4.049	18.2675	11.6732	10.2385	38.9639	41.9055
AAEL017121	uncharacterized protein	77.324	76.411	24.8959	33.5122	19.3	21.1729	479.899	297.644
AAEL017481	uncharacterized protein	776.439	826.729	1253.69	990.317	750.11	757.246	226.203	481.179
AAEL017509	uncharacterized protein	673.729	1512.49	1928.05	1132.42	669.63	0	0	1273.04
AAEL016995	uncharacterized protein	1477.52	1464.25	1822.75	1473.9	2941.54	3093.49	1629.01	1294.51
AAEL017044	uncharacterized protein	21.9952	21.6512	23.4191	31.3641	13.4066	12.9108	5.43506	13.8228
AAEL017343	uncharacterized protein	20.9817	18.6122	14.0677	17.2523	18.5228	22.0036	1.3569	13.2962
AAEL017563	uncharacterized protein	19.1779	24.8613	3.3781	4.21184	5.45432	8.91127	0	10.5111
AAEL017093	uncharacterized protein	43.3429	39.1126	1.7105	7.34752	7.16694	8.51983	1.36911	24.004
AAEL017494	uncharacterized protein	147.805	178.599	81.8695	119.958	176.922	153.455	483.525	326.844
AAEL017282	uncharacterized protein	18.2475	16.4577	22.9953	21.6895	21.0162	21.2289	2.52256	7.95336
AAEL017512	uncharacterized protein	3.2481	2.32141	2.36906	2.85185	1.83609	2.91372	118.617	55.6205
AAEL018038	uncharacterized protein	15.8473	15.3403	13.3772	11.8104	16.8981	18.0413	121.643	59.8312
AAEL017057	uncharacterized protein	24.256	31.8374	34.1984	23.9638	15.9232	17.895	13.2536	24.8392
AAEL016992	uncharacterized protein	37.8019	30.5446	0.027742	0	0.032736	0.035401	0.268099	13.5904
AAEL017388	uncharacterized protein	194.341	312.594	86.64	68.2832	90.2643	96.7941	208.405	243.367
AAEL018035	uncharacterized protein	50.5051	47.1523	19.1131	29.4273	52.2895	53.2454	23.465	34.2387
AAEL017322	uncharacterized protein	16.1348	14.0478	16.0913	20.0784	18.3852	16.2661	383.716	183.038
AAEL017516	uncharacterized protein	987.205	1017.47	404.004	360.288	2751.4	2728.78	1737.38	1347.08
AAEL017042	uncharacterized protein	32.3302	31.7363	42.0402	24.5167	25.0764	25.7408	131.282	69.6093
AAEL017289	uncharacterized protein	9.169	7.57484	13.1769	11.1998	10.6174	10.4703	0.13826	4.96745

AAEL017244	uncharacterized protein	1.29596	1.83743	2.91744	1.40054	1.82289	1.98446	145.538	73.73
AAEL017404	uncharacterized protein	0.98589	0.731195	775.302	4272.28	0.134817	1.21088	1.34722	1.06441
AAEL017184	uncharacterized protein	30.7728	17.3486	60.5252	58.1521	22.3898	22.0994	12.6367	28.8257
AAEL017207	uncharacterized protein	41.8425	42.7477	33.8698	38.139	35.7736	35.1866	20.9049	28.7193
AAEL017540	uncharacterized protein	61.0066	71.7802	68.34	59.4597	22.3553	33.1598	822.639	340.797
AAEL017013	uncharacterized protein	16.792	16.6443	22.9625	25.7429	17.6545	15.544	4.60261	12.9745
AAEL017482	uncharacterized protein	0.088899	0	0.342707	0.078335	0.560192	0.863556	59.6393	27.9194
AAEL016974	uncharacterized protein	4.19249	6.0072	0.598941	1.97256	6.98012	5.92964	1.3177	6.53658
AAEL017069	uncharacterized protein	0.150017	0	2.16158	34.2035	0	0	0	0.262581
AAEL017506	uncharacterized protein	8.73404	7.44723	7.86569	9.67279	5.59876	6.95793	81.6002	35.3273
AAEL017247	uncharacterized protein	409.851	380.426	547.392	549.49	365.87	425.75	1553.29	878.09
AAEL017470	uncharacterized protein	22.1131	24.3038	25.0168	27.199	19.3262	19.6413	1.57245	12.5235
AAEL016979	uncharacterized protein	8.79346	6.76329	12.8864	11.2892	8.40155	7.50798	0.242814	4.08424
AAEL017191	uncharacterized protein	43.2032	45.4671	126.135	100.835	31.835	32.3004	1.82848	20.18
AAEL017445	uncharacterized protein	0.116366	0.050323	0.217963	0.102145	0.469032	0.44737	22.0268	7.35968
AAEL017112	uncharacterized protein	2.35996	8.03108	13.7247	15.6391	0	7.03628	0	8.37962
AAEL016971	uncharacterized protein	17.6584	11.8756	0.020769	0.515153	0.070071	0.017016	0.171508	7.57498
AAEL017525	uncharacterized protein	11.3113	25.8162	110.871	75.5101	65.1517	40.8242	25.0287	10.0199
AAEL017520	uncharacterized protein	32.9979	37.1689	52.3588	55.1793	43.4112	35.1208	0	27.605

Abbreivation: CC_1d_C, carcass 1 day PBM; CC_1d_I, carcass 1 day post DENV infected blood meal; CC_2d_C, carcass 2 days post normal blood meal; CC_2d_I, carcass 2 days post DENV infected blood meal; CC_7d_C, carcass 7 days post normal blood meal; CC_7d_I, carcass 2 days post DENV infected blood meal; MG_1d_C, midgut 1 day post normal blood meal; MG_1d_I, midgut 1 day post DENV infected blood meal.
