# Supplementary material for: Aedes aegypti HPX8C modulates immune responses against viral infection
Source: PLoS Negl Trop Dis. 2019 Apr 15;13(4):e0007287. doi: 10.1371/journal.pntd.0007287 (PMC6464178; doi:10.1371/journal.pntd.0007287)
Supplement: S2 Table — (PDF) [file pntd.0007287.s002.pdf]

S2_Table. Immune DEGs post DENV infection (p<0.05)

GeneNames	Name	Family name	Normal Blood Meal(value1)	DENV infected blood meal(value2)	log2(value1/value2)	log2(value1/value2) normalized	z-score	p-value	Sample
AAEL000598	CECD	Cecropin	8.79978	0	4.137467456	4.142424294	2.940538	0.003276	Carcass_1 day
AAEL000611	CECE	Cecropin	57.6952	0	6.850379393	6.855336231	7.786985	6.86E-15	Carcass_1 day
AAEL000625	CECF	Cecropin	12.6296	0	4.658737042	4.663693881	3.62353	0.000291	Carcass_1 day
AAEL000621	CECN	Cecropin	69.3293	11.326	2.613826759	2.618783598	6.794714	1.09E-11	Carcass_1 day
AAEL003857	DEFD	Defensin	23.135	7.37591	1.649184142	1.65414098	2.930289	0.003386	Carcass_1 day
AAEL004390	HPX8B	HPX	743.736	609.752	0.286568021	0.29152486	3.710474	0.000207	Carcass_1 day
AAEL004120	ML1	ML	77.7553	106.332	-0.451562911	-0.446606073	-2.08749	0.036844	Carcass_1 day
AAEL004112	TPX2	TPX	1025.42	912.16	0.16885613	0.173812969	2.651961	0.008003	Carcass_1 day
AAEL000627	CECA	Cecropin	0	23.6197	-5.561918736	-5.45516626	-4.95827	7.11E-07	Carcass_2 day
AAEL000598	CECD	Cecropin	23.0968	7.33895	1.654047411	1.760799887	3.1151	0.001839	Carcass_2 day
AAEL000625	CECF	Cecropin	2.2785	26.1567	-3.521024262	-3.414271786	-4.65124	3.3E-06	Carcass_2 day
AAEL015515	CECG	Cecropin	31.3471	56.638	-0.85343834	-0.746685864	-2.37653	0.017476	Carcass_2 day
AAEL006271	CuSOD2	SOD-Cu-Zn	645.142	454.918	0.504010222	0.610762699	6.974252	3.08E-12	Carcass_2 day
AAEL003841	DEFA	Defensin	24.1906	56.6106	-1.226625662	-1.119873185	-3.34028	0.000837	Carcass_2 day
AAEL004401	HPX7	HPX	17.6291	43.2112	-1.293446472	-1.186693996	-3.05681	0.002237	Carcass_2 day
AAEL004388	HPX8A	HPX	225.725	181.715	0.312888698	0.419641174	2.928736	0.003403	Carcass_2 day
AAEL004390	HPX8B	HPX	230.614	451.652	-0.969731499	-0.862979023	-7.604	2.87E-14	Carcass_2 day
AAEL004386	HPX8C	HPX	155.745	259.284	-0.735347334	-0.628594858	-4.36996	1.24E-05	Carcass_2 day
AAEL004120	ML1	ML	17.5121	7.87103	1.153727754	1.26048023	2.117597	0.034209	Carcass_2 day
AAEL009557	ML17	ML	746.298	311.119	1.262285317	1.369037793	14.74258	3.43E-49	Carcass_2 day
AAEL004112	TPX2	TPX	1824.5	1617.74	0.173521392	0.280273868	5.707015	1.15E-08	Carcass_2 day
AAEL003389	ATT	Attacin	0.413053	8.32031	-4.332238467	-4.321870948	-2.88417	0.003924	Carcass_7 day
AAEL000627	CECA	Cecropin	2.95653	22.1999	-2.908576357	-2.898208838	-4.04322	5.27E-05	Carcass_7 day
AAEL000598	CECD	Cecropin	66.9018	169.905	-1.344611377	-1.334243858	-6.75301	1.45E-11	Carcass_7 day
AAEL000611	CECE	Cecropin	518.766	403.119	0.363878146	0.374245664	3.924081	8.71E-05	Carcass_7 day
AAEL015515	CECG	Cecropin	229.9	177.97	0.369372396	0.379739915	2.647548	0.008108	Carcass_7 day
AAEL000621	CECN	Cecropin	348.352	410.676	-0.237454792	-0.227087273	-2.16533	0.030363	Carcass_7 day
AAEL003243	CLIPB13A	CLIP-SP	127.822	84.2719	0.601012608	0.611380127	3.053047	0.002265	Carcass_7 day
AAEL014349	CLIPB15	CLIP-SP	56.7061	36.9187	0.619152184	0.629519703	2.087166	0.036873	Carcass_7 day
AAEL005431	CLIPB37	CLIP-SP	19.5256	40.68	-1.058952798	-1.048585279	-2.72849	0.006363	Carcass_7 day
AAEL003632	CLIPB39	CLIP-SP	8.77276	2.05816	2.09167566	2.102043179	2.123777	0.033689	Carcass_7 day
AAEL011453	CTL14	CTL	79.2079	37.8532	1.065229062	1.075596581	3.901209	9.57E-05	Carcass_7 day

AAEL011610	CTLGA8	CTL	46.5348	76.6135	-0.719288624	-0.708921105	-2.68505	0.007252	Carcass_7 day
AAEL003841	DEFA	Defensin	194.688	646.266	-1.73096413	-1.720596611	-15.8876	7.72E-57	Carcass_7 day
AAEL003832	DEFC	Defensin	72.6505	121.426	-0.741032736	-0.730665217	-3.47098	0.000519	Carcass_7 day
AAEL003857	DEFD	Defensin	259.918	585.462	-1.171518987	-1.161151468	-11.2442	2.47E-29	Carcass_7 day
AAEL008646	FREP10	FREP	18.0602	33.1618	-0.876708448	-0.866340929	-2.10063	0.035673	Carcass_7 day
AAEL009842	GALE12	GALE	46.7361	12.2741	1.928920108	1.939287626	4.651826	3.29E-06	Carcass_7 day
AAEL004522	GAM1	Gambicin	2.97939	29.1755	-3.291668492	-3.281300973	-4.91075	9.07E-07	Carcass_7 day
AAEL003723	LYSC11	LYSC	61.7785	103.655	-0.746612962	-0.736245443	-3.22828	0.001245	Carcass_7 day
AAEL009760	ML21	ML	8.87323	36.3369	-2.033904075	-2.023536556	-4.20278	2.64E-05	Carcass_7 day
AAEL009474	PGRPS1	PGRP	35.5475	69.8153	-0.973795136	-0.963427617	-3.33303	0.000859	Carcass_7 day
AAEL007765	SRPN10D	SRPN-INHIB	102.03	49.8842	1.032338569	1.042706088	4.316907	1.58E-05	Carcass_7 day
AAEL008607	tep3	TEP	90.4102	63.3978	0.512052768	0.522420287	2.228089	0.025875	Carcass_7 day
AAEL002309	TPX4	TPX	45.3366	19.3679	1.227008665	1.237376184	3.30147	0.000962	Carcass_7 day
AAEL003439	CASPS18	CASP	28.2583	9.5937	1.558515445	1.107640787	2.216335	0.026669	Midgut_1 day
AAEL003444	CASPS19	CASP	73.7674	27.1631	1.44133525	0.990460592	3.267806	0.001084	Midgut_1 day
AAEL014658	CASPS20	CASP	292.058	121.807	1.261657859	0.810783201	5.491252	3.99E-08	Midgut_1 day
AAEL014348	CASPS8	CASP	119.074	46.9911	1.341398988	0.89052433	3.798104	0.000146	Midgut_1 day
AAEL013407	CAT1	CAT	1285.66	660.103	0.961746104	0.510871446	7.648842	2.03E-14	Midgut_1 day
AAEL004223	CECB	Cecropin	9317.64	5138.19	0.858704352	0.407829693	16.77175	3.93E-63	Midgut_1 day
AAEL000598	CECD	Cecropin	41.6176	5.40842	2.943914674	2.493040016	4.761781	1.92E-06	Midgut_1 day
AAEL000611	CECE	Cecropin	26.2734	0	5.715531004	5.264656346	4.942459	7.71E-07	Midgut_1 day
AAEL000625	CECF	Cecropin	278.3	28.5072	3.28724266	2.836368002	13.20063	8.7E-40	Midgut_1 day
AAEL000621	CECN	Cecropin	129.503	32.8904	1.97724706	1.526372402	6.080778	1.2E-09	Midgut_1 day
AAEL002601	CLIPA1	CLIP-SPH	0.598246	4.96649	-3.053415856	-3.504290514	-2.26873	0.023285	Midgut_1 day
AAEL002590	CLIPA12	CLIP-SPH	1.54576	9.0413	-2.548213886	-2.999088544	-2.85946	0.004244	Midgut_1 day
AAEL002595	CLIPA14	CLIP-SPH	8.85063	38.0838	-2.105325381	-2.556200039	-5.40104	6.63E-08	Midgut_1 day
AAEL002301	CLIPA5	CLIP-SPH	12.6123	20.5642	-0.705303556	-1.156178215	-2.28799	0.022138	Midgut_1 day
AAEL002629	CLIPA6	CLIP-SPH	3.60168	28.1524	-2.966516005	-3.417390664	-5.34752	8.92E-08	Midgut_1 day
AAEL000074	CLIPB1	CLIP-SP	1.87514	20.3812	-3.442168779	-3.893043437	-4.77317	1.81E-06	Midgut_1 day
AAEL003243	CLIPB13A	CLIP-SP	1.10833	28.8132	-4.700270489	-5.151145148	-6.03838	1.56E-09	Midgut_1 day
AAEL014140	CLIPB24	CLIP-SP	0.464783	4.80153	-3.368864985	-3.819739643	-2.3022	0.021324	Midgut_1 day
AAEL007993	CLIPB27	CLIP-SP	50.7676	10.8393	2.227636469	1.77676181	4.243565	0.000022	Midgut_1 day
AAEL013245	CLIPB28	CLIP-SP	0.214443	3.27303	-3.931960698	-4.382835356	-1.97821	0.047905	Midgut_1 day
AAEL000099	CLIPB33	CLIP-SP	8.85333	19.6862	-1.152892554	-1.603767212	-2.8735	0.004059	Midgut_1 day
AAEL000028	CLIPB34	CLIP-SP	8.32754	53.2031	-2.675548026	-3.126422685	-7.07319	1.51E-12	Midgut_1 day

AAEL000037	CLIPB35	CLIP-SP	0.364572	4.38974	-3.589859825	-4.040734483	-2.24113	0.025018	Midgut_1 day
AAEL006168	CLIPB42	CLIP-SP	0.757815	8.53239	-3.493032309	-3.943906967	-3.10125	0.001927	Midgut_1 day
AAEL006371	CLIPB47-76		55.6895	19.1842	1.537486735	1.086612077	3.063444	0.002188	Midgut_1 day
AAEL005064	CLIPB5	CLIP-SP	1.87371	17.5188	-3.224934372	-3.67580903	-4.33867	1.43E-05	Midgut_1 day
AAEL003625	CLIPB8	CLIP-SP	2.69887	10.2944	-1.931432355	-2.382307013	-2.69709	0.006995	Midgut_1 day
AAEL012713	CLIPC16	CLIP-SP	2.85629	7.66943	-1.424976895	-1.875851553	-2.0012	0.04537	Midgut_1 day
AAEL007593	CLIPC2	CLIP-SP	0.801272	5.62634	-2.811832769	-3.262707427	-2.3444	0.019058	Midgut_1 day
AAEL007597	CLIPC3	CLIP-SP	1.74513	17.2105	-3.301882595	-3.752757253	-4.33219	1.48E-05	Midgut_1 day
AAEL004540	CLIPC6	CLIP-SP	0.213504	8.40692	-5.29924234	-5.750116999	-3.282	0.001031	Midgut_1 day
AAEL002124	CLIPD6	CLIP-SPH	2.16265	9.16864	-2.083907552	-2.53478221	-2.63764	0.008349	Midgut_1 day
AAEL010773	CLIPE10	CLIP-SPH	0.208505	3.80932	-4.1913796	-4.642254259	-2.16108	0.030689	Midgut_1 day
AAEL005644	CLIPE12	CLIP-SPH	0.133905	3.16567	-4.563229135	-5.014103793	-1.99508	0.046034	Midgut_1 day
AAEL011446	CTL17	CTL	0.477066	13.6996	-4.843801089	-5.294675748	-4.17457	2.99E-05	Midgut_1 day
AAEL011407	CTL20	CTL	51.6562	19.2501	1.424075578	0.973200919	2.694929	0.00704	Midgut_1 day
AAEL011408	CTL21	CTL	0.410259	7.82736	-4.253918911	-4.70479357	-3.1057	0.001898	Midgut_1 day
AAEL005641	CTLGA5	CTL	2195.88	792.114	1.471019235	1.020144577	18.27783	1.24E-74	Midgut_1 day
AAEL009209	CTLGA6	CTL	5.34436	14.3807	-1.428044805	-1.878919464	-2.74334	0.006082	Midgut_1 day
AAEL000543	CTLMA11	CTL	2.91455	7.66042	-1.394150342	-1.845025	-1.9777	0.047963	Midgut_1 day
AAEL011455	CTLMA12	CTL	0	6.52511	-3.706002223	-4.156876881	-2.75491	0.005871	Midgut_1 day
AAEL006271	CuSOD2	SOD-Cu-Zn	16.8768	121.368	-2.846274803	-3.297149461	-10.9382	7.57E-28	Midgut_1 day
AAEL003841	DEFA	Defensin	63.1225	14.9364	2.079321876	1.628447218	4.449764	8.6E-06	Midgut_1 day
AAEL003857	DEFD	Defensin	113.328	32.7201	1.79225529	1.341380632	5.161794	2.45E-07	Midgut_1 day
AAEL003849	DEFE	Defensin	204.207	391.537	-0.939116327	-1.389990985	-11.5271	9.64E-31	Midgut_1 day
AAEL007563	DUOX	HPX	0.461091	14.6599	-4.990679946	-5.441554604	-4.32667	1.51E-05	Midgut_1 day
AAEL007942	FREP14	FREP	292.798	159.535	0.876032731	0.425158073	3.082423	0.002053	Midgut_1 day
AAEL006704	FREP18	FREP	3143.42	1286.33	1.289074242	0.838199584	18.54863	8.37E-77	Midgut_1 day
AAEL006691	FREP21	FREP	197.46	111.805	0.820575724	0.369701066	2.222341	0.02626	Midgut_1 day
AAEL003294	FREP3	FREP	2.46422	12.2444	-2.312919113	-2.763793772	-3.1942	0.001402	Midgut_1 day
AAEL006699	FREP34	FREP	169.058	84.7983	0.99541104	0.544536382	2.938345	0.0033	Midgut_1 day
AAEL011400	FREP36	FREP	0.154572	6.04485	-5.28935563	-5.740230288	-2.78298	0.005386	Midgut_1 day
AAEL011007	FREP37	FREP	73.8202	34.3819	1.102366374	0.651491715	2.280311	0.022589	Midgut_1 day
AAEL004156	FREP9	FREP	606.46	252.965	1.261474705	0.810600046	7.912001	2.53E-15	Midgut_1 day
AAEL009842	GALE12	GALE	459.574	223.576	1.039531852	0.588657194	5.197688	2.02E-07	Midgut_1 day
AAEL012135	GALE2	GALE	225.778	76.6082	1.559334187	1.108459528	6.268753	3.64E-10	Midgut_1 day

AAEL003844	GALE5	GALE	641.786	210.874	1.605711092	1.154836433	10.92406	8.85E-28	Midgut_1 day
AAEL005293	GALE8A	GALE	423.935	161.78	1.389809814	0.938935156	7.493501	6.71E-14	Midgut_1 day
AAEL004522	GAM1	Gambicin	1085.42	696.837	0.639360263	0.188485605	2.741901	0.006108	Midgut_1 day
AAEL009176	GNBPB3	GNBP	128.26	55.5693	1.20671134	0.755836682	3.424711	0.000615	Midgut_1 day
AAEL009178	GNBPB4	GNBP	1285.41	526.139	1.288712702	0.837838044	11.85166	2.11E-32	Midgut_1 day
AAEL012069	GPXH1	GPX	355.3	139.03	1.353641466	0.902766808	6.637251	3.2E-11	Midgut_1 day
AAEL004401	HPX7	HPX	1.95119	114.061	-5.869307507	-6.320182165	-12.0377	2.25E-33	Midgut_1 day
AAEL004388	HPX8A	HPX	1.76198	89.3823	-5.664719715	-6.115594374	-10.6833	1.22E-26	Midgut_1 day
AAEL004390	HPX8B	HPX	7.70715	342.482	-5.473686886	-5.924561544	-20.941	2.26E-97	Midgut_1 day
AAEL004386	HPX8C	HPX	3.91822	182.362	-5.540462922	-5.991337581	-15.2751	1.12E-52	Midgut_1 day
AAEL009074	IAP1	IAP	376.425	185.28	1.022655288	0.57178063	4.58247	4.6E-06	Midgut_1 day
AAEL006633	IAP2	IAP	15.1446	21.7545	-0.522510385	-0.973385043	-2.04499	0.040856	Midgut_1 day
AAEL014251	IAP5	IAP	4.76864	21.614	-2.180316311	-2.63119097	-4.13416	3.56E-05	Midgut_1 day
AAEL003723	LYSC11	LYSC	677.037	273.312	1.308685875	0.857811217	8.77465	1.71E-18	Midgut_1 day
AAEL009531	ML	niemann-pick C1	360.97	146.528	1.300702565	0.849827907	6.355751	2.07E-10	Midgut_1 day
AAEL004120	ML1	ML	3.891	34.7942	-3.160633951	-3.61150861	-6.07482	1.24E-09	Midgut_1 day
AAEL006854	ML13	ML	6.35291	16.8773	-1.409594637	-1.860469295	-2.95219	0.003155	Midgut_1 day
AAEL015138	ML18	ML	27.9542	6.77578	2.04460612	1.593731462	2.915545	0.003551	Midgut_1 day
AAEL009760	ML21	ML	852.833	441.688	0.949235627	0.498360968	6.089601	1.13E-09	Midgut_1 day
AAEL015136	ML6	ML	13474.1	5842.49	1.205533647	0.754658989	35.16923	5.9E-271	Midgut_1 day
AAEL004823	MnSOD1	SOD-Mn-Fe	55.5407	71.4777	-0.363947852	-0.814822511	-3.18948	0.001425	Midgut_1 day
AAEL010171	PGRPLB	PGRP	472.865	100.586	2.232998843	1.782124185	12.97904	1.61E-38	Midgut_1 day
AAEL011608	PGRPLD	PGRP	5.57977	25.3584	-2.184186161	-2.635060819	-4.48155	7.41E-06	Midgut_1 day
AAEL009474	PGRPS1	PGRP	1481.35	527.785	1.488890294	1.038015636	15.22566	2.39E-52	Midgut_1 day
AAEL011763	PPO3	PPO	2.50916	8.08382	-1.687832729	-2.138707387	-2.23815	0.025211	Midgut_1 day
AAEL013492	PPO5	PPO	13.081	49.6011	-1.922899281	-2.373773939	-5.90782	3.47E-09	Midgut_1 day
AAEL007624	REL2	REL	43.2877	18.0231	1.26410997	0.813235312	2.119435	0.034054	Midgut_1 day
AAEL009192	SCRASP1	SCRA	0.745117	7.76252	-3.380986197	-3.831860855	-2.93034	0.003386	Midgut_1 day
AAEL009420	SCRBQ1	SCRB	46.6039	20.6352	1.175343269	0.724468611	1.989451	0.046651	Midgut_1 day
AAEL009423	SCRBQ2	SCRB	149.129	59.1912	1.333106225	0.882231566	4.216998	2.48E-05	Midgut_1 day
AAEL009432	SCRBQ3	SCRB	461.449	208.451	1.146462915	0.695588256	6.041365	1.53E-09	Midgut_1 day
AAEL000499	SPZ1A	SPZ	0.585263	24.9404	-5.413255718	-5.864130377	-5.65222	1.58E-08	Midgut_1 day
AAEL013433	SPZ1C	SPZ	0	5.0641	-3.340305894	-3.791180552	-2.35827	0.01836	Midgut_1 day
AAEL007765	SRPN10	SRPN-INHIB	2580.87	948.826	1.443642025	0.992767367	19.37712	1.2E-83	Midgut_1 day
AAEL003686	SRPN11	SRPN-nonINHIB	3.34451	22.7916	-2.768635443	-3.219510102	-4.69104	2.72E-06	Midgut_1 day

AAEL014078	SRPN2	SRPN-INHIB	0.551747	4.41461	-3.000207206	-3.451081864	-2.12601	0.033503	Midgut_1	day
AAEL002720	SRPN20	SRPN-INHIB	1.9728	16.7124	-3.082602318	-3.533476976	-4.17527	2.98E-05	Midgut_1	day
AAEL005665	SRPN3	SRPN-INHIB	2.57187	19.5905	-2.929264593	-3.380139252	-4.44079	8.96E-06	Midgut_1	day
AAEL013936	SRPN4	SRPN-INHIB	4.54609	22.732	-2.32202647	-2.772901128	-4.35969	0.000013	Midgut_1	day
AAEL014141	SRPN5	SRPN-INHIB	1.47714	9.86984	-2.740220129	-3.191094788	-3.07485	0.002106	Midgut_1	day
AAEL011777	SRPN8	SRPN-INHIB	1.57357	7.46817	-2.24671341	-2.697588069	-2.46293	0.013781	Midgut_1	day
AAEL008364	SRPN9	SRPN-INHIB	3.87512	39.1773	-3.337705082	-3.788579741	-6.55782	5.46E-11	Midgut_1	day
AAEL000087	TEP22	TEP	1.11661	9.34807	-3.065543156	-3.516417814	-3.11679	0.001828	Midgut_1	day
AAEL013441	TOLL9A	TOLL	141.476	57.0731	1.309674503	0.858799844	4.014541	5.96E-05	Midgut_1	day
AAEL011734	TOLL9B	TOLL	159.882	73.1356	1.128361786	0.677487128	3.474173	0.000512	Midgut_1	day
AAEL004112	TPX2	TPX	676.161	638.276	0.083186404	-0.367688254	-4.64838	3.35E-06	Midgut_1	day
AAEL002309	TPX4	TPX	1149.41	375.587	1.613674472	1.162799814	14.70139	6.32E-49	Midgut_1	day
