# Supplementary material for: Aedes aegypti HPX8C modulates immune responses against viral infection
Source: PLoS Negl Trop Dis. 2019 Apr 15;13(4):e0007287. doi: 10.1371/journal.pntd.0007287 (PMC6464178; doi:10.1371/journal.pntd.0007287)
Supplement: S3 Table — (PDF) [file pntd.0007287.s003.pdf]

S3_Table. Primers used for qPCR and synthesis of dsRNAs

Seq	Direction	GeneID	GeneName	Primer use
TCAGTGTACAAGAAGCTGACCGGA	Left	AAEL00949	RpS7	qPCR
TTCCGCGCGCTCACTTATTAGATT	Right		RpS7	qPCR
CAGGCTATGGCACTGTCACGAT	Left	AF204178.1	DENV-E	qPCR
CCATTTGCAGCAACACCATCTC	Right		DENV-E	qPCR
CTGTTCACTTTCGTCTTTCTGC	left	AAEL00338	Attacin	qPCR
AGGAAGCCTCCATGTTCTCAT	right		Attacin	qPCR
TGGCTGTTCTTCTCCTGACC	left	AAEL00062	CecropinA	qPCR
CCCCAGCTACAACAGGAAGA	right		CecropinA	qPCR
GCCAAAACCTGTTCCTCTTG	Left	AAEL00452	Gambicin	qPCR
CGATGTAGCATTCGGTGATG	Right		Gambicin	qPCR
GTTCACGCACAGTTTGACGC	left	AAEL00440	HPX7	qPCR
TGTTCCAGGATTCCGCCTAC	right		HPX7	qPCR
GCTGCTTCCGTTGAGTCGTA	left	AAEL00438	HPX8A	qPCR
CCCAGTGGAGGTTGATGTGA	right		HPX8A	qPCR
CCTCACTGACCCTCATTCCG	left	AAEL00439	HPX8B	qPCR
GATGCCAGATCCGATCCAAA	right		HPX8B	qPCR
ACGGGTATGCTACGAAGGTG	left	AAEL00438	HPX8C	qPCR
GCAAGATCGGCAAGAACTCA	right		HPX8C	qPCR
CAAATCGCATTCGATCCTTC	left	AAEL0134	CAT1	qPCR
GGGCCATCACGTTGATAGTT	right		CAT1	qPCR
ACACGGTCGTTTCATTGTTCG	left	AAEL00627	CuSOD2	qPCR
CACTGCATAGCTGCTCTGGTT	right		CuSOD2	qPCR
ATGGGCATGGCGTCACAG	left	AAEL00756	DUOX	qPCR
TCGGGCAGTCCGTTGTCT	right		DUOX	qPCR
GATGCCGATGAACTGAAGCG	left	AAEL00663	IAP2	qPCR
GTTCCCATTGACCGATGACG	right		IAP2	qPCR
GAAGATGCATCGCAATCTGTA	left	AAEL01509	PIAS	qPCR
GGAATGGTTGGCGTACTAGC	right		PIAS	qPCR
CAGCATCGTCGGCTTCATCC	Left	AAEL00969	STAT	qPCR
TTGACCCAGGCGATTGTGAT	Right		STAT	qPCR
GCAGATGAAGTCCAAGGAGC	Left	AAEL0007	Cactus	qPCR
GATCACGGCAAGGTGTAGGT	Right		Cactus	qPCR
GACAGCCACATTCCGACC	Left	AAEL00762	Rel2	qPCR

CTCCAGCTTGGACTCCTCC	Right		Rel2	qPCR
TTGGAAGACGCACTAAAGGAA	Left	AAEL00357	Caspar	qPCR
CACCCGTACAGCACAAAGTG	Right		Caspar	qPCR
GGCTGCAAAAAGGGAGTCTG	left	AAEL00769	Rel1	qPCR
AGATCGATGGTGGCAGGCT	right		Rel1	qPCR
ATACTTGCCGAAAGTGGTGG	left	AAEL00440	HPX7RNAi	synthesis dsRNA from cDNA
CGCAAACTGATCGCTAATGA	right		HPX7RNAi	synthesis dsRNA from cDNA
TAATACGACTCACTATAGGGATACTTGCCGAAAGTGG	left	AAEL00440	HPX7RNAi	synthesis dsRNA from cDNA
TAATACGACTCACTATAGGGCGCAAACTGATCGCTAA	right		HPX7RNAi	synthesis dsRNA from cDNA
ATGGCACTCAAGCGAACTCT	left	AAEL00438	HPX8ARNAi	synthesis dsRNA from cDNA
GCCATGTCGCAGTAGTCTGA	right		HPX8ARNAi	synthesis dsRNA from cDNA
TAATACGACTCACTATAGGGATGGCACTCAAGCGAAC	left	AAEL00438	HPX8ARNAi	synthesis dsRNA from cDNA
TAATACGACTCACTATAGGGGCCATGTCGCAGTAGTC	right		HPX8ARNAi	synthesis dsRNA from cDNA
GTACAGCAATTGGGCTTGGT	left	AAEL00439	HPX8BRNAi	synthesis dsRNA from cDNA
TGTTTGCGCAGATTATAGCG	right		HPX8BRNAi	synthesis dsRNA from cDNA
TAATACGACTCACTATAGGGGTACAGCAATTGGGCTT	left	AAEL00439	HPX8BRNAi	synthesis dsRNA from cDNA
TAATACGACTCACTATAGGGTGTTTGCGCAGATTATA	right		HPX8BRNAi	synthesis dsRNA from cDNA
ACGGGTATGCTACGAAGGTG	left	AAEL00438	HPX8CRNAi	synthesis dsRNA from cDNA
GTATGGTCGAACGGCAAAGT	right		HPX8CRNAi	synthesis dsRNA from cDNA
TAATACGACTCACTATAGGGACGGGTATGCTACGAAG	left	AAEL00438	HPX8CRNAi	synthesis dsRNA from cDNA
TAATACGACTCACTATAGGGGTATGGTCGAACGGCAA	right		HPX8CRNAi	synthesis dsRNA from cDNA
TGAACGGTGCATTCAAAGAA	left	AAEL00411	TPX2RNAi	synthesis dsRNA from cDNA
TATTCCTTCGACTTTTGCGG	right		TPX2RNAi	synthesis dsRNA from cDNA
TAATACGACTCACTATAGGGTGAACGGTGCATTCAAA	left	AAEL00411	TPX2RNAi	synthesis dsRNA from cDNA
TAATACGACTCACTATAGGGTATTCCTTCGACTTTTGC	right		TPX2RNAi	synthesis dsRNA from cDNA
