# Supplementary figures and images for: Aedes aegypti HPX8C modulates immune responses against viral infection
Source: PLoS Negl Trop Dis. 2019 Apr 15;13(4):e0007287. doi: 10.1371/journal.pntd.0007287 (PMC6464178; doi:10.1371/journal.pntd.0007287)

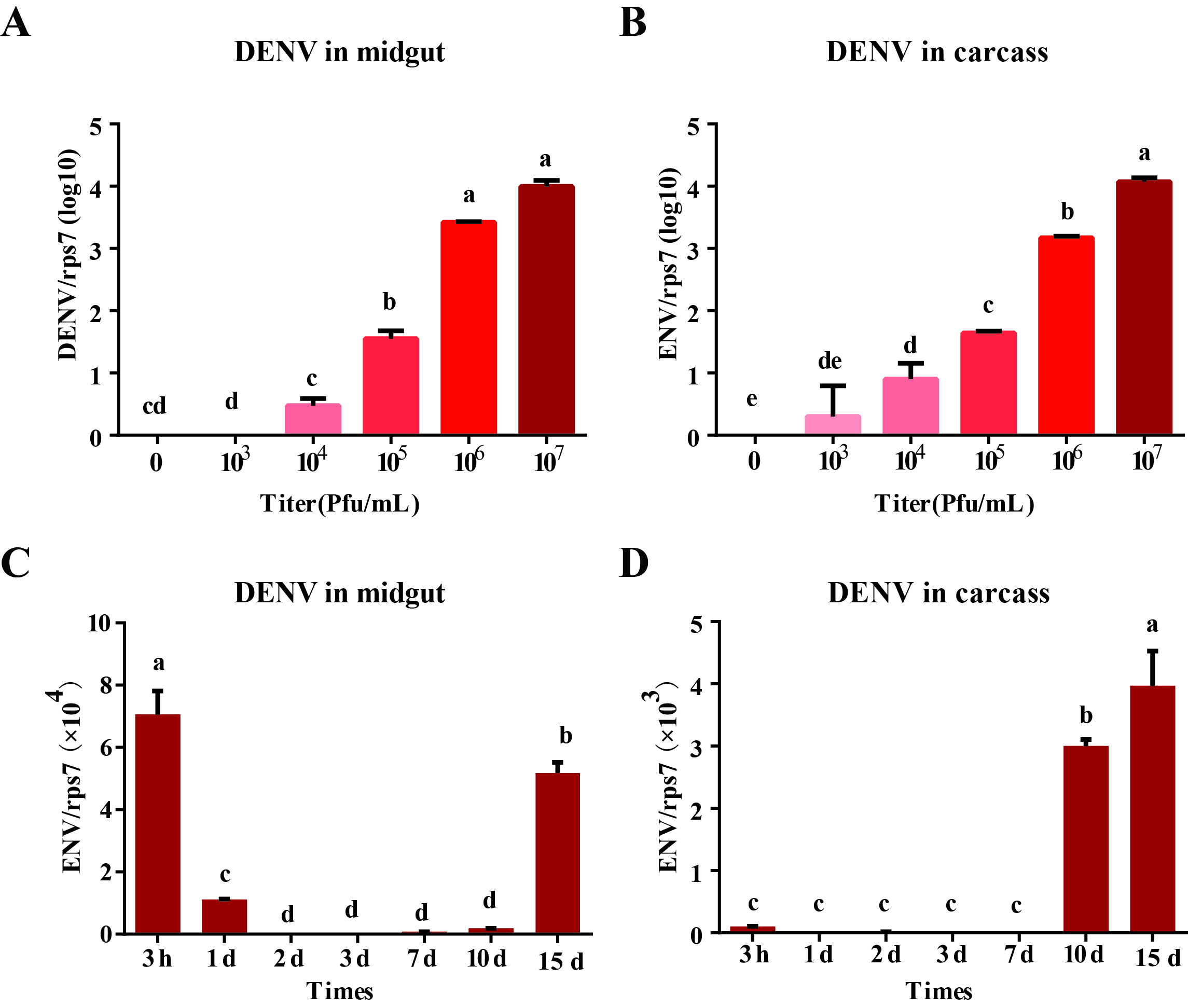

Supplement: S1 Fig — A, The DENV titer dynamics in the midgut 10 d.p.i. (post-infection). B, The DENV titer dynamics in the carcass 10 d.p.i. C, The DENV titer dynamics in the midgut post infection. D, DENV titer dynamics in the carcass post infection. Each treatment group comprised 30 mosquitoes. Identical letters are not significant difference (p > 0.05), while different letters indicate significant difference (p < 0.05) determined by one way ANOVA followed by a Tukey’s multiple comparison test. All experiments were repeated in triplicate. Data are represented as mean ± SEM. (TIF) [file pntd.0007287.s004.tif]

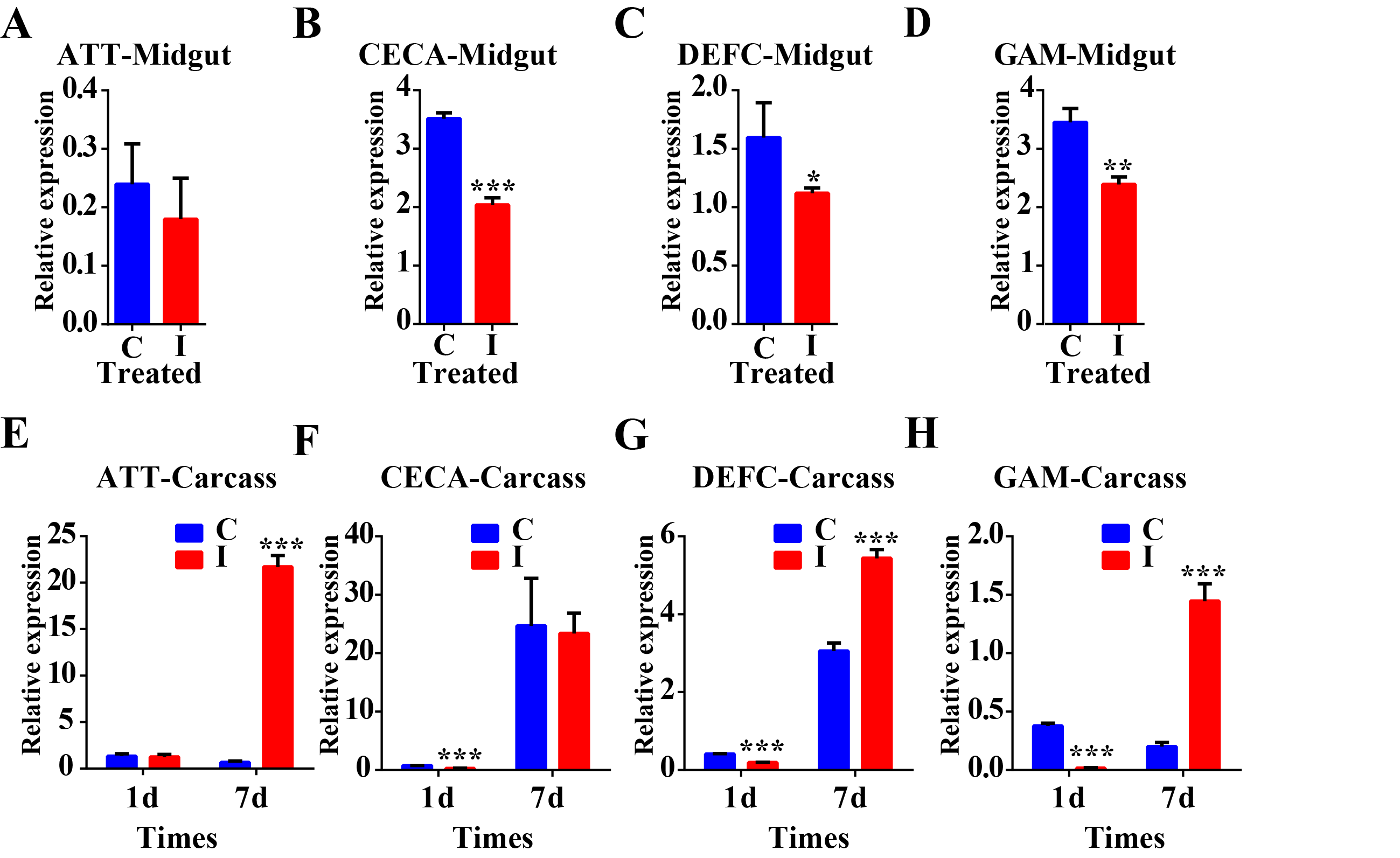

Supplement: S2 Fig — The mRNA levels of attacin (ATT) (A), cecropin A (CECA) (B), defencin C (DEFC) (C) and gambicin 1 (D) 1 day post DENV infection in the midgut. The mRNA levels of attacin (ATT) (E), cecropin A (CECA) (F), defencin C (G) and gambicin 1 (H) 1 day post DENV infection in the carcass. All experiments were repeated in triplicate. Student’s t-tests were used to determine the significance of difference in expression between treated and control groups. Data are represented as mean ± SEM. * p<0.05, ** p<0.01, *** p<0.001. (TIF) [file pntd.0007287.s005.tif]

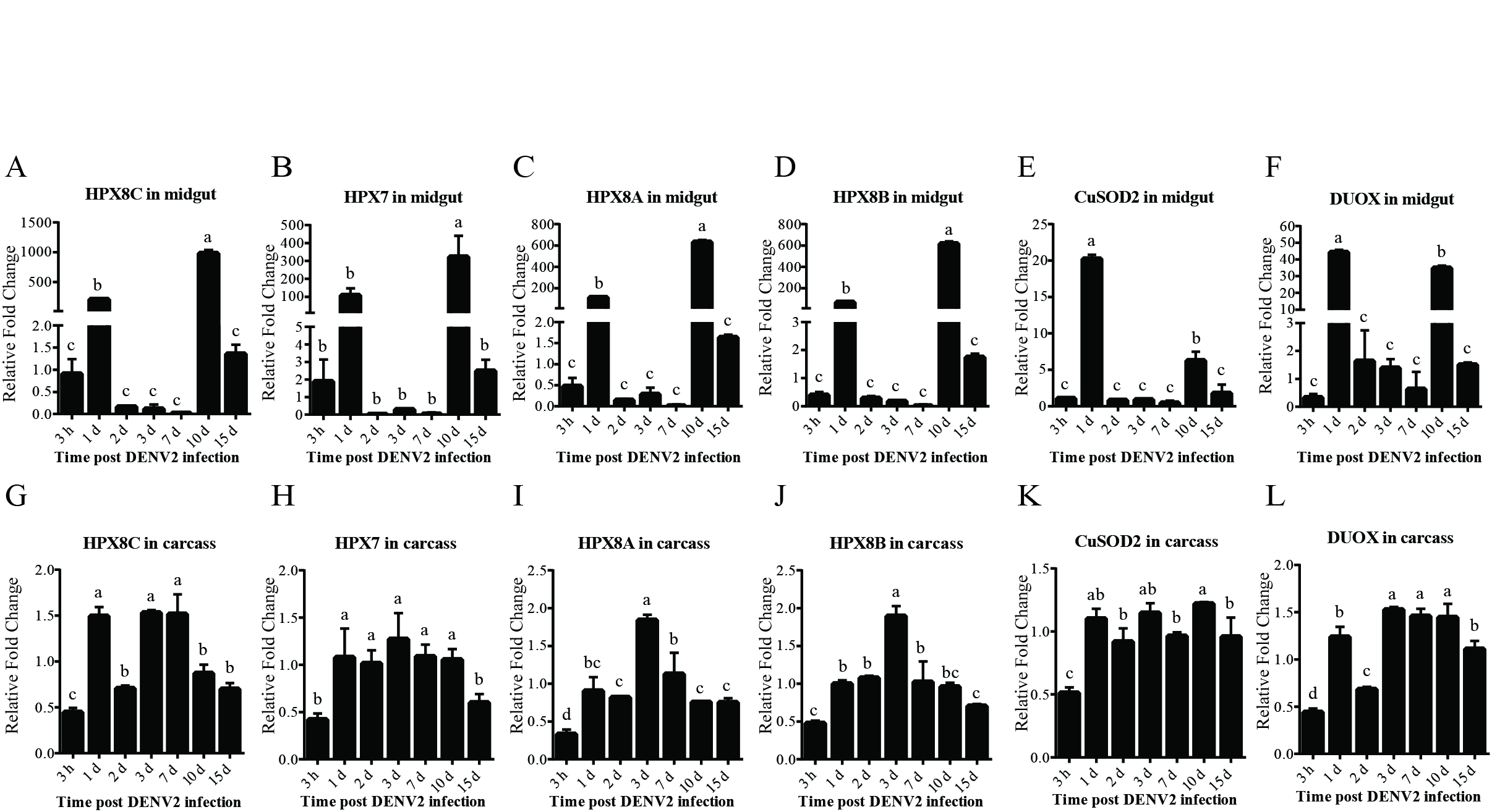

Supplement: S3 Fig — mRNA levels of HPX8C(A), HPX7(B), HPX8A(C), HPX8B(D), CuSOD2(E), DUOX(F) were detected using qPCR post 106 Pfu /ml viral infection in Ae. aegypti midgut. The mRNA levels of HPX8C(G), HPX7(H), HPX8A(I), HPX8B(J), CuSOD2(K), DUOX(L) were detected using qPCR post 106 Pfu/ml viral infection in Ae. aegypti carcass. Total RNA was isolated from the midgut or carcass of mosquitoes at seven time points post viral infection. The control is healthy BALB/c mouse blood mixed with RPMI 1640 medium. Identical letters are not significant difference (p > 0.05), while different letters indicate significant difference (p < 0.05) determined by one way ANOVA followed by a Tukey’s multiple comparison test. All experiments were repeated in triplicate. Data are represented as mean ± SEM. (TIF) [file pntd.0007287.s006.tif]

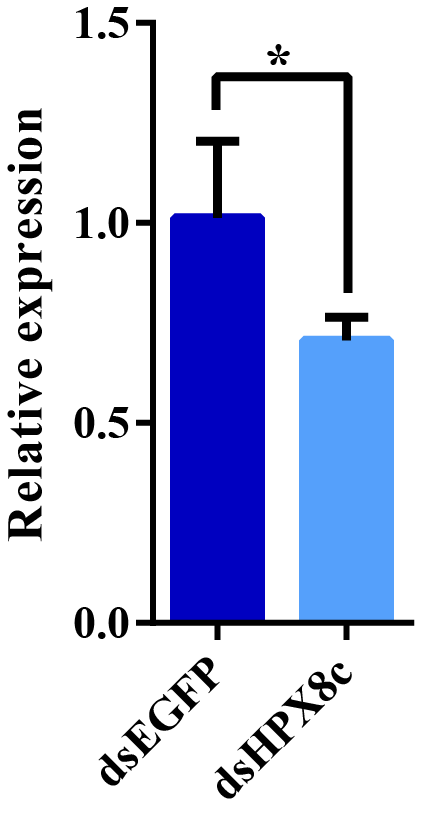

Supplement: S4 Fig — HPX8C mRNA expression at 1 day PBM in mosquitoes carcass injected with dsEGFP or dsHPX8C. All experiments were repeated in triplicate. Student’s t-tests were used to determine the significance of difference in expression between treated and control groups. Data are represented as mean ± SEM. * p<0.05. (TIF) [file pntd.0007287.s007.tif]
